# Experiences of postpartum mental health sequelae among black and biracial women during the COVID-19 pandemic

**DOI:** 10.1186/s12884-023-05929-3

**Published:** 2023-09-04

**Authors:** Megana Dwarakanath, Fahmida Hossain, Phoebe Balascio, Mikaela C. Moore, Ashley V. Hill, Natacha M. De Genna

**Affiliations:** University of Pittsburgh Medical Center, 200 Lothrop Street Pittsburgh, 15213 Pittsburgh, PA USA

**Keywords:** Postpartum depression (PPD), Adolescent, Mothers, Stigma, Equity, Black women

## Abstract

**Objective:**

The objective of this study was to qualitatively examine coping mechanisms and desired supports in pregnant and birthing Black and Biracial adolescent and young adult women during the COVID-19 pandemic.

**Methods:**

Black and Biracial participants ages 16–23 were recruited for virtual individual semi-structured interviews. Participants (n = 25) were asked about pre- and post-natal experiences with the healthcare system, effects of the pandemic, and participants’ experiences of or desires for ideal care within the healthcare system. Interviews were transcribed verbatim and coded for qualitative analysis using nVivo. Discussions around postpartum mental health evolved organically when asked about how participants were coping postpartum.

**Results:**

Nearly half the interviewees organically reported mental health symptoms consistent with postpartum depression (PPD) during questions regarding their postpartum experience. Of the 11 interviewees who reported mental health symptoms consistent with PPD, 2 were afraid to disclose their symptoms to a healthcare provider due to fear of child protective services involvement and their belief they would be treated unfairly because of their race.

**Conclusion:**

Clinicians who care for Black and Biracial adolescent and young adult mothers must be particularly attuned to structural barriers for appropriate screening and treatment of postpartum depression. Expanding investigations of intersectional influences on young mothers’ perinatal health and PPD are needed.

**Supplementary Information:**

The online version contains supplementary material available at 10.1186/s12884-023-05929-3.

## Introduction

Adolescence is already a period of significant social, emotional, and physical transition, to which the addition of motherhood can create further stress. Depressive symptoms are noted to have higher incidence among pregnant adolescents and young adults (AYA) compared to their nonpregnant peers [1], in part due to age related stigma [2, 3]. In fact, individuals who transition to motherhood at younger ages report more depressive symptoms [4]. Young mothers must also navigate the new role as a parent within an existing structure of peer relations with peers that may not have similar experiences and responsibilities [5]. Adolescent mothers may be at increased risk of facing socioeconomic challenges including lower income, single parent status [6] as well as social and emotional risk factors such as intimate partner violence, family conflict, low self-esteem and fewer social supports [7]. These risk factors may be particularly compounded by structural inequities and racism, as literature has demonstrated that low income Black and Hispanic women in the general population may be more likely to suffer from postpartum depression [8]. They are also less likely to initiate postpartum mental health care as well as follow-up treatment for postpartum depression [9].

During the COVID-19 pandemic, rates of postpartum depression escalated dramatically. According to one meta-analysis, the prevalence of postpartum depression was 34% during COVID-19, compared to 10% in developed countries and 21–26% in developing countries prior to the pandemic [10]. Several risk factors associated with postpartum depression including fewer social supports and lower income with unemployment [10] likely became exacerbated during the pandemic. This may have contributed to the the marked increase in depressive symptoms, especially for minoritzed women disproportionately affected by the pandemic [11]. Pregnant Black women have reported more stress, financial strain, and concerns about their medical care during the pandemic compared to pregnant non-Hispanic white women [12–14].

With the multiplicity of risk factors that predispose minority mothers to postpartum depression, understanding and intervening to treat mental health sequelae of adolescent pregnancy is paramount for both maternal and infant outcomes. Unaddressed PPD could have potentially devastating consequences for both infants and mothers including poor neurodevelopmental outcomes in infants, and low self-esteem, intimate-partner violence, suicidal ideation, difficulties with parenting skills, substance use disorder, and persistent mental illness after the postpartum period in mothers [15]. Underreporting of postpartum depression, especially among Black mothers, is known more broadly in the literature.

The results of one study with low-income Black mothers in New York City suggest that this population may be underdiagnosed and undertreated for postpartum depression. In this pilot intervention to prevent depression among the general population (i.e., not specific to adolescents and young adults), clinical social work staff noted that patients were reluctant to acknowledge the impact of stress and mental health on screening tools, due to stigma as well as not having practice and a disconnect between how patients might express mental health concerns and how providers ask about or interpret answers. One of the social workers in the study stated that patients were concerned that disclosure about mental health issues would lead to child protective service involvement. Another social worker noted that participants would screen negative on a PHQ-9, only to disclose symptoms of stress and concern after building trust with a provider. Only 15% of participants scored 10 or above on the PHQ-9 during the time period of the intervention, a low percentage given the financial stressors and race-based discrimination that impacted the sample. Interestingly, in the same intervention, many patients deferred services for postpartum depression, citing that they could handle the stress or had support from their church. They may have also deferred it due to mistrust or negative community experiences with mental health systems. Only two of the 30 eligible participants for intervention followed up with the program [16].

As underreporting of PPD may be high among low-income Black mothers, it is paramount to better understand the experiences of Black adolescent and young adult pregnancies, particularly those demonstrating symptoms of PPD, and to determine barriers to treatment. This is critical to ameliorating gaps in care for the most vulnerable patients during a challenging life transition. Therefore, the objective of this study was to qualitatively examine the pregnancy experiences of adolescent and young adult Black and Biracial women during the novel coronavirus pandemic in one geographic region, to assess coping mechanisms and desired supports.

## Methods

### Setting and context

This qualitative study was conducted as a secondary analysis of an existing project, the YoungMoms study (R01DA046401), a longitudinal cohort study designed to examine perinatal cannabis and tobacco co-use in young people and associated infant outcomes. The purpose of the secondary analysis–which was conducted as the larger study continues–was to qualitatively assess the impact of COVID-19 on the experiences of young Black and Biracial mothers, particularly regarding substance use, which was being evaluated in the larger study. Pregnant persons ages 13–21 were recruited at or before a prenatal visit. Participants who completed the baseline survey and were < 14 weeks gestation were recruited for the longitudinal study. YoungMoms participants who identified as Black or Biracial in the survey but who were not enrolled in the longitudinal study were recruited (by telephone, email or text message) to participate in 45–60-minute semi-structured, in-depth interviews for this qualitative study. IRB approval was obtained after review by the University of Pittsburgh Office of Research Protections. We followed Consolidated Criteria for Reporting Qualitative Research (COREQ) guidelines [17].

### Interview guide development

The interview guide was developed by the PI of the YoungMoms study in collaboration with a Co-Investigator with expertise on Black women’s reproductive health (AH) and further refined with feedback from the interviewers (ND & AH). Open-ended questions focused on the impacts of structural and racial discrimination as well as the COVID-19 pandemic on the pregnancy and postpartum experiences of Black and Biracial adolescents and young adults (see Supplement). Biracial interviewees identified as Black and one other race, and were included in the study as noted to have experiences of being seen as Black in the healthcare system. Substance use was not an inclusion criteria for the parent study or the secondary analysis. Participants were asked about pre- and post-natal experiences with the healthcare system, effects of the pandemic (including social isolation, vaccination status, and infection; coping mechanisms and substance use); obstetric racism; and participant-driven notions and experiences of ideal care within the healthcare system. Interviews lasted roughly 45–60 min. Although not the intended purpose of this investigation at inception, many of the participants (n = 11) described symptoms concerning for postpartum depression organically as part of their narrative regarding their postpartum experience, though no formal screening of postpartum depression was conducted. The first questions in the guide were used to establish rapport with the participant and elicit stories about the most recent pregnancy. At the end of each interview, participants were asked open-ended questions to give them the opportunity to share further questions, concerns, or stories that we might have missed. It was in this open-ended part of the interview that we noted so many narratives noting participants’ struggle with postpartum mental health sequelae. Many of the responses regarding postpartum mental health sequelae were noted after the interviewer posed the question “How was your postpartum?” Of note, it was posited by a community member who also reviewed this paper that in some communities, *postpartum* can be colloquial for *postpartum depression* which may have precipitated narratives around postpartum mental health sequelae. This created the desire to further capture and understand the postpartum experiences, specifically around why they may have hesitated to bring up symptoms concerning for postpartum depression and/or seek care.

### Data collection

A research team member recruited eligible individuals by telephone, provided details about the study, and answered any questions from those interested in participating. Participants provided verbal informed consent and were offered $50 on a gift card for their participation. Interviews were conducted remotely over Zoom by two interviewers (F.H., M.D.). A clinician and a researcher with experience conducting qualitative research conducted the interviews (F.H. and M.D.), which allowed for a balance of interviewing strategies and approaches in the semi-structured interview format. The interviews were recorded and transcribed with the Zoom transcription feature. Interviews were then de-identified and the transcripts were manually edited by a student and two of the authors (MM, FH) to reflect the audio recording. Interviews were conducted until the research team concluded thematic saturation was achieved. Thematic saturation was achieved when both separate coding teams agreed that interview themes started to become redundant and could be sufficiently captured by the existing codebook. Demographic data were collected from the survey for the parent study.

### Data analysis

The research team used thematic content analysis to identify major themes and subthemes, with the preliminary codebook developed from 5 interviews. Thematic content analysis is a methodology of qualitative descriptive research which involves the systematic process of examining an interview text for themes, developing descriptive terms for those themes, and coding the text under those themes. The description and interpretation of participants’ narratives are subjective, but some researchers believe this allows for less inference in interpreting the interview text [18].

The process also relied on inductive analysis, where the codes were developed from the participants’ own words rather than a pre-prescribed set of codes that were then applied to the text [19]. Two teams of investigators (F.H. and M.D; P.B. and M.M) coded the interviews separately using qualitative analysis software (NVivo) Code books were then compared for agreement and finalized through consensus, with iterative refinement of the codebook as more interviews were completed and coded.

**Table 1:** Participant characteristics

Age (years)	n
16–17	3
18–19	6
20–21	7
22–23	9
Race	
Black	22
Biracial (Black and one other race)	3
Pregnancy History	
First pregnancy	16
First birth	9
Still in high school	6
Employment	
Part or full-time employment	6
Not employed or enrolled in school	13
Home Life	
Live with parents	10
Live alone (with child/ren)	9
Living with father of the baby	4
Living with another relative	1
Other (unspecified)	1

## Results

We interviewed 25 study participants (88% Black, 12% Biracial) who ranged from 16 to 23 years of age at the time they participated in this study (March-July 2022). Within this range, 3 participants were between the ages of 16–17; 6 between 18 and 19; and 16 between 20 and 23. This means that approximately 1/3 of our participants were interviewed when they were considered teenagers (between the ages of 16–19), and the majority of participants (n = 32) were in early adolescence (between the ages of 18–24). This was the first pregnancy for most participants (n = 15). Additional sociodemographic characteristics are presented in Table 1. Out of the eleven participants reporting symptoms consistent with postpartum depression, only two participants reported telling their health care provider about these symptoms, and only one reported that they were subsequently connected to resources. Three participants specifically reported fear of child protective services as a reason for not reporting.

Four major themes were delineated using inductive analysis on the narratives of participants: (1) Symptomatology Consistent with Postpartum Depression (2) Self-Blame Around Postpartum Mental Health Sequelae ; (3) Fear Admitting Postpartum Mental Health Sequelae to a Health Care Provider, and (4) Social Support and Affirmation as Critical Elements in Coping with Postpartum Mental Health Sequelae, represented in Table 2.

**Table 2:** Themes from Qualitative Interviews with Participants

**Symptomatology Consistent with Postpartum Depression**	Q1. “But when I say that I don’t know what I mean… I don’t know if I mean like just literally physically, stop doing everything like just stay in one spot, I don’t know if that means that I wanna maybe pack my own bags up and run away from all of my problems or your problems or life. My life. This life. You know I know genuinely you know I don’t wanna harm myself. I don’t want to harm my children at all, but I just sometimes wish that I could go into a zone.” Q2. “You’re probably helping me right now, but it’s like damn, just in a little bit- I always have you tell people if I use a timer in my head that’s ticking every time I turn around, I gotta do this. I’m on this. I got- and I’m just- I’m tired of being tired. Q3. “So it’s like, it’s a lot, and I just hope that anybody that does go through it just is, strong, clear-minded, level, because it’s a lot, but the baby is, the baby is what makes postpartum disappear. Okay, that’s the best part. That’s the prize at the end. And when I look at them, I can forget all about everything.”
**Self-Blame Around Postpartum Mental Health Sequelae**	Q4. “That’s where my mental frustration comes from, because I’m tired of complaining of being tired, because generally I how I feel I should not be physically tired of… of doing- of doing a routine that I’m comfortable with doing, the routine that I set. You know my frustration comes from, you know- Just the type of person that I am, I- I’m talking and working. I’m trying to learn how to be selfish and just tackle and do what I gotta handle and do, because I feel like I get overwhelmed when I’m taking on so much-” Q5. “I wasn’t thinking about like killing myself or anything like that, but I would just feel down, think about like “Am I good enough to be like my son’s parent?” like his dad is not around so you know, I was just, I was just thinking about that too much in him having a good life with just having me so that would like put me down a little bit so that’s what caused my postpartum.” Q6. “Postpartum, I didn’t believe it was real at first, I really just thought it was just some way that moms and women just made an excuse just to be, in my eyes, just be the bad person they were, or to let the feelings out that they had. It took me to, to be to go through it with my first child and start to, I feel it sometimes because I don’t have really bad postpartum, but I did go through postpartum, and I’m going through it again now.” Q7. “He honestly didn’t even know I had postpartum just because I was crying so much by myself in the room. When he did find out I had it, he was more so, “Why aren’t you coming to me and confiding in me?” I just had to pull myself out of it because I just wanted to deal with that myself.”
**Fear Admitting Postpartum Mental Health Sequelae to a Health Care Provider**	Q8. “Actually, because my sister, I have a lot of siblings. My sister, [name], ended up doing it. but they like- they ended up putting her in like, what is it called like? Not a psych ward, but like basically where you go and they like it’s like whenever you’re dealing with depression basically and that’s what they did to her I just don’t know the name of the hospital or whatever, and they did that to her. But that was about 2 years ago. I don’t know if they’ve changed it. But I dealt with CYS my whole life and I’m just afraid of, you know that coming again I can’t deal with that. I- I just can’t deal with that. I’m scared. I’m just scared.” Q9. “But when it would come to me like going to his visits, you know they have the papers that talks about postpartum depression. I’ll just put I’m okay for all of them because I was scared that they were gonna take my child or something.” Q10. “Cuz- I don’t know- I didn’t want them to think we- usually, when some moms get depressed they think they’re going to hurt your children and stuff like that, so it was just-I’mma be honest, because I’m black I thought that-, if I told them that I’m depressed that they will try to take my daughter from me and I didn’t- I just didn’t want that risk.”
**Social Support and Affirmation as Critical Elements in Coping with Postpartum Mental Health Sequelae**	Q11. “I don’t like to think about it but I get reminded every day like damn, you know, [I] had the babies and I’m by myself. Just one thing you know, feeding them every day by myself is one thing- washing them up because that’s something that I was doing. I’m used to doing, I was gonna do that, but it just like- like damn every single day like I’m here by myself. I’m doing this by myself, you know, that sucks!” Q12. “My mom always told me, my grandma always tells me, anybody around me that I have around my son, they would tell me like you’re a good mom you’re doing real good for his age like, I was getting that a lot. Especially when my postpartum that’s another thing that like uplifted me like just be[ing] told I’m a good mom without having any help, except for my mother.” Q13. “I had a lot of postpartum depression, only because it was like I’m a young mom, I didn’t know what I was—I knew what I was doing because I had past experience of kids living with me. It’s just by myself, like I’m home with the baby—not by myself; I had him too, and he was a lot of help—”

### Theme 1: symptomatology consistent with postpartum depression

Despite the interview guide not explicitly asking about postpartum depression, of 25 participants, 11 described symptomatology consistent with postpartum depression while describing their postpartum experiences. The symptoms of postpartum depression, based on the commonly used Edinburgh Postpartum Depression Screen, include anhedonia, self-blame, feelings of being overwhelmed, increased tearfulness, anxiety, difficulty coping, difficulty sleeping, and suicidality. Mothers are generally screened at the six-week postpartum visit, a visit for which only 40% of mothers return [20]. With the additional burden of the pandemic, Black women–already noted to have lower attendance rates for postpartum visits compared to white counterparts while also showing slower recovery for attendance as the pandemic impacts subsided [21], signifying those opportunities for Black women to be appropriately diagnosed and treated for mental health sequelae related to pregnancy and postpartum has been further diminished by the pandemic. One of our participants [Q1] described wanting to “run away from it all.” The same interviewee later reflected that the interview had given her the opportunity to express how exhausted she was, describing her fatigue and feelings of being overwhelmed, both of which are assessed in screens for postpartum depression. A second participant went so far as to say that she suffered from postpartum, a colloquial term for postpartum depression and did not believe the phenomenon was real until her own struggle [Q3]. She also is quick to say her child was the antidote to the depression, calling her child the prize for her struggle. This may be the result of embedded cultural expectations that may make both disclosing postpartum depression and seeking care difficult as it means not acknowledging the gift of one’s child.

### Theme 2: self-blame around postpartum mental health sequelae

A second theme was participants’ self-blame around feelings consistent with postpartum depression. The notion that postpartum mental health sequelae was the fault of the mother and–sometimes by extension–addressed by the mother alone–was noted among participants. As noted earlier, one of our participants did not believe it was real until she experienced it herself, even acknowledging that she saw it as a personal failure among [Q6]. Even in the endorsements of symptoms consistent with postpartum depression, an interviewee stated she did not disclose her tearfulness to her partner, wanting to“deal with it herself.”

One of the interviewees expressed her frustration around postpartum depression symptoms of fatigue and feeling overwhelmed despite being a single mother of three children [Q4]. She even expressed disappointment in her exhaustion, stating “I feel I should not be physically tired of doing a routine that I am comfortable with doing.” The challenges of single parenthood, and the self-doubt surrounding one’s ability to meet those challenges was expressed by another participant [Q5], who asked herself whether she was good enough to be her son’s parent–whether she alone could provide a good life for her son. This was the same participant who did not believe postpartum depression was a real entity until she started to have experiences of postpartum depression herself [Q6]. She even noted that she conceived of postpartum as a reflection of character, stating “I really just thought it was just some way that moms and women made an excuse to be, in my eyes, the bad person they were.”

Another participant reported not even being able to tell her partner due to the desire to “deal with that myself [Q7].”

Lack of comfort disclosing depression to social supports might have increased the intensity of emotion and prolonged and enhanced feelings of isolation for this young mother. It may also be a further suggestion that the participant also understood postpartum mental health sequelae to be a personal problem.

### Theme 2: fear admitting Postpartum Mental Health Sequelae to a Health Care Provider

A third theme was fear around reporting symptoms consistent with postpartum depression to a healthcare provider. Two participants specifically reported concerns around their healthcare team alerting child protective services of their depression and risking their child or children being removed from their care, and one in particular, noted her race as a reason for increased propensity for reporting. One of our participants [Q8] related an anecdote where her sister reported symptoms consistent with postpartum depression and was eventually hospitalized–with her child placed in state custody. The intersecting histories of the participant’s sister’s experience and her own experience with child protective services in her own childhood created a web of causality for this patient who saw disclosing postpartum mental health sequelae as a reason for the medical system to report to child protective services. Another participant [Q9] admitted that she did not answer the questionnaire honestly due to fear of child protective service involvement, which likely impacted her ability to receive mental health support and may reflect broader concerns about honest disclosure around mental health concerns in the context of postpartum care. Race was also specifically cited by a participant [Q10] as a reason for increased child protective service involvement when one opted to disclose symptoms consistent with postpartum depression.

**Participant 2**.

### Theme 4: Social Support and Affirmation as critical elements in coping with Postpartum Mental Health Sequelae

The fourth theme was that social support and affirmation appeared to have a positive effect on coping with postpartum mental health sequelae, while the absence of social support and affirmation had a negative effect.

One participant spoke candidly about her exhaustion parenting three children as a single mother despite her own mental fortitude [Q11], stating “Damn every single day…I’m doing this by myself, you know, that sucks!” This same participant also noted that our interview was a unique opportunity to disclose her frustrations, telling the interviewer “you’re probably helping me right now. [Q2]”.

Another participant, also a single mother, explained that she received positive affirmations from her mother and grandmother, which helped to improve her moodpostpartum [Q12].

A third participant cited her partner as important support, assisting her to contend with her depressive symptoms [Q13], despite her reluctance in disclosing her mental symptoms to her partner initially [Q7]. Her partner supported her with caretaking responsibilities, which potentially dissipated some of the feelings of being overwhelmed expressed by single parents [Q2, Q11].

## Discussion

Our interviews reflect a small number (n = 11) of Black and Biracial adolescent and young adult mothers who organically disclosed symptoms concerning for postpartum depression often not captured by traditional screening tools or noted by a medical provider. Additional themes noted in the interviews reflect that postpartum mental health sequelae are often conceived of as personal failure; reporting postpartum mental health sequelae can be associated with a negative outcome of referral to child protective services; and social supports–either family or partner support–have a positive impact on managing postpartum mental health sequelae while absence of familial or partner support can worsen feelings consistent with postpartum depression.

While difficult to extrapolate towards broader implications for screening, diagnosing, and treating postpartum depression, the themes captured may give some opportunity to reflect on some of the structural barriers for young Black and Biracial mothers to report postpartum mental health sequelae to providers as well as consider ways to support this vulnerable population at a particularly challenging juncture of motherhood. From broader data, we know that racial disparities exist both in the prevalence of PPD and the receipt of postpartum care, with Black and Hispanic women having higher rates of PPD [8] compared to white women, and less connectivity to care [9]. While not a formal purpose of this qualitative study, it is also notable that our participants were part of a recruitment effort to characterize experiences of substance use disorder screening in obstetric care—which may have been a confounding factor.

Other qualitative studies have also shown similar prevalence of mental health concerns characterizing the perinatal and postpartum experiences of Black women. In a qualitative study by Barnett et al., self-identified women of color were interviewed in focus groups where themes were similarly identified via an inductive and iterative approach [22]. The study reported that mental health was a common theme among the focus groups, with many of the participants reporting postpartum depression. They also noted the need for mental health resources including screening and diagnosing postpartum depression appropriately.

The preponderance of unprompted discussions around postpartum mental health sequelae noted in nearly half our subjects suggests that Black and Biracial adolescent and young adult (AYA) mothers are experiencing depressive symptoms but may be under-reporting struggles with mental illness to clinicians and on screening forms. This finding is consistent with qualitative meta-analysis study by Maxwell et al. [23] where the pressure to “keep it to myself” was reflected among the narratives of marginalised groups more broadly. In this study, the pressure to not disclose postpartum mental health sequelae was notably a consequence of non-Western cultural pressures or not having sufficient language in non-Western cultures, more than stigma or self-blame. .

In our interviews, mothersmore often attributed their depressive symptoms to personal weakness rather than illness, which created stigma around disclosure. Our participants were also quick to shift blame away from their children as sources of pressure or reasons for their feelings. Social pressure for new mothers to bear the physical and mental sequelae of childbirth are noted in other qualitative studies [23] which discuss idealised mothering, but the nuances of how this may manifest in Black and Biracial adolescent mothers are less explored. In one study, Black mothers were asked “what do you do when you feel down in the dumps?” and the overwhelming majority, 63%, employed strategies that typically denied, masked, or suppressed their emotions rather than strategies which acknowledged symptoms, treating causes, or seeking professional help [24].

Additionally, the mythology around the “strong Black woman” (SBW) may be implicated in the expectation that Black women bear their mental illness alone. A qualitative study by Hall et al. [25] noted that higher levels of SBW schema endorsement were associated with higher levels of psychological distress including depression, stress, anxiety, and suicidal behaviors. Moreover, another qualitative study interviewing eight focus groups of Black women noted that some of the distress of the SBW schema was caused by restriction of help-seeking behaviors and attending to others’ needs over one’s own. TheSBW schema which may predispose young Black women to minimize postpartum mental health sequelae may be exacerbated by the intersectionality of being a young Black mother in a culture which does not often favorably view Black mothers or young mothers and can further expectations for young, Black mothers to be “superhuman” to contradict known stereotype threats. This makes capturing information around postpartum mental health sequelae for this population extremely difficult, though our experience interviewing our participants certainly revealed this to be a substantial and unmet need. Pittsburgh Healthy Start and the Infant Health Equity (IHE) Coalition, which includes local community perspectives and experiences, have suggested replacing or supplementing paper mental health screenings with one-on-one trusted maternal and child health workers, which aligns with the narratives of several of our participants [26].

It is imperative that mental health researchers and professionals develop targeted strategies that improve the comfort and remove barriers that dissuade young Black and biracial mothers from disclosing postpartum mental health sequelae and to normalize seeking appropriate treatment. Strategies that may be helpful considering the themes identified in this study would be greater inclusion of Black and biracial doulas, who are often seen as non-judgmental advocates, during pregnancy, as well as continued screening for postpartum mental health sequelae well beyond the six-week postpartum checkup. Additionally, as many birthing people forgo their six-week checkup, pediatricians may be critical advocates and touchpoints for rescreening of postpartum depression in mothers further out from the infant’s birth. Additionally, it may be important to identify community support, as many participants in this study reported social isolation as a result of being a young mother. Centering groups, which offer birthing people a cohort of peers during their pregnancy as well as new parent support groups, cancreate a community for young mothers navigating their new roles. Other interventions like early intervention and nurse visiting programs have also demonstrated efficacy in addressing disparities among adolescent mothers [27].

### Strengths and limitations

A primary strength of this study is that the participants were already enrolled in the broader YoungMoms’ study and had developed a longitudinal relationship with the clinical team prior to their childrens’ deliveries. This longitudinal relationship between the participants and the study team may have created a level of trust that facilitated the more candid disclosures of postpartum mental health sequelae. An additional benefit of our study was that many participants were no longer in their first year postpartum period at the time of the interview; this distance from the timing of their birth may have increased comfortability in reflecting and disclosing postpartum mental health sequelae as something they had overcome. There were also some limitations, including the lack of inclusion of questions about perinatal mental health and postpartum depression specifically. Another limitation was the lack of intersectional analysis and fewer number of Biracial participants to determine differences between Black and Biracial participants. the dearth of research on postpartum depression in Black and biracial AYA, more research is needed to better understand how to meaningfully care for this population.

## Conclusion

Black and biracial adolescent and young adult mothers may under-report depressive symptoms in the postpartum period due to structural and social barriers. Eliminating these barriers and improving the acceptability of reporting depressive symptoms for minority young women after experiencing pregnancy is vital to improve perinatal and infant health overall. The narratives and insight from participants in this study suggest the need to better understand social and historical reasons why Black and biracial AYA mothers may be unlikely to report postpartum depression to develop appropriate interventions to support a healthy pregnancy and postpartum period.

### Electronic supplementary material

Below is the link to the electronic supplementary material.

Supplementary Material 1

Supplementary Material 2

Supplementary Material 3

Supplementary Material 4

Supplementary Material 5

Supplementary Material 6

Supplementary Material 7

Supplementary Material 8

Supplementary Material 9

Supplementary Material 10

Supplementary Material 11

Supplementary Material 12

Supplementary Material 13

Supplementary Material 14

Supplementary Material 15

Supplementary Material 16

Supplementary Material 17

Supplementary Material 18

Supplementary Material 19

Supplementary Material 20

Supplementary Material 21

Supplementary Material 22

Supplementary Material 23

Supplementary Material 24

Supplementary Material 25

Supplementary Material 26

## Data Availability

All data generated or analyzed during this study are included in this published article and its supplementary information files in the form of tables. Raw manuscripts of the qualitative interviews themselves are included in the supplementary file.
