# Supplementary material for: Experiences of postpartum mental health sequelae among black and biracial women during the COVID-19 pandemic
Source: BMC Pregnancy Childbirth. 2023 Sep 4;23:636. doi: 10.1186/s12884-023-05929-3 (PMC10478375; doi:10.1186/s12884-023-05929-3)
Supplement: Supplementary file 1 — Supplementary Material 1 [file 12884_2023_5929_MOESM1_ESM.docx]

**Supplemental File 1.3 Interview Transcript with Participant 5316**

Interviewer: SS

Time: 45:12

I: Okay, how's your pregnancy going so far?

P : It's going… good mentally. Um, physically, I’ve been real nauseous lately. So, it's been kind of hard on that end, but it’s going okay.

I: Yeah, what have you found to help with that? Has there been anything?

P Um, sleep. When you’re asleep you can’t throw up, so just sleep.

I: Yeah, how have you- how is mentally, I guess, been for you? Like how have you been staying positive in that way?

P : I have and I’m really excited. I think nothing but good things about me bringing a human into the world and I try not to, you know, let myself get into any depressive state. Because I know what I'm doing is beautiful and it’s life, so I'm excited.

I: Yeah, that's a great mindset to have going in. So, jumping into the meat of the interview, what are your thoughts about marijuana use in general?

P: I don't have anything wrong with it. I used it and see nothing wrong with it at all.

I: Tell me more about your experience with it.

P: Um so I used it. I used it to, honestly, as a like, um- as a relaxer because I’ve had anxiety. I have anxiety. I have social anxiety and just anxiety period. And so when I use it, it tends to calm me down, bring me back to this ease state of mind.

I: When did you first start using it?

P: I started using it, faithfully, when I turned about 17 maybe. And I would use it like not a lot, but I was losing a lot of weight. Not too bad, but I was going through like weight loss, so I was kind of losing myself. So, I would turn to marijuana, and it would make me feel better and it would make me eat. Because I definitely had an eating disorder, so it would make me eat and gain myself back.

I: Is that something you would normally use socially with friends or just alone, for stress?

P: Alone.

I: And do you still use it now?

P : Um, here and there. Maybe once a week.

I: Gotcha. Okay. What are your thoughts about tobacco in general?

P : I don't like it. I don't like cigarettes, anything, cigars, vapes. I don’t do those.

I: Why don’t you like it?

P : I personally don’t like the smell of it. The smell of cigarettes or anything in that use. I feel like it's not healthy. So, I personally don't do it.

I: What do you think about the use of tobacco during pregnancy?

P : Um, I feel like it’s no other than marijuana. It's just different smells. Maybe different side effects but, um I can't say I don't think people should do it because that’s like me saying I don’t think people should smoke weed. It's some people's coping mechanisms or maybe it helps them with nausea, you know?

I: mm hmm. Yeah. What side effects have you heard, um, of for tobacco versus marijuana?

P : Um, I haven't heard any, actually. The side effects that I’ve heard from marijuana… I haven't heard any actually. I just know like brain development or something like that, but I haven't heard, like any differences.

I: Mhm.

P: I don't even know if there are, but I'm pretty sure because tobacco is- is-. Is it more dangerous than marijuana? I feel like it is.

I: Um, there’s differences. Part of why we're doing this study is to really learn about marijuana and tobacco and what effects they can actually have, so.

P: Oh, okay.

I: Yeah, um, so you mentioned brain development with marijuana tell me more about that. What you've heard about that.

P : Um so my recent appointment I told my doctor that I do use it, just to help me because I can’t eat on a regular daily basis without feeling nauseous. And my one doctor was telling me how it can affect brain development and do something with like the brain cells and she-. I don't remember exactly what she was saying, but I know she was telling me like something about the brain cells and how- or the baby may be smaller if I smoke a lot of it or continue to smoke it throughout my whole pregnancy, just things about like the growth of the baby may not regular.

I: Gotcha so, she was saying it might be negative?

P : Yeah, it might- yeah, yeah.

I: What did you think hearing that?

P : Um, if I'm gonna be honest, I didn't really take it in fully because when I went to talk to another doctor, she kind of made me feel more comfortable about it and was telling me how it's in a gray area. So, nobody really knows about how it really can affect the baby because it hasn't really had a huge effect on people's child- on people's kids. So. And then, I know a couple people who do smoke marijuana while being pregnant their baby is completely fine. So. Kinda really didn't, you know, catch me like that yet.

I: Yeah, did you do more research on your own like after the appointment or talk to other people after hearing those two, kind of, conflicting opinions?

P : Um I didn't do any research. I have- I don’t know if you’ve been on TikTok. But I have seen a couple of Tik-Tok video about it, and some people say it's okay and there's nothing wrong with it, as long as you do it responsibly and do the right kind of wraps for the marijuana. Because it can also depend on the wraps like if we're using regular blends what they call it. With tobacco blends then it may be worse, because it's so harsh and there’s still tobacco on the leaf. But if you would like a regular paper or raw papers, another word for it, then it's healthier. So, it still may have the same effects, but it's a healthier type of way of smoking it.

P : I know a couple people, like I said, who do it. And they told me there's, there's nothing wrong with it, just stop early like and get myself off of it, rather than just stopping immediately because my body is going to react differently than just stopping, like slowly stopping. Because I went from smoking every day to smoking every other day to smoking every week. So, I feel like it's working.

I: When you say people told you to like stop early what do you mean by that?

P : Stop early in my pregnancy, so don't smoke throughout my whole pregnancy. Which I wasn't planning on doing anyway, but just not smoking throughout the whole nine months. Maybe stop the third month or the fourth month.

I: What benefit have they told you that will have?

P : So just that it won’t- cause it takes three months for marijuana to get out your system, so if I were to have my baby and I still was smoking and they found marijuana in my baby system They can call CPS so, then I may at a risk of losing my child or just the development. I don't want my baby to have any other issues and I don't want to be that that 1% or that that just so happened with me. You know? So, I’d always rather be safe than sorry.

I: yeah. How do you think that, like doctors find it in your baby system, like how do they know that you've been using it in the delivery?

P : So, it stays in your system for three months, and then, if it's in your system, you know our nutrients goes to the babies or whatever we do, literally everything we do goes to the baby. So, they test the baby. They test the baby for all kind of drugs when you have the baby just to make sure they have no alcohol, or they're not addicted to anything. Because some people do, you know, harder intense drugs while they're pregnant. So, they tend to test babies or make sure that, you know, they’re not addicted to anything they're born. I know they do that.

I: Okay and is it your friend that told you this?

P : This is a family friend yeah.

I: Family friend. Okay. How do you- where do you think they, kind of, got that information?

P : Um, I feel like they did their research, because they have other kids so. They've done their research and just simply by having other kids they know.

I: What do you think about like child protective services being involved if, you know, someone does test positive?

P : Um, honestly ,I feel like it's not needed because it's just marijuana, you know, like it's not crack. I don't look at marijuana as a substance, as a drug. It's a leaf. It's an herb, so it's not bad. But it's not legal, so it is bad. So, if we're in a legal way, then if they see marijuana in my baby’s system and they feel like they have to take my child, then that's on me because I knew that. If I didn't have the information on that, then maybe they could have told me. But I knew that, so then it would have been like “kind of figured it was going to happen. don't agree with it, but I'm going to fight to get baby back.” It’s one of those.

I: Thank you for sharing that with me. I want to ask more you kind of told me that you had tapered off a little bit, like going from every day to every week. Is that when you found out you were pregnant, or had you already been tapering it down before that?

P : So, it's when I found out. Definitely when I found out I was pregnant, I don’t see myself using it as much. Definitely when I found out. When I first found out, I stopped completely and then I got really, really, really nauseous and I personally do not like feeling nauseous at all. I would rather have a headache all day than feeling nauseous. So, I- and I couldn't eat. I wasn’t too able to keep anything down, so I would smoke maybe one, one blunt and then I would get high. So, then I would be able to eat, and I would be able to focus and calm down without feeling sick or, you know, not feeling good in general. I definitely slowed down, um when I started. I went from every other day, when I first find out that I was pregnant, to every week. Once- so, it would be like one every other day, not like two blunts or three blunts. It would be like one every other day, and then, now I try to do like one every other week on like a certain day that I'm feeling really, really nauseous and I can’t control it. Yeah.

I: Was it hard to cut down for you?

P : It wasn’t actually. It wasn't because I haven't been doing it for that long. I just turned 18 in December. So, I haven't been doing it for that long and I don't really need it necessarily. I feel like I'm able- I’m in the right mind set that I can control my own mind and be able to… Because, you know, weed isn’t always going to be around so I’m not always going to rely on that. So, I have to learn how to control my own emotions and my own feelings without depending on marijuana. So, no, I don’t-. I feel like I can get off of it easily.

I: How have you learned to like cope with emotions and deal with stress now you're using it less frequently?

P : Um, just getting myself out. I'm an only child, so been sheltered and I’ve been around my mom a lot. And I’ve been, um, just in the house and enjoying my own company so learning to get out with my boyfriend or go out to work or go with my friends, being able to go out and be me or be a teenager definitely helps me with- to cope with my emotions. Music, too. Music definitely helps a lot and cleaning. I clean a lot, so cleaning definitely helps with that. Yeah.

I: I guess that kind of idea of getting out of the house, being with friends, being teenagers’ kind of brings up the question for me like how was the pandemic affected things, whether the pregnancy or marijuana use for you?

P : Um, honestly during like the pandemic pandemic, so like in 2020, I wasn't smoking. Um, not as much smoking, because my mom was here and my mom doesn't condone that. So, I didn’t- she knew that I smoked, but I had never smoked around her or around the house or every time I did I had to make sure she didn't smell me. So, it didn't really affect it at all actually.

I: Yeah. When, when you first, kind of, find out you were pregnant and you said you quit for a little bit, why did you decide to quit then?

P : Um, because I didn't know. I didn't know the effects. I didn't know how my body, personally, was going to react to it or how my baby was going to react to it. I didn't know anything. I didn’t even research, I was just confused. This is my first time ever being pregnant, so I was just like “ahhh.” Like I said, I don't want to be that 1% or I don't want to be that that, that part . I just- I don't want to risk anything with myself or my baby, so that's why I stopped. And then I went to the doctor and I talked to the “doctor doctor” who sounded serious and then I talked to the “realistic doctor” who was like, like “Eh, it's not recommended, but it's not not recommended. If, you know, if you need it- if you need to eat or if you need to snack on something, then take a hit or two. But don't do it for too long.”

I: Was it hard for you to, kind of, talk to your doctors about this?

P : Yep, it was, it was because I don't like being judged. I don’t like people looking at me weird or being like “You smoke while pregnant? You shouldn’t do that.” I don’t like that. So, it was but then I have to be honest and they’re going to see it anyways because they take my pee. So, they take my drugs- they take me pee and they can see if it is in my system. So, they’re going to to know so it's like, why not just be honest with them and tell them?

I: Did your doctor ask you or did you kind of bring it up yourself.

P : She asked me. There was like a questionnaire thing, and it was like “Do you smoke tobacco?” “No.” “Any marijuana use?” “Yeah”

I: What have other people in your life told you about talking to your doctor? Have you- did you talk to anyone else first about whether you should or not?

P : Yes, so I had, um, talked to one of my cousin’s girlfriends and she has a child, and I was telling her. And she was like “Make sure to let your doctors know because some medicines you may not be able to react to the same because you use it or some medicines, they might not be able to give you because it has a side effect with marijuana.” And it is just always best to let them know because, like I said , they- they know- like they're going to find out regardless. And then they're able to tell me the safe procedures and the not safe procedures of it. They're the ones that's going to be able to see my baby in my body while I’m going through this, so I feel like it was a good thing to tell.

I: What do you think doctors can do to help young women feel more comfortable talking about marijuana and tobacco.

P : I feel like they should definitely not, um- Sorry, I feel like they should definitely not give them any- because every time I told my doctor she gave me like a weird look, like it was like a like a “Wow, you do tobacco”. So, I definitely- I mean “You smoke marijuana.” So, I definitely feel like they should give them both, both sides on it. So, like the bad sides and then also the not the bad sides, like “sometimes nothing happens, or sometimes that's how people need to eat or something or that's how you need to sleep.” So, I feel like they should definitely give them the good look on it and then “If you continue to do it, then this may happen or this may develop with your baby or with you or with us. We wouldn't be able to give you this medicine or that medicine.” But comfortably like as like a counselor, just letting them know that it's okay that they do it. Just be aware that when you do it, what can happen.

I: What can doctors do I guess before even asking the question that would help people like you feel comfortable sharing once that does come up?

P : Um, I don't know because I feel like they asked the question just as a question just as a “Do you smoke marijuana?” And it’s just like a yes or no question. It isn’t “Do you smoke illegal marijuana that can cause development of the baby? yes or no?” like it's it's literally just a question, so I feel like that, that alone if- if you do it just tell them “Yeah”. If you don’t then “no”.

I: Besides talking to your cousin's girlfriend what else, kind of like influenced you in deciding whether to talk to your doctor or not?

P : Just, like I said, just knowing that they're going to find out and they're going to see it and they're my doctor, so they have not full control, but they have control over my body, and they see everything. They know everything. They're going to be able to find out. So, I just like to be honest, just to get- because I feel like if I don't tell them and then they find out, they are going to ask me. They're gonna be like “Well, we’ve seen drugs in your system and duh. Duh duh. Duh. Duh.” And it’s just like, oops. So, I’d rather just be honest.

I: Did you have any, you know, it sounds like you're kinda pre decided to talk to them, did you have worries about it going in?

P : Um yes, I did. I thought instantly they were going to tell like, you know, if I keep doing that, I’m going to be high risk for miscarry or stillborn. I thought they were going to give it to me raw right then and there. Just like “Oh not you can’t do that. You gotta stop right now. Your baby is- you’re only going to be able to carry it- you're going to miscarry.” I thought they was going to give it to me raw, real right there.

I: It sounds like you kind of have heard some conflicting information like from your, from your family, friends - just quit early. From your first doctor- it can have growth problems. From your second doctor- it’s a little bit of a gray area. we don't know. How do you decide who to trust in this situation?

P : Um, myself. I trust myself and I trust my body, and I know my body, personally. So, I know if I need it, to the point where I literally cannot eat, then I will do it and I know if I can just handle the nausea and take the medication that I’m prescribed, then I will be good. But I can honestly just trust myself and I don't know if you believe in God, but I pray. So I just trust myself and God . I just pray and I know myself and I know that it's going to have to come to an end eventually, so I'm gonna stop and continue to grow my little baby.

I: um, what do you think there's any difference between like talking to a researcher about this kind of stuff versus talking to your doctor?

P : I feel like talking to you guys, since we are anonymous, it's more able- I am able to really, really like let out how I really feel. Because when I took the first survey, I think. I was scared at the end. Like it was questions about marijuana and I'm like “ooh.” And there was questions about my partner using it and my mom using it, so I was like, uh. Like I don't really- don't really know. Like I was kind of nervous but knowing that my identity is confidential, it definitely helps me to be more comfortable talking to you than with my doctor. She knows me. She knows my situation. She knows my body. She knows my baby. She sees everything. So, she has more access to different people and if it- if marijuana was-. If- Since they say it’s illegal, when I think of illegal I think of “you’re going to jail.” So, when I think of that- I think instantly you know well, it's illegal. Well, I feel like I’m going to go to jail or my baby's not going to be mine or my baby's going to die or something like that.

I: Thanks for sharing that with me. what what are ways you think that young pregnant woman can get more information about marijuana and tobacco use during pregnancy?

P : um I feel- I feel- So honestly… I feel like it depends on their friend group or just simply who they ask. Like if they feel comfortable around anyone. Um, a parent, I don't know I feel like you would have to be close with your parents and your parents would have to do it to understand it because my mom doesn't smoke, but I do. So, and I feel like she- and she doesn't know that I smoke while I'm pregnant. So, I feel like if I would tell her, then it will be a whole different story and it would be a whole different everything whole different argument, whole different break down, everything. But if I talked to my sister then she's able to be more comfortable with me. more- I'm able to open up with her because she knows, like she’s been through it. She understands it. So, I feel like it's best that they talk to someone where they know they can be comfortable besides like a parent, unless you're comfortable with the parent. But besides a parent, maybe a friend or their significant other. I talk to my boyfriend a lot about everything, so he makes me feel comfortable. So, any-. Just anything, anyone that makes them feel comfortable.

I: Yeah, so it sounds like personal relationships are a really important way to get information, do you think like online as a place or your doctor has a place, what else do you think is important?

P : So, I feel like certain doctors. Certain doctors. I can tell when doctors want to be a comfortable doctor. I can tell when doctors want to be a doctor. So, with those certain type of doctors, I don't. If they ask, I will say it, but I won't talk about it. But if it's like, if it's a doctor that I can tell… If they just come in, maybe crack a joke, or they come in and rub my tummy “hey baby” and talk to my baby, I know that I feel like I can be comfortable with them, then I'll be able to talk and explain and give my opinion on things.

I: Yeah, I think that's a really good point that kind of gets out what I was asking earlier like what doctors can do before they're even bringing up the question. Maybe for you like make a joke or kind of a more casual. How else can you tell if a doctor, as you put it as being like a comfortable doctor or a doctor.

P : Um, let me think. Let me think. Just being really like, I don't want to say concerned, but interested. So, asking questions like “how are you? How are YOU? How are you while being pregnant?” not “How’s the baby?” Well, “how’s the baby too?”, but worrying about you, talking to the partner, whoever's with me, about them being a new father or how he feels about being a dad and just being welcoming. Knowing that welcoming feeling, you're able to talk to them and then they let you know, like “Hey, I'm a doctor, but I’m gonna listen, like you can talk to me. You can let me know.” And then they let me know some things, like you told me, some things I can keep to myself and there's some things if I feel like I can't keep to myself I can’t keep to myself. Which is always a good thing because not knowing, the fear of not knowing it's, you know, the fear of not knowing you just don't know so getting that warning of “If you tell me about self-harm and I'm gonna have to report it because we care about you, but if you tell me you smoke weed it's fine like we're just going to see what we can do to help you with that and help your baby with that.”

I: Yeah. Let me look through my guide real quick and just see what else. Okay, so marijuana is, kind of, has been made legal in some circumstances here, medically, some states, recreationally. What do you think about that?

P : Like I said I don't see nothing wrong with it, I feel like medical weeds is more for it's not like a head high. It’s more of a body high. it's more of a to calm down that certain area that you are affected in your body. Illegal marijuana is no better because you don't know what people put in it. People could lace it. People could do anything with it, so definitely know your source. But I honestly feel like it- it- there's nothing wrong with marijuana as long as you know your source and you know what kind of marijuana that you are smoking. And how you're smoking it and who you’re smoking it with. Yeah. It all depends. I feel like there’s nothing wrong with it. I’ve never had medical marijuana and I like I said it’s more of a body high and and I’d personally rather have a head high than the body high. Just because the body high, it just, it just feels weird. But yeah.

I: Tell me more about that, like what's the- what do you mean when you say head high versus body high?

P : So, a head high is, uh, I’m gonna see if I know how to describe it ‘cause I don’t really know how to describe it. I just know what it feels like. It's kind of like the difference between CBD and THC. So, THC is what’s in marijuana-juana. Tsst Marijuana-juana (laughs). What’s in marijuana and that’s a head high. It’s more- you're more. I don't know. I don't really know how to- I honestly really don't know how to describe. It is honestly just a feeling like you, you could tell the difference in your body. Yeah, I don't know how to describe that.

I: You had mentioned, you know, the importance of knowing your source and being careful of who you’re around and stuff have you someone you know, has something bad happen or how did you hear that?

P : Actually, I just found out. My mom because my mom doesn't smoke weed at all. She doesn't like it, doesn’t like the smell, doesn't like to be around it, none of that. So, she told me a couple years ago, like in her second or first time trying it she got a lace of coke. Coke in her weed. And she was like too high. Like not just the regular marijuana high, like an actual drug high. Ever since then she has smoked no more. That’s the only person I know that had a really crazy experience with it and she doesn't like it at all. She won't even be around it.

I: I do want to get a little more into tobacco, to make sure we're covering both so some questions I kind of have there, um, have you ever tried tobacco?

P: No

I: Okay, do you think, between tobacco and marijuana, one is safer than the other?

P : Ummm, yeah, I mean I don't- I don't know because I don't like cigarettes. When I think of tobacco I think of cigarettes. Is that what you’re talking about?

I: Yeah.

P : Yeah so. Uh, I don’t know. Yeah, I don’t know.

I: What about during pregnancy specifically do you think one is safer?

P : Yeah um. I don't- I don't think people should smoke cigarettes while they are pregnant just because tobacco is more harsher and stronger than marijuana. And it's- it's- it's already bad for you, regardless, bad for your lungs, bad for your body period. So, while being pregnant, if it's bad for your lungs then it's bad for the baby’s lungs. Like something like that. Marijuana, unless it's laced, there's nothing wrong with it. Like you can't- you can't get sick from it. It can't mess up your body without nothing being in it. So yeah.

I: Where did you hear about tobacco being worse during pregnancy?

P : I didn’t actually. I just assume. And I, yeah, I just assumed because I know tobacco is bad. And I know tobacco, cigarettes itself, anything that use tobacco, vapes are bad. So, I know it's bad so it would be bad during pregnancy.

I: What do you think about using marijuana and tobacco together?

P : Oh. Um. I feel like you shouldn't do that while being pregnant. That is just like a- that's like that's a little overboard for you- for your baby and just, just yeah. I don't know. That's a little [unclear]. If you do it, then you do it.

I: What about when you're not pregnant?

P : Oh, when you're not pregnant, to each his own. If that's how you get down, that’s how you get down. But like I said marijuana, there isn't anything that I've seen medically wrong with it like you, you can’t literally get nothing in your body, unless it’s laced and there’s nothing that can happened to you with marijuana. Cigarettes, you get lung cancer. You get something wrong with your throat. Yeah. It's just- it's just bad for your body.

I: Besides kind of verbally talking to you at your appointments, did your doctor’s give you any kind of like pamphlets or websites, any other information sources to check out?

P : Yes, I have, I have books. They gave me this big fat folder at my first trimester screening. There’s a lot of information there. I haven't been through it yet because, believe it or not, I haven't really got the “mommy mindset” yet. I know that I'm a mom and I'm about to be a mother, but it hasn't hit me yet. Maybe because I don't know what I'm having, but it hasn't hit me yet so it's kind of- it's kind of- it's kind of still, still surreal to me. Because I have diapers in my room, and I still look at them and say “Wow I have diapers in my room.” Like it’s, it's crazy.

I: Is that something you plan to go through eventually or is that, just like too much?

P : Yeah, I'm definitely going to read it because I have questions, every day. I have questions every day. My body literally changes every day, without even me knowing and like I can be completely fine one day and then the next day my stomach is on fire, like my body literally changes every day. So, I definitely- I'm definitely going to go through it with my boyfriend and learn more about my body and the development of the baby and what we can do to make sure we have a healthy growing baby. Naturally, without depending on any substance, um, or any kind of medication because I have medication from my doctor, but I don't want to depend on you know medication to make me feel better when I could just simply eat an apple or something.

I: Yeah, in terms of I guess eventually quitting because you said that you know it stays in your system for three months what's your kind of plan for that?

P : Um, it’s funny because I'm stopping Friday. So, today's Thursday, I'm going to stop completely tomorrow. And then like I said I want to learn to get in the habit of not eating without it or not- like not depending on it to eat because since I started to now, I definitely needed it to eat. it's bad, but it's something that I want to get out of because when I have my baby, I want to breastfeed. I can't smoke, you know, I can’t have that in my, my breast milk. So. I’m gonna pray on it and just do the best I can and let us, let my body go through its changes and if, and if I feel like I needed it, I don’t know I’m just gonna pray on it or maybe drink some water or something.

I: Let’s see. Okay (to background: stop, lay down) Sorry, my dog’s over there digging in his bed. Okay, do you want to talk I guess, a few more things, but one thing kind of you mentioned looking at TikTok in the days after your doctor's appointment what- what role do you think like social media has in disseminating this information?

P : Nowadays. I feel like it's helpful. It's really helpful because you get different perspectives of different women. Even though everyone's body is different, you get different, different perspectives of different women and different journeys of pregnancy with and without substances. And just knowing that there is someone out there who does it and it's not just you and you're just not the only person getting high while pregnant it kinda makes you feel more comfortable, especially in today's society. I don't look at the women as- what is that word? As- I don't look up to them, as “Oh, Christie smokes, so I'm going to smoke.”, like no. it's like “Okay, Christie smokes, let me see how it affected her pregnancy or let me do my research on Christie and then maybe go Google and see if any other moms have experienced this.” So yeah, as one of those.

I: How do you know that things you see on Tik-Tok or things you read on Google are like reliable and trustworthy?

P : Everyone is on social media. Everyone. Older moms, younger moms, older dads, younger dads, everyone's on social media. So, I feel like if it's genuine and I know like this person has a baby and I’ve seen them- I’ve seen their baby and I’ve seen them like talking about it and other people in the comments can agree or disagree, then I know it’s real and I know like “Okay, this really happened.” Google… so much. Ah. Definitely- it definitely depends on the site. So whatever site I'm on and I’m researching, if it's like a medical site, I tend to stay off of those because it's a medical site so it’s a doctor. But if it's like, maybe like, a blog from a mom or something and she's just going on about how she smoked marijuana or how she smoked and the outcomes of whatever then I'll be more comfortable in there, because I know she's a mom and I know she has kids and she has experienced it.

I: What makes you want to stay off like medical sites made by doctors?

P : Um because they are doctors. Doctors tend to give “doctor answers” and they, um, do everything the medical way. So, if I go on a “doctor site”, they say “Instead of doing marijuana you should try this or if you do marijuana then all the side effects.” There's no positive things, all the bad things like you can get cancer and duh. Duh. Duh. Duh. It’s just a lot. A lot of something that you don't want to hear while are pregnant.

I: On the subject, I guess kind of like medical sites via the American college of OBGYNS recommends that doctors do like ask about marijuana tobacco use etc and tell patients to quit, what do you think about that?

P : Tell them to quit right then and there? I feel like it's, excuse my language, but bullshit. Um just quitting, it can affect your body. Like that's like you just stopping alcohol and then you go through withdrawal so then your body's affected, so it's always good to get yourself off of it. So go from- kind of like a diet. If you look at it like a diet, you want to stop eating bread, you can't- you can't just stop eating bread completely because everything has car- You want to stop eating carbs. You can’t just stop being on carbs completely, half of food has carbs in it. So, you have to slowly get yourself off of carbs, you have to slowly get yourself off alcohol, off of any other hard drug, off of marijuana. You have to slowly get yourself off it before you can just completely stop. So just telling them to completely stop it. It’s scary and it can be dangerous for the body, because you don't know how their body reacts to just stopping without just letting themselves off of it.

I: Do you know people that have tried to quit all at once, like had negative side effects?

P : No, I don't actually. I'm the first of all my friends who's having a baby, so none of my friends. My sister I know she is pregnant right now, and she she's like- she's about to give birth and she's been slowly letting herself get off of it and she's doing fine.

I: Well, where did you kind of hear that it that it's bad to quit all at once, or what can happen if you quit all at once?

P : Um, so I've heard from different people. Like my cousin’s girlfriend she vapes, and she was telling her doctor that she vapes, and her doctor told her it's best that she herself get off of it just because you don't know how your body's going to react to completely stopping. So, she went from smoking five percent vapes, and then maybe went down to 2 and then maybe she went down to like a 1.5. I don't know if she still does them. But when, last time I had seen her I was talking to her, she went down to 1.5 so she slowly let herself get off of it. A doctor told her, so I kind of just took it around with it.

I: We're getting near the end here. is there anything I haven't asked you that you would want to talk about?

P: No.

I: Let me just look through make sure I got everything. Okay, all right, um I really enjoyed talking with you today. It was really great to hear your perspective on so many different issues and your own experience with two different doctors was really interesting um. Any final thoughts at all before I turn off the recording?

P: Nope.
