# Supplementary material for: Experiences of postpartum mental health sequelae among black and biracial women during the COVID-19 pandemic
Source: BMC Pregnancy Childbirth. 2023 Sep 4;23:636. doi: 10.1186/s12884-023-05929-3 (PMC10478375; doi:10.1186/s12884-023-05929-3)
Supplement: Supplementary file 2 — Supplementary Material 2 [file 12884_2023_5929_MOESM2_ESM.docx]

**Supplemental File 1.20 Interview with Participant 5341**

I: So how’s your pregnancy going so far?

P: It’s fine actually. I have, I really didn't get really bad sickness yet like when I started taking the prenatals- I got sick for like two days but other than that was cool. I can't really eat that much, though. I don't have an appetite anymore.

I: How’s that been affecting you?

P: Like not eating?

I: yeah has it been affecting your energy levels and stuff?

P: yeah.

I: What have you been doing, have you found anything that helps bring your appetite back a bit- helps your energy?

P: I mean, I do smoke, but I don't smoke as much anymore. ‘Cause I haven’t smoked for like a couple of days, because I was very, very sick.

I: Tell me about that. (interrupts P) Sorry about that.

P: So, I got sick like the beginning, like the beginning when we first came into the New Year. And I really couldn’t eat nothing. I had- it was like my other mom made me some chicken and I can't eat that. I smelled the chicken and I just threw up. And the other days I can't really eat nothing at all like, even if it was soup I could eat the soup at all, either. I was getting like heat flashes and stuff like I wasn't feeling good at all.

I: I’m sorry to hear that.

P: yeah.

I: how's it been going more mentally I guess? How have you been feeling in that way?

P: It’s been good.

I: Alright, so jumping right into the meat of the interview, what do you think about marijuana use in general?

P: Well, I don’t think it’s bad or anything. I use it for like, you know, depression and stuff. And it just helps me not think about things (audio hiccup) but I don't think it's bad to smoke weed, it's just you shouldn't smoke it as often being pregnant. You should really calm down on that like it's not good for you. But other than that I don’t think nothing’s wrong with it.

I: What is depression like for you?

P: I get really, really sad…about [like when I’m sober and] stuff and I just get to think about things. I get really upset. But I know how to control myself better now than I used to control myself. But my depression is just horrible. And I'll just cry sometimes just by myself. I don't really like crying but it is what it is.

I: Can you tell me about how specifically marijuana helps that for you?

P: I don’t know- it just takes everything off my mind like it really takes a lot of things off my mind it doesn't… I don't have to think about the things that I think about when I smoke like after I smoke i'm fine. And then, like my appetite comes back to when I smoke. And yeah like I think- I think weed like really helps me a lot get through like depression. But I think I'm doing good with smoking. I haven't really smoked in like four days. Like I’m doing very good with that.

I: Is that mostly what you use it for your depression or there are other reasons?

P: yeah it's really for my depression.

I: And and you'd mentioned like, if you think you should use it even less during pregnancy and it's not really good for you tell me more about what you've heard about that what you think about that.

P: well… Like I know it can affect your child… in certain types of ways you may not know, but it could affect your child like my little cousin- my cousin she smoked or her whole pregnancy and he has like autism. Because of that, he acts very, very differently from other kids and you know I don't want my child to come out like that not saying is bad or anything but I don't want my child to you know, have to go through that because I made a decision on smoking door my whole pregnancy, because I do you know I do want to breastfeed and stuff and that- that does not like affect your breast milk, as well when you smoke or drink?

I: It can affect things for the reason we're doing this study is really to learn more about what happens with marijuana and and child development and stuff so that’s definitely part of what we're doing here.

P: Okay.

I: [how else have you heard that marijuana can affect your baby?]

P: I really didn't hear much about it, I just go off of like you know…My cousin- like a lot of people said, you know it's not bad to smoke during your pregnancy. But I don't want I don't want nothing wrong with my child so I'm not gonna smoke that much. And my sister she was telling me yesterday, like ‘oh you shouldn't smoke because it's going to affect your child and stuff.’ But like… Like when I smoke it just brings my appetite back like I need like at least like you know, two hits I'm not gonna smoke a whole you know blunt myself, like I can't do that I'll hit it like maybe three times, but then my appetite’s there so I can eat, so… I don't know why I can't really eat when I'm not high, but I can't eat that much like how only he probably once a day and I'll just nibble at my food like I will eat nothing at all, or it just won't go down like I can't eat like if I think about eat, I’ll throw up. And I don't know why I don't know why it happens.

I: Yeah, I don’t know. So you were mentioning some people who told you it's not bad to smoke marijuana during pregnancy, who are those people that have told you?

P: My cousin and this other girl that I know. she just had her baby recently she told me that… that's about it for my baby Father doesn't- hold on I’m on the phone. My baby’s father doesn't want me to smoke during my pregnancy at all and my mom doesn't [think that it’s good for me to smoke during my pregnancy either.]

I: Tell me more like what your parents told you about it… or your mom.

P: I don't know about my mom too much because I don't know her history, I know she used to smoke, but…I'm not sure of how much of a smoker she was, but she didn't smoke with none of us or do anything when she was pregnant with us, but she always tells me like ‘Oh, smoking is bad for you’. Like in general, even before I was pregnant she’ll tell me like smoking is bad for you, but as long as you know, it's not like no type of drug like you know…coke and all that, like I don't feel as if this it adds up to that stuff like I feel like that's a better coping mechanism than doing crack and you know all that other stuff. I feel like weed is better than any other type of drug.

I: What do you think makes weed better than other types of drugs?

P: I don't know it just like I mean it does depend, too, because you can get laced and you can die from certain people’s weed if they lace you, because they could put you know crack and stuff on it too like you never know that's why you have to watch how— weed that's not as— it's better but it's not you know, like weed can still kill you if you get laced from it if they put other types of drugs on it. That's why I watch what I smoke. That's why I don't really smoke that much anymore, because a lot of people i've heard like people have you know died from smoking weed because they got laced.

I: What, what can you do, I guess, or what do you do to avoid getting laced weed?

P: [I know the difference in weed. So I know if it's not good weed or if it’s good weed, or if it's laced. I never had laced before. I’ve never been laced but, like you can tell, like there's like there will be little crystals on it that you can see. That’s on the weed and you can see it perfectly fine, but some people wouldn't notice it if you don't know weed.]

I: You said earlier, like you, don't smoke that much during pregnancy, how do you know, or like, how do you decide how much is safe to smoke?

P: I really don't know how much I should smoke, but my doctor did sound like she told me, I should stop like I have, I have been stopping like I haven't smoked that much. I've been sober since I got and that was the first of January I got really, really sick. I think yesterday was my first time smoking, since the first.

I: Tell me more about talking with your doctor about this.

P: We didn't really make it a big, you know, topic like a big subject– she just told me like it isn’t good for you and you shall really smoke during your pregnancy, but it's like so who's been smoking for years and then it's like I had difficulty eating now due to not smoking. I mean I'd rather not smoke. But I still need like you know, like a hit or two. Just to eat, you know just to feel better because if not I’ll be really, really sick all day. Really sick.

I: did your doctor bring that topic up, I was not something you brought up to ask about?

P: No, it was me. I just told her like I do smoke just to let you know, like I did tell her that.

I: Did you have any worries going into that conversation?

P: No, I didn’t– not at all.

I: How did you I guess like how did you decide you wanted to tell her or why was it important to tell them?

P: We was- I forgot what me and her were talking about, but I think she asked me if I like smoked tobacco products or something. I told her ‘no like I don't smoke tobacco’ and I don’t smoke cigarettes, I told her like I did smoke weed though. And she's like well (you) shouldn't smoke weed, it is bad for you during your pregnancy. But we didn't get into depth about compensation or anything.

I: Did she tell you why it's bad?

P: No, we don't really have a big conversation.

I: Did you feel like any questions you had you were able to get answered, or do you still have lingering questions about that?

P: yeah I don't have any big questions on that for real. Oh, I don't yeah I don't really have any questions on I think you know she answered everything I had to talk about to her.

I: Okay. So I'm gonna jump back a little bit and ask about your thoughts about tobacco in general.

P: Well, I don’t think tobacco is good, honestly. Tobacco like it could just give you so many problems and so many different ways, like one disease and stuff like you never know like- tobacco is (disgusted?? sound) I'm not gonna lie like I used to smoke like blacks and stopped like a long time ago. That was like… two years ago. I was smoking- the last time I really smoked a black was when my brother got shot in September, but other than that, like I won't I won't essentially smoke it if I don't have nothing to smoke I won't go act like ‘Let me go buy a black. Can you give me this?’ You know I wouldn't do that. Like tobacco is not good for you and blacks– they're worse than cigarettes and cigarettes are still bad for you. I think someone in my family like their lungs just became really bad from smoking like you know, tobacco and stuff. That’s why I won't smoke it.

I: What do you think about tobacco use during pregnancy?

P: I don't know how it would affect the child that much, but I don’t feel like you should really smoke tobacco during your pregnancy like I don't think that's good for real- like shouldn’t really do it. I don't think it's healthy for your child honestly. Like any type of smoking is really not healthy for your child smoking, drinking none of that's healthy for your child. Because in the future, you never know your child to have brain difficulties or something wrong with their breathing and stuff. You know, some people aren’t as lucky, as some people that smoke and some people that do smoke like- Their babies go through some… traumatizing things and some people, you know they just got lucky for their child like their child didn’t have to go through you know, certain things that children had to go through.

I: Do you think between marijuana and tobacco, one of them is safer?

P: I mean, I don't think neither safer, but like I said I would prefer you know, weed over tobacco anyday.

I: Okay. What makes you prefer weed over tobacco, if you think like they're both equally safe- just the way they make you feel?

P: [The nicotine in the tobacco has a stronger hit], than weed does like– has a way stronger hit like I don't like the way tobacco hits, it's really strong. And I will never like, you know, smoke it ever again in life.

I: What have other people told you about marijuana and tobacco either during pregnancy or just in general?

P: Well… I really– my mom– she only told me like you know weed isn’t good for you, but tobacco a lot of people say- like tobacco you shouldn't smoke tobacco cuz it's really, really bad for you, for you and your lungs, but that's the same as like you know, smoking weed is bad for your lungs as well because it’s same thing. And if you smoke blunts that's made out of tobacco— woods that's made out of tobacco as well. But I really never had a big conversation on you know, how it could affect [yourself]. I just know that it could give you a lung disease, but I don’t want to know too much about smoking tobacco.

I: Where would you get information about marijuana and tobacco, if you had questions?

P: Like if anything I'll ask my doctor about it. Doesn't really have a source. I’ll just ask or I'll look it up on the Internet, you know, figure out some things from the internet. That's the only thing I can do– go to the Internet or go to my doctor.

I: And how do you determine the trustworthiness of things you see on the Internet, because obviously there's some conflicting information out there?

P: yeah it depends, sometimes I really don't trust the internet as much because you know the internet does lie to you about a lot of things. I wouldn't choose the Internet, I’d rather a person tell me than the Internet because the Internet can just lie about the stuff you're trying to figure out. Because like you know I did have covid like while I was pregnant. The doctor told me, I can have a miscarriage you know during having covid like i'm not too sure, but I looked it up on the Internet, I didn't really get too much information about it so just wanted to talk to my doctor about it. But she doesn't have a regular cell where I can just call her and talk to her. But I'm not sure if I still have covid but I've been [you know from the 1st, that’s when I got really sick. I had covid but] now i'm totally better- like I don't… I'm not in the bath throwing up or that I can't move, I can't eat nothing. I still can't eat as much like- I still won't eat like that was before I even got covid I didn’t eat that much. I can tell I lost weight from not eating though. I can tell that because when I went to the doctors last time I was like 140 something now my 133 maybe. If I’m not eating… and I know that’s not good for me but it's just hard sometimes.

I: You were saying you wanted to ask your doctor which [isn’t easy since they don’t have a phone you could call,] what is the process for getting like a question answered from your doctor if one comes up between appointments? Are you able to do that?

P: Say that again.

I: Like you were mentioning, you wanted to ask your doctor about covid and stuff like that, but she didn't exactly have a cell phone you could just call her on. What is the process or what do you do if you have a question that comes up between appointments? Are you able to get in contact with her?

P: I really never call her. But I have an appointment coming up soon… like Monday. By then I should be fine, you know I shouldn't be sick no more like I'm not sick anymore. But like I do still have congestion, but that's all, that's about it, but I do want to actually like how it could affect the baby, you know in different ways, like cuz like I don't really know, like the doctor told me like it is possible when I went to the er the doctor was like it is possible for you to have a miscarriage. Because you have covid but it's more of a reverse for you, you know I didn't get the shot or anything so you know it's more of a risk for me to be hospitalized than it is for someone who got their shots.

I: [Besides having covid yourself, how else has covid affected your pregnancy] just being in the pandemic?

P: [It hasn’t affected me too crazily. My pregnancy, I would have sharp [pains] in my stomach. And it was only like maybe a day or two I had really bad sharp pain. But I have cramps. It isn't that normal for like when you like– is cramps supposed to stop when you're pregnant, because I had them for a while?

I: I'm not sure I think you'd have to ask the doctor.

P: Yes, I looked that up on the Internet as well, they said five - six weeks like cramps are supposed to stay but I just had cramps like from like maybe a week after. Like I didn't find out I was pregnant so like December, the beginning of December. I was having cramps the week after I found out or, not even when I found out and I'm still having cramps I was thinking you know my period will come. But it wasn't. I was actually pregnant, because I didn't believe I was pregnant when I found out. I had to wait til I got to the doctors for real to get that confirmation that I was pregnant and I was still getting cramps even after I went to the doctor, I was getting cramps like I think my cramps stopped maybe the last day of December. But after that, like I did I had sharp pain, other than that… Nothing too crazy.

I: How has living in the pandemic impacted your marijuana use, do you think that's had any effect?

P: It made me smoke a lot more than what I did before, because there wasn't nothing really to do, you know? When you have to just sit in your house when the pandemic first happened, it was horrible like all I did was sit in the house is smoke, go to sleep, smoke again… smoke. I did my school and stuff- like I was cool with school. I did my classes and stuff, so it wasn’t too crazy.

I smoked a lot more, like 10 times more than I used to smoke. I can say that, like before the pandemic I didn't smoke as much, but once the pandemic hit I smoked like 10 times more than what I smoked before the pandemic. But Since i've been pregnant like I’ve chilled out a lot with smoking, I won't smoke. Like my tolerance for weed now is very low, like I used to have a very high tolerance, where I could smoke, maybe like… 3-5 blunts a day- probably even more at that. Which now I can't really even smoke a whole you know blunt, so I can't do that– I won't be able to smoke no blunt by myself, I guess, I could probably hear like five times and then I'm done, I can't smoke it like I used to.

I: Is three to five how much you would usually smoke like during the beginning of the pandemic or before the pandemic like when was that time period where you had that tolerance?

P: I had that throughout like…. during the pandemic. It was like when I was smoking that much because I wasn't going to school, you know I didn't have somewhere to be at a certain time or none of that I just had to be in a house or I'll just go over my friend's house and we'll just smoke like. I will say that I spent a lot of money, a lot of money on smoking. It was a lot of money, like, I will smoke like $20 worth of weed that'd be done in like 10 minutes. I'll go buy another $20 worth of weed. Finished that and then you know roll up again just keep on buying so like maybe I’m spending 100 every day, you know i'm saying like that's a lot of money, just to be you know spending on weed like hundred dollars every day that's a lot of money.

I: Yes.

P: And then at that you have to buy you know the blunts and stuff like that money as well. I think I've really chilled out a lot with smoking.

I: Some more about how that affected you financially over the long term. Did things change with your use once you realize how much you're spending or did that not really matter to you?

P: yeah it really didn’t matter as much to me because, like I did like…Once we were able to like you know work again and stuff you know, I worked, I was able to… I worked at a summer job. I got paid really good, and when I got a full time job, I was getting paid good as well, so it really didn't affect me that much. But then, you know, once you get older and you realize all that money you did spend that money could have gone to other things like I do think about that now like ‘dang all that money I was spending on weed.’ I could have really been rich, I can be rich. I could’ve had all the money in the world if I didn't spend it on just smoking weed.

I: So tell me more about when you first tried using marijuana.

P: [When I first started it was a totally different experience.] When I first hit the blunt… It was just like I was on a roller coaster. I was spinning around. It wasn't like you know, I was, I felt sick or anything, it was just like a good feeling and then ever since then, when I first hit that but I just couldn't stop. I just continually smoked and smoked. When I first started smoking I wasn't that big of a smoker. I didn’t care too much. I would get sick if I smoked too many of them. If I was to smoke two Ls like I was, I would go up like I can handle that my tolerance wasn't that high yet.

I: What led you like, why did you try it at that time?

P: I don't even remember it was just me and my sister and my sister she smoked. You know she's older than me so she was already smoking. And I don't know- I don't remember– why I even smoked. I don’t remember any of that- I don’t remember why but I just remember I smoked with my sister. I like the feeling and I just have been smoking since.

I: How long ago was that?

P: When I was 13.

I: So you've cut down a lot since you became pregnant, have you ever tried to cut down before?

P: No, I haven’t. I would have tried, but it didn't work. [The people I surround myself with] they smoke a lot so just like when people smoke and they’re around you- you want to smoke as well, like you want to hit that L.

I: So what has it been like for you to cut down?

P: It hasn't really affected me too crazy. I thought it was like you know, affecting me really badly, but not smoking for a couple of days, [it was like wow, I can really not smoke and be okay.] Because like when I didn't smoke, like, I would just like… I know when I need to smoke and when I don't smoke. When I start to get sad out of nowhere I just be like yeah I need a smoke real quick because, like I don't like to be depressed or anything, be sad about anything. And you know that was just my coping mechanism like ‘oh yeah, let me just go–’ even now like, I still think that way, but I won’t just go and buy weed all the time.

I: What have you been doing to cope instead?

P: Honestly, I have just been sleeping. I don’t really do anything– like I’ve been doing online school, but I don’t have to go into school. So I do my school online, and I do all my schoolwork. I'm catching up now at school, so I really haven't focused on smoking or anything. that’s not a big worry because (yawn + audio failure) in my future, I have a child, I have a child to worry about now so smoking is not like that big because a child is more than smoking weed you know it's more expensive than just smoke weed in it sorry weed all the time that's expensive as well, like… So it just made me think like I shouldn't smoke that much. I shouldn’t really smoke that often.

I: Since you said, like a lot of people you surround yourself with do use marijuana has cutting down for you like affected any of your relationships with those people?

P: Not really. I don’t be around him as much as I used to, you know, when covid first hit. It was just like everybody was like nobody smoke that much once covid hit that's when everybody started smoking all crazy. Like ever since you know, we were able to go outside and stuff like… I don’t be around them as much as I used to. I used to be with them every single day. Now I don't really see them anymore, even though they live up the street from me and I only talk to people anymore as distance myself.

I: So you've chosen to distance yourself or just happened?

P: yeah I chose, I chose to.

I: Tell me more about that.

P: Well, like some people- I don't know– some people just be you know different like once once you see someone's true colors and how to really be acting. You just don't want to be around like you don't want to be around negativity or none of that. Like I don't like to surround myself with negative energy. Because negative energy just isn't it for me I don't really mix well with negative energy like I don't like it rubbing off on me. I rather be around myself than be around people who just throw off negative energy or who just acts fake when you know…They want to come around or they talk to you when they need something or you know.

I: Yeah, did you feel pressured by them to keep using marijuana?

P: No, not at all.

I: I'm also curious whether you plan to quit at some point during your pregnancy or do you think using like at a pretty low amount is what you want to keep doing?

P: I do plan on stopping.

I: You do?

P: Yeah.

I: Do you have a timeline plan tonight I know some people do, or is it just like when it feels right?

P: I don’t really know for real. I just know I want to stop. Like… My baby's father is cool with it right now, but he’s not cool with me smoking like he does not approve of me smoking whatsoever like he does not care for me to smoke at all, but he says you know, I’m still at the beginning. Not too many weeks or anything. Like I can smoke, but I can't smoke that much. He'll tell me once you get to a certain amount of weeks like you're not gonna be smoking anymore. He told me that when we first found out I was pregnant.

I: And then I can ask the same questions about tobacco like when did you first start using smoking blacks like you said?

P: Maybe when I was like 13-14.

I: why did you start that?

P: It just gave me a little buzz or whatever, but that was just it.

I: And, did you normally use that, for the same reasons as marijuana more social? What was the difference in using them?

P: Well, like a black it just gave me like a tiny little buzz, but weed is just like– it will get you high. Blacks– it depends on the blacks as well. I used to be able to smoke a whole black just for myself, but it was just for a buzz but… Now that I’m looking back on it now that I’m older, I wouldn’t go back to smoking blacks, that’s just not cute. It’s not even cool either because it’s just nasty. The taste of it is nasty– that’s why I don’t really smoke blacks and stuff.

I: Did you ever use marijuana and smoke blacks at the same time, on the same day?

P: yeah.

I: Was that like versus using them separately?

P: I don't know, like it didn't really affect me nothing too crazy with blunts- like smoking them both on the same day, like– It didn't really do too much. It's just like you know, smoke weed and smoke a black, and it just gives you a lot of you know, higher feeling, but I wouldn’t really imagine it affecting me nothing too crazy.

I: So I think pretty much just have some more questions about things like your experience with your doctor left, so I'm curious if anything like– what have others told you about talking to your doctor about marijuana or tobacco during pregnancy?

P: Nobody told me anything about speaking to my doctor about it. It was just me, you know out i'll just you know have questions for her about you know smoking and stuff that's about it, though.

I: I think we may have hinted on this a little bit earlier, but I just want to ask again like what influenced you to talk to your doctor about this, why did you decide to do that?

P: We were talking about tobacco… And I just wanted her to, you know, to be aware that I do smoke. I wanted her to be aware of that. She just told me like it's not good for you, you just shouldn't smoke. And then, after that she had to go deal with what she had to deal with because that day, like the machines and stuff we're down– couldn’t really do too much, we had to wait until the computer went back up for us to get everything in the system. So yeah we just had a tiny position and nothing too big about it.

I: So what makes it important to you that she's aware like what benefit does not provide? Why didn't you want to make sure she was aware?

P: I don't really know why I wanted to make her aware, I just wanted to let her know like– Like if there was like I don't know if it could affect me or something I just wanted her to, you know, like if something's wrong like it's because I smoke, you know.

I: So besides her, briefly, talking to you did you get any other information like pamphlets or anything like that, at your appointment about substance use during pregnancy?

P: No, I get no pamphlets or anything, just a book about pregnancy. It doesn't talk about smoking or anything. It just talks about like you know when I go to the doctors for certain things and there's like videos and stuff. I’ll look on my phone to find the book there's like a scanning thing. But I haven't talked– they haven't talked about you know… tobacco yet or weed.

I: What do you think would help young pregnant women like yourself get more information about these kinds of topics that we're discussing?

P: I don’t really know. The only person I can really say is your doctor or like you know if you do like a drug and alcohol program you can always get answers from them, but I don’t do no drugs and alcohol program. I don't have to (unintelligible).

I: I was just gonna say like– what do you think doctors can or should do to help women feel more comfortable and to get more information to them?

P: I think it just depends on like you know your relationship with your doctor, because some people don’t feel comfortable talking to their doctor about things like that. See, I’m telling my doctor things like that. So I’ve had her since, you know, I was 14. since then it's like i'm comfortable with talking to her about smoking and stuff. But some people may not feel comfortable talking to their doctor about smoking.

I: What can doctors do to build that relationship and help them feel comfortable, especially if they don't have a prior relationship with that person, and they’re a newer patient? What can they do?

P: I just feel like they should just, you know, give them respect and build trust. they just you know build a little bond, because when I first like you know… talked to my doctor I was really, really scared. I didn't want her to check down there, none of that. I was just really, really shy. I didn’t want none of that done. Now that I know her more like I'm very cool with her– just you know checking me, but when I first– I didn't– I didn't trust her. I didn't like her, doing that, like. Didn't like talking to her, addressing things that I needed to talk to her about. I’ve had her, you know, I’ve had her– I would go to my cousin and ask her about things before I would have asked my doctor, so I feel like if you have a family member that knows about you know certain things you could talk to them. If you don't feel comfortable with your doctor, like your parents or someone who had that relationship with your mom or your dad. You could talk to them before you talk to your doctor and they could give you advice on things, but other than that I don’t really know.

I: Is there anything that you noticed your doctor did that really helped you start to feel more comfortable opening up to her?

P: Yeah, she– She talks a lot and we spoke about a lot of things and stuff. I just feel like we were building a better bond than what we had like we had like a better trust. Well I trusted her like better than what I did before, like because we did speak we speak about like certain things about sex and stuff because I don't really feel comfortable talking about that with other people. But my doctor just I don't know– they just have to show you that you can trust them and I feel like she showed me that she can trust me, I mean I could trust her with information that maybe I didn't want to tell my mom. she won’t go back and tell my mom about things, or you know family members about things or go to anybody, you know talk about somebody's personal life like I was like she showed me that.

I: Besides asking their doctor and family members, what else do you think would help young pregnant women information on these kinds of things? Could be something that doesn't even exist, like what would you kind of dream up if you could help everyone get their questions answered?

P: I don’t know, the only thing I can say is like you know, the Internet, but the Internet doesn't always give you (audio malfunction) You know unless you’re at the doctor or hospital something and they have pamphlets and they can just grab one of the books. Just grab one of those and read about it. I don't know.

I: What makes talking to me as a researcher different from talking to your doctor about things like marijuana and tobacco? Is it really different to you?

P: I don’t really think so that much. I’ve never really had a big talk with my doctor about, you know, smoking like before pregnancy… but I don’t think it's a big difference. I don’t think it’s too crazy of a difference.

I: Do you feel more comfortable sharing with a researcher or with your doctor like are there different kinds of things you would discuss… anything like that?

P: Not for real.

I: last question here, so, in some circumstances marijuana has been made legal, for instance here like medical marijuana became available in 2018. What do you think about that?

P: Like medical?

I: Yeah, what do you think about medical marijuana or marijuana becoming legalized?

P: I think like your doctor prescribing like marijuana is better than you just getting it off those you know just somebody off the street, because it's prescribed to you, and they like they had their own specialist check out the leaf for you. It's not like oh you buy it off somebody else in the streets you don't know what’s in that weed. The doctor, they know. They have a specialist to tell them what's going on, and if it's a good weed for them, or no, you shouldn't give this to this patient, you know I feel like they should have different prescriptions for different people like when it comes the weed because every weed isn’t the same like there's different weeds. I feel like prescribed weed is way better to just get it off someone from the streets honestly.

I: Well that was pretty much the last question from me, is there anything that I didn't ask that you would want to add or that you think would be helpful?

P: No.

I: Well, I am going to go ahead and turn off the recording here.
