# Supplementary material for: Experiences of postpartum mental health sequelae among black and biracial women during the COVID-19 pandemic
Source: BMC Pregnancy Childbirth. 2023 Sep 4;23:636. doi: 10.1186/s12884-023-05929-3 (PMC10478375; doi:10.1186/s12884-023-05929-3)
Supplement: Supplementary file 3 — Supplementary Material 3 [file 12884_2023_5929_MOESM3_ESM.docx]

**Supplemental File 1.24 Interview Transcript with Participant 5301**

I: Okay, first I want to thank you for agreeing to this, we really appreciate your input and I’m going to use the term LGBTQ+ throughout the interview. So that just means anyone that's not 100% heterosexual. Is it Okay if I refer to you as bisexual throughout the interview? How do you want me to identify you?

P: Bisexual is fine.

I: Okay, so I'll try to use that. And that's pretty much the intro. So, first, I just want to ask a little bit about your pregnancy, how do you feel like it's been going so far, how do you feel?

P: I feel fine. I do have a lot of morning sickness.

I: You're out of the first trimester though, right?

P: Yes.

I: What have you liked about being pregnant so far?

P: Watching my bellow grow. But I don’t like the pain.

I: Are you experiencing pain?

P: Yea, lots of pain.

I: Yeah? Is there more pain, besides just the morning sickness that is 24-7?

P: Yea, back pain, general pain.

I: How has your body changed; how do you feel about those changes?

P: I actually didn’t gain weight at all. If anything, I probably lost weight. This baby is making me sick all day.

I: It's probably hard to have an appetite when you're literally nauseous all day every day.

P: yeah, it's literally like I don't eat breakfast, I eat maybe a little bit of lunch.

I: How do you feel like your health care, your doctor, have been about you being sick?

P: they've been very helpful; they prescribe medicine that I ask for. They do everything that I've been asking them to do. So that's one thing I'm really happy about. Because I’ve been pregnant before and I know exactly what works.

I: Okay, do you have the same doctors you had in your first pregnancy?

P: Yes.

I: Great. For us, this makes a good transition into asking you the next section of the interview. It is going to be all about your health care experiences, good and bad, and that includes doctors, nurses, even like the clinic environment physically, how you feel being there. So, you said that you feel your doctor listens to you about what you need?

P: yeah, my doctor is very helpful, gives me everything I ask for and they prescribe it.

I: that's great, I'm glad that they are listening to you because you are the expert. Have you had any negative experiences so far with your health care?

P: No.

I: Okay! Now, I want to ask a little bit about your sexual orientation and health care. So, did any of your doctors, providers, during your pregnancy, mentioned anything about you identifying as bisexual, or even just ask about it?

P: No.

I: How would you feel if they did? Is that something you want them to talk about with you? What are your thoughts about that?

P: they never really asked me. But if they did, I would tell them I am sexually attracted to girls but I’ve never been with one before. So, it’s not really much of an information I can give them.

I: Do you think it's important for them to ask about that stuff? Or you don't think it really matters. What do you think? Is it a part of your care, or it's not relevant?

P: Not relevant really.

I: in general, is the healthcare environment friendly towards pregnant LGBTQ+ people?

P: for the most part. I have seen a couple of situations where they have been uncomfortable in the hospital because of their sexuality.

I: Yea? can you tell me about those.

P: This lady was pregnant, and her physical appearance looked like a man. When the doctor came in, they looked at the female appearing one who is just the partner who is not pregnant, but the doctor assumed she was the one who was pregnant. And the doctor later got confused, left room and said they needed to check something. They later came back and was like “what is your name again?” and “I’m sorry, I just don’t recognize people if they look different from last time I saw them.”

I: yeah, I feel like that wouldn't feel good to that person at all. I wonder what the differences between sexuality and like gender you know.

P: I think it's usually worse for a man transitioning into a woman, and less bad for a woman into a man. Because it's like, people can see there's a physical aspect. Also, you have to ask about sexuality often.

I: And, in general, what resources do you feel like have been helpful to you about for your pregnancy?

P: Obviously, the YoungMom study course. Besides that, there really isn’t anything else.

I: Are there resources that you wish you had access to, that don't exist right now?

P: No.

I: If your doctor was going to ask about your sexuality, how would you like them to do that?

P: Just ask me straight up if I have a sexual orientation or something like that.

I: OK. Is there anything else, before we move to the next part, that you want to share, about your sexual orientation and being pregnant?

P: No.

I: Okay, so the next part is going to be about marijuana use, so I like to remind people during this section that we never report this information to anyone… it's totally confidential. None of your family doctors will hear this information. So, before we start the marijuana questions, do you have anything you want to ask me?

P: No.

I: ok. So, can you tell me a little bit about the first time that you tried marijuana?

P: I was around the age of 14. And I was with my friend, mostly under peer pressure.

I: Can you tell me more about that?

P: I was with my friend. She just had a baby, so it was me, her, and her sister. I had never done it before. They ended up texting their friend and was like, “hey, bring it to me.” My friend’s brother later came, he was my then boyfriend. We all went to the bathroom, and it was me him, his cousin, and my friend. I was in bathtub because I was feeling uncomfortable, wanted to stand away from them. They said, “ok, well, how about you try it?” and “Ok, now take another one,” because each person hit 3. I hit one and started choking to death-- I couldn’t breathe! Everyone started laughing! I told them that I really couldn’t breathe, and they were like, “calm down and drink some water.” His sister was laughing. We then all ran out of the bathroom, sitting in the hallway, looking across at each other, everyone was just laughing. I was like “I don’t understand what’s so funny,” which also ended up making me laugh, and I could not stop. Then my grandma tried calling me on the phone. I started to freak out, thinking, “what if she finds out that I’m high?” I could not stop laughing. Grandma asked me over the phone, “what’s so funny?” I said, “nothing!” And just couldn’t stop laughing. But I was definitely freaking out because I don’t know what to do. My grandma later came to pick me up. Initially. we got in the car, my grandma was like “you smell like marijuana,” I had to pretend, so I was like “oh, I do? That’s probably because I was around people who were smoking.” Grandma asked me why I was with people who were smoking. She eventually caught on, but I was really trying hard to cover up at the time because I didn’t know how to tell her. She’s a church lady, such a drama queen. She sat me down to talk about marijuana and was like, “you don’t do it… right?” and I was like “yes, I don’t do it!”

I: This story that was great-- I don't think I've laughed ever so hard in an interview… yeah, your church lady grandma knew what was going on though!

P: I was just stuttering and laughing and she’s like “what’s wrong with you?!” and I was like “I don’t know-- I got allergy!” But my grandma knows I don’t have allergies. And grandma later tells me, “so, your grandpa and I had a talk; we think you’re high. Just please don’t hang around with the wrong people.” My grandma thinks that marijuana's a gateway drug, that if you try marijuana, you're going to want to try crap, and even if you think you can just do it once, you’re gonna want to try it again.

I: Do you agree with that, like what do you think about people that use marijuana?

P: Honestly, don't think marijuana is a gateway drug at all, because even after the experience I had, at first, I did it because I wanted to get high with my friends. I quit because I was pregnant. After I had baby, I feel that I wish I could use it to get away from reality, but it ends up just being relaxing for me, and it did not really help me get away from reality.

I: Okay, so you stopped smoking marijuana after the first pregnancy. But then, when you got pregnant, this was with your second pregnancy, you quit during that-- is that right?

P: Yes.

I: And then, when you had postpartum depression, you kind of picked up smoking a little bit then.

P: Yea, and I had a type of eating disorder where I got a tube in my body, to help my stomach or something like that. But basically, I just stopped taking edibles after a while, and I don’t smoke.

I: what's the difference between the two for you? How do they feel differently for you?

P: Edibles, using it I know it’s about to kick in in 1 hour or so, and because I have control over when to get high, I plan well so that it doesn’t hit me when on bus or in school. Also, edibles… it’s a less intense high. If you're smoking, you’d expect it to hit you harder, because you take it all in at once; it’s just sitting in your brain and you feel dead for like a couple of seconds. So, there's less of that with edibles and more just like the calmness.

I: Was it hard for you to quit when you realized you were pregnant?

P: With my first pregnancy, it wasn't that hard at all. I quit whenever I want to.

I: What about this pregnancy? Was it hard to quit?

P: Not really. I found out I was pregnant really early on. God gave me a chance-- now I gotta stop doing what I’m doing… because I’ve got a baby on the way! I stopped smoking. I didn’t want my baby to be hurt. I was not addicted to it. I can quit whenever I want to. I was only smoking because of the other people around me. They were smoking because they feel depressed or want to be happy. But for me it was just a way for me to get away.

I: Thank you. How do you think that we, healthcare research, can like help support pregnant women that maybe want to quit or cut down on weed during pregnancy?

P: I don’t know. I guess, I would like to see more information on how to go cold Turkey.

I: Okay, well, now that you are not smoking weed, what do you do to deal with your stress? What do you do to cope?

P: Video games. And then I try to keep myself away from my partner-- he does smoke, so he will go out and do what he does, and try to keep it away from me.

I: Ok, this is the last question about marijuana. So sometimes people report that being bisexual influences their substance use, maybe because of discrimination, we don't really know. But do you think that your sexuality has an effect on your substance use with marijuana?

P: Of course not, I don’t even think the two are related.

I: Tell me more.

P: Marijuana, I would describe it as a drug that people take to feels something. I don’t know what they feel because it’s different for everyone. Some feel bad, some feel good. I don't know what they feel because it's different for everybody, OK. And, yes, marijuana influences your brain to feel a certain way, but it’s not going to make you go like, “oh yea, I like girls now,” I just don’t believe that. And “now I’m bisexual because of marijuana,” I don’t believe that. I think you’re lying.

I: What about the reverse of that? Do you think being bisexual affects how you use substances?

P: I would think it would be the same thing. They are separate. I don't believe that if you’re bisexual you smoke more or smoke less, what you did with marijuana or anything like that-- unless if you’re peer pressured, because if somebody you associated with, you hang out with, is bisexual and is smoking marijuana, then I mean, of course, you might be like “okay yeah, let’s do marijuana,” but that would be a personal experience in my opinion.

I: Thank you for explaining that, I know it's kind of abstract and hard to figure out, but I understand what you're saying totally. Okay, so the next part is all the same questions, but we're going to talk about tobacco use and tobacco use during pregnancy. So, have you smoked cigarettes or vaped, cigar, anything like that?

P: I have vaped before. But no cigarettes.

I: What was vaping like for the first time?

P: It was around maybe three years back, I was with my best friend. It was when vaping was becoming popular, so she would vape when we were out at the Juul bar. I've always watched her do it, I'm like “guys… it smells like death, I don't want to do that,” and my friend would be like “No. Just try it.” And I would say, “No, I just want to stick to marijuana.” And eventually, we were leaving and we were in a car. While in the car, she keeps hitting it in my face. I took her pen off of her. She taunted me, “You gonna do it? I dare you. You not.” I was like, “I’m not scared.” So I took a hit from her pen, hit it for the first time on video. It was way, way different than smoking marijuana for me. It didn’t hit the back of the throat like marijuana. It made me choke and cough, because the smoke hit my nose. It’s just different. I did not know what to expect but did not like it. I’ve never tried a Juul ever again. It gives you that lightheadedness like marijuana, but only a sec, then you cough, then the lightheadedness goes away. Big difference from marijuana.

I: So you basically vaped like once or twice, and hat was enough for you to be like, “you know what this isn't for me.”

P: Yes. I prefer marijuana. With vaping, I get sick after I smoke, especially on an empty stomach, if you hit it a couple times. Yea, no, I’m done. My stomach would start churning, my lungs would start hurting. It does smell good and taste good but only for a little second. I would not recommend. In the commercials you see people going to the hospital-- I don’t want to risk it.

I: Right, what are your thoughts about it, like for people that are smoking during pregnancy?

P: I would think, “dang, what is wrong with them?” because it can hurt your baby and your stomach like that it makes you feel nauseous. And not just that, people are going to die with some problems like blood clot and stuff like that if you smoke. I feel like it’s terrible because it can affect the baby, too.

I: Okay, the next question is, they're called ideal world questions, so I want you to imagine being pregnant in your idea of a perfect world. In this world, what do you wish all LGBTQ+ women knew about pregnancy?

P: That it is hard to do it on your own. If you’re LGBTQ+ and you want to be pregnant, you have to realize that you do have to have a partner. If your partner is the same sex as you, but they didn’t contribute to making the baby, they are not obligated to stay with you and your baby. I have a friend. She and her partner chose to have a baby together. If the partner-- you’re not paying the 8000 dollar to have your DNA given to making the baby, so you can technically walk away. So, a bunch of legal stuff. If the people aren't doing this through a medical procedure, the partner is not really responsible for that pregnancy and that baby. Because the other person could walk away whenever they feel like. That’s what happened to my best friend-- she called me, she said “hey, my girlfriend and I are having a baby. We want a baby!” I’m like, “who’s going to carry?” She said, “Me!” I’m like, “What about your girlfriend, how she does feel? She must be 100% down with it.” Months past, my best friend called again and was like, “she left me.” Why? “We had an argument and I told her she has to stay because of our baby. And my girlfriend told me that the baby is not hers, so she doesn’t have to take care of it.” Well, I think the girlfriend is right, technically she is not and she did not provide her DNA. Like I said before, the girlfriend is not part of the medical procedure to make the baby, so she really isn’t responsible for that.

I: That is such an interesting response, no one has said that before and I haven't thought about it, but it's a great thing to bring up and it’s an obvious thing that could cause problems and potentially be a hard thing to solve.

P: If you're raising a child, on your own, or if you're raising a little one, you have to be prepared to set a good example for that child—LGBTQ+ aside, being bisexual aside – like my friend, she'll want to imprint that to her baby’s head the second they are borne, but then they’ll wonder why their child is different from other child… that is because you have imprinted early on when they are young and now it’s what they are used to.

I: Yea, that's another good thing to bring up. Not something I had thought about before. What do you think, in the perfect world of healthcare, what should providers specifically know about pregnancy or in LGBTQ plus women?

P: Be mindful about the fact that there are differences to talk about.

I: Now, what do you wish all LGBT Q plus women knew about tobacco use?

P: I mean, they're just like any other human. But they just have different sexual orientation. That’s all. Like, if you smoke, your teeth fall out – like this happens to everybody, not just LGBT kids, okay.

I: Yeah, I like that spin-- they're just people and everyone should have access to the information to make the choices. Well, that is it for me, except for that I wanted to know, maybe what I could have done better, or if you think that we need to ask something about this stuff that we're not asking. I want your opinion about that, and then the interview is pretty much done.

P: Job great job there's nothing that I will ask you to ask me more.

I: So we pretty much covered all of the topics. I can only do as good of a job, as the person I’m interviewing. And I like your stories, I was so into it. You can't see me but I’m nodding along. It was so great, I really can't thank you enough for being willing to share your experience with us, that is so valuable.

P: it's something that I wish I could do more often. I never told the story of my first-time smoking and stuff.

I: It was funny and it almost reminds me of like a comedy skit, like that was the level of funny; I could see every single point in that story happening, like in real time, I felt.

P: it's funny because I don't even get along with the girl that I smoked with and that’s just crazy and funny to think that. It is so hilarious how you see people react to a drug they’ve never done before, yes, yes, yes and it's funny to remember how different generations of your family reacted to it.

I: And the part when you try to cover it up, like, it's just so good, I can picture, like a 14 yo doing that, you know. And yea, different generations react differently.

P: I honestly feel like its not a big deal anymore in this world to use marijuana. Like we use it as an antidepressant, use it to figure out the world, it’s really not a gate way drug, just a fun drug.

I: Sounds like there’s benefit to it.

P: Yes. It’s something you can prescribe. I know it’s not the same for everybody but for me it’s not addicting, because I quit like it’s nothing. My grandma is always like, “be careful… know who you are getting it off of… Don’t do it because I have a nice house and I don’t want you be living here doing that.”

I: Do you ever get cravings?

P: Only when I actively think about it. Like if I’m at work, sometimes I think about and I’m like, “man, I wish I could have some right now.” A little craving. But if you have a little bit of discipline to tell yourself you don’t need it, you’re ok.”

I: If you have enough self-discipline and self-control, sounds like.

P: Yes, there's a big difference. Because there's people in the world who believe they don’t have what it takes to quit… Because they always ask me like, “how did you quit when you got pregnant?” I just quit because I don't need it. Your body doesn't need marijuana… marijuana just closes your body down, you're just walking around eating stuff, like a zombie, you smoke, you lay down in bed, go to sleep, go to eat. But I feel as if… if people understood that the body doesn't need marijuana, and that they just crave it because that’s something they are used to. They feel like they're addicted to it. That's why I feel like alcohol is different.

I: Yeah, I mean there are reasons why marijuana is illegal versus alcohol aren’t. I would love if we asked about that, because I think there's a lot of stuff there. Personally, I would find that interesting.

P: Personally I feel alcohol should be illegal. It can be an antidepressant but it can be harmful. Black out. With marijuana I’ve never heard of people blacking out or killing or hurting others. But with alcohol, if you have too much in one night—Poisoning, you could be dead. For me personally, all the times we try alcohol, it was very, very, very bad. Blacking out, waking up in the morning with ravioli all over me.

I: Like in a lot of ways, alcohol does have more immediate serious consequences.

P: Yea, and that’s why I mean when I say we have to fight for marijuana to become legal but alcohol and other bad stuff has been around for centuries. That just doesn't make sense, because there's so many people that, like my mom, she's drunk all the time. A lot of terrible stuff can happen. On the other hand, with my first experience with marijuana, I just couldn't stop laughing, and a lot of it had to do with dealing with my grand. In terms of consequences, that's not that serious compared to what some people deal with with alcohol.

I: Okay, I'm going to put $50 on that card, and then we haven't started it yet, but hopefully within the next month, we're going to be doing another type of study about racism and Covid 19. And that one pays $100 per interview, so if that's something you're interested in I can reach out to you, and in a month.

P: Okay, and that will be great.

I: Okay. Thank you so much, I hope you have a good rest of your day.
