# Supplementary material for: Experiences of postpartum mental health sequelae among black and biracial women during the COVID-19 pandemic
Source: BMC Pregnancy Childbirth. 2023 Sep 4;23:636. doi: 10.1186/s12884-023-05929-3 (PMC10478375; doi:10.1186/s12884-023-05929-3)
Supplement: Supplementary file 4 — Supplementary Material 4 [file 12884_2023_5929_MOESM4_ESM.docx]

**Supplemental File 1.25 Interview Transcript with Participant 5276**

I: We’re ready. So, how's your pregnancy going so far?

P: It's okay. My stomach be hurting a little bit.

I: Mm. Your stomach’s hurting?

P: Yeah.

I: Have you found anything that’s been able to help with that?

P: Like, they said I got a low iron cause I’ve been having like, um, I’ve been going to the ER and I’ve been having like a little bit of bleeding, but it been going here and there. And the pain is like- the pain is like painful like I’m having my period, like cramping real bad cramping like you’re almost coming on your period, that pain. But I went to McKeesport hospital, but I wanted to go to Magee. They gave me pain pills. Two pain pills. I guess it's whites?

A white one and silver thing that you pull apart.

I: Hmm.

P: I'm not taking those. Magee didn’t give them to me, so I'm not taking them. I don't know if it's gonna hurt my baby or not, but they say it don't. But I don't believe McKeesport.

I: Mmm.

P: So, I'm not doing that.

I: Are you, like, what- what have you been doing instead? Anything?

P: I've been staying home, laying down, watching TV and going to see my friends and come back in house.

I: Besides the stomach pain and stuff how else has it been going?

P: It been going good.

I: Mhm. Alright. Let's just jump right into kind of the meat of the interview, what are your thoughts about marijuana use?

P: It stinks.

I: Can you tell me more?

P: and it's not good for you and I don’t like it.

I: What are your thoughts about tobacco use?

P: Well, worse.

I: What do you-

P:I don’t even like the smell of that.

I: Do you know people who use marijuana and tobacco? Or tobacco?

P: My mom smokes cigarettes and my dad smokes cigarettes.

I: Mhm.

P: And that’s it.

I: What do you think about the use of them during pregnancy? like marijuana?

P: What you mean by that?

I: Like, what do you think, um, about using them during pregnancy like why women use them or if it's okay to use them, if it’s not okay?

P: It's not okay. I heard it's not okay to use them because if you have weed in your system y our baby can get taken.

I: Where did you hear that?

P: Like my aunt told me. My friends told me. The people that is pregnant that have been pregnant before, and that is pregnant that had kids before me. They say that if you smoke weed and they found weed in the system your baby can get taken. And my boyfriend's mom told me the same thing. That's why I don't smoke while I’m pregnant. I don't like, I don't like smoking. When I found out I was pregnant I stopped smoking. I'm not losing my baby for nobody. I’m not- Everybody said it’s good for you to smoke and it helps you, but no, I'm not losing my kid.

I:When you say smoking do you mean marijuana or tobacco?

P: Marijuana.

I: Okay.

P: Now, cigarettes, no.

I: Yeah.

P: Now marijuana, like weed, everybody says it’s good for you while you're pregnant, but no it’s not. You can get your baby taken.

I: Who has been saying it's good for you then that you know?

P: Like. Like. My friend’s friend said it’s good for you, but they used to smoke while they was pregnant. But I don't know.

I: In terms of being like good for you, what do you think they meant?

P: Like it’s healthy for your baby. Your baby can get bigger or something. Like, no? Is that true?

I: Um, do you think it's true?

P: No, cause my aunt was pregnant, she was smoking.

I: Mmm.

P And I'd be looking at her, when she told me- when she told us that she was pregnant, I'm looking at her like that's not good for you.

I: Mhm.

P: And alcohol and like can wine do anything to you?

I: Wine?

P: Yeah.

I: Um, it's an alcohol like the other types of alcohol.

P: Some people.

I: So, the people that have- some of your friends and your aunt and your boyfriend's mom who have told you that, you know, it could result, if you use marijuana, it could result in your baby getting taken away is there- does anyone you know had that happen to them?

P: My, um, my sister's friend did cause she was smoking with her baby. She was smoking. They found marijuana in her system, and she got her baby taken.

I: What do you think about that?

P: It's like. It's dumb. That’s your fault. You're dumb Why would you want to smoke then you got your baby taken? That’s dumb and retarded? What? On the phone with YoungMom. A meeting with YoungMom. (Talking to background). Happy- hold on- Happy Birthday! She can’t hear me. Excuse me, ma'am Happy Birthday. You're welcome (to background). But, um, my bad. Go ahead.

I: Yeah, you were just talking about, um, someone you knew who has their baby taken.

P: Yeah, and it’s not- My sister. My sister told me and stuff because my sister stopped smoking when she found out she was pregnant and it’s not good. Like why would you? Why would you want to get your baby taken? That’s dumb. You could’ve smoked after your pregnancy or whenever. That’s dumb.

I: Why do you think other people continue to smoke during pregnancy, then?

P: Cause they said it’s good for them and they be stressed out and they want to like get high or something to relieve their stress.

I: What do you think about children like being taken from their mothers because of marijuana use? Do you think that's okay, or do you think that shouldn’t happen?

P: That's not okay, but if parents want to be dumb that's on them. That's on them and that’s dumb. Like why would you want to do that?

I: Um, jumping back a little more comparing marijuana and tobacco, do you think one is safer than the other?

P: No.

I: Okay.

P: Cigarettes is worse.

I: Okay, you do think cigarettes are worse?

P: Cigarettes can get you cancer.

I: What do you think about using marijuana and tobacco together?

P: Oh no. I would be dizzy if I do that.

.

I: Why do you think you'd be dizzy?

P: Cause you’re smoking weed and smoking a cigarette at the same time. That’d probably get you lightheaded.

I: Is that's something you've ever done before?

P: No.

I: Um so, has your doctor talk to you about marijuana and tobacco use during pregnancy?

P: My doctor said it’s not good at all.

I: Did you feel comfortable talking to them about it when you were at your appointment?

P: No, I was okay.

I: No. Okay. Um, what do you think about like the medical legalization of marijuana here in 2018? Do you think- what do you think about that?

P: What you mean?

I: Like do you think it's good that medical marijuana is available? Do you think it's bad?

P: No. I think it’s bad because you never know what’s in your weed.

I: You know, do you know anyone that has used medical marijuana?

P: (Background noise) Huh?

I: Do you know anyone who's used medical marijuana? Had a medical card?

P: No, what did you say again can you repeat it again? My bad

I: Do you know anyone who's used that like medically like gotten a medical card.

P: No.

I: No? okay.

P: I didn’t know they had medical cards.

I: So, how old were you when you were when you first tried marijuana?

P: I was like 17, turning 18.

I: Mhm. What was that like for you?

P: At first, I was like dizzy because it was my first time doing it. But when I started getting used to it, I wasn't dizzy.

I: Mhm.

P: But I always like- I don't smoke with other people, because I don't know. I don't know what weed they get it from, and I don’t know if they add other stuff in it. somewhere. Somewhere (to background). I never know if they put heroine or if they put anything in it. Like any drug that can make you be high or anything. I don’t know.

I: Can you tell me more about like when you first tried like why you first tried it at that time?

.

P: Like I saw like all my friends doing it and I just wanted to try it.

I: Were you with your friends when you first tried or is that something you did alone?

P: I was with- I was with my friend, but we was like- we was like- I was with my friends.

I: Mhm.

P: I finally told my mom that I started smoking weed and I got in trouble.

I: Do you mostly use like blunts, joints, bowls or would you like use-

P: No.

I: as an edible? What would you use?

P: Blunts.

I: Why is that?

P: Cause it’s like I don’t use nothing else. Only blunts.

I: Mhm. Have you ever tried any other forms or is that all you've ever used?

P: Oh.

I: Have you tried any other ways?

P: No.

I: So, you told me, one of the kind of, factors on you quitting was learning that like your baby could be taken, what, what were other things that influence you to like either continue smoking or quit smoking during pregnancy?

P: What you mean by that?

I: Like was there anything else that influenced you or that helped you make your decision?

P: Oh, it helped me. It helped me. I'm not doing it.

I: Can you tell me more about how you feel when you use marijuana?

P: How?

I: How it feels when you like are high, when you use marijuana?

P: It just like relaxing. Just lay back and watch TV.

I: And how do you usually feel like beforehand.

P: Calm.

I: How about after the high is like- after you've come down from the high?

P: Still calm. I just be watching TV.

I: Okay. Are there certain situations or like time where you are wanting to use it more?

P: Huh?

I: Are there certain situations that kind of like make you want to use marijuana or like ways you feel?

P: No.

I: Okay, is it like a way you deal with stress, or is it really just like more of a fun thing for you?

P: It's like a fun day for me like fun thing for me.

I: Okay. How long ago did you quit?

P: When I found out I was pregnant.

I: Do you remember how long ago that was?

P: That was… May.

I: Okay. Did you quit-what was the quitting process like?

P: It was nothing. I just wanted to do it. Like it wasn't- what’s the- it wasn't doing nothing for me. It was just like- I stopped doing it because you never know like my one friend, she got high off of something and somebody put something in her weed and she didn't know. And she got it off this one person or one time got it off this one person and (unclear 14:30). I will never- I don’t want to smoke no more because of that.

I: Did you just quit all at once or was it something where you like cut down over, over time?

P: Yeah, I just quit all at once.

I: Can you tell me more about your quitting process?

P: When I found out that my friend was in the hospital because of that, because she was like super high, and she was like goin’ psycho. I stopped doing (unclear 15:03). I said, “alright, I’m not even going to smoke no more” because I don’t know what y’all be putting in the weed nowadays.

I: Mhm.

P: And I’m not losing my life for nobody.

I: Had you ever tried to quit before you found out you were pregnant?

P: Like, I was doing- I was like something here and there, but it was not like heavy like how I did before.

I: What were those times like?

P: It wasn't nothing.

I: Have you noticed since you quit suddenly kind of like any situations where you- is it something you still crave or not really?

P: No. I crave food.

I: Did you notice any side effects when you quit?

P: No.

I: Do you think this is something you would talk to your doctor about?

P: Yeah.

I: What have other kind of told you about talking to your doctor, like any messages or influences that other people have given you?

P: Nothing.

I: Nothing? Has anything else influenced you in deciding whether to talk to your doctor or not?

P: No.

I: Where do you learn about like marijuana and tobacco use and its effect on pregnancy?

P: When?

I: Where… and when. Sure.

P: Like other people was just sitting here- like other people, like all my family was sitting here tell me about it and my friends were sitting here telling me about it.

I: Um, back to tobacco a little bit, why do you think like that's something that you've never really used.

P: Cigarettes?

I: Yeah.

P: Cause they're nasty and you could get cancer.

I: Do you think there's anything different about talking to me like a researcher versus talking to your doctor about something like this?

P: No.

I: No. Okay. And like in an ideal world or or what could a doctor do to help you feel comfortable sharing more about your marijuana use?

P: Nothing.

I: There's nothing they could do?

P: No, I would still talk about it.

I: You would or you wouldn't?

P: I would. Can you throw this away (talking to background)?

I: So, what- When you are your doctor’s appointment did, they give you any information about marijuana use or tobacco use during pregnancy?

P: No.

I: Like any pamphlets or anything like that when you were leaving?

P: No.

I: And you touched briefly on like that they they’d asked you about marijuana and they told her was bad, can you tell me more about that or if they asked about tobacco also?

P: They was just saying it’s just like bad for you in your pregnancy.

I: Did anyone else at your appointment talk to you about like social worker or front desk.

P: No.

I: No. What do you help- what do you think would help like women, young women get information about marijuana use during pregnancy?

P: Hmm, I don’t even know.

I: Do you have any ideas about like what the best way for them to get information is?

P: To ask people.

I: Like who?

P: Like, your friends if you know them. Like anybody that's been pregnant before, they can really like sit here and tell you.

I: Yeah, that's a good point. You’d brought up like some of your friends were telling you that marijuana could be good during pregnancy and some of them are telling you, you know it was bad because your baby could be taken away, how did you decide like which ones to like listen to?

P: That your baby could get taken.

I: That's how you decided like that was more serious.

P: Mhm.

I: Has quitting- stopping using marijuana affect any of your relationships?

P: No.

I: No. You said marijuana was something that you usually use alone has, um, has that always been the case?

P: Like smoking by myself?

I: Yeah.

P: Yeah.

I: Has always been kind of for the same reason that you're mentioning.

P: What you mean by that?

I: Like you were saying it's because you're worried like something could be- you don't know where other people are buying it and stuff.

P: Yeah.

I: Was it always for that reason, or was, was there a time where you kind of like just preferred it or something?

P: I would just smoke by myself, because you never know what people put in it.

I: Have you had an experience where there's something in it?

P: No.

I: No. But you know people who have. Um, can you tell me how like COVID, the pandemic, has affected your marijuana use at all?

P: No.

I: You don't think it has?

P: Because I don't care about it.

I: Did you- did it like make you use more or less anything like that?

P: (shakes head)

I: No? How about your pregnancy? Has the pandemic affected your pregnancy?

P: No.

I: Okay. Um, is there anything I didn't ask that you would want to share?

P: No.

I: I'm gonna take a minute to look through the guide and make sure I got everything.

P: (talking to background)

I: I would like to explore, just like a little more about like what made you feel comfortable telling your doctor about marijuana use even though you kind of knew there might be risk of like your baby being taken away if you were still using it. Like how did you decide to talk to them?

P: It was just like a normal conversation. I'm not gonna lie about it. It's a normal conversation.

I: Well, did you have any like worries going into the conversation?

P: No.

I: And did you like, like how they handled it after you talked about it? Like what- what happened afterwards or can you walk me through the conversation?

P: Like. I don’t know. It’s like hard to explain. I don’t know.

I: Well, is there anything that you would change about it like anything you wish they'd done or wish had happened?

P: No.

I: Okay. When, I guess changing gears again, Wwen you did quit marijuana what kind of like support did you have? Who were you like talking to you throughout that?

P: My best friend.

I: Can you tell me more.

P: Like I was talking to her and she said that's a good thing, and I was just like talking to her about it. It's like normal. Like she was telling me to stop smoking cause it’s not good and like you never know what people put in your weed.

I: Mhm.

P: Like me and her was talking about the same thing cause she used to smoke too. But she don’t smoke no more like she- Like she stopped.

.

I: Seems like you've had, like some of the same concerns about, you know, something potentially being put in that you didn't expect in your weed for a while, what like is there a reason why- why hadn’t you quit sooner do you think?

P: I did quit sooner. I did quit sooner.

I: But you went back you said right? Like You'd tried to quit before you started using it again?

P: Like- Oh, it was just like for the fun.

I: Mhm.

P: It was like for fun.

I: Can you tell me more.

P: It was just like for the fun and it was like a birthday party that I was going to and everybody was just smoking.

I: Like what makes it fun like what do you mean when you say the fun?

P: It was like- I was just going to like birthday parties and it was like birthday parties, and we will assist like having parties and just having fun. Just drinking and smoking, but I wasn't drinking.

I: Mhm.

P: I was just smoking.

I: Let me once again see if I’ve just missed anything. Do you have any more thoughts about like what would help you or other young women get information about, about marijuana use?

P: Huh.

I: I kind of asked already, but do you have any thoughts that that have come to you since then, because you said you weren't sure, like what would help young pregnant women get information about marijuana and tobacco use?

P: I don’t know.

I: Okay. All right, um. I really appreciate your time today. I want to ask again like is there anything I didn't ask you?

P: No.

I: or that you want to add? Okay.

P: No

I: Any any final thoughts at all. Alright, I'm gonna go ahead and turn off the recording.
