# Supplementary material for: Experiences of postpartum mental health sequelae among black and biracial women during the COVID-19 pandemic
Source: BMC Pregnancy Childbirth. 2023 Sep 4;23:636. doi: 10.1186/s12884-023-05929-3 (PMC10478375; doi:10.1186/s12884-023-05929-3)
Supplement: Supplementary file 5 — Supplementary Material 5 [file 12884_2023_5929_MOESM5_ESM.docx]

**Supplemental File 1.2 Interview Transcript with Interviewee 5198**

Q1 5198

Date Administered 1/13/2021

Interviewer: 50

P: Yeah, yeah (child speaking).

0:02

I: I see those (researcher responding to child).

P: Mommy, yeah (child speaking).

0:06

I: Yes. So how has, how have you been feeling so far in this pregnancy. How's it been?

0:09

P: Terrible. I have a headache too (child speaking).

P: You have a headache too? (Speaking to child)

0:23

I: Sorry, you have a headache (to child). What's been terrible about it?

0:24

P: The nausea, that’s the only thing.

0:28

I: Is that like normal for your pregnancies or like new with this one?

0:32

P: Okay, normal. Last time I had something. I forgot what they called it. Um, I lost, uh, I went down to like 70 something pounds because of the nausea and stuff. So, I didn't eat anything for days, literally. So, this time it’s not that bad.

0:52

I: It's not that bad this time?

0:54

P: Now before I didn't have any medicine because I wasn't even in the country. So pretty much dealing with that myself.

0:55

I: Has the medicine, how is it effective for you for that so far?

1:03

P: Yes, I haven't thrown up at all. So, just nauseous.

P: Is she coming to my house? (child speaking)

P: No, she’s in her own house. (responding to child)

1:13

I: We're going to stay in our own houses to keep us safe (responding to child).

1:15

P: Okay.

1:19

I: Okay, but I mean, you seem cool. I'd love to meet you (responding to child). But yeah, that's…

I'm sorry that you went through that.

1:26

P: Yeah.

I: It's scary too.

1:30

P: Oh, yeah.

1:32

I: Uh okay, I'm gonna kind of like dive right in. Other questions. Uh. Generally, like, what are your thoughts about, about marijuana use in general?

1:44

P: Don’t do it.

1:50

I: You don't do it, or nobody should do it or what do you mean?

1:52

P: Nobody should do it.

1:55

I: Okay, tell me more about that.

1:57

P: Umm, according- I don't know anyone personally who does it, but according to what I hear it’s more harm than good. So, avoid it.

2:00

I: Okay, where do you, where do you like, get your information from

2:09

P: The internet. Yeah, I don’t know anyone personally who does it. So

2:15

I: So, like nobody in your like social system. How do you? When you find information on the Internet. I want to know. Like, how do you determine it's trustworthy?

2:28

P: Ummmm. Number one, if it's on Facebook, I'm not listening to that. No, like if it's an article from, like, you know, news sources or whatever then I guess I’ll believe it. And I don't know if you watch that kinda stuff. But, hey (speaking to child). People like to talk about their personal stuff on YouTube, their experiences with stuff like that. So, I also take that into account.

2:50

I: Okay, so a little bit of like, like act like, like studies and stuff you read, and then also like people's personal-

2:57

P: Experiences, exactly.

3:05

I: Experiences. Okay. Have you ever used marijuana at all?

3:07

P: No.

I: Um and no one that you know really has either?

3:12

P: No, I don't know a single person who has done it.

3:16

I: What kind of, what do you think prevents them from trying to… like from trying it. Like, what are your-

3:20

P: Religious beliefs.

3:26

I: Anything like specifically or just general

3:33

P: Well, I'm Muslim and in that in the religion it's like you're just supposed to stay away from things that can intoxicate you and marijuana does that, so.

I: Mhmm

P: So, that’s automatically don’t do it.

3:50

I: That makes sense. It's clear.

3:52

P: Anything that can harm you physically, you’re supposed to stay away from it. That's part of it.

4:00

I: Is that true for tobacco? Also, or is that, is there a difference with tobacco that you, see?

4:01

P: I don't really know anything about tobacco. Isn't tobacco, just like I don't know, cigarettes that you chew on? I don't know.

4:15

I: No, I mean like cigarettes at all, like smoking cigarettes or whatever.

4:17

P: There's no. the, the harm outweighs the good. So just because you feel good. What are you doing to your body? It's hurting your body. It's hurting your lungs. So automatically canceled. Can’t do it.

P: That’s my stuff (child speaking).

P: Okay.

4:39

I: That's her stuff (speaking to child). What about, do you think there's a difference like when you're pregnant?

4:46

P: Hey (speaking to child). (child crying). Okay, sorry about that.

4:57

I: Like her little safe space in there. She's cocooned with mom. That’s beautiful. Um, I'm wondering, so that's how you feel about it in general. Can we switch and talk to how you might feel about it when people are pregnant? Like, what do you think about a pregnant woman using let's say marijuana?

5:06

P: Same rule applies, but I don't know... It seems like as a pregnant person, you would definitely want to stay away from that kind of stuff, because you don't know what that's going to do to the baby.

5:24

I: Yeah. So same, it's very similar. And then also you have to worry about the baby kind of

5:33

P: Exactly. Double risk.

5:38

I: Double risk. Okay. And is that how you feel about tobacco use when you're pregnant too?

5:40

P: Correct.

5:46

I: Do you think that one is safer than the other, just from your opinion.

5:50

P: One is safer than the other?

5:54

I: Yeah, just wondering.

5:56

P: You mean between marijuana and tobacco.

I: Yes.

P: Or?

6:00

P: According to what I hear they say marijuana is safe.

6:02

I: Okay.

6:06

P: According to what I hear, but I don't know for sure.

6:07

I: Yeah, um, has a doctor talked about this stuff with you at all?

6:12

P: I don't remember talking about this kind of thing with the doctor.

6:18

I: Do they, so they did, did they ask you any questions about smoking or

6:22

P: Oh yeah, yeah, at the beginning of every appointment they always ask that.

6:26

I: Okay, do you feel comfortable sharing with them?

6:30

P: Um, if I did that kind of stuff. Of course, I would tell them no. But since I don't, then I tell them no.

6:39

I: Why would you tell them no if you did it? I'm wondering.

6:45

P: If I actually did it then, number one, that could make me like a high risk or something like that. Maybe they could take your child away or some weird stuff.

6:49

I: You know, I, this is like all the kind of information that we want to get.

6:58

P: Oh okay. Wait, wait, wait (speaking to child)

7:06

I: I, it's, I'm watching how aggressively you have to rock her and it’s so interesting.

7:11

P: What you want? Okay hold it. Sit away from mommy (speaking to child)

7:25

I: Where do you have you, do you know somebody that's had like their kid taken or anything from that? Like, where does that kind of like knowledge come from?

7:36

P: Um, I, I don't want to. It's not the same situation about drugs or anything, but there have been people in the past, who attempted to get us taken away from my parents. So, I'm kind of familiar with how that works.

7:56

I: Mm hmm. Do you feel comfortable like sharing more about that?

8:02

P: Um, sure. So, um, you know, my dad. He has 12 kids. It's a lot of us, and he's not rich, to be frank. He’s not rich. So, some people, specifically ex-wives, would use that and contact health services and try to get them to obviously split us up. So yeah.

8:07

I: My mom is one of 12 kids. Big family.

8:34

P: That’s a lot

I: Yes. Big Catholic… 70 billion cousin family.

8:41

P: So, are you doing your part to continue that legacy?

8:45

I: No, no, no. Not for me now. Um, that's a great question, but I'm supposed to be asking you questions Ma’am.

8:54

P: Alright, fine.

9:01

I: Yeah, I'm sorry that you went through that with the system, though. That's can be really stressful for the family.

9:03

P: Definitely

9:10

I: Yeah, did you, did your like parents like your dad or anything what was their like messaging around like marijuana and tobacco use growing up?

9:12

P: My parents are very religious. So definitely “hell no”. So, no, no. We don't even have those discussions you already made it clear. That's not cool. That's it. Like we don't even talk about that.

9:22

I: What would, what do you think the consequences would be.

9:35

P: From my parents if I did that.

9:40

I: Yeah, or just from your community like.

9:43

P: They'll be very, very, very, very disappointed. There's nothing they can do but just very, very they’ll be disappointed. And surprised.

9:45

I: Has that happened to anybody?

9:53

P: No, no, I don't know a single person. No.

9:54

I: That's interesting.

10:00

P: Mom (child speaking). Yes (speaking to child). Like I said, like we're Muslim. So that's very clear that that kind of stuff is off limits. So, if someone is doing it, they're not going to make it obvious and they’re not going to tell you that they're doing it, just because.

10:07

I: That yeah. That makes sense to me that they would kind of hide that.

10:24

P: Yeah.

10:27

I: Hmm, let's see, I'm thinking. I'm wondering, like, if there's a difference in talking to someone like me like a researcher and talking to your doctor about this stuff. Do you feel like there's a difference, or is that pretty similar?

10:45

P: I feel like there's a difference. If I told you today “yeah, I smoke marijuana”. I do this little, blah blah blah, who are you going to tell?

11:00

I: No one.

11:10

P: Who are you are going to tell? Would you tell my doctor?

I: No

11:14

P: No. Okay, so then there's a difference. You wouldn’t tell my doctor. There's really nothing you can do to hurt me. I guess, if I told you that.

11:21

I: That makes sense there's less consequence, maybe.

11:29

P: Exactly, yeah.

I: Yeah, I can see that I'm why I'm wondering, why do you, do you have an idea about why people might use marijuana or tobacco, like in general. Why, why do people. Why do people do that?

11:32

P: My age specifically? Maybe because it's fun and their friends do it. Peer pressure. Everyone's doing it. That's about it.

11:57

I: What do you think would make someone continue to do it while they were pregnant?

11:59

P: Umm, maybe they have this idea that it's safe. Like you keep hearing on the news and stuff like that. Marijuana is actually safe. It’s not like this. Blah, blah, blah. So okay. It’s not gonna affect the baby or me. So why not have some fun and be pregnant?

12:04

I: Do you hear that about tobacco or do you hear something different about tobacco while you're pregnant?

12:21

P: Different definitely different.

12:26

I: What do you hear?

12:28

P: Smoking? Bad, death. Pretty much. I never heard anything good about tobacco.

12:31

I: Where do… you do hear stuff, you think, that's good about marijuana?

12:37

P: Yeah. Hey (speaking to child). I never heard any like physical consequences for marijuana. I've never heard anything like okay you smoke, you get messed up lungs you get cancer, but I never heard anything like that about marijuana. At all. Hey. Okay. I got you. I got you (speaking to child).

13:08

I: So, there's no, like, you're not getting any information about what it might do.

13:12

P: The dangers. Yeah, I haven't heard anything about that really. Other than it makes you feel good and safer than whatever else people do.

13:18

I: Where, where is that, like, where are you hearing that at?

13:26

P: online, news, people who support that stuff. Like oh, we want it to be legal. There's even petitions that I've seen for that. So, I don't know where they get their information from but…

13:35

I: Yeah. Do you think it's… Do you agree with that?

13:43

P: Do I agree with that? One second. One second (speaking to child). If I believe it, that's all because that's all I hear, but I can't say for sure. Yeah, that's true. Um, isn't it, uh, legal in California?

14:00

I: I think so in Colorado, a few places, I think.

14:15

P: So that would give me the indication, like, okay, maybe it's not that dangerous because it's legal in a whole state. But then again, slavery was legal. So, I don't take that into account. Yeah.

14:30

I: And the laws don't always, yeah, protect people, they're not-

14:33

P: Definitely not.

14:36

I: Right laws are often about money. For sure. Yeah. Um, what do you think, is there a way that you think that like doctors can provide women with this information, like information about marijuana and tobacco and like have conversations about it while they're pregnant?

14:46

P: Hmmm. That's a good question. Um, I don't know. I know. I know when you're at your appointment. Hey, wait a second (speaking to child). I know when you're at your appointment they’re like, “oh, do you do this?”, but I've never told them, “Yes I do this”. I don't know what their procedure is for someone who does do it. So, I couldn’t say.

15:05

I: Mm hmm. Mm hmm.

15:22

P: I don't know.

15:24

I: What would you want someone to do if you were like, if you needed that kind of help.

15:25

P: If I needed that kind of help and I want to get help. Um… hey, hey (speaking to child).

Um, if I wanted to get help and I had that problem, then I would say doctor should always have some sort of resource on standby. If that situation was to occur. I don't know what kind of resource or how people handle that. But yeah.

15:54

I: Okay, so we had, you said something a little earlier about, like, like services being involved. Like what's your opinion on like, do you think it's okay for services that to be involved about, you know, marijuana use during pregnancy. Like what's your… What do you think?

16:18

P: Definitely. Um, why? Because you have a whole baby your stomach. It's not only about you. So, they have to make sure everyone is okay, not just you.

16:20

I: Mm hmm. I'm thinking. I'm thinking about what to ask you next. What do you think like your experiences with your healthcare provider like what do you think, is there anything that could be done to make you feel like more comfortable during those visits or like, you know, how has that relationship been for you?

16:56

P: Actually, Magee specifically, amazing. I love how I love how they're set up. I don't really have any concerns with them when it comes to getting help, except for behavioral health

17:11

P: That's my only problem with them. You kind of gotta run around to find somebody. It’s kind of annoying. So, if I really was reaching out. Well, I don't know if that comes in the same category substance abuse and behavioral health or not. But if I did want to get help. Would you go to some? Would you go to behavioral health for that? For substance? Yeah.

17:46

I: Yeah, I think it's a mental health concern. It's different. But, you know, same

17:48

P: Yeah, Yeah, I don't have. I don't have anything to say that they needed approval on except for that.

17:58

I: Yeah, okay, um, that's really interesting to me. So, what do you mean, has it been hard for you to like access help in a certain way or like?

18:01

P: Um, I was trying to find a therapist or whatever you want to call it and I had, I had a run around like they gave me a whole run around to get that sorted out, so.

18:16

I: Did that end up happening for you?

18:23

P: Yeah, I did actually have my first appointment few weeks ago. So, that's awesome. If it was any other person, I would have just gave up like whatever I'm not doing this, no more, but I don't like unfinished business. So, I just went with the flow.

18:30

I: Yeah. So, what did you have to do to get that to get that kind of help?

18:42

P: Um, so at Magee, I spoke with a social worker. They gave me the behavioral health number. I call that number.

18:47

P: That person directed me to someone else. That person didn't answer. I call that same number again. They directed me to somebody else. That person didn’t answer. Call them again and then the whole time they were able to help me. So, I know I didn't help me before. So, it was a process.

19:10

I: That, no. I mean, what I'm thinking is like, you know, if someone wanted resources about marijuana, like using less marijuana in a similar manner as you wanted resources about behavioral health, it would. Like that could frustrate them. Like that's a lot of perseverance for you.

19:31

P: Exactly like and you know okay whatever. Screw this. If it happens it happens whatever, just because of stuff like that. I think specifically, specifically mental health you shouldn't be getting a run around because you don't know what's happening with somebody. That was-

19:47

I: Hard to ask for help.

19:48

P: Yeah, so that wasn't cool in my opinion.

19:50

I: Yeah. No, I really appreciate you sharing that because I think that's like, I'm sorry, you went through it, but the experience is valuable here for you to share anything.

19:59

P: Right. Mm hmm. That's annoying.

20:05

I: Yeah.

20:08

P: Especially during a time like this. Like if you want me to do something. If you're helping me send me directly to the source, so I can get everything figured out don't refer me to California or whatever. And then just for me to go back to you. And I don't know, whatever. Weird stuff.

20:10

I: No, you know, I like hearing this, it's like you had to call someone, call someone, call someone to be sent back.

20:32

P: Back to that original person in the first place and figure all that out. So, that was stupid. Sorry.

20:36

I: No, I mean, that's a real criticism. I’m okay with negativity when it's like well placed, you know.

20:44

P: Yeah.

20:48

I: Yeah, what could they do differently, like, what's a better

20:50

P: I don't know. First, I have a question for you. First, is the Magee Behavioral Health center or whatever, only for pregnant women?

20:57

I: I don't actually know. So, I'm employed by Western Psychiatric actually, in like the research unit. So, we're pretty far removed from like Magee Behavioral Health

21:17

P: So, if I'm… Magee told me to call you guys.

21:22

I: To call with WPIC?

P: Yeah.

21:25

I: Yeah.

21:26

P: Told me to call you guys and then. Forget about it. That’s confusing. I don't know. I don't know the difference. I'm like, okay, am I calling Magee Behavioral Health or am I calling West Penn. when I think of you guys, I think of a criminal insane asylum. So, I don't really know.

21:34

I: No, everything you're saying right now is so important, and I want to like express very clearly that like I appreciate what you're sharing because it is very important. Because it's, it's, like, “what's Western Psych?”

21:57

P: Yeah, I don't know anything about that place at all. I just sounds like something that criminals go to. Ok. Ok. Ok. Ok. (speaking to child)

22:10

I: So, did they end up (child crying) for your behavioral health or?

22:20

P: Um, yes, I end up, I did end up speaking to someone at Magee. So yeah, like I said. That's weird. Yeah.

22:32

I: Weird system.

22:33

P: Huh. Wait a minute, is I'm sorry, one question about Western Penn or I don't know the name for it. You guys treat all different kinds of people. Right?

22:40

I: Western Psych does. Yeah.

22:50

P: Mm hmm. One of my friends who went there. At one point, I'm not sure. I think she said she did.

22:51

I: Yeah, they have like, from what I understand, they have a lot of different programs and a lot of different you know from very severe mental illness where you need inpatient in hospital care to you know, not that any mental illness isn’t serious but to, you know, weekly therapy or something that's less concern for harm for yourself and others, I guess.

23:14

P: Do they do it for kids and adults?

23:27

I: They have kid’s programs there too.

23:30

P: Okay, maybe she did, yeah.

23:32

I: I do wonder though if your experience of like finding it hard to kind of get your foot in

23:35

P: Yes, exactly. It's, I don't as a person who has never done mental, I don't know, who's never talked to a mental health doctor or anything like that. Don't know anything about it. Um,

I, I could honestly just went along went online to what betterhealth.com those websites mental health websites and did it, but I didn't trust that. You know it's easy. And I don't know. I never heard about it until coronavirus I didn't really trust it. So let me just try and go through, um, my insurance or whoever, I'm working with, and see how that works and it was very difficult. A normal person would just give up.

24:14

I: So, do you have an idea of how to like make that like OB to like behavioral health process easier. Like what would have helped you?

24:24

P: Easier said than done, but when it comes to Magee and it's a lot of pregnant women there. You should always have someone who specializes in that stuff on standby in case somebody was having problems and they were interested to go. Always have someone on standby. Not let the patient do the research.

24:41

I: Do you think the like, like the social worker wasn't the right person for that you want like?

24:59

P: She wasn't the right person to give me the resources that I needed?

25:04

I: No, I'm wondering, like, I'm trying to think about how to like ask this question without like biasing you or something. So, I'm trying to think about, like, you know, I feel like it could be said that the social workers kind of supposed to be like that support person there, but I’m hearing that wasn't your experience at all.

25:27

P: She was, she was helpful, she just gave me a bunch of papers with numbers on them and names. So, I that that means you need to put the work in and get everything set up. Like I'm not gonna do that for you.

25:30

I: Okay. That's useful, which is, yeah, I see the merit. Do you think that, like, that's okay to do or do you think people need more, like you know, kind of support, like hands on?

25:59

P: When it comes to mental health, definitely because, like I say, you don't know what someone is going through or feeling that time. They might end up doing something that could, you could have prevented all because of that, I don’t know, lack of help and assistance.

26:03

I: Mm hmm. Yeah, so. But yeah, getting a lot of papers. When you also don't know an area or don't. Exactly, yeah. It could be really hard.

P: Mhm.

26:30

I: But if you could be like really honest. Like, what would you have rather happen, then? For you.

26:44

P: What I would have rather happen? I would have liked someone to explain to me how.

That area works. Where you would start. And what you're hoping to achieve at the end of it, not just give me a number that says, call this when I literally have no idea with how the behavior health system works at all. So, yeah.

26:55

I: Thank you for sharing that. Health systems can be complicated. Like so complicated

27:10

P: Yes, like can you imagine all the people who don't even try to get help because of that.

27:19

I: Yeah, or like the people that aren't as savvy as you.

27:23

P: Exactly. Okay, so I know how to do my research. That's why they give up. Just any old person really like “Okay, then whatever. I guess my mental health is not important.”

27:35

I: Yeah. Yeah, someone that doesn't have support, family.

27:43

P: Mm hmm.

27:47

I: The internet. Like ohhh, you get lost so easily. I am curious about the, you know, I feel like this is kind of on everyone's mind. Like, how has the COVID 19 pandemic like affected this pregnancy for you at all? If it has?

27:56

P: Um, I don't think it has. I'm pretty much done exactly the same thing that I did before COVID. I'm always, I'm a homebody in general. I don't leave the house really at all. Except to go to the store. So, nothing has changed for me. I’m in the house this pregnancy. So, nothing has changed really.

28:30

I: How do you feel about like going to the doctor in the hospital for your visits and stuff during it?

28:33

P: I don't mind at all. I don’t have any concerns about that. I just go. Since now, you know, they're doing like half telehealth and half physical, so. They even gave me a blood pressure cuff, which was kind of cool. So, I can do it from home.

28:55

I: Yeah. That is really cool. I the the kind of like last question I would like to ask is, like, um, what first, like, you know, do you have any feedback about questions that I'm asking? Like that. Not that are like offensive, but I'm not asking in the right way, or do you think I'm not asking something that I should be? Like what, you know, help me be better at this if you don't mind.

29:16

P: Questions that you're not… You're fine. I don't really see anything. It’s just if you ask me a question I answer. I don't see anything.

29:35

I: Yeah, you're pretty open.

29:40

P: Yeah, I don't think. Would anybody participate in something like this, if they weren't open, you can ask them.

29:41

I: I think that I could see myself at certain points in my life saying, oh, 20 bucks. Yeah, and then putting in very little energy for it. Sure. That's something

29:50

P: Okay, I can get that.

30:03

I: And that's okay. If that's where someone's at. That's where they're at, but yeah.

30:09

P: For sure. No. No, you're perfectly fine. You made it clear, like this is confidential and all that stuff. So, I feel like if somebody else were to do it they would feel safe to spill all the tea.

30:11

I: Thank you. I appreciate that. Um, do you have questions for me before I let you go about your day?

30:27

P: Ummm. When is the study going to end?

30:37

I: Oh, so it depends on how much you want to do of this study, like.

30:39

P: Not for me. In general.

30:46

I: In general, well the, the study that is funded until for like three more years or so.

30:48

P: Mm

I: So, we'll be recruiting new OBs for the next three years and then hopefully if people are want to, we’ll meet with you, four times, you know, once in each trimester. And then once after your baby is born.

30:58

P: Mm hmm. And then after those three years over then what

31:16

I: I think so. You're like asking super good questions that are like they’re like a little, it's like above my pay grade. You know, like, I'm just a wee research project assistant. So, but my, I think my bosses would love to get the study funded to do more assessments with the women and children that are willing throughout their developmental life.

31:26

P: Um, okay. So never ending.

31:50

I: Yeah, that’s a much more quick way to say what I just said in 1000 words.

31:51

P: Okay, got it.

31:56

I: Yeah.

31:57

P: Okay, that’s interesting.

32:00

I: Any other questions. See, those are great questions.

32:02

P: I don’t know. I don’t think I've participated in a study. Not that I can remember. I don't think I've participated in any studying. If I have, I don't remember.

32:07

I: How's your experience been of this one? What do you think?

32:17

P: It's very easy. It's not difficult at all. You just talking about yourself. So, yeah. Yeah, it's easy. I don't know. I don't see anything… difficult about it. Very straightforward.

32:38

I: Yeah. That's good to hear. That’s good to hear because sometimes it seems complicated to me, but…

32:42

P: No. Well, to you. Research assistant.

32:47

I: That was a. That was a good one. I could burn I feel like you just had.

32:54

P: and sorry. I know this is personal. Do you have a medical degree or something like? Sorry. So, like, what do you do? Like, what is your career?

33:15

I: So, the people that like get the study that plan it and like think of it and know design the questions to ask you. They have PhDs. So, they have their have doctorate degrees of research and then, I come from, like, a direct services background, actually. So, I used to work with families experiencing homelessness, but I would like to, to move to research now. So, my I'm actually almost done a master's in psychology.

33:33

P: Oh, I always say I'm going to be a psychologist.

33:56

I: Do you want to do that still?

33:59

P: Yeah, but nobody got money for that, but I used to always say that when I was younger.

34:02

I: I have negative money. From my student loans but yeah, that's true. I think you would be wonderful at it, though, you know, if you need a little encouragement.

34:19

P: Don’t gas me up.

34:22

I: If you got. I mean, I would, I think you'd be wonderful at it. Really.

34:23

P: Thank you. I appreciate it. Yeah, when I win the lottery one day.

34:30

I: Yeah, you have a lot of time too, you know,

34:34

P: A lot of time, I’m about to be a mother of two. I'm not even 21

34:38

I: Okay. In that light, but I just mean that I'm, I'm going to be 33 this year. And I'm kind of just starting the journey. Like, I started a little late.

34:50

P: Okay, I get you. You know, you still got a lot of time to, to continue your family legacy.

34:56

I: You slid that in. You slid that in

35:03

P: Even if you got to adopt.

35:07

I: Yeah, maybe, maybe, maybe one day. I just love the hard work that you guys do, that moms do, and and I know I'm not quite ready for that.

35:19

P: No, I think you're more than ready. You’re 33 and your grandparents had 12 kids so.

35:27

I: You know, they're good, and my family is good at that, you know, I got two sisters, they can pick up the slack for me. That's what I'm thinking.

35:35

P: No, you gotta put in the work too.

35:39

I: My mom would love you right now.

35:43

I: Well, I really loved talking to you. I can't wait to interview you again.

35:52

P: Yes.

35:58

I: It's gonna be really fun. I'm gonna call you during your second trimester for a bunch of things.

36:00

P: Okay. All my information is pretty much going to remain the same as it has for years. So.

36:11

I: Yeah, hopefully, we have some more interesting questions to ask us, so you don't get bored.

36:17

P: Okay.

36:22

I: I'm going to put $20 on your card.

36:23

P: Awesome.

36:28

I: And then if you have questions or think about anything after this interview, you can always call or text me.

36:29

P: Okay, no problem. Definitely. Thank you. I appreciate it.

36:35

I: Nice to meet you.

36:40

P: Nice to meet you too. You take care. Don’t forget the legacy.

36:41

Nicole Boss: By me
