# Supplementary material for: Experiences of postpartum mental health sequelae among black and biracial women during the COVID-19 pandemic
Source: BMC Pregnancy Childbirth. 2023 Sep 4;23:636. doi: 10.1186/s12884-023-05929-3 (PMC10478375; doi:10.1186/s12884-023-05929-3)
Supplement: Supplementary file 6 — Supplementary Material 6 [file 12884_2023_5929_MOESM6_ESM.docx]

**Supplemental File 1.13 Interview Transcript with Participant 5363**

I: So, how’s you’re pregnancy going so far?

P: it's fine, it's pretty good.

I: Tell me more. How have you been feeling?

P: Just regular morning sickness. Tired, really tired… That's really it. It's just the beginning so it's not really much.

I: And how have you been doing emotionally?

P: Oh very emotional if that's what you meant. yeah.

I: I guess, I mean like have you been feeling as like that kind of physical stuff and weighing you down and feeling happy like how-

P: Oh, I just felt like I cry about everything. yeah that's really more so it. Now other than that, yeah I could be happy.

I: Is the crying about everything, or like being more emotional with pregnancy or was it that way before?

P: Honestly, I don't remember how it was before, because it's so- like- it's been that long but- I think it's kind of the same with morning sickness and everything.

I: Right. Well, have you been able to kind of like find anything to help with the morning sickness or [have your doctors recommended anything]?

P: No, I just threw up. That's it, nothing much really.

I: Well, hopefully either it'll go away soon, or you can find something soon.

P: yeah I will- whenever I go to my appointment.

I: Well, jumping right into some of the interview questions, what are your thoughts about marijuana use in general?

P: Honestly, I don't really… I'm not gonna say I don't see no wrong with it, but like some people use marijuana for other things like… Medical marijuana. So you know, like it can be a purpose, why people smoke it, so obviously it's like a cure for something. That’s really it.

I: When you say it's like a cure for something, what have you heard about that, like what it can be used for medically?

P: yeah… can you repeat that?

I: Like you said, you know it's a cure for something it's used medically. What are some of the things you've heard it can be used for medically?

P: Autism, cancer… arthritis- I heard arthritis. Oh yeah that is a good one- anxiety. That’s really it really.

I: What do you think about the medical legalization of marijuana like seriously question 2018, what do you think about that?

P: I think… hold on one second (mumbles) Okay repeat that again.

I: You think about the medical legalization of marijuana.

P: Did they legalize it?

I: medically here in 2018.

P: I think that's pretty decent, because if somebody really needs to smoke weed then that's pretty okay like I feel like it's not bad for people to smoke weed all the time. It kind of helps out with a lot of things, maybe like… mood swings and sometimes they even being sick like the stomach ache or something. Some people use it for appetite.

I: yeah. Do you know anyone who's using it for a medical reason?

P: Oh yeah I know a couple, I know, like maybe only two people. Two people. They’re close.

I: Do you know anything about their experience with it?

P: No, not really. I just know that one of them, they use it for arthritis like she had real bad back pains or something like that, and she said that it will help her maybe sleep sometimes or not sure what else but she said that it would kind of numb her body. Because they would give her that, like they would give her a good amount of it. Of course she still got to pay for it, you know what I’m saying but they would give her a decent— — girl where did you get cheese from? I'm so sorry.

P: She… yeah I lost my train of thought she's crazy I’msorry.

I: That's okay. So what do you think about tobacco use in general?

P: It's not good, it's not good it's not good at all because, like… I mean when I smoke cigarettes after I have our first kid it took me, maybe like two months to start smoking cigarettes again, but I never would smoke them whole because they're like nasty so like I was in this pregnancy, when I found that I was pregnant I quit smoking cigarettes, maybe like two, three days later it's like it just happened I don't think cigarettes are good, though I know, honestly, but some people smoke on you know… a lot.

I: What makes you say they're not good?

P: They nasty. I feel like they do something to your teeth. Because when I was smoking them up, I got a hole in my tooth right now, and I really do feel like it was from smoking cigarettes. So that's not good. What else? …Tobacco- it’s strong, really strong. The smell of it is bad- maybe that's just because I’m pregnant though. I didn't like cigarettes at all when I was pregnant for both like they're just horrible.

I: yeah have you heard about any other health effects, besides some of the dental things that you think you've experienced?

P: I’ve seen like on commercials like holes in your throat. Not that it's funny but you know i'm saying like- that's about it.

I: yeah yeah, what do you think about the use of tobacco during pregnancy specifically?

P: Oh no I don't think it's good. I don't think tobacco is good to use during pregnancy only because I feel like it's too strong and something happens to the baby.

I: When you say “strong,” what do you mean?

P: I feel like there’s something in the cigarette. That I don't know what's in a cigarette, but I just feel like it's just something bad because when I would smoke it well, I did smoke, maybe, like my first pregnancy, I stopped smoking at 14 weeks, but I found out, I was pregnant at…Maybe… 11. So I stopped smoking at 14 weeks, and it will make me nauseous or give me bad headaches. Even when somebody smoked it around me like I would throw up.

I: What do you think about marijuana use during pregnancy?

P: Honestly- like no it's not good, but my last pregnancy, I did smoke my whole pregnancy, because of how my morning sickness was and when I gave birth my placenta was… it was very black, but my daughter- her pee wasn't dirty or nothing was wrong with her, she was fine like i'm not saying it's okay to smoke, while you're pregnant because everybody's different like like this pregnancy. Yeah I still smoke weed like… and it makes me nauseous so I'm stopping because I want to see if that's why I've been all nauseous like that. Because, it might be different this pregnancy like weed and cigarettes just make me nauseous. It's not good.

I: yeah do you have kind of a plan to stop or…?

P: Just just to stop.

I: Okay.

P: I just stopped smoking cigarettes like two days after that I was pregnant.

I: I want to hear a little more if you can tell me what it was like quitting cigarettes.

P: what?

I: What was it like quitting cigarettes for you during your pregnancy? Was it hard or..?

P: [“No” sound]. It's just like “I don't want it”, like my head or like my stomach whatever it was kept saying I don't want it, I don't want it and I just would never pick up a cigarette. And I tell you I didn't pick up one cigarette, not one cigarette and maybe like six weeks after I gave birth to her, I started smoking, but I wasn't smoking them whole like it was just cause of anxiety or postpartum or something. But I never really smoke cigarettes like that. I haven't even bought a pack, it'll probably be like one or two or something like that. They’re not really all good. They’re nasty.

I: You think between like marijuana and tobacco, do you think one is safer to use during pregnancy?

P: Yeah, weed. Because it does help my morning sickness like if I didn't have no weed or anything like when I was in my last pregnancy, I could not eat anything. I would not eat, I would just continuously throw up and not be able to eat anything. So I just smoked my whole pregnancy. Of course, you know the hospital knew. [They had to pee test us and everything, but] I did, and she was fine. It was just because of the sickness, though, like it was more so the sickness than anything. I mean, of course I wish I would have stopped, but I couldn’t- I was too sick.

I: Tell me about that. Why do you wish you could have stopped?

P: Because I mean it did bring like CYF or whatever. They did have CYF come and, you know, check on us and all that. It closed, the case closed, but this time I don't want to go through that.

I: Tell me more about your experience with that.

P: It wasn't so bad like we had to take questions, and they would ask us questions and then what was our plan, they would come, make sure the baby had everything she needed and stuff like that and and maybe my case took maybe like two weeks to close. She would have to come- matter of fact, she would have to come to my house, you know, like they'll come- she came maybe like twice, once a week and then they closed it. Because they said they're not really looking for marijuana like marijuana is not really a big issue but they have this drug.

I: What do you think about CYF becoming involved when like marijuana or tobacco are used during pregnancy?

P: Honestly, I feel like it’s stupid. Not really like that way, but I just feel like I never heard of somebody- a baby getting hurt while smoking weed during pregnancy that's why it's so hard for me to say it’s not bad to smoke, while you're pregnant like I never heard anybody say anything. And if they said that they didn't like weed- they just never smoked weed so they wouldn't know how it was. Never… But I feel like CYF shouldn’t do it because it's not really a big deal, it's just not it's not necessary. This opens doors for- …there's other things going on, besides marijuana.

I: Have you heard of anything that can happen like either to the mother and the baby from using marijuana or tobacco during pregnancy?

P: No.

I: Okay.

P: No actually, I saw [that something happened to the brain.]

I: Tell me more about that.

P: I never really like you know- he said, she said, but I just heard- saw- what happened to the baby's brain or something. I don't know.

I: Where did you hear that? Do you remember?

P: No.

I: Have other people talk to you about marijuana and tobacco use during pregnancy?

P: No.

I: Is it something your doctors have asked you about?

P: Oh yeah they asked me like the same thing you asked me. Do I smoke during my pregnancy or like anything I tell them the truth, like yet this is- I smoke weed but not cigarettes I can't do cigarettes. I can't. It's just nasty to me, other people are different- it's just really nasty.

I: Did you feel comfortable telling them about your tobacco or marijuana use?

P: Yeah I really honestly don't really like care… to say that I smoke weed because it's like it is, what it is, because it helps me like I'm not about to sit here and suffer like if I say it helps me helps me- if I mean I could go get a medical card. I can, and that's something I'm probably going to look forward to maybe after I have this baby or whatever, but everything else nah. I’m not ashamed or anything.

I: yeah did they do anything to help you feel comfortable or anything they did right or do you think it was just all kind of like an internal thing for you?

P: It’s internal.

I: How do you feel like you kind of developed that attitude around it?

P: Because I was getting sick. I really blame it all on that, like it's because I was throwing up and everything like that. morning sickness, it is bad like when I get up in the morning, it feels like my stomach eats itself and then automatically I feel nauseous and then I throw up or if it's too hot or something. I throw up like it's constantly so I really do blame it on that. Because if I was fine during my pregnancy I'm pretty sure that I would have stopped to avoid maybe CYF and everything else that came with it, but I couldn't. I told him that. I couldn't. I said I could have stopped, so I just dealt with whatever came with it, everything was fine like my daughter has everything had everything so she's fine.

I: So I'm curious, why did you tell them or why did you think it is important to tell them about it?

P: Because there was going find I mean there's one find it either way like they pee test me or anything. There’s no point to lie and like if I didn’t tell them it's like a… they could probably find something else for me but nothing else worked for me. Like the C-band or the pills or… it didn;t work for me it was like I was throwing it back up.

I: I want to hear more about your experiences with both marijuana and tobacco, but I'll start with tobacco like when did you first start smoking cigarettes and stuff?

P: 15. For both

I: Tell me about why you started, then?

P: Oh cuz I was a bad kid. So I was just smoking. Like I was smoking weed and stuff like that. So I don't know what made me start, though. I think I just picked up a cigarette. For that… Well, we… I started smoking cause I thought it looked really cool like I ain't gonna lie, so I tried it and it made me laugh, like the very first time I smoked weed I just was laughing, laughing, laughing, and I just liked the feeling ever since then, I never stopped. So that's really it.

I: What do you normally use marijuana for like prior to pregnancy like was it a social thing, or is it something you alone?

P: No, well at first when I started smoking, yeah it was kind of a social thing, but then it would…My moods like I- I got like anxiety, depression and bipolar disorder and stuff like that, like from the doctor, so I was smoking weed that helped my mood stay mellow I should say. To be chill. Because I can get crazy. For real.

I: And how about tobacco? Same question.

P: How did they make me feel?

I: yeah how did it make you feel and kind of what situations did you use it in?

P: Probably for drinking liquor. So, like if I was drinking liquor or something I would smoke cigarettes. I think that's when it started when I started drinking at a young age, but then I would get tired of them. There were times where I would want to smoke cigarettes because probably cigarettes are just nasty but I was smoking a lot. I smoked them a lot so I'm kind of glad I quit. Because they really are nasty. I mean at first it was a social thing that's what made me start really doing it, I was out partying and doing it so maybe a social thing I want to see how it tastes or something.

I: Have you ever tried to quit cigarettes before you [found out you were pregnant?]

P: No.

I: What kind of support did you have in the quitting process?

P: With smoking?

I: Yeah.

P: It was pretty good. Everybody’s proud of me. Pretty good they encouraged me to stop. But it wasn't really hard, though, like it was not hard at all for me to quit smoking cigarettes during my pregnancy.

I: Yeah…

P: I'll say during my pregnancy it wasn't hard, it's just I didn't want it.

I: Since it was something you can use socially initially, did it affect any of your relationships when you quit like the people you have used it with in the past?

P: No.

I: [Have you ever tried to quit marijuana before- now you're thinking about quitting, but have you ever tried to quit before?]

P: Yeah from our past legal situations like I had to so it was still hard and I couldn't- I couldn't. I don't know- I'mma really look more into weed and why people smoke weed and what is in there in the long run. I'm going to look into that now because that's really interesting but I've tried to stop. I really did. When I had to. I was forced, so I had to. I mean that was a rough one when I had to stop smoking for like seven months I did, and that long period of time because of my personal you know situations and it was bad maybe like the first two weeks I would get really sick, though, even when I wasn't pregnant. It will be hard for me to eat, so I will throw up. I couldn't eat anything but then it was like what are you gonna do now? Are you going to keep starving yourself? So I was forced to eat. It was OK, then when I came home, maybe… took me about…Two weeks to start smoking again and I was supposed to because I was on probation but It was kind of hard, it is hard for weed.

I: yeah so it's interesting it's actually been a lot harder for you to quit weed- marijuana than it was for cigarettes.

P: Yeah, cigarettes- honestly I'm gonna keep saying that I think it's because they're really nasty like they really are like being pregnant, maybe not like them. So, like I said, even after I gave birth and they started smoking cigarettes, again I didn't buy packs or anything. Like that I'll probably get like two three from a store and then smoke them. The cigarettes would last like three a day like I would put it out and then smoke it, then the next day smoked the other one like it was something like that.

I: Since it has been so difficult for you to quit marijuana in the past, how are you kind of approaching quitting this time?

P: Because like even when I do smoke it's not really working. It's not working like help (unintelligible) me stuff so I was, I was telling my mom, I want to see if maybe I did, and just throw up in the morning. I have a routine basically- like train my body. Throw up in the morning, get up, try to eat some really fast, get my body full or something like that, because I still do kind of be nauseous now I don't know it's different like last pregnancy different.

I: Can you tell me again, I think you did well with the legal issues with CYF is the reason you’re quitting now- is that right?

P: Can you say that again? I'm so sorry.

I: I think like you said this earlier, I kind of forget, the reason you're quitting marijuana now is because you don't want to deal with CYF involvement in this pregnancy?

P: I mean like- Like if it was the same circumstances, as before, and like I gotta tell them a smoking weed because it is and that thing yeah it's not really because of CYF (says something to someone in the background) it's not really cuz of CYF. I don’t think I'm worried about CYF because I know for sure that they are not going to take my kid. It's just something that I got to work on with my own self like you're just trying to help me.

I: Gotcha.

P: I’m not really worried about CYF.

I: Okay, so then I’m wondering like, why is it important to you for yourself to quit marijuana now?

P: kind of like personal things, chemical imbalances and things like that. And even being sick like when I was smoking cigarettes before I found out, I was nauseous and I wonder why- I just started smoking cigarettes again, so I was nauseous. And like even when I smoke weed it'll throw me off a little bit, but then I hop- my stomach will just hop right back in and be hurting so this time is really more about the same thing, like cigarettes making me nauseous the chemical imbalance.

I: In the past, when you've used marijuana and tobacco, at the same time, in your life had you ever used them together?

P: What you mean?

I: Like on the same day, or on the same night or something like back to back?

P: Yeah.

I: Tell me more about that.

P: The cigarettes will be a booster for the high. Like if I smoke a blunt I'll smoke a cigarette right after like right after. Maybe just boosted. I don't know but that's what I've heard that it boosted the high. Did it boost mine? No, I just liked the taste.

I: So did you notice any difference in the way you felt at all when you use them both versus just marijuana?

P: What do you mean?

I: Like you said, some people say [cigarettes] boost the high, but you weren't really sure, did you notice the difference?

P: Maybe so. Maybe I did. Maybe I did. I would be high and maybe fall asleep. Maybe so. I probably did. I never really noticed.

I: yeah. So you have used like marijuana during your pregnancy, has anything changed, while you've been pregnant? [How you use it or when you use it? Has the pattern of use changed at all?]

P: No, it's all the same.

I: I want to next move a little bit more into more questions about doctors and your experience. What have other people if anything like told you about talking to your doctor about using marijuana and tobacco during pregnancy?

P: Nobody really tells me anything. Like it my doctor. I’ll tell the doctor anything I want.

I: I mean like did you talk to any friends or your parents or anything like that about whether you should or shouldn't tell them about your use?

P: No, nobody [told me not to tell them.]

I: Okay. And what were some of the we've covered this a little bit already, but like what were some of the reasons that you did want to tell them about your marijuana use or your past tobacco use?

P: Because they ask yeah, I gotta pee in the cup what am I gonna say? Like I'm not smoking? I was just telling them I smoke weed only because I don't care that I smoke weed like I feel like if I was to ever go to a doctor or anything or get in trouble about we like i'll be able to be like Okay, this is why I smoke weed and I'll be okay like I can like medically prove that I could get medical marijuana if I wanted to like so that's why I’m really not worried.

I: And how did they respond when you told them about it?

P: Like a doctor, like ‘oh okay.’ [And you know like] you know it's not good for the baby. You might want to stop or I’ll tell CYF or never really tell me how it'll affect the baby no.

I: yeah I was curious about that, when you said that if they tell you like, specifically, how can it be bad for the baby that sounds like no.

P: not really not that i've heard or paid attention to.

I: Is that something you'd want to know, like if they did-

P: I'm pretty curious. I’m pretty curious.

I: yeah have you thought about asking that or like looking that up somewhere else?

P: Yeah, I’mma ask it. probably at my next appointment.

I: Did they give you any other information, besides in the conversation with pamphlets or brochures about that kind of stuff?

P: And they gave me books and stuff. I never [read] them.

I: Where do you think, like other young pregnant women like yourself, can get information about marijuana-tobacco use during pregnancy, if they do have questions?

P: Probably from their doctor or things like this… or from the internet or different people. Asking around.

I: Besides, some of the effects on the baby which we just talked about is there anything else you would want to know about marijuana and tobacco during pregnancy, either, that you have asked her that you haven't been able to find out?

P: No, not really besides what it does.

I: Do you think there's a difference, or like what is the difference between talking to me as a researcher versus talking to your doctor about marijuana and tobacco?

P: No. Not at all, I really don't. I think I feel like it's the same type of thing like I'm on record[ing] here. People can see me here. So it's like there's no difference to me, besides talking to a regular doctor too.

I: yeah would you say, like all the same things, or do you feel like maybe you're more willing to be honest with your doctor or you’re more willing to be honest with me like anything different all in terms of what you would share?

P: Well, I would share. It sounds like I'm sharing more here because, like they don't really ask me that, like those types of questions so not a little bit of both it's not really… in between.

I: yeah all right so what other things do you think would help young pregnant women get more information about these topics and what's the best way to get it to them?

P: Things like this and more So for me, talking to other people. Like I'll talk to other people and get other people's opinions.

I: yeah tell me more like who, in your life, would you talk to?

P: My family, my grandma and my mom.

I: yeah.

P: People like that, maybe my friends that had kids and not the ones that don’t because they don't really know. So my friends that got kids and things like that.

I: So outside of your personal life like what else would help or outside of like the resources in their personal life or other kinds of programs or things could help young pregnant women get information about this?

P: I'm not sure. Not really sure.

I: Alright.

P: They would have to just look around you know- things just don't come to you so like if you're looking for something you have to- you know me honestly I go to google first about like if I need to find some out real quick the yeah I'm gonna go on Google. Otherwise I don't really know.

I: In terms of Google like is there a lot about or have you googled stuff about like marijuana and tobacco during pregnancy?

P: Say that again.

I: Have you researched online/Googled things about marijuana and pregnancy and tobacco pregnancy before?

P: No. not yet not yet.

I: Is that something you plan to do?

P: yeah. Maybe a little bit after this actually.

I: Just a few questions left, so the next one is in an ideal world, what can healthcare providers do to help young women feel more comfortable talking to them about marijuana and tobacco use? I know, for you it's not a big issue, kind of like internally having that confidence to do it, but for someone that doesn't like what can doctors do for you to help them feel more comfortable sharing?

P: They just- I feel like they just got to be trustworthy. Some people don’t want to open up to a doctor, because they probably think it’s going to cause legal problems or whatever, but I feel like weed and cigarettes, especially cigarettes if they're legal in the store, I feel like that's not nothing to have legal problems about. So maybe they got to be trustworthy and it depends- you're right about that, because some people probably don't open up to their doctor, or other people sp I really don’t know. That's just something they're gonna have to work on.

I: Yeah okay. How can doctors prove or show that they are trustworthy?

P: I don’t know.

I: Okay. Is there anything that your doctors have done to show you that they are trustworthy?

P: Me, honestly, it’s a female doctor. Like I rather, of course, it's a women's hospital, but I rather have a female doctor talking about another female perspective- that’s my opinion.

I: yeah that's important thanks for thanks for sharing that. That's great. So a few more just random questions I have. I'm wondering if, like the covid 19 pandemic has impacted or marijuana or tobacco use at all?

P: No.

I: Do you feel like it's impacted your pregnancy at all?

P: Nah.

I: Is there anything I didn't ask about today that you would want to share on this topic for the recording?

P: No, that really covers everything.

I: Okay, all right I'm gonna go ahead and turn off the recording here.
