# Supplementary material for: Experiences of postpartum mental health sequelae among black and biracial women during the COVID-19 pandemic
Source: BMC Pregnancy Childbirth. 2023 Sep 4;23:636. doi: 10.1186/s12884-023-05929-3 (PMC10478375; doi:10.1186/s12884-023-05929-3)
Supplement: Supplementary file 7 — Supplementary Material 7 [file 12884_2023_5929_MOESM7_ESM.docx]

**Supplemental File 1.7 Interview Transcript with Participant 5196**

I: Is it okay, if I utilize the term bisexual moving forward when I asked questions?

P: Yes.

I: Yes, okay cool and let me see I'll also use LGBTQ, just to have like umbrella terms, but like to get that out of the way, so I know that I am you know, using what people prefer. So, first we just start kind of like we warm up a little bit so, can you tell me, you know what it was like for you to be pregnant? Have you had another kid since 2020?

P: No. I just had three. [name] was my last baby.

I: Okay What was it like with him?

P: It was horrible. I threw up every day. All day and woke up at three o'clock in the morning to throw up. The game medicine at the clinic and it didn’t help. They gave me sleeping pills and that didn’t help.

I: yeah I really feel for you that's really [awful]. Because it's nine, ten months of being really sick. Was that how all your pregnancies were? Were they kind of different each time?

P: They were different. With my older son, it was good. I loved being pregnant with my older son. [With my daughter that pregnancy was good] despite the issues with my mom and stuff like that, despite that type of stuff I was good. I had her and then I had postpartum depression, but even carrying her I liked it. It was just [name] he gave me the hardest time.

I: [Did you have any postpartum with your youngest?]

P: No. I didn’t have any postpartum which made me shocked, after all that throwing up and stuff like that. I thought I would have but I was fine.

I: [I wonder if it had to do with the fact that you were sick the whole time. My best friend, her pregnancy was like that and once she was done she wasn’t sad that she wasn’t going to be sick or anything anymore.]

P: Yup that’s the main reason because you can eat, you don’t have to worry about throwing nothing up- because when I came home I ordered me a pizza and I just ate the whole thing by myself. He was sitting in the baby swing just swinging and I was eating pizza.

I: What was your experience like, with your doctors and stuff?

P: I love all of them. I loved all the OBs and the doctors in the clinic. I even loved the people that took my- I mean you come in, you do you're sitting there and they call you for your appointment and I even love them, they were nice to me. Everybody was nice in there.

I: What is your favorite thing that your doctors did that made you feel you know, supported or cared for?

P: I love the Mychart thing they did, and I also loved the resources they gave me. Magee gives you a lot of resources that can really help you out as far as parenting classes- different- all types of things, I still have the paper with all different resources on it from when I was pregnant with my oldest son I just kept it.

I: Did you use any of those?

P: Yes.

I: [Were any of them particularly related to your sexual orientation or anything?]

P: no.

I: Is that something that you'd like to see or something you don’t really care about or need?

P: It was fine with me.

I: Do you have any experiences that you thought could have been better or have gone differently?

P: I will say my births. I had three C-sections, with my youngest I think it was a student and I don't like students working on me. It’s like you're still learning and some type of way and I don't want to be the guinea pig. And the lady was holding my hand and they put the needle in my back, but they hit a nerve.

And it shot down to the tip of my toe and she was like what's wrong and I'm like tell them to take it out. (unintelligible) please take that out so and then even with my older son I had an incident like after I had him. We was upstairs and my friends came to visit us and I was talking to my friends and I was letting the nurse do her thing for the baby you know on (unknown) with her like I know that’s my newborn but I didn’t want to be on her back like this is her job. I’mma just let her do her job. She tapped his head off the beds that they sleep in. She tapped his head and I heard the tap and thought it was her hand until my oldest started crying and she never said nothing. My friends told me as she was leaving that she just hit the baby's head. Like that’s pretty rude. You could have said sorry or you could have been like I apologize. Accidents happen, that’s okay- you deal with babies all day long but how your body's tired and you just want to go home, so I just didn't like that she didn't say nothing that she just walked out the room.

I: The communication wasn’t (unintelligible)

P: Yes, yes, especially with that situation. I didn't like her, communication and then, when my daughter… it was good, no problems with my daughter- no, it was just my two boys.

I: So the one you had a student and it was- (cut off)

P: Even with my oldest son, students were there, but my mom was there. This time I gave birth to my younger son I was there by myself. (something unintelligible afterwards due to background noise)

I: Do you feel like they are not experienced enough, they don’t listen well or- what happened?

P: I think that I think that is one of the reasons I think like because their students and you know I’m still young. Some of them, I think, like okay well you know she doesn't know she's talking about she’s just young and pregnant. I know a lot and I know (unknown word) isn’t supposed to be like that. I know enough. I that (unknown) isn’t supposed to be here like that.

I: yeah, you’ve had C sections before.

P: Yes, yeah I pretty much knew that that was not the way it's supposed to feel.

I: If any would know, it’s someone who had done it before twice. Did anyone during the time of your healthcare ask you about your sexual orientation or the gender of your partner?

P: I think that there's a few times on my paper and that sort of stuff, I believe, like as far as my sexuality and things like that. So yes, I believe so.

I: Are you comfortable sharing that with your doctors?

P: Yes.

I: What is the best way for them to start that conversation or bring it up to you?

P: I would say start off nicely. But be nicely I even if you're not (unintelligible) their preference of who they like they love you know just still be nice like they still are human you know, no matter what type of sex they with other people- everybody in there, but we still got a whole if he's saying we all got loads of blood, bones and a skeleton. I feel like we’re all humans, but some people nowadays it's a lot of judgmental things going on, so I feel like that is a problem.

I: yeah. yeah I think you're right. It is hard to have a conversation with someone that comes out feeling like you know from the jump, making you feel judged or has ideas maybe. what was it like for you to be pregnant and identify as bisexual?

P: It was regular to me, because I haven't been with a girl in some years so I've been dealing with the same person for four or five years so to me it was you know regular I'll be you know I don't have a problem, like you know I’m bisexual so I do like girls, but I, you know, I was dealing with the same person so it was normal to me.

I: yeah do you think that, like you know being pregnant changed how you thought about your sexuality?

P: No.

I: Is there anything else that like you think is important to share regarding being bisexual being LGBTQ plus and receiving like medical care?

P: No not really.

I: Do you think it's different at all for like medical healthcare?

P: Maybe in some ways it probably is, I want to know, like to say how, but I feel like in a certain way it probably is I (unintelligible) probably experienced something different, but pretty much.

I: The next few sections are kind of all about marijuana and tobacco use.

P: Okay.

I: I like to say, like obviously we'll keep everything confidential we don't share with doctors or anybody else. So, can you just tell me your memory of the first time that you used marijuana?

P: Yes, I was 18.

I: Were you alone, with someone, what was it like?

P: At the time I was with my kids’ dad. It was just us two.

I: Were you scared? What did it feel like?

P: Before we even smoked it, I was nervous, you know- I never smoked before then, but like I was dealing with a lot like my sister just got killed. So I’m just like Okay I'm not a drinker and I don't smoke cigarettes or blacks or anything like that so I'm like don't do that either so I'm like- okay well, let me just have a few puffs but how can I just felt mellow to me. I felt real mellow like I felt lifted off my feet, for the first time, you know all the stress of my sister getting killed- all of that went away. It took all of my stress and pain away just for that moment and I felt good.

I: Powerful, that's powerful. Like grief is so hard and for it to be able to kind of give you a break even if just for a moment. What's your relationship with marijuana now like how's that changed?

P: No, no, no smoking is expensive because I had kids to maintain and a house to maintain. I have rent and bills and stuff like that so I don’t smoke it too much now it's only if I had the money to buy it. if I don't have the money to buy it. I need to pay my bills so that I don't worry about it, I know it will still be there.

I: Does it still give you any kind of relief or anything?

P: Yes.

I: So did you smoke at all during your pregnancy?

P: With [oldest] I smoked it in the beginning, and then towards the end I stopped smoking my daughter… I think I smoked it with my daughter, and my youngest son, no. I was throwing everything back up, so I couldn't even get a chance to smoke.

I: So didn't even help with the nausea or anything like that?

P: Nope and I was shocked, because a lot of people were recommending it, especially people that had babies and stuff like that. And it was like well you don’t have to smoke a lot and I'm like no, this is not working, and then I’m just gonna throw it back up, so I’m okay I’ll just throw up.

I: Have you ever consciously decided to stop smoking for a while or was it something that you let go of?

P: I pretty much just let go of. Like some people need like marijuana in their system, every day, they have it. Me, I’m okay without it. I feel like sometimes I will be better without it.

I: yeah Have you ever had a time in your life, where you were smoking, it almost every day or has that not been you?

P: Yes, in the beginning when I first started smoking, yes. Probably for like the first three months I was probably smoking it a lot.

I: Did it stop working as well as it had been or what shifted for you?

P: I just started moving and stuff like that. I didn’t have the money for it so it wasn’t about it going bad for me. I just didn’t have the money for it so I wasn’t too worried about it.

I: What is it like you know when you are able to buy it and use it- what does it do for you?

P: Maybe three babies, so you know when everybody is sleeping so I’m in here cleaning and it gives me a piece of mind. It’s just a whole different piece of mind.

I: Can you explain that more?

P: Oh yes. I'm mellow and real chill. Sometimes I even go to sleep faster that way because there'd be times where I'm up thinking and I'm overthinking and I could be up to three, four in the morning just sitting there overthinking. It is sometimes that bothers me so I can smoke then going to sleep. I’ll watch a TV show and I'll fall asleep, but without it sometimes I can overthink a lot and I don’t like that. I don’t like to overthink too much but… sometimes I can.

I: yeah so it kind of just turns the radio down a little bit in your brain?

P: Yes, a whole bunch literally cause I overthink so much throughout the whole day.

I: yeah it's nice to have a little off button sometimes.

P: Yes.

I: yeah. Is there anything about using it that you like that you don't like anything about the experience that’s negative for you?

P: Yes, it's costly, it can be costly sometimes if you don't have the money for it, you know it's costly.

I: So do you think there's anything about like identifying as bisexual that kind of affects your like how you use marijuana like you know some times there's like discrimination against LGBT Q plus people anything like that?

P: No not me personally because everybody smokes marijuana today well majority of majority of the world smokes marijuana. I mean young older people, older than my grandma that be smoking marijuana yes i'm like okay your.

I: your friends would you like to do with your friends and stuff like a social thing?

P: yeah sometimes when my kids are at daycare and my friend’s kids are at school or something we will smoke when we’re kid free.

I: yeah so when you have a little break from kids you time?

P: Yes.

I: This is the person that you're with anymore, but does the person that you were with for a long time, do they smoke?

P: Yes. I think he’s been smoking his whole life.

I: So he's one of the people you were talking about earlier that's kind of like maybe means that every day?

P: Yeah, he’s one of those types of people that need it everyday I think.

I: Do you have anything that makes you crave it or is it like a trigger for you to realize Oh, I really want to smoke right now?

P: Sometimes I'll say sometimes- every kid I think everybody's kid has a time in a day, where they just you know they’re real hyped and things like that and not listening to you, so this could be a time I’d be smoking. Y’all drive me crazy up the wall about the street.

I: Just having to deal with the constant kind of kids?

P: Yes.

I: Are they all under five right now?

P: yeah.

I: I bet you don’t sit down.

P: I’m shocked I’m sitting right now.

I: This is a special time, thank you for giving it to me. What do you think would help, like you know other young women that are pregnant, and maybe want to quit smoking like what would you tell them right now?

P: start spending your money wisely or phase, you need or your baby may need first or even like paying your bills first some people don't get money and they'll go directly to buy marijuana. No, I think it is best to handle what you need to handle for as your baby, your household, even yourself and then whatever you have don’t have to spend on bills or payments then you know treat yourself, but some people put it first and that's where a lot of people put out here and why they put marijuana first instead of last, but it’s still gone be there.

I: yeah that's a really good way of putting it i've never heard someone kind of explain marijuana like that. So do you think that before we move on to the tobacco questions which I know you don't even need so probably pretty short- So is there anything that you think like researchers should know about you know being Bisexual and smoking marijuana?

P: No, because all different types of people smoke marijuana straight, you know older, younger, majority of everybody smokes marijuana I don't think it really has anything to do with your sexuality at all. It’s your choice, if you want to smoke it or not.

I: Okay, thank you, this one, I feel like it's gonna be out, you have not used like cigarettes or like little cigars or anything like that?

P: no.

I: Why not?

P: If you look up cigarettes and marijuana, there's a lot of difference like I don't like I don't know too many people you know got holes in their throat due to smoking marijuana but, like the commercial is due to cigarettes, you see them with the holes throats and the machine through their throats and then a lot of other problems happen and then not only that- I watched my mom smoke cigarettes as a little girl.

And I mean she's a chain smoker so she's constantly smoking cigarettes. I can't do the smell. It’s like way different from marijuana like when you don’t- when you don’t smoke marijuana- marijuana stinks to you until you start smoking it. Cigarettes- I can’t, I don’t know if it's the smell and it has too many negatives to it.

I: Too many- like the health stuff you were talking about?

P: Yes, yes.

I: Using that you know you would have your family use like your mommy's and that made you not want to smoke, or sometimes it goes the whole other way. So let me think about what else I want to… so if you don't smoke you never want it, so you never even tried it?

P: No.

I: Okay, so what, how do you deal with stress, then?

P: Sometimes I go to sleep.

P: Sometimes I will do something that's taking my mind off that situation, especially if i'm thinking about it too long i'll go ahead and wash clothes or occupy my mind. I'll put the crayons out for the kids that were taking my mind off for a minute, so my mind has a minute to settle down. And then I'll go back to that situation, if not the next day. Like a cigarette… cigarettes are wash harsher than marijuana. People smoke while pregnant that's really not supposed to do, like nice that's way different than marijuana to me.

I: What would you say would help women stop smoking or not smoke cigarettes during pregnancy?

P: I would say more water. I would say that every time you want to smoke a cigarette drink a bottle of water.

I: Hydrate yourself.

P: yeah instead of like okay I’m going to light this and inhale it, and especially when you're pregnant, because even with marijiuana you’re smoking for two.

I: What do you think about okay so that's kind of what you do when you're stressed out. What about we're just going to go through like the big emotions basically like what about when you're feeling really depressed or down?

P: I’ll just cry. Yeah, I’ll cry before I turn to a cigarette.

I: You just feel it?

P: yeah.

I: What about when you're angry?

P: Oh, I sit down too. I’ll find something in my house to occupy my mind because I always have something to do- something always has to be done here. So I’ll just do that and do something that I put off for a couple of days and then I finally do it because this is not the time and I am not in the mood.

I: So stay busy?

P: Yes.

I: What do you wish all doctors, nurses and students in the healthcare industry knew about pregnant bisexual women?

P: I don't know. That's a good question because I don't know I never got treated differently or anything, so I will just say still treat them like humans, despite what they like. Even if you're not comfortable with that you know that's not what you're working for you're working to you know get them through their pregnancies, help them give birth and stuff like that you're not here to judge don't worry about who they’re laying down with that night so… To walk into the office and talk to our doctors for them to say ‘are you bisexual, are you gay?’ Like that's not it that's not my business I’m just here for a check up.

I: How should it be asked if they were going to ask you?

P: I think they should ask it kindly- not like you know, some people might say something, and it comes off as short or you can hear the tone in their voice is like they're disgusted or something. I don't think you should do that. Because it's about actions and facial expression that's what I pay attention to, so you might come out, you might even say nice, but I can see the facial expression interface that you're not being too nice about it, so I think those two.

I: I think that's a really good point because a lot of people focus on what's being said but there's so many subscripts within our body language and what you're saying about like the tone- you can tell a whole lot.

P: yeah.

I: Okay So what about what do you want bisexual women to know about pregnancy or LGBT Q plus people to know about pregnancy?

P: It's still the same. It's the same if you were married or like if you were straight, or if you were going it alone. It's still the same way. It’s still the same way, so I don't think there's no difference. You're still a woman, you're still carrying a baby, you still have to go through vaginally or a C section way you know you still stay in hospital for two to three days, just like every other woman would so.

I: Thank you, so the last well there's like a little bit after but the last two big ones are like you know what do you wish all bisexual women and girls- so like think about you younger- knew about marijuana use?

P: It's expensive sometimes depending on your situation, it can cost you a lot…

I: Like money wise or living?

P: Yes, yeah it can cost you like, for his money wise especially if like I said, especially if you don't have it- don't go get it if you don't have it. Because you don't have to pay Larry back a $20 bill, because you asked to go get you some marijuana now that's not no- if you don't have it don't go get it. Don’t go searching for it either, it never works out.

I: What I’m hearing you say is to focus on your priorities.

P: yeah.

I: yeah cuz money does kind of show our priorities, a lot of times. Okay, now, what about you younger LGBT Q bisexual women, what do you wish that you know we knew about tobacco use?

P: So it's not it's not the same as marijuana, but it has side effects like marijuana does, like, I said to me cigarettes are just more harsh especially when you're carrying a baby you're inhaling and all that nicotine like that, and you could smell cigarettes like me, personally, I could smell the cigarette- I don't like to smoke alone, so I can't like Oh, can I get a cigarette and I just seen a couple people like while they were pregnant, you know outside smoking cigarettes and I'm like oh poor baby. That's the first thing I thought, oh my poor baby and usually people, women, who smoke cigarettes during the pregnancy their babies usually have the problems in the ICUs, and all that type of stuff they usually have that type of problems, you know given birth babies come out really, really small.

I: So more complications?

P: Yes.

I: Yes, yeah smaller baby stuff like that can happen from using tobacco. Did your doctors talk to you about like marijuana tobacco, when you were with any of your babies?

P: With my first and my second one, yes, they asked me if I was smoking, I said yes.

I: You think they handle that okay? like how was that for you?

P: yeah good actually because it made me go back home to do some research as well.

I: Okay, something like talked about what might happen and stuff or was it just like?

P: Yes, it's clear what can happen during pregnancies even if everybody's different. They let me know everybody's different, but these are side effects for smoking marijuana. In that they don't have too many effects if you were smoking a cigarette or if you’re vaping while you're pregnant, I say yeah.

I: Okay, so it kind of helped you become curious and open to learning more about it?

P: Yes.

I: So that's the end of the interview. I do like to ask you if you want to, if you have any feedback for me on how I can be better at asking things differently in a more clear way, so I like to ask that before we officially end.

P: You did a good job. You did a wonderful job to me.

I: Thank you. Thank you for helping me end the week on a nice note, you know?

P: Yeah, you're welcome.

I: yeah This is great i'm going to put the $50 on your card Now you can always reach out to me like if it acts funky or something's wrong with it, or you know we're also doing interviews- I don't know if you've been reached out to yet, but they're also doing interviews about like racial discrimination and covid 19 in the health care system.

P: Okay.

I: I think that's another $50 payment so that's something you're interested in I can let my coworker know that but no pressure, because I know you just you know I don't want to interview you too much.

P: yeah but that's something I'm interested in.

I: Okay cool I think it's a cool interview, I think it might be shorter, but yeah. Do you have any questions for me?

P: Nope.

I: No? okay well I'm gonna pay this $50. I hope you have a great weekend. I hope your babies are healthy and happy and I hope they give you some peace this weekend too.

P: We’re going to my grandma’s house for Memorial Day Sunday. Thank you, miss [interviewer name].

I: Thank you bye.

P: bye.
