# Supplementary material for: Experiences of postpartum mental health sequelae among black and biracial women during the COVID-19 pandemic
Source: BMC Pregnancy Childbirth. 2023 Sep 4;23:636. doi: 10.1186/s12884-023-05929-3 (PMC10478375; doi:10.1186/s12884-023-05929-3)
Supplement: Supplementary file 8 — Supplementary Material 8 [file 12884_2023_5929_MOESM8_ESM.docx]

**Supplemental File 1.4 Interview Transcript with Participant 5221**

WEBVTT

Q1 5221

Interview Transcript

I: Okay cool. So this interview is kind of- the idea is for you to talk more than me, so it's really about your experiences and your thoughts. I’m going to hide myself from myself. Um, so how do you think your pregnancy is going so far?

P: So far, it's a rollercoaster but I mean, for the most part yeah. Health wise it's going really good.

I: That's good. What do you mean a roller coaster then?

P: It's very stressful. Whether you have support or not, it is very stressful, but the more, I guess the more support that you have the better it is because then you're not worrying about okay, is this person gonna answer me if I say this, or is this person going to acknowledge that I’m talking to them but you'll always have them extra people to fall back on.

I: yeah what kind of like, what are your main stressors do you think? This time. This pregnancy.

P: My main thing is how my son's gonna act with this one and I don't want twins.

I: Are you having twins?

P: I don't know. Everyone keeps saying I am so I’m really hoping no.

I: How do they know?

P: Because I’m so much bigger with this one that I was with my first.

I: Oh

P: I mean like. So much bigger.

I: How far along are you?

P: I am 11 weeks and five days.

I: Okay, who's telling you that?

P: Literally everybody. Everybody to see me when I was pregnant with my first they- they're like you, you’re gonna have twins and you're having twins. Not only that I’m the next person in line through my family to have twins so I’m not ready for it.

I: Yeah, hopefully the doctor can tell you one way or the other, so at least you can like prepare.

P: Right

I: Whatever comes.

P: Right well in the sonogram that I had, they said that there was- there was a possibility that there was two because of the way that the sonogram looked and I- I almost broke down and cried. I was like please don't tell me that.

I: Yeah.

P: So, but I can't wait for my next one I’m excited.

I: How old is your first now? You told me before, but I forgot.

P: he's two.

I: Okay. That’ll be fun for him, maybe.

P: Right.

I: Okay, so I am wondering specifically about thoughts that you have about marijuana use. Just to jump into the meat of the interview.

P: Me, personally I do not like it. I used to- I mean I’m not gonna lie I used to smoke it before I got pregnant, but after I got pregnant It didn't help. Everyone was like “Oh it'll help with the nausea. You'll feel better just try it”, I was like okay I’ll try it, but I didn't like it. It didn't help me at all. It just made me more hungry, more stressful, and more tired and I wasn't, I wasn't with it, so I stopped it. And with this one, I actually haven't smoked since. It's almost been four years.

I: You tried that with your first and it didn’t work and then this time you didn't. You knew not to even-

P: I won't even touch it anymore.

I: Who told you that, like who was telling you that it would help and stuff?

P: A couple of my friends. Um, the one she was pregnant and before I got pregnant and she was doing it. And I heard, I also read that it is good for the child's brain development so I’m like you know what I-I didn't believe it. Why, I don't know. I just I didn't think that it was true, so I let it go. But she said that she she smoked throughout her pregnancy and it helped her with the nausea, so I figured okay I’m gonna give it a try and see what happens. I did it for three days, and it was just making everything worse, so I stopped and I figured something else out so.

I: What ended up working?

P: Sea bands, the little pressure point bands you put on your wrist.

I: Oh, really? Yeah did a doctor help you with that or did you kind of find all this out on your own?

P: Um well, I have a friend whose like- she’s had two kids herself but she's like real I can't say a freak, but she's a freak when it comes to all natural everything. And she told me, instead of taking pills, and all this other stuff she told me to get the sea bands because her whole family used them and I guess that's what her midwife told her to use because she wouldn't take pills, so she told me to try them. I did. Best thing I ever used.

I: That's awesome I’m glad that worked I have friends that are like the same kind of weird like.

P: Yeah.

I: Like kinda like hippies but like extreme.

P: Right

I: Yeah I’m glad that worked for you. Um where did you- so you, you said that like you read that it was good for brain development, but you didn't believe it like, how do you kind of figure out that information?

P: Well, when my friend, told me that it was good for brain development. At first, I was asking doctors and they were like “Yeah in a way, it is good, but it's not like- you're not supposed to do like every day use for it” and I was like okay. And then, when I read it online, it was like oh yeah the brain develops two times faster and I’m like okay. But it didn't make sense, why? What chemicals in marijuana help a baby's brain develop, but it messes up the adult’s brain. Like it didn't add up, so I- I- I can't. I just I need to see more proof that it helps a baby's brain. What in it helps a baby's brain but what's messing up the adult’s right?

I: Mhm yeah so you could. It wasn't adding up to you.

P: Yeah.

I: Okay, so if this is true, then why is this also true?

P: Right

I: Did you feel comfortable talking to your doctor about that?

P: At first, no, because I thought I was going to be judged and I thought that they were going to take my child off me because CYF has been in and out of my life since I was pregnant, because everyone kept saying that I’m using and abusing drugs and alcohol. I had CYF in my life, the whole throughout my whole pregnancy, with my first child. I got drug tested every month, and it- it was a wreck. I was literally a wreck. So, for me to bring it up to the doctor and be like listen I did this once CYF is already, you know, dragging me down because everyone's making phone calls saying that I’m doing this. I was worried that they were going to actually take my child off me. Even when I was in the hospital literally the day that I had him, they took him away from me, had me drug tested like throughout the blood, urine, everything that you could think of. They had me drug tested. They tested my alcohol levels. And then they looked at me and said we're sorry for the inconvenience, we had to take your child off you because CYF is called so caseworker came in I said, “You know what this is the seventh time already, and I haven't even gotten to see my child yet.” So it was, it was upsetting so I since then I will not touch anything. I can’t

I: Yeah how did that feel like to have to be, you know, to be treated like that?

P: I felt like I was being singled out, um, because there's, there's so many people that give birth in the hospital that are on so many things, but they get to take their baby home with no, no questions asked at all. But me everything was healthy. I was healthy. I was doing everything I needed to do. I followed a diet that I asked my doctor for. I wrote down everything that I possibly needed. I had a journal, of all the stuff that I was doing every day, all the pills I was taking that they were giving me, how many times a day I was doing I was eating, what I was eating, how many calories I was eating. And I felt like I was being singled out and they were thinking that I was a joke, so it really hurt like for me not to be able to see my child over false accusations that I can't even report. It really hurt like that was that was the all-time low for me, and I- I didn't know what to do. I called everybody freaking out, crying and I was calling everybody. I called the cops myself, and I said listen I don't know who's making these reports, but you need to find them, because this is messed up. I don't get to see my child because of these accusations. They drug tested me. They did everything. Everything came back clear. Why don't I have my child? So they did find out who called and they did press charges, but he didn't go to jail. Which he was the he kept thinking he was the dad, but I got a DNA test he wasn't even the dad and he was mad.

I: Yeah and it sounds like you were just doing so much to make sure that you and the baby were healthy. It sounds like nobody was like believing you.

P: Right.

I: That's frustrating.

P: It is.

I: What made you kind of decide that it was worth telling your doctor then after, after all that had happened?

P: Because I didn't want- it was our- one, my business was already out there and I didn't want to be looked at differently so I basically I said to him straight up I said “listen, for the first three, four days of my pregnancy, when I found out, I was pregnant when I had the nausea, I would smoke a couple, you know couple hits off of a blunt, not a bowl, a blunt, here and there to try to see if that would help with my nausea” and she was like it's okay, you know you could talk freely here. I said well, how do I know that because I’ve had CYF called on me. She goes we're not gonna- we're not going to report that. One, your test came back negative. We have tested you every time that you come in. I didn't know that. But they tested me every time I went in. I’m actually glad that they did and I’m glad that they didn't tell me, because then I would- I would feel singled out again. But, um so after they told me that they were testing me, I was like okay, cool whatever. And they said that listen it- it helps a little bit. It helps the baby's brain develop, little by little. They said that there are certain parts of the brain that don't develop until after they're born fully. But the marijuana helps a little bit to, um, enhance it. So I was like alright, but I didn't want to try it because I just couldn't. But it got to the point where they told me that I was okay to do it, but I wasn't- my personally I wasn't going to because I can't. I don't know. I just can't stomach it.

I: was that- Could you handle it before you got pregnant and, and it changed or just?

P: Before I got pregnant I’m not gonna lie before I got pregnant, yes, I used to smoke it all the time. But when I got pregnant, that was it. I couldn't do it. I stopped and I told everybody so don't even come around with it, I can't do it, and then I got my nau- like I said, my nausea got really bad, so I tried it and it didn't work, so I completely broke off of it.

I: Did you try to quit was that, like you're like “Okay, I’m pregnant now, I’m going to quit” or was it a situation where like my body doesn't like this anymore like? How did you-

P: I was already in the process of trying to quit because I, I felt like it was ruining my life. And before I got pregnant I was technically, in a way, homeless. But when I found out that I was pregnant I called everyone, like all my family, I said listen I need help. And everyone said, as long as you get clean, you can come home. Got clean straight right from the door. I told I told my adoptive mom. I told bird mom. I told my uncles, everybody and they all said the same thing. If you get clean, we will come get you. And since then never touched it.

I: Four years ago.

P: Yeah.

I: Congratulations. That's hard to do.

P: It is very hard. But I- I did it.

I: What do you mean- okay, so I have a few questions because I just feel like you're, you're, what you're telling me is like really important. You said it was you, kind of felt like it was ruining your life like what was your experiences with it?

P: I had just been, every day, I was spending money that I could have used for something else. So, before I got pregnant, um, I would take, well, wouldn't take money, but I would work for my money, whether I had to clean a family friend’s house or do whatever. I had to work for my money, and then I ended up giving all of that money up. And I still had nowhere to go and I felt like I could have used that money. Looking at it now, I could’ve used that money for something else. Definitely could’ve use it for something else. So, after a while like I said I was so tired of spending the money. I was tired of you know, earning the money but not being able to show for it and I gave up.

I: When you decided to quit did the like experience of like getting high change or do you- you just say like I don't care what this feels like I’m not going to do it anymore?

P: I, at first, I was like panicking because the experience of the high, it was- it was- it was fun like it relaxed me, calmed me down every day. So the days that I was like really stressed out, I had that to fall back on to calm down and relax. But, I didn't want to harm myself anymore like not harm myself, but I didn't want to hurt my life. Like I was tired of ruining my life because of it, and I couldn’t- I wasn't allowed to see my family, my, my friends, they started to get try to get me on smoking it more and more and more and I didn't want that. So I, I had to stop. I wanted to see my family. I wanted to be able to hang out with my uncle and my cousins and everybody else. I wanted to be able to be there with the family and be able to go to Christmases and Thanksgiving and everything else, because I wasn't allowed to because, I was always high. I think it was more of a family thing. I wanted to be with my family and I didn't want to be by myself anymore. So I think that's like the main reason I quit and the second one was oh crap, I’m pregnant.

I: Yeah it was like just enough, a little incentive more.

P: Yeah.

I: That's so interesting. Uh, how did you like what was your preferred way to get high like smoking, eating it, bongs, bowls, you know?

P: Yeah. Mostly smoking it. I would smoke it out of the blunt and I’m just one of them people that didn't care. I’d walk around smoking it and if a cop stopped me I’d tell them like “Listen, I’m going through a lot. You want to throw it, go ahead and throw it, but you throw it I’m picking it back up.” And I did. It got to the point where the one officer he watched me pick it up and I burnt the end and I put it right back in my mouth. I killed all them germs and I put it right back of my mouth and he was like “yo, are you serious?” I said “I am” and he- he looked right at me and he was like listen. He ended up taking me to the police station and tried to get somebody, one of my family members to come get me and they all told him, you might as release her because we're not coming to get her. And that's- it kind of hurt me. I felt, I felt like I ruined their life too. And I I felt like I ruined their last name. So, but he did release me. He put me in a shelter, so I wasn't on the streets anymore, and he he helped me find some side jobs, like, um, cleaning and I’d go over and I clean his house for him. And I help the other officers and I volunteer at the fire hall so. Like I’d clean the fire trucks and everything else so. Yeah he said, as long as I didn't smoke and I turned my life around they'd help me so.

173

00:19:28.710 --> 00:19:42.660

I: It was you quit like, four years ago, so that was like it became available in medical places like in 2018 so that was after you quit. How do you feel about like medicalization of it, or you know?

P: I honestly I think the whole medical thing “oh, would it prevents cancer and helps the-. “ No, it doesn't. No it doesn't. There's nothing in that it might take the pain away, but that's it. It doesn't help with the cancer. It doesn't prevent cancer. It doesn't- no all that is lies. Unless they can show proof of how it helps. Unless they can actually say, okay, this person has cancer we're going to do a test. All right, then I believe that it helps them. But til then I don't help nobody. It just gives them that relaxation and it takes the edge away from the pain. That's it. All it does.

I: Why do you think they have like shifted it to be medical marijuana no now?

P: Because so many people are smoking it. So many people went to jail for it, for having it on them, and if they got tired throwing people in jail for it. Because there are so many- before it became legal so many people were going to jail for, and they were getting released, going back and getting released. Like I think they got tired of arresting them and releasing them, just to arrest him again and release them. So I think that's why they legalized it. Is because everyone’s smoking it.

I: Yeah I think that's a good point. It's very- it's more common and there's so many nonviolent drug offenders in the prison system.

P: Right.

I: Do you remember like this was four years ago, so you haven't smoked marijuana in that time. Do you remember, like what you were smoking daily like a gram, an eighth, or like whatever like the amount?

P: Oh. Um, daily. Let's see. Probably in an an eighth. Sometimes a little more, but for the most part an eighth every day and I don't- I don't know why I did it. I don't even know why I even picked up that first blunt and said yeah I’ll try it.

I: When did you try it? When did you start?

P: Uh, when I was 16. I went over to my friend's house because we were doing a school project, and she had it because she was holding it for her boyfriend. And she never touched it a day in her life, but her boyfriend came over because we were all working on the same project. We went to the same school, so we were working on the same project. Well, he he rolled a blunt and he asked her if she wanted to do it. She said no, and then she looked at me and she looked back and she was like you know what I’m gonna try it and then he asked me, and I said- I was thinking about it. I’m like maybe and then he goes here, just one hit won't hurt. Well that one hit ended up turning into oh okay, I’m just gonna keep doing this. Yeah so I can't say that I got addicted to it because it was so easy for me to quit, but it wasn't that easy.

I: mm hmm. Yeah. What was the hardest part of it, would you say?

P: Definitely trying to find another way to relax. That was the main struggle trying to find another way to relax.

I: Yeah, what did you- what did you find? What do you use now?

P: Well. Sorry, I got cats and they’re just running. For the most part, I like I’m an arts and craftsy person and I like to do hair, so I ended up doing like side jobs of everyone, like all my family and friends hair. And then I started doing nails, so that was kind of like real calming. It was fun, for me, and then it got to the point where I started coloring a lot and ever since I had my son there is no time to relax. You sleep when you sleep you're relaxing.

I: Yeah that makes sense. That’s so cool that you kind of you used arts and crafts and like just other kind of like creative stuff at first to help you calm down.

P: I mean still to this day, I still do hair and nails because I love to do hair. I’ll dye hair, I’ll curl it, I’ll style it, I’ll cut it, whatever you want me to do, I could do it. Um, I didn't go to school, for it I’m self-taught. But I’ve had so many people that don't want to pay, you know 150 some odd dollars, just to get their hair done. So, I asked I’ll go to a salon and be like “okay, what would you pay, what how much, would you charge for this to be done and how long do you think it would take. So what would be your final price? They would tell me and I’d cut it in half because I’m not technically a professional shop, you know cause I’m working from home, but it's something.

I: Yeah and I’ve had professional shops like do me dirty, not and I walked out of there not liking it and I feel like I’ve heard other people that just kind of have the gift, you know.

P: Right.

I: being able to understand what people's faces look good with, their hair texture, and all that stuff is-

P: Yeah.

I: Yeah. I want to ask a little bit about tobacco use too. Kind of, you talked a lot about marijuana but I’m also curious about like what you think about tobacco use.

P: Um. Well, I really. Honestly, I just quit smoking cigarettes like two weeks ago. I know so proud of myself. I haven't touched one since. I’m not craving them.

I: That's hard. I quit smoking before and it's so hard.

P: yeah like everyone around me smokes so, so it's like okay, do I want it? I think I want it? Do I need it now? I don't need it, but you know I’ll get them cravings here and there. But. I- I have Italian in me so for me not to be doing something with my hands, it drives me crazy. It really does so that the motion of the cigarette every day that was like my number one thing. After I quit, I didn't know what to do. I was looking at my hands and I’m always moving them. I’m doing something with them. I it was hard to quit. It was. It's still a struggle, right now, because of the craving but… I can't say craving but the temptations there. But I don't need it, I want it, but I don't need it.

I: How long did you smoke for?

P: Hah, oh. Seven years and I’m only 21.

I: Yeah, I mean if you grow up in a home where everyone smoke so they're always around. It's not hard to get them when you're very young and I know the drill

P: Yeah, my mom, my adoptive my mom wasn't happy when she found out that I was smoking them. But, she said that she would rather I smoke them in front of her instead of hiding it, so I did it for a good year without her knowing. And then she found out because she walked into my room and seen all the ashtrays and all the cigarette butts and I tried to keep my room clean, so she couldn't smell it, but she seen that I was smoking. So, I mean like I said she wasn't happy with it, but she told me, she was like I’d rather you smoke in front of me than try to keep it a secret. And I- I’m kind of happy that she had an understanding she goes, she was smoking, since she was 10 because of her mom and everybody in her family so. She, she really wasn't upset with me which I’m kind of shocked because she's one of them “be a good girl.” So, I was really shocked when she said that. But, right now, I don't understand what it did. It was just that motion. It always kept me busy, so I don't even know why I picked up a cigarette either. That didn't really didn't even do anything for me, it was just something to do

I: Yeah. Di you smoke when you were pregnant the first time or did you try to quit, did you cut down, did you keep going? What was your-

P: Oh no with my first pregnancy, I smoked. I probably smoked a half a pack every day with him. But I did eventually cut down, and I was smoking like five six a day, so I was able to make a pack last me quite a while. But, um, three months before I had him I did quit. I quit smoking cigarettes and then, after I had him I quit and I wasn't smoking for what three and a half months after I had and then the urge to pick up that first cigarette because it wasn't for me, but I picked it up and I lit it, and that was like okay now I need that.

I: Yeah, they’re so addictive.

P: They are. They are.

I: Was it hard to quit when you were- that first time?

P: Not really, I mean the first time it was it was hard in a way, but I just- I completely stopped. I don't know how I did it. But I completely stopped. I bought a pack of cigarettes and went through every single one of them, you know within a couple days. And I just never picked one up after that. I don't know why. I don't know how I quit but I just quit. This time it was super hard. Yeah this time it's a struggle. I want one but I don't need it.

I: Do you quit cold turkey or do you do it like, what's your method?

P: The first time I did it, I quit cold turkey. And I felt better about myself, though, because I had more energy. This time I cut myself down.

I: Yeah.

P: Yeah, so I was smoking, maybe a couple hits off of a cigarette here and there, because I used to short them. I’d smoke half, put it out, wait a couple hours, smoke half and be done. But before I quit for about three days, I was taking a couple hits putting it out, take a couple hits, put it out. And then it got to a point where I was, like all right, I don't want that, so I was always keeping myself busy.

I: Yeah. I feel like business, is important for you to just not just to be able to forget that maybe you want one.

P: Yeah

I: Do you think that, like one like marijuana or tobacco is like worse or better for you?

P: um, honestly if I had to say one was worse, I definitely say tobacco is worse. I can't really say that marijuana is worse only because I’ve had the effects of both of them, and my family has. My whole family has been smoking cigarettes and two of them actually have lung cancer because of the cigarettes. So it's like if I had to pick one or the other to be worse, definitely tobacco. Because I still have yet to find out what's bad with marijuana.

I: Do you talk about like quitting smoking with anyone? Like do you have like support for that or is that something you do on your own?

P: I. I can say, I have some support but not really. It was something that I it's something I do on my own like. I don't know how to explain it but it's just when I quit I I was by myself. I told myself I’m done that's it. I want a better life. I have asthma, as it is so it was really taking a toll on my lungs and for me to quit and be able to actually breathe again, I feel so much better because I I couldn't even run. When I was smoking I couldn't run. If I ran, I was done> I was gonna have an asthma attack. Now I can run three miles and back and be fine.

I: It does feel good when your body starts to like heal. Which one, do you think was harder for you to quit so far?

P: Definitely cigarettes because I’ve been doing them longer than marijuana. So, definitely cigarettes.

I: Did you talk to your doctor about cigarette use or anything?

P: yeah and they tried to, um, they tried to give me the patches and they tried to prescribe the- told me to do the nicotine gum, and all this other stuff and nothing was working. So.

I: Yeah. What do you think makes this interview with me as a researcher different than like a conversation with your doctor?

P: Honestly with doctors, I feel like you have to watch you say because some of them, some of them out there will just be like “okay well, you did this? Oh, I’ll need to report this” not “oh, you did this so let's see if we can cut this down” like smoking cigarettes. Let's see if we can cut this down and we'll see how we can go about it. See what you can do to quit instead of “Oh well, you're doing this? Oh well, we need to report it.”

I: Yeah, how can they like, how can doctors make themselves like more approachable or like how, how can they change that so you would feel more comfortable opening up to this kind of stuff with them?

P: I think if they didn't have to report every little bit of information and try to be that one little- try to have that little bit of insight to talk about whether they did it or not, to give us a little help, I think that would be better. Instead of jumping to conclusion and saying “Oh well, since you did this, we have to report, because this could happen.” But if they would sit there and say “Okay, you did this. Do you want to do it again? Do you want to stop completely? Do you want to go back? Like what do you want to do? And try to help us, I think that would be more beneficial on us. Because then this way, we have more insight of what we can do to change it.

I: It sounds like the interaction, you had with the like the system like as a result of like your medical care was really scary.

P: Right.

I: And that, instead of like a treatment approach, sometimes it sounds like the doctors take a more like punitive or like judgmental stance, maybe, or whatever. Yeah I can see that, and why, having it like what you're saying is having a different approach would make a really big difference.

P: Right.

I: Yeah. How do you think we could like get information about this stuff to women, like you, like information about marijuana and tobacco use and them together?

P: Um honestly I feel like it. I honestly I feel like if if there was- (background voices) If there was studies- I’m sorry.

I: It’s fine. We're all doing the best we can.

P: If there was studies that would be like okay here's my proof that smoking does this to help, you know, smoking tobacco does this to help, um, and they actually showed us proof then maybe that would help us. And, just like the same thing we'd if they showed studies “Okay, we will help you with this and, here is my proof,” then I think that would be better. It'd be beneficial to us because then we know what we're working with. But as of right now it's a he said she said type thing about how does marijuana help you.

I: I am wondering, too, if you think there's a difference between like using marijuana alone and using tobacco alone or like using them both together at the same time.

P: Using them together like you if you smoke marijuana first and then you smoke a cigarette supposedly your high lasts little bit longer than what it should. When I was doing it myself, I don't think that actually worked. I think it was the same same thing all the time. You smoke, you get that high just because you smoke a cigarette doesn't mean anything.

I: Yeah, yeah do you feel like there's one it's like worse to do both of them together or is it the same, if you’ve got a guess?

P: Um, I think it's worse, honestly, because you're really hurting your lungs right, then so.

I: Like the physical damage.

P: Yeah.

I: Like its lunchtime now mother. Okay, I want to thank you because I have like I found this interview so informative and you've been like such a wonderful participant. Really probably my favorite one don't tell anyone else. Do you have any like suggestions on how I can like ask questions better, things that I said maybe that didn't come off good like, you know, anything about the interview that you would change.

P: Honestly, no. I think-

I: Aw, that’s nice.

I think for me no because you were straightforward and you didn't try to like sugarcoat anything, so honestly I think the way that you were asking was, was good, it was perfect. The way you were approaching the questions that was perfect. You don't have to change it.

I: Thank you. That’s nice to hear, because this we're I’m still like new with this, so I feel like nervous. I’m nervous. I want to do a good job and make sure I asked you like the right questions.

P: Yeah.

I: Right yeah. And if you think of anything you can like email or whatever is best for you. Okay um oh wait I forgot has a pandemic change this at all for you.

P: No.

I: Yeah, because you stopped.

P: Right.

I: That question is like starred, and I just forgot it. But, um, yeah, thank you so much. If you like this, we could do this again at other trimesters or like maybe after you have the baby, we can check back in.

P: Okay.

I: Yeah but I’m going to put the $20 on your card right now and I guess, I mean that's all I have unless you have questions or anything?

P: No.

I: Okay well so nice to meet you miss (participant’s name).

P: Well, can I ask like a personal question. I’m sorry.

I: Of course.

P: Okay, you look like you're really young like you look like you're 22-23 do you mind if I ask how old you are?

I: Not at all. I am 32. I’ll be 33 this year.

P: Wow, you look really young.

I: Thank you! I use prescription acne cream and it like-
