# Supplementary material for: Experiences of postpartum mental health sequelae among black and biracial women during the COVID-19 pandemic
Source: BMC Pregnancy Childbirth. 2023 Sep 4;23:636. doi: 10.1186/s12884-023-05929-3 (PMC10478375; doi:10.1186/s12884-023-05929-3)
Supplement: Supplementary file 9 — Supplementary Material 9 [file 12884_2023_5929_MOESM9_ESM.docx]

**Supplemental File 1.14 Interview Transcript with Participant 5276**

Interview 5.20.22

43 minutes in length

Okay, so i'll frame some of those questions like that, but if I ask something and you'd rather, like I ask it differently, or if there's something you want to correct me on--like don't hesitate. um (Okay) so we kind of start out with a general question about what it was like for you to be pregnant?

Hmmm, it was painful, it was really stressful. Um the idea, like the metaphorical idea of like oh i'm about to have a baby and be a mom that was beautiful, but, when it came to you know, dealing with the physical things every day it was definitely a hassle, it was definitely a lot.

Um do you have like specific things that you remember that you were like ughhh?

Ummm definitely me being, you know, i'm only for 4’10”, i'm only 4’10 and my average weights usually a around 130. You know, just all that extra weight was just a lot, I remember being in pain all the time, I couldn't sleep right. Um you know and definitely I got winded so easily. And I tried to stay as fit as possible but then next thing you know they're like you know just make sure you're sitting down don't move around too much, but yeah, yeah. It was definitely just definitely the back pain, you know my have my stomach stretching out so much so fast definitely hurt that too.

Yeah, people never talked about how it like what we do talk about it but it's not like part of the common discourse where we hear people talking about like you know it must be uncomfortable for some people's skin.

Yeah its unbelievable. Like, you know, when you don't realize you're pregnant it's just like whatever, I look normal to myself. But then they tell you, and you're like oh man Im getting big. and then a month goes by and I'm liketwo times the size, I was. yeah.

That would be hard for me.

Its, it's ridiculous. Like I said the metaphorical ideas around it, you know it's all really beautiful, but when it comes to actually dealing with it physically, it's definitely, it's definitely a lot.

like how you put that like you know you're acknowledging that it's a beautiful concept and like an important one.

Exactly.

But the reality, there's other parts to it too.

Yeah, yeah also the morning sickness was really, really bad.

Um did you have that intense at the beginning, or was it intense the whole time?

Most of the time, like the last two months was when I finally stopped throwing up, but throughout the whole time like about, about two months in awful it's awful throwing up.

It must be hard to be in pain and nauseous all the time.

Yeah, I couldn't eat anything, like I i'd eat things anyway, and I'd be like I already know that I am going to throw this up. But it had to be so specific like, I’d have to eat like soup. A lot of soup, in order to not throw it up--soup and crackers.

It would be hard for me to not eat like know exactly like I would be like this is probably going to i'm going to regret it, but. I'm also like mean when i'm hungry, I mean when i'm in pain like.

Yeah I was so cranky about it all.

Yeah, well how are you feeling now?

I feel better, I feel good. You know, i'm like, you know, I'm staying active around the house. You know, I play with my son a lot, you know I do all those kinds of things. So, I'm pretty active. I feel good, I can eat, what I want now too so.

Good i'm glad to hear. Okay, so i'm going to switch gears but i'm going to bring into the conversation, like the questions about health care. Particularly your health care around your pregnancy, and so I really want to know, like as honest, as you feel you can be like everything, like the good parts, the bad parts.

Meaning?

Like with your doctor with the your Labor and delivery experiences.

Okay, okay

I mean with, with all of that it was, it was pretty good. My doctors were really understanding my doctors, tthey definitely treat me right, you know. If anything, you know, i'm going to say that with my mental health history, my past mental health and everything. And you know I told him about any past injuries i've had, they took extra special care of me. They were really on point with taking care of that keeping update on questions and making sure they ask specific questions, just to make sure I was able to answer it all, and everything and they can understand me well. So I had a really good experience with my doctors.

That's really good to hear so they were open to talking about your mental health and, like those kinds of things to.

Yeah yeah and they were really supportive. They were they were really, they were super supportive. And you know I can't lie, I only have my dad around and as a man he cant really understand everything about pregnancy, but um, I can say that my doctors were another support system for me, and you know they kept me positive about everything.

That's great that's great i'm so glad that you had that experience because it's not. Yeah there's everyone has a different time during pregnancy and it can be so stressful and so beautiful, so i'm glad that you had that support.

Yeah it was really awesome, yeah I loved my doctors. I even had my regular Ob gyn come and visit me on the day I gave birth, you know. She came in my room. I thought that was awesome. She was there, she just happened to be there, she found out I had my baby so.

I love that. Can you think of the things that like we're really important to you that they did, like any like skills or like how they talk to you?

I think some important things is, you know, if I missed an appointment they would, they would call me, and they would just make sure I was doing okay. And you know they wouldn't base it off of oh, you know anything, besides I just hope she's okay. That was one thing cause that felt really good to know that people were there and making sure that, you know, I was most of all taking care of myself, because I really needed that at that time. And also them, you know, they gave me a lot of, what is it callled they basically they got me into a lot of programs that helped me out, you know learning stuff about how to take care of a baby and what to do as a mother and you know. And even how to you know how to properly reach out when things aren't doing so good, or I feel like I just can't do it anymore. Um yeah they just like, I got an programs that basically prepared me for everything about being a mom. Um also, I, you know, with this injury (gestures to scarring on face), I had some brain damage and, you know, they got me to really go and take care of that and everything so that was good. So they basically, they would just make sure I was taking care of myself and make sure I knew what I was prepared for and kept me positive the whole time. Hmm so it was just like I said I had a really amazing doctors.

Yeah it sounds like they were like you know, obviously it's like Ob gyn to they're doing that stuff. But but i'm hearing is that it was also like they got you connected to resources. yeah she took care of your mental health, they took care of some other injuries that you like, it was very holistic care.

Yes, it was it was it was amazing.

That's awesome where did you get where did you go to the doctor?

Um, I went over to Alma Illery. I don't know where that's at I forget where that's at, but I know the place is called Alma Illery.

Okay, so you didn't go through Magee or anything?

No, no, I started going to Magees after, after I needed to see like a specialist for some things because they wanted me to go see doctors, because they wanted to make sure I wasn't high risk.

Mm hmm, so you started at mcgee and then you switch to on the alma illery

Alma illery and then magee.

What were your experiences like at Magee?

Oh yeah absolutely the same thing, they were all like I said, all my doctors altogether were really supportive really good with me really understanding.

Is there anything that you, you know anybody experienced that you didn't think super positively about?

No, no, I, for the most part, I had a really positive experience I really did

That's amazing.

I never know how to like bridge these questions perfectly so it'll probably be a little bumpy but did any of your health care providers during your pregnancy talk to you about your sexual identity like being pansexual.

No, no, they never really truly asked about it um. Ugh yeah know they didn't really ask about that much.

Would you how do you feel about them talking about that with you.

I wouldn't have minded I wouldn't mind it, I would have been okay with it. i'm, you know, i'm a really open person so for the most part, you know, I would have felt really comfortable talking about about all of it, but I don't I just don't remember them asking about it that's all.

Do you feel like it's something that is important to ask about her doesn't really matter in the Grand scheme of what you're doing.

Truly I don't think it wouldve mattered, do you know what I mean. I think it kind of does though when it comes to mental health, because I do believe that stuff can affect mental health. You know, I mean it's good to ask the questions about that about a person to figure out, you know, maybe they're going through something else that others wouldn't understand, and you know I can help them understand that as well. So I guess it could be important yeah.

Like in terms of mental health stuff do you mean like just like discrimination or like how it might make you at risk for certain things, what do you, what do you thinking about.

Yeah definitely. Anybody who isn't heterosexual is definitely more at risk for for judgment, for you know, some people don't get accepted by their family, some people have to hide it, some people don't get to live their lives that way, and you know others just really fully are just discovering themselves and know they'd like to talk about it. It can affect mental health, it can affect your daily life, how people treat you.

Have you um it did being pan sexual like affect how you thought about being pregnant at all.

Just going away yeah.

Can you tell me how it did or?

I guess, I want to say because I kept telling myself like, I really don't like men anymore, I don't like men anymore, because, you know, I sleep with a man and then he doesn't want to be a part, a part of my son's life that broke my heart. And then i'm like I could be with a woman, but then i'm like, oh i'm having a baby how am I going to be with a woman and have a woman actually trust me? You know she's like you're just going to go run off, I feel like they're gonna, say oh you're just going to run off with a man. You know I love women, women are beautiful, they're amazing in every way, but, you know, I don't know, I guess, I guess, sometimes I feel like it hinders me as being taken seriously as a pen sexual woman. You know, for anybody, because you know i've done this thing that most people would consider just for straight woman. You know what I mean, I love the idea of being a mom you know, I was so for it, but I also think about, you know, future relationships and, finally, finding something serious and how it might effect that.

Yeah that is such a thoughtful response because it's like you know there can be like you're not straight enough or gay enough kind of thing which is tough.

Exactly like that yeah.

Especially what you're talking about. In relation to being pregnant and now being a parent is another kind of like element to fold into that and I think that's really interesting to think about because we're just kind of starting to talk about it.

Right, right.

Um do you think like so kind of like the reverse of that question so like you kind of just told me how it affected your pregnancy, do you think your pregnancy affected, how you understood your sexual identity at all.

Oh. OK. So no.

They are kind of interchangeable questions. You kind of already answered it I think. let me see so did you think that the physical environment of the health care centers that hospital was like at all welcoming to LGBT Q plus people.

Yeah I think so. I think it seems very welcoming.

What made you feel that way.

I think, because I was surrounded by, you know, different kinds of doctors, all kinds of people and, you know, for the most part they just treated me, you know, I love how my treatment, especially at the hospital, was based around just my son, it was just about the baby, it's about the baby. And you know, of course, I have to take care of me to do that, but no it's just about the baby and that was the best part.

This is kind of the last, thank you for sharing that this is kind of the last question in this section and then we'll go and talk about like marijuana and other stuff. um but what I guess like if we are talking about you know, helping doctors or nurses create some kind of like script or training, where they learn how to talk to people about their sexual identity like what would you want to hear to feel comfortable talking about that, with your doctor.

I think i'd want to hear that I was going to be treated the same, with just as much priority, with just as much seriousness, not just as someone who you know, may be confused may be you know, making the wrong decision or i'm just unsure myself. I'm very sure myself, I just want to be able to feel like they know that I'm sure myself, they know that I am this person and a person in general. It’s about knowing that you're going to be accepted as the way you are, and you know treated with just as much respect.

Yeah that's such a. Beautiful response, thank you. Okay, so, would you like to I want to give you the choice, do you want to talk about marijuana or tobacco first.

Let's talk about tobacco first.

Okay let's do to backup So what is your can you like, just so I know how to frame the questions with your tobacco use history kind of like generally.

Um, I smoked a lot of my life. You know I quit here and there, for like a couple months at a time, but for the most part, I smoked since I was about maybe like 16 or 14 one of those years.

Can you like this is one of my favorite questions in both the tobacco and marijuana marijuana sections, can you like set the stage for the first time, do you remember the first cigarette or blonde or you know, whatever tobacco product, it was that you smoke.

Yeah, yeah I was, I was 14 and it was summer and my sister, my older sister she was smoking at the time, and she had a whole pack a sister itself, somehow, I guess a friend had bought it for her. But the parents were gone. At least my mom and, at the time, her boyfriend we kind of grew up with them me, my sister, and my stepsister we like grew up with that being together. But yeah, we were all sitting on the back porch it was summertime and my sister had dared me to hit a cigarette for her. And um, so I did, I did, and then I was like hold on, let me keep trying and keep trying, and then I finally tried, it like it was like four puffs before I actually like stop coughing and I finally got it right, but it was like that.

yeah it takes a little bit It is like a skill skill, but like a technique almost. yeah yeah. : I feel like you can often tell when actors and actresses are not actual smokers in movies yeah okay so it's with your older sister did your family smoke like growing up.

Yeh ah my mom and my dad did, both yeah.

So what do you like about, do you use cigarettes or do you vape? And what do you like about them?

I use cigarettes, I use cigarettes. Um, they start my day, they start my day. It wakes me up a little bit in the day time. Um, it gets me, like I said, it gets me up for the day, it gets me moving. And then, you know, I don't know if it's because i'm a smoker already, or if it's just because I do get stressed, but when I get stressed, and I have a cigarette it definitely helps me like take a breather, calm down, it's a good thinking moment, you know just were like (exhale) that.

I do now, I do yeah yeah It is like : It does do some emotional calming there.

yeah it does.

So when you are there things that you don't like about smoking like are there, bad sides to it.

Of course, of course um you know, like, like for my whole life, I've had to deal with like, you know, the stuff, like the costs, that come with like running out of breath easily, I don't like the health issues that comes with it. But also like I hate having to take my time away from doing something just for that moment, you know. I think to myself, like I don't even really need need a cigarette, like i'm not going to die without one, but you know, I definitely want a cigarette so let me just go take my time real quick. You know, like I said it takes time out of my day I could be doing all kinds of stuff at that time.

It also sounds like that time kind of like your time.

Yeah yeah.

When you were pregnant did that change your relationship to cigarettes what was that, like, for you.

Um when I was pregnant, I was smoking a little bit, I wouldn't smoke as much, like i'd have like maybe like two cigarettes a day, if it was everyday, mostly like I say like five days a week, and for those five days and have like maybe two or three cigarettes a day.

How cutting down like how was that for the harder easier.

Being pregnant with my hormones everywhere, it was pretty much easy. It was okay. It was, I felt guilty every time I had one, though, because, like, I know, like some health issues come with it. I just felt guilty every time I had one, that's that's kind of how it changed my relationship with it. Just made me feel like I gotta quit this stuff, I gotta quit as soon as I have my son, I'm going to quit and then I did for like, you know, a good two months, and then all of a sudden smoking cigarettes again.

I feel like there's a lot of guilt around just being a mom in general, and this is so, what he said seems so insightful that it's another place where there's probably some guilt. Were there things that triggered you during pregnancy to like want a cigarette.

Yeah sometimes me and my dad would argue. And you know, my dad, my dad I love my dad, he's a good manm he's a really great dad, you know, he supported me a lot but you know, sometimes he would get cranky. And the I would get like secondhand cranky and because I was already cranky like we get into these huge fights that were like over absolutely nothing. And I would like alright dad, alright dad, I just i'm going to take my space and I am going to go have a cigarette and you need to take your space dad and ill go outside and take mine. So yeah, just these little fights we got into, just little fights. And i'm definitely also you know I guess just sometimes I put a little bit too much effort into my day or i'd have to clean something and it'd be like it's just too much I can't do it, i'm in pain, so Id just have a cigarette about it.

Are there things when you wanted to smoke, but you were like no i'm not gonna i'm cutting down right now, are there any things that you found helped you.

Oh, I guess, I guess, like the fact that cigarettes, while I was pregnant made me sick. For the most part, like I said, that’s why i'd only have like a couple because the more I had in the day, the more they were going to have the ability to make me absolutely sick. So I kind of have to cut down, it turned me off from that, you, its like being pregnant and not wanting to eat certain foods just because you don't like the smell.

That natural incentive.

yeah exactly.

Did you have any conversations with doctors like how are your doctors without talking to you about this stuff.

They were good, they were okay, I was open and honest with them. I'm like Im still smoking a little bit. You know, they reminded me of the risk factors, and you know they said, if you need any help with like a patch or anything just let us know. So they were like, you know, all there for me. They were like, all right you're going to make your decisions and we can't stop you but also if you really want the help, if you really want to you know stop doing this, we will help you with that as well.

So, they told you about what could happen well also. What i'm hearing it's like meeting you where you're at with it. yeah yeah. um do you think that being pan sexual has anything any relationship for you to your smoking habits or have having started smoking.

Ill say, and this is going to sound weird, but I want to say yes, in a way, because when I was like going through this whole thing with women and, like all kinds of like trans people like, I had this one like 2 your phase, i've been calling it a two year phase cause like I was just like totally turned off by men, and I would like, I would flirt with women by offering them a cigarette, and I would flirt with trans people in by offering them a cigarette (laughter). It’s like hey, want a cigarette? Hey you want to smoke? And that’s how I would try flirt.

um I think that's such a brilliant connection to make because you're giving a very specific example, but what i'm thinking about is how like you're in a like how I would code, this is by saying, like you know it's a social connection mechanism.

Exactly.

Exactly yeah yeah, which is so interesting because it's you know it's true.um okay and it's also interesting that, like.The people that you were kind of hitting on all have these you know sexual and gender minority identities and they also all smoke.

Yeah, yeah it worked it's not like it didn't work it doesn't. work.

Like I have evidence this is an Effective. hitting on people mechanism. good okay. So is there anything else, before we touch on the marijuana questions that you think is important to talk about for like the tobacco section.

I think this was like already pointed out but i'm just gonna say it, because it kind of boggles my mind how like cigarette smoking in the community, and you know the LGBT community, I think it's a bigger thing, because I think, mental health is a bigger thing for people like that, for people like me. I think if youre like, you know, whether youre bi, trans, pan I think definitely there's a lot of stress that comes with it and um mental health issues and bullying and all that. I think they find themselves smoking more often, at least it seems like it.

you're right it's it's true. So thank you, because like you oviding your insight into why you were doing it, you know, we were able to like laugh and find that funny, but that is super useful information.

Absolutely.

um okay so let's the marijuana questions are going to be exactly the same, but I just want to check did you smoke marijuana during pregnancy at all.

No.

that'll just change how I asked them. Okay, so will you then, then I haven't reminded you yet so I feel like it's important we won't talk to you this is not part of your healthcare anything so we don't share with up anyone. Can you tell me about the first time you use marijuana?

I was also 14, this was also that summer. Ugh my step sister at the time took me to hang out with her friends and they were like smoking a blunt around me, so I just like, they asked me for wanted to hit it and I did. I barely remember that experience, I really like I just kept hitting it was like yeah this is fun, this is fun and then it got a little too fun next thing I know it can stop laughing.

That doesn't seem like a bad experience.

It wasn't bad, you know, they were really cool people you know. And they kept telling me make sure you're careful, make sure you're careful, so at least like, you know,they were conscious of the fact that I wasn't they weren't sure if I could really handle it at first.

And you did get like you're telling me you did get high that first time like you had the effect from it.

I did yeah.

So, what is your relationship like with marijuana throughout your life kind of since then.

Um, it started out okay, it started out good, like it was a social thing, all of me and my friends were doing it. So it was like that, you know. We would all go to parties and everybody would have a blunt or, you know, thered be like a big group hanging out and we'd run a couple plants and stuff. And it was definitely just like a big social thing at the time, um but, you know, as my life progressed on it became, it became kind of like this negative thing. I will say because, right now, i'm like completely against it, I can't do drugs anymore, just because. I do believe it is a gateway drug, you know, it started with just weed and then it became other things, so when I look back on it, you know at the time, I was thinking that it was okay, but I look back at it and really it just wasn't okay.

So what it. yeah what you experienced, then you know you have retrospect now and it kind of changes, how you understand.

Yeah, I was a bit too open minded about just like marijuana in general, like i'm like if this is okay everything else must be okay, you know. And you know, I was irresponsible with the way I used it so.

How did you like, how was quitting for you, was it hard to quit marijuana.

No, no, when I finally stopped doing all the drugs then I stopped smoking weed, you know. It definitely wasn't hard, I was just like, so over, you know, being high all the time, or you know not feeling the like the energy I deserve to have, and all that stuff.

So what was it doing for you, that was good, and then you know what was what was it taking away from you.

I think, when I was going through my depression really bad, I think it definitely helped me feel good, but it didn't help me get up and actually do something about it the right way. So, you know, I could be in my house comfortably and not feel suicidal or, or just feel really down, like I felt good, but I really needed the energy to actually get up and, you know, go see a doctor and go get meds, and it kind of stopped me from doing that.

correct me if i'm wrong, so what I understood was that, like it kind of was like treating you in the moment but more like a bandaid than an actual.

Exactly, exactly

that's a very. Like fascinating perspective that you have on that now When you were quitting were there certain like triggers that you found you had to like stay away from or was there anything that helps you stop.

Um I just really had to, there weren't really any triggers, it was just more about staying away from the people that did and try to coerce me and get me smoke it. Like hey, I bought you some weed today if that’s okay lets go try to smoke it. I just had to cut those kinds of people out of my life, that was basically the gist of it because I knew I wasn't gonna put in the effort to actually go look for it.

Were you able to find new social supports that didn't have that

Of course yeah, of course.

Did you feel like there is a relationship between like you know being pan sexual or trans and having that Like you know good one group to smoke more than the other, or something like that.

I believe so, yeah, I believe so, because you know most of my I don't know. I was living in Florida when I when I was quitting, finally, but, you know, I remember, I remember the people who had tried to get me to smoke, the most-- actually no, nevermind because I did have a few straightfriends that you know would try to get me to smoke. I don't believe there's any correlation per se I believe it does different things for different groups of people. I believe it's wide variety and range of people, though. Not based on sexuality, I guess.

different things for different people. yeah I feel like I had a hard time like kind of asking figuring out what I was trying to ask that question, so I apologize, it was like a little that's a little confusing, as it was sleeping my mouth, I was like What am I even say. see, let me look at the piece of paper and keep them okay So what are the only doctors and healthcare providers like, how can they talk to pregnant women, about marijuana use.

Good question because right now, I do have a friend who is pregnant, who is using and I don't know, I don't know, I don't know the background behind it, but I believe, you know, they should inform people like they did with me with smoking cigarettes, you know. I think it's good to inform them about the risks and, you know, kind of do that same thing, where they say “hey if you need help quitting, we'll do everything we can to help quit”. You know, it's good to go over maybe why they use it, why they use it is a good question. I think just going over all the information to help understand the most, overall i'm not sure if it's illegal or not, But you know if it isnt, if its not illegal then i guess maybe just you know, trying to get people to do what's best for their baby.

yeah I think the legal stuff is changing so much too.

It is, it really is. Especially, you know, with people getting their medical cards all around now, you know, because there could be healthier options or there could not be. Im not really sure of all the science behind all of that, I just know it's good to treat it like a cigarette, I guess treat it like a cigarette.

yeah. I think that also makes it simple.

yeah absolutely.

Because people seem to you know we're like more aware of the health risk of cigarettes, after years of the campaigns to make us aware, but I think with with marijuana we don't have that clear messaging. Exactly yeah that's a good point Okay, we are moving into well there's actually forget about these questions, all the time, so i'm just going to condense them.um did you use like marijuana and tobacco together and, if so, like how did it change the experience of like being hired to do that

Hmm I did, and it gives you, I used to do it because I loved getting like this head high. Like I’d feel buzzy all around my body, you knowm i'd be like, everything would be so like kind of like a blur but understandable. I love smoking cigarette because it'd be like all in a rush and it helped it, like just kind of, I always looked for like the physical effects like, you know, the pain reliever and, you know, the anxiety reliever. I looked for that. So I tried to get like the high, you know, like the body and head high and then just try to get these benefits from afterward like where i'd feel really hungry and I’d get a good meal and all that stuff. So yeah, I did ,I did smoke cigarettes in and weed at the same time.

yeah and that was a very like comprehensive. So basically these are like perfect world questions so your version of the perfect world, what do you wish all like pan sexual women LGBT Q plus women knew about pregnancy.

I think they should know that just because you're pregnant doesn't mean that you are, like, completely disqualified from being who you want to be and how you feel you are. You know, when it comes to sexuality it's, it's such a natural human thing to love. Love has no gender, love has no specificies. it's just love. if we can love her kids and we can love a dog, then I can love someone that is the same gender as well. I wish they knew that, I wish I wish they knew that it's not, what's the word i'm looking for, it's not any say about like you know how you're feeling all the time, you know. i've seen like you know men getting pregnant, like trans men get pregnant, you know, that's an amazing thing. I believe, like it's such an open, open world that we need to really be open minded and never let it make you into someone that you feel like you're not.

yeah I love that I love that because we do have like such a narrative around who can get pregnant like what. You know there's just such a history of what that looks like what love looks like so I love your reminder that there is not one picture here.

yeah exactly.

Okay, so this is the next question will be very similar So what do you wish all healthcare providers knew about pregnant LGBT Q plus women.

I wish they knew that sometimes they kind of need a little bit more support than others, because, you know, it can be a lot more to deal with in the mind and the mental than other people. You know, most people are like i'm straight and i'm having a baby and, yeah i'm not sure what to do with the baby. But when it comes to being a part of that lgbtq community, you're like okay, Well, first of all, you know i'm Bisexual and I want to be with women and i've got these mental issues to deal with them and i'm being judged and you know all that stuff. So maybe they need a little bit more mental health attention than others.

yeah and I think that goes back to like a few things that we've talked about like your experience and why it was good yeah and also when you were kind of talking about the different needs of people Exactly yeah. So the last few are going to be about marijuana tobacco So what do you wish all LGBT Q plus women knew about tobacco use.

I wish they knew that, I wish they knew that sometimes there's other ways of relieving stress. You know, I want everybody to be healthy, I don't want, I don't like the health issues of it, i'm sure that people don't. But you know, sometimes, you know, if you're really stressed and you really need it for stress, there are ways to quit that will help, you know, and there are ways to deal with stress in other kinds of ways as well.

What about for marijuana the same question, what do you wish, you know LGBT Q queer people know about marijuana use.

That marijuana use is, at some point, it's not fun anymore. and that, you know if you're medicating with it that's one thing, but if you're just looking for a good high, there's you know there's other ways to kind of just take care of yourself. I think some people look for that high just because, you know, they don't like the way they feel and, and maybe it's just about, you know, getting on medication. And seeing, you know, maybe if I still feel like I need to smoke weed, maybe i'm actually medicating with it, or you know, if i'm just chasing a high, it's important to maybe seek medical mental health for that.

yeah yeah I think that. you're hitting on like a common chord which is just that that and that people don't talk about like you know the impression of marijuana is that it is natural, and like you know fine whatever, so I think it's interesting that you're bringing to attention that like people do use it. To to treat untreated mental health conditions yeah yeah. So the this is that's the end of the interview, but I like to ask, like what do you think i'm not asking, or are there questions that you, you know, like what can I do better and basically.

I think pretty good, I think you know I got to share a lot of personal things. And I think, overall, like being part of that group feels good, it feels good I think. I think of any questions you could be asked. I believe you asked a lot of questions.
