# Supplementary material for: Experiences of postpartum mental health sequelae among black and biracial women during the COVID-19 pandemic
Source: BMC Pregnancy Childbirth. 2023 Sep 4;23:636. doi: 10.1186/s12884-023-05929-3 (PMC10478375; doi:10.1186/s12884-023-05929-3)
Supplement: Supplementary file 10 — Supplementary Material 10 [file 12884_2023_5929_MOESM10_ESM.docx]

**Supplemental File 1.10 Interview Transcript with Participant 5345**

** Very rough audio on the part of the Participant.

I: How's your pregnancy going so far?

P: I would say it’s good. Of course, I have my days where I don’t want to be bothered.

I: isn't going kind of how you'd expect it or is it different?

P: What did you say?

I: I was just asking if it's going how you expected so far, if it's been different than you expected.

P: I didn't expect to be [any kind of] way.

I: So we can just go ahead and jump into some of the main questions first of all, what are your thoughts about marijuana use in general?

P: Personally I don't use it. I don’t know what to say about it. I don’t use it so it’s just like I don’t know.

I: What about tobacco use?

P: I don't really like it, even though- I have family that do smoke cigarettes. I don't like that they do, but they're their own person [so I can’t tell them what to do.]

I: What about it don't you like?

P: The smell… I hate the smell. That’s about it. And of course it can give you lung cancer and all that stuff.

I: What do you think about tobacco use during pregnancy?

P: I will say it's bad.

I: Why do you say that?

P: you're just supposed to basically care for your child [while they’re in the womb.] And of course, you would want to come out with a healthy baby.

I: Have you heard of anything that can happen if you do use tobacco, while you're pregnant?

P: Of course I did a search for certain things I heard like a child can… -it could come out premature or have some type of birth defects, stuff like that.

I: Where do you do research?

P: On Google.

I: Are there any specific sites you always go to? What's the process?

P: No. Google gives me the answer right in there and I’ll just read it right there, but if I gotta do a lot then I [give up]. (unintelligible)

I: How do you know, like what whether you're finding on Google is trustworthy and true?

P: I say well- if they give you the answer right then and there, I feel like it’s legit, but if you gotta go to other different websites to find an answer- I don’t really… trust them. Or if I had a question, my step mom’s a nurse so I could just ask her questions.

I: Okay. Have you asked her any questions about marijuana and tobacco in the past?

P: I don’t use them. None of it.

I: Okay well, what do you think about marijuana during pregnancy?

P: I will say I will give it a negative 1000 out of 10. Even though some people say that they have to like… use it because their doctor prescribed it or something, but to me if it's not prescribed to you, you shouldn’t do it while you’re with child. Because it’s harming your child and you’re harming yourself.

I: Tell me more about what you've heard about like marijuana use during pregnancy.

P: The same thing. The child can develop slowly or slower- or slow down the growth of the baby. Come out premature…. What else? … I think like the brain won’t function as fast either. It’s a lot. I can’t remember if I (audio) or where I seen this at, but it was a lot basically nothing I seen it on a piece of paper. How smoking marijuana can affect your baby, but yeah…

I: Was that from your doctor that piece of paper and you think?

P: I can't remember where I saw it at, but I’ve seen it somewhere.

I: have other people talked to you about using them during pregnancy or anything?

P: No.

I: Okay. Between the two of them, like the marijuana and tobacco, do you think one of them is worse?

P: I would just say both are just not good at all. [You shouldn't use it.]

I: Does your doctor asked you at all about marijuana, tobacco at your appointments?

P: Yeah, they asked if I used it, and I said no.

I: How did you feel about that, like conversation and how they responded?

P: — I didn’t feel uncomfortable or anything. [Of course they’re going to ask you those types of questions. I didn’t feel uncomfortable or feel no type of way.]

I: Have you ever tried either of them before?

P: No.

I: Okay. Where do you think that you kind of talked a little bit about where you've gotten your information, where else do you think young pregnant women usually would go to get information about marijuana and tobacco?

P: I would probably say to the doctor if they want to be more sure. Yeah I would say your doctor.

I: What are the things that you think you would want to know if you were in that situation?

P: I don;t know. I wouldn;t even put myself in that kind of situation. Because I'm already afraid of what I eat, stuff like that. I just want to have a healthy child, that's all that matters to me, so my child is growing [and I’m doing what I’m supposed to.]

I: Well, do you have any idea of why some people do use it while they're pregnant?

P: And people just don't care, like, I had a friend who did it both her pregnancies, she smoked marijuana very heavily she just didn't care- she thinks she knows everything she really doesn’t. Yeah your kids might be okay now but… Maybe when they get older, they might start falling into problems.

I: Are there any other reasons you've heard that people use it either while pregnant or in general?

P: Some people are prescribed it and some people use it, because they don't care, like they just think that nothing is going to happen to their child.

I: What do you think about it being prescribed? It became like- medical marijuana became legalized here and in 2018, what do you think about that?

P: I have a grandma who is prescribed it so I don’t feel no type of way about it. I mean me because it's like (unintelligible) medication now like- it's kind of weird but… mmm.

I: Your grandmother, you said?

P: yeah.

I: What do you think about that?

P: She really has a lot of pains, and really bad back pain so it helps with that. That's all I know. I don't know what her pains are or what causes her pain.

I: yeah another kind of hypothetical question: if you were in, again, a situation like where you are using marijuana or tobacco, do you feel like you would feel comfortable telling your doctor about that, when you're pregnant?

P: Like if I did?

I: yeah like if you did?

P: Yes, and no. Of course your doctor wants to know everything to make sure you’re okay, so of course I would tell him, but the “no” part- I would be embarrassed that’s why I said that.

I: Tell me more about why you would feel embarrassed.

P: And then I heard if they do find like marijuana in your body they can possibly get your child taken away from you, if that makes sense. (unintelligible)

I: What do you think about CYF becoming involved and children, possibly being taken away?

P: I would probably say stressful.

I: Do you think that it’s okay for that to happen? What are your thoughts?

P: …I don’t know… yeah, I don’t know how to feel about that situation. [I would say that if you cared about your kid you wouldn’t do it.]

I: How do you think doctors and, by extension, CYF knows if you're using marijuana during pregnancy?

P: Of course they do a lot of blood drawing- which I don’t like. But I guess when they test your blood they do see that, and they have- they test your pee too, so they see it in your pee.

I: [And you said that you would feel embarrassed about telling your doctor about your usage. Tell me more about why you would feel that way.]

P: At the end of the day, the doctor wants to make sure you’re okay and stuff. I’m pretty sure the doctor would be okay with you doing that. The doctors have [unknown] about their patients so…

I: If you were in that situation: How would you want your doctor or health care provider to respond if you told them something like that?

P: I would probably want them to help me.

I: What does that mean?

** The Participant is almost impossible to understand during this part of the interview.

P: [To answer any questions about childbirth and causes of- how can I put it? What can I like- how can I put it? Basically the pros and cons of- what could possibly happen to the baby while you’re doing it.]

I: One of the kinds of worries that you mentioned, possibly having in that situation is like you're like CYF involvement in your child being taken away, do you think you would have any other worries going into a conversation like that telling them that you were using marijuana or tobacco?

P: No.

I: Okay. What else would help young pregnant women get more information about marijuana and tobacco use during pregnancy?

P: [You can only help a person if they want help - to me.] You can’t help a person who doesn’t want help.

I: [let’s say someone did want help. hypothetically. What would be the best way for them to get it? Is it people in their life? A program? A doctor? Like what kinds of things would help and who should do that helping?]

P: A program or if they got (unintelligible and background noise).

I: what would that program look like?

P: Something like this…

I: What about this… study would be useful in that situation?

P: Oh, can you guys ask quite a lot about marijuana and tobacco use.. Basically, help them. You can just help them- asking questions they will respond to you to give a response back on how to help them.

I: Okay.

P: yeah.

I: So, again hypothetical like in an ideal world, what can doctors and healthcare providers do to help young women feel more comfortable disclosing to them about marijuana use tobacco use?

P: Of course doctors could tell you what could possibly go wrong with your child. It could be childbirth, it can be used to being pregnant, and basically that. Sometimes like when a doctor tells you strongly it can scare you but it all just depends on who the person is to me.

I: Do you have something in mind when you're saying like sometimes the doctor tells you something and it scares you like, is there anything you're thinking of?

P: Say if you like broke your arm or fractured your elbow, or say if you had a totally bad car accident- sometimes they say you'll never be able to walk. There's people out here, who told you that so don't ever be able to walk and they’re walking. Stuff like that.

I: Yeah so when they do have to tell you, those things either you know the side effects of marijuana and tobacco during pregnancy or you'll never be able to walk again and whatever- what kinds of things can they do to like make you feel better in those situations and make those less like hurtful and less difficult to hear I guess?

P: I guess they could word it differently. sort of to the best of their ability.

I: Do you feel like you've had any experiences with doctors yourself where… they've kind of delivered bad news, or something that was pretty hard to hear and have done it in a good way, and do you know any kind of like things you noticed about that?

P: No.

I: Okay, how about a bad way, have you ever had anything where you're like really they didn't handle it well?

P: ** Note: I believe she asked to talk about a recently deceased relative. The audio is very poor on the Participant’s part here as well.

I: yeah. Absolutely.

P: He actually passed away in August. He wasn’t well… when he came out he was a newborn, but like- when he came out he had to immediately get rushed to Children’s. He had fluid in his head. And of course, Children's was saying- they tried, they tried everything we could do, but I feel like they could do more. They could have did more but… basically they told that he couldn’t breathe on his own. He was trying to breathe on his own, he was trying too hard so they put him back on the oxygen tank. And, of course, is getting right down to the wire they're like ‘he’s not getting better.’ And we’re thinking that he’s getting better but then bad news came. Basically I felt like they were rushing my brother and his baby’s mom to pull the plug, so… basically just pull the plug cause he couldn’t make it. He couldn’t breathe on his own. So yeah…

I: Thank you for sharing that with me. I'm sorry your family had to go through that. So it sounds like being rushed [or something can be really detrimental in that situation.] Do you have any other thoughts or any other examples of things a doctor shouldn't do?

P: That, for example. Rush. Yeah…

I: And at your appointment, even though you didn't use you haven't used marijuana or tobacco, where were you provided with any information about using them during pregnancy?

P: I don’t think so.

I: Any like just pamphlets or anything they said after you so no?

P: No.

I: Did anyone else at your appointment ask you about it- anyone else on the medical team you nurse or social worker?

P: No, not that I remember.

I: Okay. Do you think there's any kind of difference between talking to me as a researcher versus talking to your doctor about marijuana and tobacco?

P: No (very soft)

I: You don't think so? Do you have the same- like again it's hypothetical- like if you were using do you think it would be different?

P: yeah.

I: In what way?

P: I would probably ask more questions. I would ask why.

I: Well, do you think you would feel different about it? Like less or more willing to share things like that?

P: No.

I: Did you have any questions about marijuana or tobacco during pregnancy going in?

P: No.

I: No? Okay, and you did mention like you had- I don't know if you'd said it was a friend or someone who had used marijuana during pregnancy- Is that something you've ever talked to them about?

P: No. I actually got exposed on the Internet for it. Yeah, that kind of went viral. yeah.

I: What do you mean? Tell me about that.

P: Basically, she still has conflict between this young girl she had her first pregnancy and they basically like just keep bothering her and stuff. So, I guess, one of the girls screenshotted a picture or it was a video and a screenshot it- like just taking a picture and basically it was her while she was (audio cuts out) the same day or for baby shower and it basically just posted it was just like passionate about it. yeah. Of course, everyone had their own opinion on it.

I: In general, do you feel like you've kind of gotten messages, either from the media or your family or people you know, about what you should and should not tell doctors?

P: [“No”] (noise/ non verbal)

I: No? Okay, and I want to ask you, the American college of Ob gyns, recommends that doctors ask about marijuana use and encourage patients to quit using during pregnancy, what do you think about that?

P: What did you say?

I: The American college of Ob gyns- just kind of like the overseeing body- recommends that doctors ask patients about marijuana use and recommend and encourage them to discontinue using during pregnancy, what are your thoughts on that?

P: I mean, that’s their job so… I don’t think they’re wrong for trying to help, and if you don’t take the help- you just don’t take the help. And if you do- you do.

I: Okay we've covered this a little bit already, but can you point anything… If you've ever kind of needed to discuss something sensitive which you don't need to disclose but just things doctors have done to help you feel comfortable… If you've ever had to have a hard conversation with a doctor.

P: I would say I haven't experienced one of those yet. [I wouldn’t know.] (audio)

I: I want to ask, how has the pandemic affected your pregnancy, if you think it has?

P: Oh well… Of course, well I'm pregnant, because I feel like the pandemic has just died down [for] real, but we still have like… We- to me, I still feel like there's still rules and stuff we have to follow, but it's just like they're not as strict as they used to be. I can't really speak on how the pandemic has affected my pregnancy because the pandemic has kind of died down to me.

I: I just have a few more questions, a little bit more about your own experiences. Could you tell me why you think you've never tried marijuana before, if there’s a reason?

P: I just feel like it’s not for me.

I: What makes it not for you- like about it?

P: I don’t like the smell and how strong it is.

I: Is there anything else that we haven't discussed already that you kind of heard about marijuana and tobacco use during pregnancy either online or from friends?

P: [No.]

I: No? Okay. And how about tobacco, why do you think tobacco isn't something you ever used?

P: I don't like the smell and basically it gives you- the cause of lung cancer.

I: Is there anything I didn't ask you that you kind of want to say about marijuana and tobacco use during pregnancy or about how to help women feel comfortable at doctor's appointments and things like that?

P: [No.]

I: Okay I'm just gonna look through my guide to make sure that I haven't missed anything before we finish up… Yes, I do have probably one more.

So I asked a little bit, like why you think women use marijuana tobacco, while pregnant and you kind of talked about how you feel like they don't care and things like that- have you heard anything about like what it's like to quit using marijuana?

P: No… well, yeah I have an aunt that is currently in the midst of not using it. And I basically stopped going outside- [and I questioned like ‘why did you stop going outside? Like what does that have to do with anything?’] and she really didn't give me a definite answer but… yeah she basically just stayed in the house. She has a work at home job so… I mean, I would say I'm proud of her… that she’s trying and still going. And that she hasn't done it- and I don't know when she stopped- but the last time I talked to her was quite a few weeks ago.

I: You know, so it was hard for her to quit using marijuana?

P: Yeah, she said it’s hard, but she also uses tobacco too, so… (microphone blowout) tobacco- Like there's times, where she stopped and then she started again. I’m just like ‘Why don't you just stop all together?’

I: Did you tell you what some of the heart aspects of quitting marijuana were?

P: I guess she can't eat. [I guess that’s] why she does smoke it because I guess we don't want to give you what makes you hungry. I guess it gives you what they call “the munchies.” She can’t eat unless she smokes so…

I: What do you think it would be like to be in that situation, maybe like while you’re pregnant using marijuana to either help you eat or for stress things like that. How do you think it will be for you to quit?

P: Well what helps me when I like can’t- Basically like say if you're in a situation it's like making you angry or irritated okay really react, how you did when you weren’t pregnant. What helps me is I listen to music or I like to write. So I’ll either write or just won't bother people at all so just stay in my room until I calm down. Or I’ll go talk to my mom or my godmom.

I: yeah that's a good example. What was it like for you, at first, to change the way that you were reacting?

P: I would say like when I first found out I was really stressed out because I'm in the same predicament that I was in a couple months ago, like I say… I can't predict how long ago it was now, but it was stressful like a lot of people in that back gave negative comments and stuff like that, and I could say that I almost miscarried so that's basically like… caught me down for only like a [bit]. It caught me down for a while so…

I: Thanks for sharing that with me. So how… I mean like at that point, how did you kind of like find new ways to cope?

P: I just talked to my godmother. She was like you can’t react how you used to, and stuff like that. Mediation, my mom was telling me to meditate. Meditation does help a lot. And my cousin- She used to meditate when she was pregnant and she told me that it helped her too. [So I was like why not try it?]

I: Was that something you ever went to your doctor for advice on?

P: No.

I: Alright, I think that pretty much ends our interview. Anything else at all that you want to add before I turn off the recording?

P: No.

I: Go ahead and turn off the recordings here.
