# Supplementary material for: Experiences of postpartum mental health sequelae among black and biracial women during the COVID-19 pandemic
Source: BMC Pregnancy Childbirth. 2023 Sep 4;23:636. doi: 10.1186/s12884-023-05929-3 (PMC10478375; doi:10.1186/s12884-023-05929-3)
Supplement: Supplementary file 11 — Supplementary Material 11 [file 12884_2023_5929_MOESM11_ESM.docx]

**Supplemental File 1.17 Interview Transcript with Participant 5346**

I: So thank you also for agreeing to do this. This is like one of my favorite parts of the study. I think this is a really interesting and kind of important thing. That doesn't get talked about a lot and isn't studied very much so I'm glad that you're willing to share your experiences and knowledge with us. We kind of like to ease in so first, I just want to kind of hear about what it was like for you to be pregnant.

P: At first I didn't know, I was pregnant until I was already in my second trimester so after that it was going good until about 36 weeks because I had preeclampsia from stress and due to a whole bunch of stuff that was going on between my family.

I: Yeah that sounds like (audio failure)

P: He came early came at 37 weeks.

I: Okay, so it's good for a while, like you know around 20 weeks has been for a while and then was it like stuff just started happening in your life?

P: Well, his dad decided to cheat so I moved from West Virginia back to where my mom lives, which is New Castle. And then it just went downhill from there. A lot of drama.

I: Did the baby's father come up here now or did you have space between him?

P: [I kept space between him for a while and then] He moved back down a PA to come see him, but [he] don't see him so. I don't care.

I: Okay, so was there anything physically that was really hard for you?

P: Not really. I mean it’s his choice, if he wants or not, but it'd be nice if he sees [him] more often.

I: And you said that like… Your preeclampsia was related to being under all that stress?

P: [nonverbal agreement]

I: Yeah. I knew that that's what happened but it's scary to think about how like mentally and physically, you can be so connected.

P: Yeah.

I: So throughout your pregnancy I'll just use the term health care, but I'm really referring to, like any of the experiences, you had in the hospital with the doctors during your pregnancy. So how do you feel like those experiences were?

P: Good, the nurses were nice- they made me really comfortable, especially when I was down there alone. When I had the emergency C section, they did it pretty well and super fast so...

I: How did they make you feel comfortable?

P: Just the environment they had. They were [made] sure I had everything I needed and, if I needed anything to let them know.

I: You said that you were alone, did you receive most of your care alone or did you have labor by yourself?

P: When I was sat down to Pittsburgh, I was alone pretty much the week before Christmas. I came home after Christmas and I do believe it was, so I spent the holiday up there. But during Labor I wasn't alone, his father was actually there surprisingly.

I: Yeah. Did you enjoy his presence with you during labor?

P: I mean, I was tired during it, so I don't know but afterwards and before I really didn’t because he was being a butt.

(overlapping audios, therefore untranscribable)

I: yeah. Did you have any kind of bad experiences or not even like bad, but maybe not like things that you felt could have been done better for you?

P: At the hospital?

I: Yeah, with any of your doctors or anything?

P: Not really, no. They all were good and amazing. They did pretty much everything that they could.

I: Did you get all your care like up here in Pittsburgh or did you get some of it in West Virginia?

P: I actually got an all in PA because I like- I found out, I was pregnant, when I was down in West Virginia and then that stuff happens I moved back down so. It’s been like Pittsburgh city and New Castle. I like jumped around.

I: That's where your family is?

P: yeah.

I: Did any of your doctors, nurses, during your pregnancy, did they ask you about your sexual orientation at all?

P: No.

I: Is that something like what do you think about that? Do you think they should ask about it? How should they do that?

P: I mean if they want to ask, then I mean I'm obviously gonna answer honestly but I mean they don't have to ask.

I: Do you think is relevant to your sexual health care?

P: I mean, not really but if they have to ask that I'm just gonna be like well ‘I’m this’ or ‘I’m that’ so.

I: So they could just ask and you'll tell them and it's kind of not?

P: Yeah. It won’t be an issue like ‘are you gay?’ and I’ll be like yeah, not fully but you know.

I: It’s not a little gay you know? (laughter follows) [Do you feel like the hospital environment was friendly to LGBTQ people?]

P: yeah.

I: And if they did ask about it,, what do you want them to ask? How do you want them to say it?

P: I'm sure they just asked like ‘what's your sexual orientation?’ I would just be like I’m Bisexual and if they have a problem with it then I'm just going to get a new nurse.

I: What things would you not want them to say or do after disclosing to them?

P: Probably have an attitude, or not want to help out as much…It's not my fault. I know how some people can be like that.

I: Yeah. Was there anything like just in general, like a resource whether it's for mental health or for like diaper bag stuff like that. Was there anything that you found so helpful when that was a helpful resource to you during pregnancy?

P: Yeah there's just like an [elect] parenting group I'm in that helps with clothes, diapers and formula. They help with all kinds of stuff for babies. It's pretty neat.

I: Do you think being bisexual affected like your experience as a pregnant person?

P: No.

I: Did being pregnant affect your like sexual identity at all? Like the reverse of that question.

P: No.

I: Well, I guess, before we move on to the kind of marijuana and tobacco questions- is there anything else, like that you can think of about your healthcare experiences and being bisexual that you think researchers should know or other people in your situation might find helpful?

P: Not that I can think of.

I: Some of these questions will come back up throughout the interview, so it can be kind of circular so we'll switch to marijuana use and I like to tell people like this is confidential so we're not going to report any of this. it's not going to be linked with your name.

So let me ask about- I love this question so, can you tell me about your like the first time you use marijuana?

P: I remember, I was with my sister when I was like 14 years old, and we were at her friend's house and we were in the basement and that's basically when I first started. I haven't really smoked in like four years now, so.

I: You’ve quit for a while now?

P: Yeah. It's just boring.

I: [How did you smoke it the first time? With a bowl or a blunt?]

P: It was a bowl.

I: Was it like an older sister?

P: Yeah she's a year older than me.

I: And it was just you three?

P: Yeah.

I: Did you like how it felt?

P: I mean… It gives you a mellow vibe just depending on what one it is because there's like different strains but being young and stupid obviously you like it at the time.

I: And then, after that you said you stopped smoking for four years, did you smoke consistently after that time or once and you kind of like [didn’t smoke again?] (audios overlap)

P: When I was 14, I’d smoke like twice, maybe three times with my sister, and then, when I was like 18 I started smoking like pretty much every day, but then I was like ‘this is dumb. Why do I need to do this?’ so, then I was like ‘I’m going to just not do that no more.’

I: What was quitting like?

P: At first I felt weird because your body was so used to it and, like you're just all like [verbal noise akin to ‘eh’ or discomfort] cuz sometimes it helps you with like certain things, but like anxiety, or like whatever you have going on, but I just got (unknown) to me and they're pointless because one you're wasting money and two it's just not fun.

I: [So it was helping you with anxiety or something and then it stopped helping or you didn’t have the money…?]

P: The whole thing with that, like… It didn't stop helping with anxiety or anything like that, but it's just you never know what people could put in it like lace it or something.

I: [Have you had any experiences with weed that was laced?]

P: Yeah one time I had spiked - that was like way before pregnancy even was thought of.

I: What happened?

P: It was spice. (overlapping audio) It was crazy- made me feel like my brain was melting.

I: Really? For how long? How long did it last?

P: Almost an hour really.

I: So spice is synthetic?

P: It’s hardcore- like weed. Like makes your body feel like… it's like burning up and stuff like your brain feels like it's bubbling and stuff. I don’t know how to explain it- it’s weird!

I: Yeah that sounds so different from other weed related feelings.

P: Right. It made me feel like I was dying to be honest.

I: yeah Is this something that people ask for specifically?

P: People ask for that. I never have so.

I: Is it more expensive than regular weed?

P: I believe so, or it's cheaper- I'm not sure.

I: [Back when you were using more often, like everyday, what did it do for you?]

P: Normally when I would smoke out going on adventure hikes and stuff and like exploring and it calms me down because new environments and stuff always give me anxiety. I've smoked weed on walks and it was pretty enjoyable.

I: [What were the negative parts about smoking?]

P: Pretty much make you sleepy all the time or like if you wouldn't smoke, for a very long time you'd get cranky. It's kinda like the effects of cigarettes, just a mellow version.

I: Do you ever crave it now?

P: Not really no

I: So when you were quitting, what helped you be able to do that?

P: Nothing really helped me- I just quit. I don't really think I have an addiction personality so it took me like a day, just like done, and then I just continued.

I: You just quit cold turkey one day?

P: Yeah.

I: Okay, did you have anything like being cranky or any kind of withdrawal stuff after?

P: For like the first couple of days, but then after that I was okay.

I: Do you affect your sleep or anything?

P: No.

I: Do you think not smoking, you know or smoking changed like your relationships with people did it affect that at all?

P: No.

I: Okay, some bisexual people have experienced discrimination or stigma and that can affect you know their marijuana use, do you think marijuana use is connected to or related to your sexual identity at all?

P: No.

I: Is there anything that healthcare people, mental health people could do to help support, particularly like pregnant women, while they're quitting marijuana?

P: I mean I'm sure they can. I mean that never really happened to me because I've quit for like a long time.

I: What kind of coping mechanisms do you think help you now?

P: Writing and listening to music- stepping out of my comfort zone, I like to try to do new things.

I: What kind of writing?

P: Basically, like journalism or something like that. Just write down how I feel, or what went on with your day.

I: you're in an elect parenting group, is that something where you like it's not only like…like you can help get baby supplies and stuff but do they also offer social support or, like any activities and stuff like that?

P: (audio) do activities I'm not sure if they do support emotionally but I haven't really gotten fully into that she was supposed to come, like a week or go to pick up this paper that she had me fill out, but she has it came yet, but that's because she goes to Butler and like a few other towns so…

I: Okay, this is the last question for the marijuana section so as researchers, we just really want to better understand you know marijuana is during pregnancy and I know you didn't- you quit like long before you got pregnant… But do you think there's anything more important that you think we should know about it?

P: Any know that it could help with certain things during pregnancy I'm just not really sure what but… I wouldn't do it because, like I don't want to risk anything because, like I [even] stopped smoking cigarettes, when I found out, I was pregnant with [child]. And smoking cigarettes, that is, like it's hard to quit but I managed to do it, I powered through it.

I: Congratulations, I was a longtime smoker and it is really hard to stop.

P: Yeah.

I: It's the hardest thing I've ever quit- for sure. I still have dreams about it, which is so weird, very bizarre.

P: During my pregnancy I had urges obviously but then I was like I don't want to [ruin] his lungs he's already like developing late and early and all this like I literally had to get shots in my butt to help his lungs and liver develop, so I was nervous because I thought he wasn't gonna make it.

I: Yeah, yeah you were scared for him.

P: Yeah.

I: The tobacco section is super similar to the one we just did so basically I'll just repeat a lot of the marijuana questions, but for tobacco and you might be able to go into more detail, since you used tobacco more recently. So what was your first time using tobacco or smoking, what did you use to do like vape or was it cigarettes just so i know what to call it?

P: It was cigarettes. I don't know. Me and my cousin did that obviously being we found- stole them off her dad actually. (overlapping audio, unintelligible) I didn’t really get like a nicotine high or feel anything and I didn't fully start smoking until like- again 18 so… I guess back then I wasn't like… I [didn’t know] what I was doing with cigarettes. I probably wasn't inhaling them at that time.

I: When you started smoking, smoking at like 18 like did you buy your own packs? How much would you smoke?

P: At that time I’d probably smoke two or three a day. But, at the time when I was able to buy just buy it like a pack and then try to stretch it out as much as I can, and then just repeat that.

I: [Okay, so you were always trying to make it last a long time?]

P: Yeah. Like I smoke cigarettes, put it out and then I'd be like…[uhh] wait an hour then be like [uhh] wait another hour.

I: Okay okay so that's very interesting, you were like conscious even at the beginning that you were kind of like… okay don't smoke, the whole cigarette or like cigarette after cigarette after cigarette?

P: Right.

I: Do your friends and family smoke? Do the people around you smoke?

P: Yeah, my family, they smoke cigarettes.

I: Did that make it hard for you to stop or how did that affect you?

P: It didn't really make it hard for me to stop but just being around it, it kind of made me like [disgusted noise] because of the smell of them, just like ‘Oh, why would I even smoke that’ and then I’d always think like maybe I should then I was like ‘nah I can’t.’

I: Yeah it's funny when that switch flips where it's like stop smelling good- starts smelling bad it's weird that it can be so different the same thing and I can perceive it differently. What did you like about it when you did it? What do you do for you?

P: [It freed my stress and calmed my anger down.] That basically that's all it did…Like calms your nerves.

I: When you were quitting, what was that like emotionally for you?

P: It wasn't really too bad of an emotional, because obviously my baby’s more important than my lungs so… I just quit right then and there when I found out and then haven't really smoked since.

I: Do you have any cravings or anything?

P: Oh yeah cravings but I act on it, because I didn't want to mess anything else up.

(overlapping audios and cutting one another off)

I: When you're walking through your thought process of (unintelligible) what happens?

P: Normally, every time I would crave a cigarette because, like at the time my ex would smoke them I'll just stare at them and then I was just think no I can't really do that. Because like you want it so bad, but you can’t.

I: Yeah when you were going through harder times like, with your ex that was even more wanting to smoke, are you done by that point and been done thinking about it?

P: I mean, I did, but I was obviously still pregnant so I just went on walks or just like find something else to distract my mind off of it.

I: [How difficult was it on a scale?]

P: Hard-wise, it would have to be like a nine, but quitting wise would have happened like a one easy-wise.

I: Okay, did you- were you like ‘okay so I’m going to cut down’ or you said you like, how was your process, your thinking? How did you figure it out?

P: I'm not really sure, because I just like stopped right then and there with it.

I: [You didn’t cut down? You were like ‘I’m done’?]

P: yeah.

I: What do you think has helped you the most about that?

P: Probably… writing and walking like going out for walks and stuff because that calms your nerves and stuff.

I: Right because it's like… It sounds like you know… You have some stress, some like your nerves will be bad and then cigarettes will kind of calm that down so you needed a thing that did something similar. And it makes sense that writing and walking I mean that's, we know that those things do help. Did you talk to your doctor about quitting at all?

P: Not really that when they asked if I smoked I said yeah I used to, but stopped as soon as I found out.

I: Okay, so if there's anything that you wish, like bisexual young women knew about tobacco use, what would you kind of tell them?

P: I would tell them not to because, especially if you have asthma, it will definitely make your life harder. Because it ruins your lungs.

I: You feel that kind of stuff?

P: I mean, not really but some days it gets pretty hard to breathe due to having asthma.

I: Do you think that, so they call it, like the gray period when you don't know you're pregnant, but you're actually pregnant, do you think you like accidentally used during that period at all?

P: I yeah because I didn't find out until I was two months from it, so the first trimester I smoked through the whole thing.

I: [You didn’t know so once you knew you were like ‘oh okay’?]

P: Yeah. I found out June 17th and I was like oh I'm done right there. No more.

I: It's amazing that you could make that change like that. Very amazing. Have you read or how did you know it was bad for your baby?

P: Because I read on it, plus my mom smoked while she's pregnant with me and that's how I had asthma so. I didn't want to give him asthma because it sucks.

I: Yeah.

P: It can harm the lungs of newborn babies and seeing how I had complications with him it definitely would’ve affected him.

I: Yeah. It does sound like good forethought that you would need to quit. [And that’s what happened?] Did you really get sick or anything during your pregnancy? Was there anything physically that made you want to smoke or were you physically disgusted by them?

P: I was disgusted by it, like even the smell of it like if somebody smokes near me, I have to walk away because I'd like throw up.

I: What about now has that stayed the same since you’ve had your baby?

P: I mean no because my senses aren't as strong as they were but they've given me headaches if I smell it.

I: Do you feel like you know anything about being an LGBT Q person is connected like- how's it connected to using tobacco?

P: I'm not sure about my experience like I'm sure other people have like experience with that, but not for me no.

I: Do you talk about that stuff with your family or anything, or is it just like they don't know- they don't need to know?

P: I mean they know because I tell my mom everything so she obviously knows, but I'm not ashamed like if they have questions I'll obviously tell them.

I: Does your baby’s dad know?

P: Yeah he knows.

I: How do you think it's like affected, you know your life in general now to be bisexual?

P: Honestly, it hasn't really affected anything. Like growing up, obviously I was confused and stuff but then, as I got older, I was like well that's who I am.

I: You just kind of accept it like?

P: Yeah.

I: And do you feel like you've always been able to talk about it or was it like a process for you to come out and stuff?

P: I would talk about it, but then I was like well i'm not really sure, but as how I feel so. (audio failure- following statement may not be accurate) Like I shy away with it.

I: Do you feel like your peers are pretty accepting of that?

P: Some of them, yeah. Most of them, I mean they can have their opinions, but… they don't bother me.

I: So someone's not like they're judgmental about it, how would that affect your friendship with them?

P: I tell them ‘peace.’ (audio failure) Okay bye.

I: Yeah.

P: I don't let things bother me. At least I try not to.

I: [Is it hard for you to be around your friends when they smoke or is it a challenge for you?]

P: Not really. Because [they’re their own person and I’m my own person so]

I: [Being around it is not a trigger- -?] (voice lowers to unintelligible)

P: No.

I: How do you feel about [child] being around it?

P: He's not around it, if someone smokes I make them go outside or if we’re in a car I make them stop, get out and do whatever like I don't want him to be around it, especially being this little.

I: He's not around it?

P: No.

I: Okay, have you ever- like we call it co-use, so I know you haven't smoked weed in like four years but- did you ever use marijuana and tobacco at the same time?

P: No.

I: Why not?

P: I just never really thought about it after like getting high or whatever- I don’t know.

I: [Smoking weed never made you want like a cigarette or anything like that?]

P: (non-spoken disagreement)

I: Okay. Interesting. Okay, so these are going to be like the last section, and these are… they're kind of abstract, I think. So just do your best, and if you need clarification, let me know because I also am not super great at knowing how to frame these the best way.

And so we kind of talked about this a little bit, but what do you wish, like all bisexual girls knew specifically about pregnancy?

P: No matter what your nurses and doctors will be there for you, and if they don't support that, then you could obviously just get a new doctor and don't be shy about voicing your opinion on what they're doing.

I: [What you just said made me curious. Were there ever times where you had to advocate for yourself?]

P: (non verbal agreement)

I: And can you describe them? What was that like for you? Is that hard? Are you like I gotta do this for my baby?

P: It was hard like I have obviously had a couple of nurses who are totally rude so. I wasn't shy about it, I told like the head nurse or yeah she was the head nurse all about what they were doing and saying you know- all that.

I: What did they do? I want more information.

P: I'm not sure what she did, but I know I did have the nurses come back to my room.

I: What did they do to you? What was your interaction like- I'm sorry that happened to you.

P: They were basically belittling me because I was a first time mom like- obviously I know how to take care of children, I did that my whole life growing up. I just didn't want to like… hold my baby a certain way, because he was so tiny and I didn't want to drop him because he was literally like this big when he was born. He was so tiny. And I felt weird holding him, I felt weird changing him because he was so tiny. I'm not used to tiny babies. I’m used to the big chunker babies.

I: Yes, and what were they saying?

P: They were like ‘oh you're a mom you can do this, you can do that.’ I’m not just a first time mom- I’m just a first time mom for a pre-me. It's not easy.

I: What did you want them to say to you? What should they have done in that situation?

P: They should have at least told me what to do, and not to do- at least with a pre-me and at least give me a little more information about how to take care of pre-mes, but.

I: It sounds like you wanted them to educate you and they weren't doing that so you just talk to the head nurse?

P: yeah.

(they interrupt and talk over one another)

I: I’m sorry what did you say?

P: I said she was very helpful, she was the one that actually was there when I got my spinal tap done.

I: Was that painful?

P: Not really surprisingly. They shoved it in my back twice so. I don't know.

I: So she was kind of there with you through some tough things?

P: yeah.

I: Were there other times, where you felt like you had to really stand up for yourself or make it clear what your needs were?

P: I mean, I made it known that I was mom and what I wanted is what was going to happen- not what they wanted, so I feel like that's what some of the nurses were a little mean. Well I'm mom and I don't want him to wear the hat when he's in the incubator because he had a rash. You can’t do that to a baby like he's breaking out in a rash and is still continuing to put the hat on while he's in something very warm.

I: So you want it… yeah like you're saying ‘I’m mom This is my child and I'm in control of his medical care.’? And they were not- maybe respecting your wishes and making you say it multiple times?

P: They wouldn't really respect anything I would do because I don't know what the deal was- they were like- I don't understand, I guess, because I thought I had postpartum depression and I never had that so like I don’t know. They’d like belittle me on that, so like ‘ ah, she’s just depressed. It’s her first baby- blah blah blah’ I’m like ‘it’s my first baby- I’m anxious not depressed.’

I: yeah where did they come up with that?

P: Honestly I don’t know. They just- ‘damn she's depressed.’ I'm just like because, like I'm sore, I don't want to get out of bed doesn’t mean I’m depressed. I literally got cut open. I struggled to get in and out of bed.

I: Yeah. Let's just say that about yeah I just wonder what I would feel like if someone was saying- (cut off by P)

P: I kept saying I was like I'm not depressed I have anxiety. There's a difference, a huge difference there. I'm perfectly fine. I don't feel like harming myself or my baby, never would I ever do that, like- I don’t know.

I: That I have more questions about that. So, like obviously- kind of like you know you had [baby] kind of premature. I feel like a lot of moms, a lot of people that's a reason to be anxious like you know your baby’s health undetermined at the time, and I just did they like talk to you about that at all, or was it just like they decided, you were depressed and like did they ask you if you wanted help for your mental health stuff? What happened?

P: They never really asked me anything, they just said ‘oh you're gonna have him now’ literally when I went to the ultrasound they were like messing around- taking forever; and I'm like ‘what is taking so long?’ They did the ultrasound, they called Pittsburgh like what's happening? Turns out my fluids were extremely low so they didn't even tell me that I had to find that out, on my own, so I was sitting in the hospital room waiting. And they're like ‘oh we're gonna perform an emergency C section. I was like ‘what?’ because I was at the time I was only there with my ex and I was like well I want my mom to be here because she had four C sections so. She knows a little more about it and She finally came in and calmed me down about that, because I was obviously having anxiety and they just did that, and then I was it. They never asked me about Oh, are you depressed or you this, I told him I had anxiety, but they're like depression.

I: [Did they explain anything to you? Did they just tell you that there’s something we need to do and not tell you why or help you understand what was going on? What did that feel like for you?]

P: it's just- they never really told me anything, besides my fluids are low and about I just needed to have an emergency C section and then afterwards they like- during the C section they explained a little bit. [They gave] like important details like you’re going to feel tugging, and all this and that so… but afterwards they just ‘alright, you’re on your own.’

I: Do you think there's any kind of follow up care? Like mental or emotional [something else] that you would have liked?

P: I think like healing wise with like the surgery questions and concerns like obviously they would have to add to that, but I guess just whatever you’re thinking about in the long run, future wise like ‘will everything be back to normal like in their own position?’ I don’t know. I just get so nervous about stuff like that.

I: Yeah well, one thing that I've noticed when I have anxiety is that it's helpful for me when people explain it. Because it makes me feel better when I understand what is going on. That's one way that I think it's helpful. I'm also just wondering if, like you know what it would have been like for you, if you've been in the hospital and maybe they had done some kind of follow up care where instead of labeling you as depressed- maybe ask you how you were feeling?

P: Right, but they never really did that. They watched and observed me, which is weird. That's not what you do and then just label ‘oh, she’s depressed-’ that's wrong to do.

I: yeah what's the better way to like instead of judging you? What's the better alternative? What could they have done?

P: I mean they could have just pulled me aside and just talked to me that way it was just like... How do you feel about certain things?

I: How are you feeling now like you know your three months after the surgery like [child] is obviously growing?

P: I'm not really worried or anxious now seeing how I got the hang of it but certain things that he [can do] does scare me like- He gets a stuffy nose, because he has allergies, because I have allergies right now they're kicking my butt. But other than that I don't really get anxious. Besides what I think you said, congested that’s what makes me nervous.

I: Like it's just I think a lot of first moms, new moms like it seems pretty on brand and normal to like to have that first baby be like why can't you, why aren't you breathing perfectly or you know there's just like so much.

P: Right.

I: Yeah. So this is like a little bit of a curveball but what do you wish all your health care providers all your nurses, what do you wish they knew about bisexual women?

P: That they’re not weird.

I: Yeah.

P: And like some girls, when [they] say that they're bisexual the nurses just get like don’t try anything with me. Like no…that's not how it works.

I: Yeah it sounds like [there are a lot of assumptions,] and maybe they’re not helpful to have right away. This is the- well there's like you know I asked like if there's anything that I could have done better anything, but this is the last question of the interview, what do you wish all bisexual girls knew about marijuana use?

P: I wish they knew the pros and cons about it, because it can be scary because people can lace it and they could also pretend that you like you're getting the weed but you're really not you're getting spice, which is totally different so… I wish they would know that because especially if you're out public smoking, yet you definitely want to be in a comfortable environment because you never know what could happen.

I: So that you wish you knew they knew the pros and cons about it and, like that it can be laced with something and also like that the environment in which you use it matters.

P: Especially with the people you hang out with too because, like you would want that comfortable feeling.

I: Well, so before we end, is there anything that you wish I had asked kind of related to the questions that I didn't?

P: [You said everything the right way,] I should say, like and I feel like I answered them all correctly. I don't know how to word it.

I: Yeah there's like there's no technically right answer, but I feel like you're really helpful and I feel like that's like what right is in this situation. So is there anything that I could have done differently to make you feel more comfortable or like make the interview better?

P: I mean if people actually stuff I answer honestly like I don't try to sugarcoat it because I really have nothing to hide, because, like if I did it obviously going to be like ‘oh yeah I did it.’ I don't know. I'm like an open but closed book at the same time.

I: So do you have any questions for me, before I stop taking up all your time and pay that $50?

P: Not really at the moment, no.

I: Okay, if you think of anything you can contact my phone number, you can call or text me.

P: Right.
