# Supplementary material for: Experiences of postpartum mental health sequelae among black and biracial women during the COVID-19 pandemic
Source: BMC Pregnancy Childbirth. 2023 Sep 4;23:636. doi: 10.1186/s12884-023-05929-3 (PMC10478375; doi:10.1186/s12884-023-05929-3)
Supplement: Supplementary file 12 — Supplementary Material 12 [file 12884_2023_5929_MOESM12_ESM.docx]

**Supplemental File 1.9 Interview Transcript with Participant 5364**

I: How's it been for you to be pregnant so far? How's your pregnancy going?

P: Honestly it's been really good. I thought I was going to be way more sick for longer and I haven't. It's definitely been really good.

I: That's awesome. That's really great, so you were sick at the beginning and now you're feeling better?

P: I’m able to eat. I’m able to work. I’m able to do everything I need to do.

I: That's nice. I feel like it really- I've heard it like both ways like wonderful pregnancy experiences and then I heard just like people that are like this is not what I thought I was signing up for basically.

P: Yeah at first, you know, it definitely feels that way, but it definitely grows on you, if you're a person that can adapt to life changing stuff, it grows on you.

I: How's it been for your body? How does it feel to go through all those changes?

P: Honestly, really amazing. I love being able to feel my baby move.

I: Yeah I think that is amazing.

P: Yeah.

I: [So to just kind of jump into your healthcare experiences…] How do you feel like your trips to the doctor have gone so far?

P: Pretty good. It kind of took forever to find out the gender of my baby because I feel like they weren’t doing what they were supposed to. As far as I know, I'm not scheduled for any recent appointments. But I thought I was supposed to be going more frequently once I hit my second trimester and going into my third trimester. I'm pretty much waiting for someone to call me, and let me know when I can start scheduling appointments again. I can honestly say from a scale one to 10… four it’s pretty bad.

I: Yeah that's pretty bad so i'm hearing maybe like more communication and like what else is going on?

P: Yeah basically I just need more communication because, like I said it took me forever to find out the gender of my baby because they were just- they- I don't know. It's like they had everything else to do except for making sure I knew the gender of the baby.

I: Yeah. Was it hard to get an appointment? Or what was the-? (cut off by P)

P: (unintelligible) It was very difficult. [It was like- when I called Magee I had to be transferred to different places]. Every time I tried to tell them my doctors and all my nurses names that were taking care of me they were like oh like at first they couldn't find a [person] and then they find that [person] I'm like well if they're not. It was very frustrating.

I: That sounds frustrating, they should know who their doctors are probably.

P: Yeah.

I: How is your so it has Dr. [person] been your primary caregiver throughout the pregnancy or have you had different ones?

P: Yes.

I: How's your relationship with her? How do you feel like that is?

P: She's a very good nurse, I can, yes, is a very, very good nurse. And it's been a while since I've seen her because they haven't- I haven't been able to schedule with her, because they just they haven't been able to schedule me with her, but the experience that I did have with her in the beginning of my pregnancy was very great and amazing, and I would definitely suggest her for anybody else that’s having a baby.

I: Okay that's good to hear. It's unfortunate that it's not a smooth system but I'm glad that you have that relationship with her it sounds like.

P: Yeah.

I: [During any of your earliest appointments, did anyone ask you about your sexual identity or anything like that?]

P: No.

I: How do you feel about that?

P: Honestly I don't have too much to say on that. You know, obviously I'm a woman, so I don't- I don't really- I don't really have too much to say about that.

I: Do you do wish that they had talked to you about [your sexual attraction] and stuff? Is that something that you think they should be asking about?

P: I mean not really.

I: Tell me more. Why, why not?

P: Just because I mean personally myself that's not something that a person shouldn’t want to hear. Like ‘oh, are you sleeping with a male or a female?’ Basically that's just it.

I: Like you don't think they need to know that kind of information about you?

P: Correct.

I: Okay, if they were to ask you are there ways that you would prefer that they bring it up?

P: No, honestly I don't have any kind of preference on the way to bring it up, except for the instance I brought up.

I: [If they asked you, you would opt to not share with them?]

P: Yeah.

I: Okay. Have any doctors talked to you about that at all?

P: No.

I: Okay. Is there anything like in the environment that you felt was geared to like LGBT Q plus people?

P: No.

I: [Do you think that the doctor would change their view of you if they knew you were a bisexual?]

P: No.

I: Were there any resources that the doctor so far have given you, in general, that you thought were really helpful for your pregnancy?

P: Yes, they gave me some information on me applying for medical. I was able to get Wick through my nurses and that's pretty much it so yeah.

I: OK, so they were able to like you know- did you have to sign the sign up by yourself, or did they help you do that while you were there?

P: They helped me sign up for my wick but I had to do my own insurance.

I: Okay okay. Can you think of anything that might be helpful to like have as a resource for bisexual people that are pregnant?

P: Honestly, no.

I: Okay, let me see. [These are the last few questions before we go into the marijuana and tobacco questions. Do you think being bisexual effected your pregnancy in any way?]

P: No.

I: What about the opposite of that? So do you think be like… you know, being pregnant effective being bisexual at all? Does that make sense?

P: Oh no course not, I mean honestly when I think about it, I know a lot more women that adored kids than men, so not at all.

I: What do you mean? Can you explain that to me? I think that's really interesting.

P: I don't really know how to explain it, except for like I don't know like who wouldn't want to be a mother? I think like women are more open to becoming a mom than a dad [is willing to become a stepdad] if that makes any sense.

I: Yeah I hear that so it's like you know if, in terms of like partners that you might have you've found that women are more receptive to the idea of stepping into a parent role.

P: Yeah.

I: Yeah okay that's really interesting and I hadn't considered that that would you know be someone's experience, but it does make sense.

Okay so before we move on to the next section, which will be about marijuana- is there anything else that you think you might want to share with me about you know being Bisexual and receiving care for pregnancy? Even if that is just like that it doesn't matter or whatever.

P: I mean, I definitely think that has to matter. I mean being pregnant is not easy, but other than that, like, I mean I don't really… I don't feel like it doesn't matter, it definitely does matter and… honestly, I don't know, I wouldn’t suggest that anybody go to Magee for the beginning of their pregnancy I guess.

I: Okay. Do you wish you had a different like you know hospital resource that you could go to? Like what's your ideal care situation?

P: I mean I do have another hospital but it's just like I’m too far along in my pregnancy honestly it's like why really changed it and I did hear some good things about Magee. it's just more of their scheduling time like I feel like my experience, hopefully, as far as giving birth shouldn’t be bad- I’m going to pray for it. But yeah I really don't have too much to say except for what I already said.

I: Yeah and that's why we're asking because, like you know it shouldn't be hard to schedule now given all the technology and access and all that stuff you know? UPMC is so big and I feel like that should be a solvable problem.

P: Yeah.

I: Do you feel like you've been treated pretty well within that hospital system or not really?

P: I’m sorry, can you say that one more time?

I: Yeah. [This can be a pretty big frustration. Do you feel like you’ve been listened to by Magee?]

P: At times, yes. But honestly I would prefer not to go more into detail with that, if that’s fine.

I: Yeah that's totally fine. we don't just say you know, like we don't we won't share any of this with your doctor like we're not part of your like care team right.

P: Yeah.

I: So they won't-

P: I understand.

I: Yeah I just want you to know that we won't share. So the next questions are about- this is the marijuana section, and then the marijuana section is basically the same as the tobacco section like they're kind of like mirrors of each other. So there are different I guess tracks, is a good word I could use like, so there are questions for people that have tried marijuana or smoked marijuana but.

Like you know, quit when they found out they were pregnant. There's questions for people that smoked while they're pregnant and there's questions for people that have never even tried it once, so [have you used marijuana before? Have you smoked or eaten it or whatever?]

P: Yes.

I: Okay. So did you continue to use it during your pregnancy at all?

P: No well, like the first two months- like the first month because I really didn't know, but the second month to help it throughout the nausea but other than that, no.

I: Okay okay so that's like that's like I think valuable information so like you know you said to help with the nausea did that did it actually end up making you like helping you feel better?

P: Yes it did.

I: Okay. So before you were pregnant, and I think this question is really fun because it's just interesting to me to hear about people's experiences so, can you like, I don’t know, set the stage on the first time that you used marijuana like what that was like for you.

P: It was pretty regular.

I: It was pretty regular?

P: Yeah, I don't know how to explain it. It wasn't nothing like when I first tried it. Wasn't nothing too weird but it wasn't like a bad experience. I really don't know how to explain the feeling. It's like trying to explain a high filling which I wouldn't even know how to explain, I guess, I know I wouldn't say helium because helium gives me a headache. Yeah I wouldn't really know how to explain it.

I: Were you like, with your friends or were you alone? Were you with an older sibling or something like that?

P: I was with a(n) (older?) friend.

I: Okay. And, did you, you know actually, get high, the first time, or was it you had to use a few times before that happened?

P: No, I got high the first time.

I: Does it kind of relax you or does it make you have more energy?

P: Yeah I guess. Like I said it all depends. It can relax you. It can help with nausea and dizziness. It can help a lot, but I feel like if you're not around the right people, it can also affect your temper because, like- There are moments, where I didn't catch myself and it's like I went from a good mood to like if I went to work kind of buzz, and somebody says something I don't like and then it kind of- I can be mad all day.

I: So it depends?

P: Yeah.

I: It's not always the same?

P: Correct.

I: Okay, is there one like do you prefer, one of the effects over the other?

P: No.

I: Okay, Have you noticed- does it depend on how you smoke- like a bong and it's a particular kind of high versus like a blunt or is it just like you said more like situation [based]?

P: Only smoke blunts so I wouldn't really know how a bong feels. I honestly, if I smoked it already I feel like it would be the same feeling, to be honest, it wouldn't be like a higher feeling, a lower feeling or just anything (something unintelligible) you know?

I: Are there things about it that you don't like?

P: No, except for there is- depending on where you get it from some people have different reactions to it. I wouldn't say I was one of those people, but I have witnessed you know, some people have pretty, pretty intense freak outs.

I: Okay, so not necessarily something that's happened to you, but something that you have been around that.

P: Correct.

I: Did you think it was just too strong, or were you worried it was laced with something?

P: I’m sorry, can you say that one more time?

I: Yeah totally. Did you think it was just like a bad reaction or too strong, or were you concerned that it might have been laced with something else?

P: No, It was probably what was put in it.

I: Okay, so you do think it was like how to not just weed or something?

P: Correct.

I: So Are there things that make you…Like you know you mentioned earlier, like it helps with being sick and stuff so that there's like a reason why you might want to use, but are there other reasons that maybe make you want to smoke?

P: Stress.

I: Like relationship stress, job stress, any kind of stress?

P: Any kind of stress. I feel like all stress is (audio) to be (technical?).

*Note: Unsure of the final word in the sentence though it sounded like she said “technical.”

I: Does it just make [the stress] go away?

P: No it kind of just helped. It's like meditation. It helps me slow down and catch my breath.

I: Okay.

P: Just basically slow down.

I: Okay, is there anything since you've stopped using it, that you found that helps in that same way?

P: What do you mean?

I: I feel like you stopped smoking. You tried to stop smoking like- did you find anything else that helps you cope with stress?

P: Oh no I still definitely get mad-mad.

I: Okay, so you still have that reaction, so there’s nothing that’s replaced it?

P: Correct.

I: Okay. What was it like for you, when you realized you were pregnant and you were thinking about quitting?

P: [I didn’t really have to think about it. I want a healthy baby don’t I?]

I: Sure, but a lot of people, you know they want a healthy baby and they still smoke and I won't judge them for that. I think yeah that's kind of what we were interested in knowing like what-? Is quitting hard for people? Are there tricks that people use or is it just like-?

P: No.

I: Okay. So you don't think it was hard or there weren't any tricks that you used?

P: It wasn't hard for me at Ieast but I can't speak for everybody.

I: That wasn't hard for you? Just because you were worried about your baby? What do you think made it easy?

P: Just because I don't need to smoke.

I: Okay, so not having that like…Like addiction, maybe?

P: Correct. Addiction is a terrible thing, nobody should be addicted to anything.

I: Okay, do you think weed is addictive at all?

P: Of course, they can be addicted to the wrong people.

I: Okay. So do you think there's like anything related to being bisexual and like you know that relates to smoking marijuana at all for you?

P: No.

I: Okay. It's like the same last question basically in every section like- Do you think there's anything that like I didn't ask you about like marijuana use and sexual orientation and pregnancy, that you want to like share that you think is important that maybe we're missing?

P: No.

I: Is there anything that you think doctors should know about it?

P: No.

I: Have you ever tried cigarettes, cigars, vaping- that kind of stuff?

P: No.

I: So this will be a short section, why not?

P: Cause I don’t like the smell of it.

I: Okay, the smell is gross to you?

P: It is very disgusting.

I: Do you have people in your life that smoke around you and stuff like that?

P: Yeah.

I: Has that affected how you think about it?

P: No, I still don’t like it.

I: What do you do right now to deal with your stress?

P: I sleep. I walk away from situations that I know I should walk away from.

I: Okay. So you walk away when you can walk away and you sleep.

P: Pretty much. Yup.

I: Okay. So what about when you're feeling like sadness, like you know how there's a difference between feeling overwhelmed and stressed out and like being down. Do you cope differently with those kinds of feelings?

P: No but that's the thing- I try not to really let myself get sad so…yeah I don't need to think about [sadness].

I: Okay, so you just don't you don't really think about it, how do you do that?

P: Well, I think of the way [it’s going to affect my baby. Whatever I feel the baby feels.]

I: And the last one is really similar in this section. What about anger?

P: The same thing goes for anger and sadness.

I: So anything- so knowing that your baby is going to feel what you feel helps you…

P: maintain it yep.

I: Okay. What do you like, hoping that does, for your baby?

P: I don't know. My baby doesn't turn out like me. Make my baby a little- (cut off by I) I don't know. (unintelligible) nobody wants their baby sad or mad. That's what I'm pretty sure I meant to say. I don't know. It don't do nothing for me- I just know I don't want the baby feeling that.

I: Okay, so it's like… To me I'm just hearing like you're just already starting to protect and care for your baby.

P: Correct.

I: And this is like one way that you can correct and care for your baby?

P: Yes.

I: Okay, that makes sense that makes a lot of sense. I really understand that.

P: Thank you.

I: Yeah and I think a lot of people don't necessarily think about it that deeply where it's like know like the feelings that you feel like your stress hormones like your baby probably does get some of that.

P: Yeah.

I: Do you think like your sexual identity has had anything to do with like you know how you think about substance use?

P: No (softly)

I: Do you want me to repeat that one?

P: Yeah- no, I said no.

I: No? Sorry I didn't hear you. Do you think that they are two separate things?

P: Yeah, my bad. I’m in the bathroom.

I: Okay, so this is going to be the last section and then you can like also just like tell me anything I didn't ask about. Like you know I'll ask you if there's things that I can do better in the interview or questions that you would ask differently, but… So these are the perfect world questions, and I think they're like they're kind of abstracts, but I think they're really cool because- They just frame things a little differently so imagine that you're pregnant in a perfect world. So first like what do you wish, like Magee and all healthcare providers knew about like bisexual pregnant women?

P: Nothing, to be honest, because I mean there's nothing to really know. We’re regular just like everybody else.

I: Okay. What do you wish that all like bisexual women knew about pregnancy, before getting pregnant?

P: In the beginning, it kicks ass.

I: Okay, a little warning.

P: Basically, yes.

I: Okay, and then the last two, for this is what do you wish all you know LGBT Q plus women, bisexual women knew about marijuana use?

P: Honestly, nothing except for the effect that it could have on your child. Honestly I wouldn't tell anybody not to do it because I have done it before and I'm also not the type of person to encourage somebody to do it because you could spend money on better things.

I: Okay, so just tell them about the effects it has on your child but you're not going to judge anybody or tell anybody how to live their life?

P: Correct.

I: Correct, but it is expensive?

P: Yeah.

I: Okay, so the last one.

P: [If you don’t know the right people.]

*Note: this may be incorrect.

I: I guess you've got like an in, you know, you got to have like the right kind of friends.

P: Yeah.

I: Okay So what do you wish of like bisexual women knew about tobacco use?

P: I don't know because, like I said, I don't really smoke tobacco. I don't know what kind of effect it can have when you[‘re pregnant- probably cancer].

I: So that is it. Those are all my questions, but I just wanted to see if you had suggestions to make the interview better or suggestions that I might be able to change how I'm asking stuff so it makes more sense. So what do you want, what do you think I could do differently?

P: Honestly, nothing you very that you great this was the fastest I’ve ever gotten done with one.

I: Good, good I'm glad that it went well for you, I just was- yeah I don't want to like you know. Take your answers and try to drag them out more so I was just trying to move through the guide and take what you get.

P: I understand. Thank you so much for it.

I: Yeah that's fine, so I'm going to load your $50 payment. And I don't know just text me if you have any questions. I’m thinking about you and your baby and I hope everything goes well for the rest of your pregnancy.

P: Thank you so much.

I: Okay, have a good rest of your day, thank you.

P: You too bye.

I: bye.
