# Supplementary material for: Experiences of postpartum mental health sequelae among black and biracial women during the COVID-19 pandemic
Source: BMC Pregnancy Childbirth. 2023 Sep 4;23:636. doi: 10.1186/s12884-023-05929-3 (PMC10478375; doi:10.1186/s12884-023-05929-3)
Supplement: Supplementary file 13 — Supplementary Material 13 [file 12884_2023_5929_MOESM13_ESM.docx]

**Supplemental File 1.6 Interview Transcript with Participant 5323**

I: Okay, so how's your pregnancy going so far?

P: It’s going alright.

I: Have you been having nausea and stop or how have you been feeling?

P: Like the first…few weeks, that was it.

I: Gotcha, so starting into the meat of the interview, what are your thoughts about marijuana in general?

P: I don’t think marijuana is like a bad drug but it's- pregnant I don't think you should be smoking.

(both cut each other off and overlap the audio)

I: Can tell me more [about] what makes you say that?

P: well… What part about the pregnancy part or just (interrupted by I) general?

I: Yeah, either. (other phrase lost in audio overlap)

P: Well, the drug I don't just- I just don't think it's bad because I have smoked marijuana before and it wasn't something I got addicted to personally. It's just kind of something I wanted to do for the time being, and then, when I was pregnant, I was like well… probably shouldn't be smoking anymore. It’s probably not in the best interest of my baby, so I just stopped altogether.

And just smoking when you're pregnant I just think there's a risk factor that I personally wouldn't want to risk with my child- with the development with them breathing and just things I wouldn't want to deal with personally.

I: Why have you heard about those risks?

P: My doctor was talking to me about you know just the risk of smoking period and you shouldn't be taking any drugs when you are pregnant, when you're a baby is developing, or just period.

I: Did you tell your doctor when you were in your appointment that you had used it before?

P: Yes.

I: Did you feel comfortable talking to them about it.

P: Yes.

I: What was there anything they kind of did to help you feel comfortable?

P: Just the facts of knowing certain things about marijuana like- If I was smoking like… crack or something I would be more like I don't want to tell someone that but marijuana is like a recreational drug nowadays, I was very comfortable to talk about with most people at least. So I didn't feel like it was like a big thing.

I: Is that something they asked you about or did you bring it up at your appointment?

P: It’s something they asked me about.

I: And had other people in your life I kind of told you whether or not you should tell your doctor like is that something you kind of thought about going in?

P: What do you mean?

I: Like did you talk to any family members or friends about whether you should tell your doctor that you were using marijuana?

P: No.

I: No? Okay, and where else do you get information about whether or not it's risky to use marijuana during pregnancy?

P: I read about it a lot, when I found out I was pregnant. I was like well, you can but… it's really not recommended.

I: Did you read-? (interrupt one another) Sorry, go ahead.

P: It was on Google, some website talking about it. It talks about the risk of smoking period, the risk of smoking marijuana just saying you just shouldn't take any type of drug.

I: How do you decide when you are kind of reading information on Google or hearing from your doctor, how do you decide whether information is trustworthy?

P: I kind of just think about it, realistically. Like would- like- ‘Should I be doing this?’, like, I feel like this intuition like I shouldn't be doing it.

I: Alright going back a little bit, what are your thoughts about tobacco in general?

P: I don't think smoking tobacco is good for you. When you're pregnant or when you're not. I don't think it's the best thing to do, doesn't have a lot of… It doesn't have any health benefits for one. I just don't think that's the best thing to do, for your body.

I: Whatever you heard and makes it bad for your body?

P: When you read cigarettes, it says nicotine is addictive. And when you get addicted to things you start spending money on it. You start… Wanting it- just you don't really need it. It affects how you breathe… Your teeth… just things that I personally wouldn’t want.

I: Yeah specifically during pregnancy, what have you heard and what do you think?

P: Well, the same thing goes back with marijuana, but I think tobacco use would be worse than marijuana for the baby… effects that the development really that's what it does, like the breathing and effects all that, like… Your baby's going to breathe in what you breathe in, so if you breathe in tobacco all the time that's what they're going to breathe in is going to be hard for them to develop.

I: What do you think about using marijuana and tobacco during pregnancy, do you think that's worse than using just one more about the same?

P: I guess I would think that is worse.

I: Okay. Why is that?

P: Well they're both drugs and he shouldn't be smoking drugs.

I: And I do want to talk a little bit more about your doctor's appointment and stuff where you tell your doctor that you were using marijuana. How did you feel going into that conversation? Did you have worries about how they would react?

P: I was a little uneasy, but then I was kind of like well they're my doctor so… they should probably know.

I: What other people told you about talking to your doctor about using marijuana? Have you been told nothing? …Okay.

And what do you think kind of like, influenced you and your decision to talk to them?

P: Just feel like you should be as honest as possible with your doctor because they're just there to help you.

I: And how did they respond when you told them?

P: They were just like ‘oh okay.’ And that was it.

I: What and what also they tell you I guess about the effects of marijuana use during pregnancy and tobacco? You mentioned that a little bit.

P: They don't really talk, they don't really talk about… marijuana and tobacco in depth. But their basic thing is… if you are doing any of that you should stop or you should begin to stop so your baby can develop as best as it can.

I: And I guess I'm kind of wandering, the timeline a little bit so, when did you quit using?

P: I probably quit like… a couple days after I found out.

I: Okay. Tell me more about I guess like how long did you use it and what was it like for you?

P: Well, I like I was on and off so sometimes I did it- And like sometimes I didn’t like sometimes I will use it for a couple weeks, and then I just wouldn't for a couple weeks so kind of on and off and around that time it was on and off.

I: How old were you when you first started using it?

P: I was 15.

I: Okay, and what did you use it for like was it socially or was it for stress relief what?

P: Why-? Socially because I was young.

I: Gotcha. And how was quitting for you like, was it hard or was it easy?

P: It was easy.

I: Had you ever tried to quit before?

P: yeah.

I: What happened those times?

P: I quit. I quit for the time being, that I wanted to- just like a personal choice for me. Like if I wanted to stop for a while, then I did, and then, if I wanted to start again, then I did.

I: You said, using marijuana was like a social thing for you, has that affected you socially since she quit you know, since finding out you’re pregnant?

P: No.

I: No? Okay. Did you have support when you were quitting- like a support person?

P: I didn't really need support, but my boyfriend's here, and he quit with me so.

I: Okay yeah that's awesome. Is tobacco something you'd ever tried?

P: Tobacco is something I did try at one point in time, but would I go back to it? No.

I: Tell me more about that.

P: I don't smoke cigarettes, the only thing I was smoking was Black & Milds, I think I did that for like a month on and off before I found that I was pregnant, because I just- pretty much like got into it, when I was like well I think I'm going to quit smoking weed so I was like well, maybe this will help I don't think it does so. I stopped smoking blacks that was harder than not smoking marijuana because nicotine, I feel like is more addictive.

I: Tell me about the quitting process for nicotine.

P: I just feel like if me personally, if i'm going to quit smoking nicotine, I just have to quit cold Turkey like I can't buy anything more can't smoke anything more just going to be it.

I: Was at the same time you quit marijuana?

P: yeah.

I: Did you- were you ever kind of using those at the same time?

P: Yes.

I: What was that like?

P: I don't know, it's kind of weird… For me, because I was new to it.

I: How did you feel when you use some of…?

P: The nicotine for me didn't give me a high anything so it was kind of like. I was just smoking and then I just smoked weed.

I: Was there a pattern, to the way you use something like that you always use nicotine first and then weed?

P: No.

I: And was there a situation where you would pick one over the other? How did you decide what you want to use?

P: I think sometimes I wanted to smoke black and mild before the weed, but then, sometimes it was the weed, because weed makes me tired, but the nicotine did it so it was like well black and mild I feel better smoking, because it doesn’t make me tired.

I: Had you thought about quitting that before?

P: Yes.

I: You had? What was that like?

P: Thinking about quitting tobacco was harder than thinking about quitting marijuana. I feel like it's more addictive to smoke tobacco than it is to smoke marijuana for me personally.

I: Did you have any like side effects when you quit?

P: Just normal withdrawal symptoms like I would think about getting a black and mild and I'm like I can't because I'm pregnant and I don't think that's in my best interest.

I: Is that something you read online about? You read a lot online about too, in addition to marijuana? What did you read about online?

P: Basically along the same lines, like… smoking tobacco is like a big risk for your baby. If you're pregnant, know they can't breathe and it's things like that.

I: Is that something you talk to your doctor about too?

P: [affirmative sound]

I: How did that go?

P: It was the same thing with weed. ‘Well did you quit?’ and I was like yeah.

I: What information were you provided with at your appointment about marijuana and tobacco? You said they touched on it briefly, were you given pamphlets and stuff too? Or did anyone besides the doctor talk to you about it?

P: They didn’t- It might have said in a in the like paper they gave me in the beginning about just smoking like general things about smoking, but it wasn't anything like in depth, because I didn't say like I needed help or anything so they didn't… you know, go too in depth about it.

I: And how did you feel- I guess, same kind of same question asked about marijuana- how did you feel going into the conversation with your doctor to tell them about your tobacco use, what did you have worries there too?

P: Yeah.

I: Tell me about those.

P: Just smoking tobacco I just feel like that's not good for you in general. It really wasn't something I would want to talk about with anybody… especially my doctor but that's something your doctor needs to know.

I: Why is it important to you for your doctor to know what substances you might be using?

P: Just in case something goes wrong or something is off. That might be the reason why.

I: What makes you more comfortable- I mean less comfortable- talking about tobacco use, why do you think you are less comfortable?

P: Because I feel like tobacco is worse than marijuana- health benefit- health effects at least.

I: What health effects have you heard?

P: Well, it messes with your teeth, your skin… Your breathing in general. I'm sure there's other things, but I don’t - I'm not too on top with tobacco effects.

I: Yeah, what do you think doctors can do to help young women feel more comfortable talking to them about marijuana and tobacco use?

P: Basically, just let them know like they're not there to judge them or anything they're just they just want to know what's going on, just in case you know something does go wrong. And they might know the reason why, especially if you haven’t quit.

I: Yeah, did you feel like your doctor did that?

P: Yes.

I: Any other ideas for things they could do?

P: Doctors pretty much do a lot like even if you're having a hard time quitting and stuff like they'll help you in that, but you just have to say, like hey I'm going through this. I don't know what else to say other than they're just there to help they're not there to judge you.

I: Yeah, if you had had a hard time quitting do you think you would have felt comfortable reaching out to your doctor?

P: No.

I: No? Why’s that?

P: If I had a hard time… I think I would feel like I'm being judged. Because it's something I really should stop because I'm pregnant, but there's something I feel like I can't stop.

I: Yeah, so still running with this hypothetical were like say you would have a hard time finding something- if you didn't reach out to your doctor, what I guess like support or resources do you think you would feel comfortable using?

P: I probably reach out to my boyfriend and have him help me out through the process and I'm sure he would.

I: What do you think would help young pregnant women like yourself get more information about marijuana and tobacco use during pregnancy?

P: You can always talk to your doctor, number one. You can always look on trusted websites, you know not just regular people on reddit and…stuff. You can even talk to people you know, if hey have knowledge on it.

I: Yeah you mentioned trusted websites, not just using reddit: what's been your experience with like information from social media around this?

P: I never really looked on social media, because a lot of times it's like… Not the right information. So it’s just best to look on like… web MD, Mayo clinic, just websites like people that know what they're talking about not just random people.

I: Yeah. What kind of things, I guess, did you want to know about the effects of these things during pregnancy?

P: I kind of want to know more about the effects of marijuana in pregnancy than tobacco- ‘cause tobacco is pretty much like… You can probably just figure that out or you could really just think about it, and things like that.

I: Are you able to find everything that you want to know, or do you still have questions?

P: I don't really have questions because the bottom line is you just shouldn't be smoking.

I: Do you think it's different at all talking to me about this as like a researcher versus talking to your doctor?

P: Probably.

I: What’s the difference?

P: You just want to know information just… Just for other people. A doctor kind of just wants to know information about you for your health benefits and things like that keep up on you. But either way it's fine for me.

I: Yeah you think between a researcher and a doctor there's you know one person you're more comfortable sharing with, or you’re willing to share more?

P: I don't think so for me.

I: Okay, so under some circumstances marijuana has been made legal, for instance, like your medical marijuana legal in 2018, what do you think about that?

P: Like I said in the beginning, I don't think marijuana is a bad drug. I mean, as long as it's not like mixed with anything I don't think it's a bad drug, so for it to be legalized I don't think that that's a bad thing.

I: Tell me more about marijuana being mixed with things.

P: Like events mix with like a different drug like cocaine, or like it's mixed with like tobacco- if it's mixed with… there's different types of drugs that normally, weed would not be mixed with, I think that that's been.

I: Is that something you worried about?

P: Yeah.

I: What made you worry about that?

P: You hear about it a lot on social media and just in general, like about people lacing drugs and putting people in the hospital on changing their life basically just because they smoked.

I: What did you do to avoid a situation where you got like laced?

P: I always got it from someone I trusted, and so, when I actually personally knew.

I: How's the pandemic impacted your marijuana use or tobacco use?

P: I don't think it did, as I didn't. I didn't smoke tobacco during the pandemic and marijuana I didn't really smoke too much, I did sometimes sometimes I didn't.

I: Do you think it's affected your pregnancy at all?

P: No.

I: No? Okay. Let me look through my guide and see you, and I might be missing. Is there anything I haven't asked that you were hoping to talk about?

P: Not really.

I: Maybe talk a little bit more about your doctor's appointment and stuff… How would you want a doctor to respond after you told them about marijuana tobacco use? I know you said you're kind of just okay and moved on is that what you want, or would you want a different response?

P: If you quit and it's I don't feel like it's too much to talk about after that. Right okay well that's good that you quit and that's alright with me.

I: Had you quit before your doctor's appointment?

P: Yes.

I: Do they ask you more about that?

P: No.

I: Do you know anyone else has had an experience with marijuana or tobacco use during pregnancy?

P: I've known a few people.

I: What have you heard from them?

P: They didn’t tell me, but my friends were telling me like you know things they smoke weed even though they're pregnant, [I sat there and i’m like] ‘wow’.

I: How did that- I guess impact, how you felt going in?

P: It just made- it just always made me think like I'm not gonna do that.

I: Why do you think some people continue to use during pregnancy?

P: I feel like they're in the habit of doing it and they just don't want to quit, or it's hard for them to quit- it’s one or the other.

I: Why do you think it can be hard to quit?

P: You get so used to doing something it just becomes routine. So you just keep doing and keep doing it, and then you might reach a point where you're like I can't stop.

I: When we were talking about laced drugs and laced marijuana you'd mentioned how you see a lot about it on social media. I'm curious what else you've seen on social media specifically if you saw anything else on tobacco use during pregnancy?

P: Not really because most people think like well I’m not going to put this on the Internet so not really it's not really talked about a lot on social media this kind of like… Just something everybody knows that you should not be doing.

I: Have you kind of looked for information on social media or have you mostly stuff with more like mainstream websites?

P: Yeah, mainstream.

I: So the American college of OBGYNs does recommend that doctors ask about marijuana use and does want them to tell their patients to quit during pregnancy, what do you think about that?

P: I think that's what they should do as a doctor.

I: How do you think they should approach that?

P: Number one: ask if they're smoking marijuana and even if they are- if they aren't- they should still be like because a lot of people lie. They should still be like well you know if you are- if you were- it's good that you quit because this, that and the third can happen, it can affect your baby and…

Like when your baby's first developing like that's like a really fragile moment for them like the first trimester… is really fragile so for you to be smoking is not good. I feel like that should be explained, even if they stopped or if they didn’t stop.

I: Outside of doctors, what are other ways where young women can really find trustworthy information about marijuana use during pregnancy or tobacco use?

P: I'm sure there's always someone out there that did smoke marijuana or tobacco that could be like a spokesperson, even though that's a pretty sensitive topic to talk about. You can always talk to someone you know, like I said, if they've been through it, or if they know information about it.

I: Why is it important to seek out people who have had that same experience?

P: It's relatable and we're probably going to feel more comfortable talking to someone that's been through it versus a doctor. More than likely.

I: Is that something you tried to do?

P: No.

I: No? Okay.

I: Think that's pretty much all of my questions. Is there anything that I didn't ask that you would want to talk about?

P: No.

I: ‘No?’, okay. Let me just make sure. Okay yeah that's it, so I’ll go ahead and stop recording here.
