# Supplementary material for: Experiences of postpartum mental health sequelae among black and biracial women during the COVID-19 pandemic
Source: BMC Pregnancy Childbirth. 2023 Sep 4;23:636. doi: 10.1186/s12884-023-05929-3 (PMC10478375; doi:10.1186/s12884-023-05929-3)
Supplement: Supplementary file 14 — Supplementary Material 14 [file 12884_2023_5929_MOESM14_ESM.docx]

**Supplemental File 1.22 Interview Transcript with Participant 5348**

I: How’s your pregnancy going so far?

P: It's going okay. I’m a little nauseous but not puking. I’m having like stomach pains but it's not severe like I've been taking up tylenol because that's what they said I could take so I've been taking them and it’s been helping and I take the vitamins they gave me. So everything else is going good.

I: Have you been taking anything that's been really helping with the nausea?

P: I was supposed to get these vitamins to help you sleep and then the nausea and stuff but my (something) has never called me, so I have to give them a call to see if it's like in or whatever so once I get them that I should be good.

I: yeah well that's good it's going pretty well so far, how are you feeling emotionally about everything?

P: Mood swings are like really there. I’m like– sometimes I'm Okay, sometimes I'm really angry and sometimes I'm just like okay I'm still upset– like I don’t know what to do so it's like it's difficult.

I: I guess going into some of the big interview questions, what are your thoughts about marijuana use?

P: I don't have a problem with it because, like you know I guess cuz I was raised with it so like it doesn't really affect me…But, and like I used to do it, but then, when I found out I was pregnant, I was like okay I'm cutting everything off– like all the medications from my doctors, I'm trying to get like ‘what can I take?’ so I can stop smoking weed and stuff like that, so I can have a healthy pregnancy, but marijuana is okay with me. They like– if my bipolar pills don't help, the marijuana does because it keeps me calm, but I rather take my medications than smoke marijuana that’s just… (stops speaking)

I: What do you mean you were raised with it– around it?

18

00:01:59.190 --> 00:02:11.460

Sarah Stroud: I guess– I wasn’t raised by my family. The people that I hang around with like– I guess the area that I'm in everybody does it so it's like okay I’m hanging out with this one person, and then their friends do it too, my boyfriend does it, so it’s just like Okay, I guess I'm raised around it now.

I: What were your experiences with it?

P: My experience was… I don't know– I would say, like when I was really having a bad moment and, like my pills weren't working for me my…aunt's husband bought this marijuana pen thing from the marijuana store or something, and she let me hit it and then ever since then, like if my medication wasn't working I would take a puff and then I would start like okay I'm feeling better, so I wouldn't rely on it, like some people do to get high and stuff I rely on it, because it helps with like my bipolar issues and stuff like that.

I: How old were you when you first tried it?

P: I was 18, 19. I was old enough.

I: So since you've become pregnant and quit, what have you done to cope with some of those issues from your bipolar disorder like when your medications are not working?

P: I actually have been trying to do some yoga and like stuff that I can do while I’m pregnant because— I look on Google for stuff like that, so I've been doing yoga, stress relief stuff, breathing– basically, just watching TV just to keep my calm down. It's been working or if that doesn't work I play games like the piano game, and it keeps me busy.

I: What do you think about tobacco use in general?

P: I can't stand it. I don't like it. I can’t stand the smell. I don't like using it. I have never smoked it a day in my life. I don't plan on smoking, but like my boyfriend does, but like he does it away from me so that's kind of nice, but other than that I don't like it's just not my thing.

I: What are some good and bad things you've heard about it?

P: Well, I really never heard anything good about it, they always say don't ever do it because it gets you addicted. People have been dying from it– like the vapes or stuff like that, so I guess, I really never heard anything good about it. It’s all negative, but people do it because they've got used to it.

I: What do you think about using tobacco while pregnant?

P: I've seen people do it, but for me-wise, I could never do it cuz like…I don't want to mess up the baby and stuff like that so I don't know. Ever since I found out I was pregnant, I changed everything like how I'm living my life and what I'm doing better. That way I'm healthy, my baby's healthy and everything [ends up perfect].

I: Tell me more about some of the things you've changed like when you say you’ve changed everything.

P: I changed like… doing less like– I was always running in and out like doing other things hanging out with my friends, doing stuff like that, so I cut all that. I stopped smoking weed. I stopped drinking because I turned 21 last year and ever since then, I was like drinking and partying but ever since then I stopped so that way I wouldn't have health problems during pregnancy. So I would say that I changed a lot from now. Like now I'm like two months, I really haven’t drinked. I don't drink at all at the moment and I don’t smoke weed. I don't hang out my friends like i'll hang out with them, but I don’t go to the club or bar– stuff like that so it's– I changed a lot and I would say it's probably the better change cuz I like me now than I did before hanging out with the wrong crowd and stuff like that so it's really nice.

I: Since like marijuana and alcohol and stuff seems like it was a big social activity for you, has it affected your relationships at all since you've quit?

P: No, like my friends are like they're still there, but they mostly just do what they want, so I guess like say ‘okay i'm good’ like I don't really care, like my child's coming first so y'all just do what you want. We talk, they text and see how I am but we don't physically hang out, which is fine, because I can really care less about hanging out with them because I realized they weren't really friends, they were just people that would use me for money so reality, I never really lost anything I'm just gaining better. So I think it's actually a good thing that I'm pregnant because it helped me really realize what I was missing, like what I'm not missing and stuff like that that makes sense. So I think the pregnancy could have happened any sooner, because these friends are not friends if they don't have your back now, so it's really nice.

I: And what do you think about marijuana use during pregnancy?

P: That's not like tobacco. marijuana and I see people do it while they're pregnant too, and they say it's good– it's not going to harm the baby. Marijuana is good, helps with problems, not gonna lie, but, for me, on the other hand, I can never sit there and keep smoking and knowing that I’m having a baby because, like I don't know how my baby’s going to turn out from it, or you know stuff like that. I don’t want them to have down syndrome, or whatever happens. So I just stopped everything and just be healthy, but I think marijuana is good to do if you're doing it for the right reasons, and not like everybody else does around here– they do to get high, and you know forget about their problems then they and their problems will still be there, they don't want to fix them. But other than that… Right now marijuana is not good for me.

I: What do you think are the right reasons to use it?

P: Using marijuana is good for health problems– Like, for example, for my bipolar. Like anxiety, stress, stuff like that is good… anything from medical problems. Now other problems are not good like I just think– if you're just going to use it to get high and joke around then what's the point? You’re just getting yourself messed up at the end of the day, so I think that's what it’s good for, just medical problems.

I: You mentioned for both marijuana and tobacco that you know people who have you seen during pregnancy or you've heard of people who have used some during pregnancy, who are those people that you like, know or have heard of?

P: My old friends that used to be friends back in like 2016, 17 and she has a lot of kids and she and her boyfriend said she did– They all smoked marijuana so she was smoking too and then she would smoke cigarettes and stuff like that so from her like experience I guess that's how I learned like I don't want to do that with my kid because, like her kids are not… I don’t know how to say it… they're not… right because of what all she did like drink and stuff like that, so I learned from basically that so I guess that was a good experience like exploring what not to do, and she was the example of what not to do.

I: Tell me more about [quitting. Did you decide as soon as you found out that you were pregnant? How did you come to that decision?]

P: Well, I have been wanting a kid my whole life, and when I said I wanted a kid I was going to be committed to my kid. And I was going to stop doing everything that I'm doing now, and once I found out that I had two lines on my pregnancy test, I immediately called my doctor, and I say okay, I need to get checked, so I know for sure, like am I actually pregnant or is it a myth like because I know, sometimes pregnancy test can come back like acting goofy so I just wanted to know before I definitely do stop everything and when the doctor gave me the like okay it's green like you good, you're pregnant. I was like okay, I’m changing everything like this is what I've been wanting this my whole life, and I think I was wanting a kid because my past and my childhood was not good. And like I never had a family role model to actually show me. Having a kid of my own, it would be really good for me to have that experience that I never got to have, like, I just want the best for my kid.

I: What are some of the things you think will happen if you keep using marijauna during pregnancy?

P: I really don't know because, like I never had a kid before to actually find out. I don’t plan on finding out smoking, or whatever, but with people that I do know they sometimes their kids have problems or disabilities– stuff like that, but I don't know if it’s because the marijuana or because they were just doing other stuff with it like drinking and doing all these other drugs that are out there. So, like, I really honestly just marijuana but knowing [my] experience, I will not take the risk, and then they would have like miscarriage or something for it so like I just… I’m good on that.

I: What was [quitting actually like? Was it hard for you to quit? Was it easy?]

P: Drinking wasn’t hard because I'm not a big drinker like I'll drink light drinks like Margaritas but other than that I don't really drink hard stuff. Marijuana I just started back in 19, then I stopped. It wasn't like a daily thing for me. Then, when I hit 20 it was not a real big thing for me. 21 is when I was like okay I'm doing it like every day and then now at 22– And I was like my boyfriend does if he doesn't like all the time, so when he smoked I smoked but I can take one puff or two puffs then I’m good because I get high super quick, so no it wasn't a big problem for me quitting. I didn't have withdrawals from anything like that. It was ‘okay like I'm having a baby, game on. ‘ There was no question about it, so it was pretty easy for me. Now asking my boyfriend to stop is probably the hardest thing. He’d have [a hard go of it] because he grew up on it, since he was like a kid and then now he's like 23 and he still does it every day, so he would probably have a harder time. I'm fine.

I: What kind of support did you have while quitting?

P: I really had like… My family– they really didn't like me doing it, but they really didn't care because I'm older, so like it’s my choice but when I say I'm done– like my family was excited because, like they like me being high or whatever. Now my boyfriend like… I really know how he feels about it, like he says, like it's a good thing that you’re going to stop and stuff, but then it sucks because I used to smoke with him and like we used to like do little things together now it's different. I don’t smoke so it’s like he's smoking by himself, so like I guess he likes it, but then doesn't like it, but he understands why I had to stop so I guess it's easy… that it's not easy, it depends on the day. For me, I can care less because I rather have a sober mindset then have a messed up mindset and do something stupid that I’ll regret.

I: In terms of it, depending on the day, what are some of the, if there are any, like triggers that have made you like, want to use?

P: When I say, like [when I am really, really stressed out or in pain] and like tylenol is not helping or something, then I'm like ‘okay like just give me a puff’ and then I'm like ‘wait don't do that, don't listen to me’, you know don't give it to me. And then he doesn’t so it's like I figure out the problem by myself, without going to weed so I'm doing really good on it like I haven't smoked ever since I found out, I was pregnant and so I think i'm taking is tylenol and my vitamins, that’s the only thing I take so I'm doing good.

I: Does your doctor and healthcare provider talk to you about marijuana or tobacco use during your appointments?

P: Yes, they do say stuff and then they asked my boyfriend if he smokes and stuff and then he’ll be like yeah and then they'll be like okay well just go outside because the baby actually inhales it. It’s like secondhand smoke or something so he's been going outside the window or outside with it's not too cold outside go outside, but if it's cool he’ll go in the window, so (unintelligible) it's pretty fine– everything's fine. As long as I’m healthy that’s all that matters.

I: Do you feel comfortable talking to your doctor and telling them about that?

P: Yes, I mentioned, I like when I go to the doctors I tell them everything. I make sure I ask them questions like what should I do, am I doing it right, do I need to gain more weight, is my baby okay? Like I make sure I'm on top of everything, and then I write it down in my books when I'm eating what I'm going to eat, like the diet plans and exercises I do. Because like my goal is I don't want to be fat and I don't want to be skinny, but I don't want to be unhealthy. I'll gain weight if I know my baby's gonna be okay, because, like when I went to the doctors, they told me that I need to gain weight, which is fine. I'll gain weight, just to make sure my baby’s okay, but as soon as that baby comes out I gotta lose the weight so… Other than that, everything's good. I always make sure my doctor knows if I'm really sick or if I'm really in pain or something. And it's nice to have that UMPC app on my phone and stuff. I use that to text them if I can't make it to the office or something or to have questions I always make sure I could text them.

I: Did you have any worries like going into that conversation telling them that you'd used marijuana in the past or that your boyfriend does?

P: No because I feel like…it's my life at the end of the day, like if I wanted to use or not use it if you can't really judge me because, at the end of day… it's my decision and how I plan out to be as a mother, so when I am when I tell them something and then I get like that weird look or something and then I just be honest with them i'm like… you might have a problem with it, but I don't as long as I know my baby's healthy– no problems or anything, nothing's wrong with him or her then I'm okay. So like I have no worries about it, but maybe someone else does or whatever, but I see it, is my life my choice.

I: Tell me about the weird look you're describing. Did that actually happen, it was not like from a different encounter?

P: Well, nowadays when [a doctor says you have to lose weight or] you shouldn't be smoking marijuana or you know they don't believe in it, I feel like that's them that's not me so if I want to be 600 pounds or you know skinny as a toothpick. That's my decision if I want to smoke some weed or smoke a cigarette. That is my decision. You don't have the right, maybe you have an opinion but you don't have the right to tell me what I should do with my life. So when a doctor tells me that they'll always give you that look like a doctor look, I guess, I call it. And then I just think in my head like you can say anything you want, at the end of the day it doesn't affect how I will be as a mother, I can be the best mother and do marijuana, smoke cigarettes and you know we're big and heavy or skinny as a [tooth]pick– it’s just… They just be acting weird sometimes when they say you shouldn’t smoke, we should lose weight or gain weight.

I: How do you feel like you've developed that kind of attitude of letting it roll off your back even when someone is a [judgmental doctor or provider?]

P: I'm not gonna lie, it took a couple years because, like I said, my past was really bad growing up. I never had a mother figure, my father was never around. And I was really insecure about myself, getting bullied constantly and then I was 16 years old, my grandfather took me in and I barely went to hospitals, I took my medication, I got everything I need to take care of and then I started getting confidence, where I can dress in front of people, you know, start sticking up for myself and start learning to say the word no. So, ever since then, especially living out in [area] where I'm from and stuff we're learning from the hood and the white and black culture or whatever it really helped me like ‘okay, this is real life’, like this stuff's happening so I gotta like… grow up and you know start sticking up for myself, because if not, then you know I can either end up in a coffin or you know somewhere that's not going to be good for me because, like people are really cruel in this world so like and I don't want that, for my kid so you know, I think me learning now is going to be good for when I have the kid, because then I could teach the kid, this is what you need to do instead of you know, letting people push you down and talking bad about you because you're not cute enough or you have like nice clothes and stuff like that, so I think it's a really nice experience for me and like definitely going to be a good experience when I have my child.

I: Have you– from other people in your life or online, maybe, have you gotten messages or ideas about what you should and should not tell your doctor when you're pregnant?

P: No, like I never got stuff like that, like my family will always tell me like just be honest, you know tell them what's really going on, how you feel, and that's exactly what I do. And like I said, if they have a problem with it and that's on them. At the end of the day they’re the doctor that’s supposed to help me. They’re not supposed to judge me. So how I feel whatever I have to stay or whatever, I feel, or I don't like or dislike or whatever they should be okay with, even though they have the opinion that it's not okay.

I: Tell me a little bit more about… I guess not conversation how you felt when and how it could have gone… and what the doctor could have done better I guess?

P: Instead of like I think the doctor, any doctor, instead of judging for what people do, or what people look like… they should actually do what the doctor is supposed to do– that's what you go to school for and actually help the person, support the person, find like other doctors, they can go to and just instead of just giving them medication and say here take this twice a day and stuff like that actually do your job, and look into what’s actually going on, because me for example like I've been telling the doctor since day one, but I’ve been having stomach problems. And every time I live with doctors, they say I'm fine and everything's good. But I know, deep down, like something's wrong like I'm having real bad stomach pains, and they just never believe me like they'll do X rays and stuff but they never take it farther. So it's like I'm coming to you because I'm actually in pain like I feel like I'm getting stabbed. And you're just telling me I'm fine. Why can't you just find me another doctor that does that stuff instead of just putting me on a pill thing and saying that it’s going to work?

And I feel like that's what doctors don't understand like they're giving people all these medications.. Meanwhile, the medications that you're giving them are like getting people addicted to them, and that's what people are like, that's what doctors like are not getting because, like my pap goes to a doctor's every day because he has real bad health problems and every– he takes 30 pills night and morning and they're all different, and like they're just making it worse for him, instead of like giving him all these pills… Just let him live his life, you know before he ends up dead, instead of druging them with all the stuff. And I think that's what doctors need to actually realize like it's not about giving them a pill and calling it a day like they won't be back– it's about actually caring for the person to make sure they're not going to end up you know living in pain their whole life and then just die.

I: In a situation like yours, where you were specifically talking about marijuana use with a doctor, what can they do to show that they care and help you feel comfortable?

P: I would say that it’s not just about showing them that they care or (unknown), it’s just the fact is like if you're going to sit there and ask me my medical records if I do that stuff, don't have a smirk or say something ignorant just be like ‘okay, I understand,’ or like even when you asked me a question like do you think that's a good choice and if I say to be honest, I think it is because it helped me with my bipolar and stuff then you should be thankful, because obviously that's helped me way more than what your pills were doing for me. Your pills with this either, making me gain weight, dizziness, drowsiness or whatever the case may be, but then you've got marijuana over here that's actually god's leaf.

And it's doing things that your pills are not doing for me. It's helping me calm down and be more relaxed and stress free. yeah I get high off of it, but it's not that high where i'm going to get a ticket for driving or stuff like that. Then I think it's wrong because they're putting in alcohol with weed when in reality they're saying you can't hold a gun, while you're high or whatever. And when you're drinking, you can still hold a gun, meanwhile, when you're drinking you can't be on the road.

You know, being high has nothing to do with your choices, you can still know what the heck you're doing and what you're about to say or what's going on, but when you're drunk…people start taking advantage of you. they'll start doing things, they'll start acting weird, getting in car accidents, stuff like that, so the weed thing is how it's so bad in this world. it's really not. It actually helps people. Now alcohol does not help like it might help for a second– But if you like an alcoholic and drink 24 seven and then you want to go drive or do something stupid who they say if you're going to make it out or not. at least with marijuana you know you're going to be okay, at the end of the day. And that's just how I feel about that.

I: If you feel comfortable sharing, what are some of the ignorant things that have been said to you, particularly if you have any like in reference to using marijuana?

P: (unintelligible) You will get you get called stupid for using it. you're wasting your life. You need– you deserve better than smoking that garbage… stuff like that. It's basically from my family because, like my family is really confusing because, one second they'll hate it, the next second they love it so it's like you're bashing me because I'm using a god's leaf. But you're also supporting it so you're really confusing me more like do you want me to do it, you don't want me to do it and I guess that's an argument at the end of it all it's always an argument. So I will say all this just the basics.

I: Anything like that that you've heard from doctors?

P: No, they just really just get the look like that doctor look or they'll go like [mmhmm] or something like that– yeah something that kids do.

I: yeah I keep hearing people talk about this doctor look. Can you tell me anything more about that you haven't already?

P: I really that's really all I see is just that doctor look and then the like, once you give them your opinion or something– they will change the subject so it's like why even asked me if you're not going to actually sit there and listen to me. You're just wasting your time, my time for an answer that you wanted to know, but now you don't want to know.

I: So where do you get – if you did have questions, where do you get information about marijuana or tobacco, if you have questions on that?

P: Google. everything I need to know is on Google, even though Google is not always 100% right it's really the only thing I have to talk to because you go to one person they’ll say this, and then the next person will say that it's like Okay, who the heck do I believe so, if I'm going to believe anybody it's going to be Google or Siri, those the only two.

I: Are there certain sites that you always go to?

P: Actually I just type it in on the Google search bar and then whatever I see that looks interesting I'm going to click on it, I really don't do a lot of looking because, like it's just a lot of looking and lot of reading that I don’t have time for so I'll click on the top one or the second one. And then i'll read through it and i'm like Okay, you know, this is a good answer or, this is a bad one I'm not gonna listen to it, I go off by that, but if sometimes I'll go off by an image like cuz I'll click on images and it'll show me some things. I learned from there. Other than that those are the three things I do when I need to get answered.

I: What do you mean when you say like this is a good answer, this is a bad answer like, how do you know?

P: Because I also look at the reviews so like once I go down, and you see all the reviews then you're like if it's like five bad reviews and then you got like two or three good reviews i'm like okay that's just a bad answer there’s more bad reviews than good reviews and then, when I go on the next one they'll be like, ‘Oh, this is so good, this is what you should do’ and then there's more like you know i'm like Okay, let me just go off by that especially like if saying I’m looking up sample face routines for my acne or something I look at the reviews and then whatever review sounds better or if there's more good reviews i'll go off that one and 99% that one was the best one for me so that it's easier that way.

And I do the same thing like having a baby, like if i'm not talking to my doctors about it, I'm really going on, Google or asking Siri okay like…how should I hold the baby or how should I you know wrap it up and stuff like that and they'll either give me like videos or like pictures with quotes on it and I just read them and then I screenshot them or write them down in my book, and that way, I know, like Okay, this is what I need to do.

I: In regards to marijuana, if there were any kind of questions you had, have you been able to get all of your questions answered or there's still things that you like, want to know about it?

P: Think I have all my questions answered, the only real one that I would love to know is why is it such a big problem that people actually smoke marijuana. Because I don't see it harming nobody you know, I see alcohol harming more people than we harms anybody. So, like that's My big question when I type that in they will just give you, like all these positive things, but then you go on the news articles things on your phone or whatever, and it will say well marijuana's bad and it's doing this and that it's like… Okay, like i'm so lost I lost where i'm supposed to be at cuz am I supposed to listen to the President, and then the news, or am I supposed listen to everybody that's tried marijuana, especially for their kids that have cancer or autism and stuff like that, and if it's helping kids with autism, and all that other stuff I think if my kid had any problems with autism or had medical problems I would literally tell my doctor that marijuana would be the way to go for my kid cuz it helps me. So it helps all the other kids that have problems out there, I don't see what's so wrong with it not helping my kid.

I: You pretty much touched on this, but what do you think about the medical legalization of marijuana?

P: I think about how it's good. I think it's good, but then you have some people like my family that think it's bad so it's like should I support my family's decision or should I go on my own decision and believe what I truly believe it like good or bad and I finally realized it's time to stop listening to everybody else and doing what everybody else wants me to do and believe in my own self and if I had to answer that I would say that it should be allowed in the United States Pennsylvania, it should be allowed for anybody. You know, it should be like a stranger, a homeless person can walk into the weed department and say I need this because it's helping them, you know they then my cuz we everybody is selling on the streets, because it's cheaper. But that's only because you only live in the buildings with a card, but not nobody has that money like since covid started who's going to pull $250 out for a card that you have to renew every year, no one has time for that meanwhile, you can just go on the street and some guys selling it for like 10,15. Just so people are chill like I think everybody in this world will sit down and just say forget some medications that are not that important and just smoke a blunt or something, the world would probably have more peace and less shootings and less killing because they're all state I think that's just because when I know people that have smoked weed and they've been in jail and they have all these problems and they’ll hit their heads and stuff and then they smoke they’re calm in like two seconds. And then they take medication, it's like you have to wait two hours for the kick in, so I think smoking weed is better for everybody.

I: Do you know anyone who’s used it medically?

P: yes, my last partner that I did have– he had a card, and he was on disability and he loved the card because he loves smoking weed and stuff but the problem was upgrading the card or renewing it when it became out of date and stuff. He had trouble cuz he had to pay his bills. He wasn't getting enough on disability, so he had to ask somebody to give him 250 every year to pay for this card. And then he just gave up on his card and just bought it from the streets, because it saves more money and less time to drive all the way to [place] and stuff to get that. So Oh, and my aunt’s husband, he has the medical card too and he loves it, but he spends like 300, 400 dollars just to get stuff. And I just think that's outrageous like if you have the medical card and you're already spending 250 for that car, why do you have to spend four or $500 just for a leaf that you can get from the streets that God put on the earth that just makes no sense but I'm not protesting or anything so… that's their choice.

I: Thanks for sharing that with me, I was wondering kind of what your experience has been hearing about like the expense and stuff so it's very interesting. So you mentioned one of your unresolved questions is like why marijuana's talked about as being so bad and stuff– is that something you asked your doctor about we thought about asking them about?

P: See, I thought about it, but then I'm like if they're going to sit there and give me that look and then ask me the question and then don't listen to the question and change the subject– what's the point of me wasting my breath to sit there and say so, what is the reason why marijuana is so bad? Meanwhile, you got people spending 250 for a card to go to the store to get it, but you're sitting there saying that marijuana is bad for you or doesn't help but meanwhile the medications that we take… they help, but then you get withdrawals, and all that other extra stuff.

I: Did they provide you with any information about using marijuana or tobacco during pregnancy?

P: Yeah because I got a book. Actually I got a book upstairs and it says like what to do, or it tells you about the marijuana and stuff and then tobacco, so that came in handy and I'm like okay I'm glad I definitely stopped smoking. But other than not when I'm not pregnant and I'm not breastfeeding, then I'm going to start smoking again because I feel like it's better for me, but at the moment I'm so good on smoking. I'm not feeling it, I'll just wait until my time again.

I: In an ideal world, what do you think doctors or health care providers can do to help young women feel more comfortable, for instance, you like being more comfortable to ask that question? Or just even to tell them about marijuana use in the first place, like what can they do to help in the perfect world?

P: [If this was a perfect world, what they actually need to do is listen to the patient.] And let them explain like what actually helps them instead of you saying okay, this is what pill you're going to take, you know, like I think they just need to sit there, listening to us and understand where we're coming from instead of just giving us a medication that's going to either get us addicted to it or ain't gonna work for us and then we're sitting there struggling to figure out why is it not working or why we feel drowsiness or vomiting and stuff like that.

I: What can they do to show– what kinds of things have you seen them do, or what could they do to show that they are listening, understanding and trying to help you?

P: I feel like the only time they actually listen is when… it's literally about what they're giving us like their prescription they're giving us, but if it's about weed or something like about our weight, I feel like they listen enough because at the end of the day it's our fault, you know it's never just reality it's always the person's fault, because we're gaining weight or this is why we look like this because you're not on this diet. But half of these diets, like weight watchers and stuff the same thing they're shown on TV, the same thing I got in my fridge so what makes it any different?

Like that cake chocolate cake is literally what I can make at home and I'm still going to gain the same weight. So for me spending 500, 300 on a car to have free food delivered to me, which is nice free food delivered to me, instead of shopping for it but it's not helping my weight. It's just having free food delivered to me, so I feel like they need to stop thinking all these pills are working for us because some people don't even like to take pills. So what are you going to do just give them liquid stuff to take instead of a pill, and just open your eyes and see reality like okay marijuana is better for this person, because he is used to it, he likes it and makes them feel good you know that's what people need to start realizing.

I: Besides the doctor look what you've described what kinds of things today either do or say that show you they're not listening?

P: Besides look, I would say, definitely like I said earlier, like when they asked me simple questions like, why do you prefer smoking marijuana instead of the pills, and then you start telling them and then… they'll hear it, but then, once you’re done talking– they'll just change the subject so you want to know their feedback on it's like what the heck, why'd you ask that question, then, if you don't want to answer the question I'm giving or listen to what I'm saying to answer a question back. That’s just… doctors like I feel like you went to a school all them years to be a doctor, but then you get out and it's like you're not doing your what you learned all them years it's just like one ear and out the other you're just in it for a paycheck and that's what it looks like.

I: Thank you, this is all really important and useful. Thank you so much for sharing everything so far. So what do you think makes talking to me as a researcher different than talking to a doctor about marijuana and tobacco use?

P: The difference is like I can actually tell you’re listening and actually want to know the stuff you know with a doctor, they say it, but they don't want to hear it, or they don't want to listen to what the person has to say. It's just and we're facing is right and what you're going to start doing and that's not the case on a lot of certain things because like I said a lot of people get allergic to these medications or kill people because they're taking too much of them, or they have withdrawals off of them and doctors are not seeing that and it's like Okay, if you overdose or something it's our fault, because you're giving these medications, but if you overdose on it's technically not our fault, because the pills ain't working, but she won't, let us smoke weed or anything you know so we're going to keep taking these pills, and so they start working. And I think that's what doctors need to start realizing, so I think talking to you is totally different than talking to a doctor because you're actually listening, you're all in for it, you’ll hear what I have to say, but doctors could care less.

I: You know, besides yourself the pills not working us to you know who's had bad experiences with prescription medication?

P: My one friend that used to work with, they were taking pills, and they just weren't working for him so like… He stopped and then he just started getting worse and his mom moved him to marijuana and then he started, you know acting normal will not acting normal but acting right, I would say. Cuz like his pills were just making him feel weird, making him drowsy, and then my family member– my cousin actually he's supposed to take pills, but he's 15, him and my brother 15 and they have to take medication or something and they don't work for him, so they go off by smoking marijuana and everything thing, so I just feel like marijuana is a better way to does and some prescriptions.

I: How has the pandemic affected your marijuana usage?

P: No, it has not impacted me at all, like, I never have to rely on weed, I can say that for sure, like if I am broke or whatever, and I want to smoke, I'm not going to be the type of like okay I gotta go work on the corner to get some money or something. Weed is not my priority in life, yes it's good it helps me but I don't need it 24 seven day. Like I said I'm not the type person to use it for hi miss or fun and type person use it for my problems, so this pandemic- it sucks, don't get me wrong, it sucks so bad and like people are losing their jobs and stuff but I will never have to rely on some weed to feel like I'm okay or I'm happy because that's not going to make me happy. It's gonna make me broke, and disappointed because I'm broke. I don't have any money to support my child now or my family, and so I rely on the important stuff before I rely on marijuana or tobacco products or whatever so nope doesn't bother me at all.

I: Tell me more about the financial aspect.

P: Financially, I would say before like because I lived with my gram for like four years and now like– since I'm with somebody we met together for almost eight months and now I'm trying to learn how to pay bills on my own and stuff… And like I get a check that's [an amount] or something like that, but it's not enough to cover for my living situation now that I have a kid and stuff, so I'm really worried and struggling about fat because it's like I can barely take care of myself sometimes. How am I going to support my child, but I finally realized like now, when I have my kid and it's actually in front of me– all my money is going to my kid regardless. There will be food in my house, because I get EBT but I won't worry about getting my hair done, my nails done until my kid’s 18 and walking out my door and saying okay well this time, but you just let me go. So right now the bills are really rough like we're trying to find a place, because this place that we live in is like it's okay like it's family suitable but it's just that where we're living right now is not the best– like the people around here– I wouldn't like, I don't really like them. They're not my cup of tea. There are a lot of drug addicts around here like they don't just do weed, they do other stuff like coke and heroin and stuff like that so like I really don't my kid raised up in this area so that's why we're trying to find a place. So hopefully once I get my kid and stuff situated, we will have a better place, and then I give a call to social security and try to like let them know what's going on and that way I can get more money on my checks that we can support the family and stuff like that, pay my bills.

I: It seems like I guess like recently before pregnancy philosophy really been like that other things like your other needs took higher priority over marijuana was there a time where that wasn't the case and, like how did you develop that looks philosophy?

P: I, to be honest, my money just goes to anything. To be honest, I never had anything to look forward to, I didn;t have to pay bills, I didn’t have to do nothing so when I wanted something, that’s what my money went to. New clothes, hair, weed, stuff like that, but ever since I moved to my boyfriend’s I realized okay I can't spend my money on every Amazon or everything. I have to actually start saving, so it took me a while and then like around November ish I was like okay it's time for me to grow up like I got to actually grow up I want a kid I want this, you know I gotta do what I gotta do. So I started making these envelopes and putting money in it, every time I get paid and then like $100 but then it would not work out as well because a bill would be late due, because my man works, he gets good paychecks but his money goes to the rent and that's like $600 and then we have water, sewer, gas and electric so it's like Okay, all this money I'm saving has to go to a bill regardless, so I typically don't save that right now, no more. And it's just like messing a lot of things up and now with covid getting worse, apparently, a lot of people are losing their jobs or jobs are shutting down again so it's like… holy crap what am I going to do I'm about to have a kid here in August and I don't know what the heck I'm going to do, because if my man, just like loses his job or like covid hits or something bad for him, then i'm just going to be like struggling cuz I don't know what else to do.

I: Thank you for telling me all about that. What else do you think would help young pregnant women, like yourself, get more information about marijuana and tobacco during pregnancy?

P: I think just having someone to talk to like we have counselors or talk to. Are they helpful? Not all the time. Some counselors really don't care. You know they're just they're in it for the money and that's what this whole world has basically come to– they’re in it for the money anymore, it's not about. What you're actually going through, how we can help you. It's just like okay after your appointment I'm getting a 300- 400 dollar paycheck, so I think that's what everybody's worried about now just so they can survive. For me to sit down and talk to somebody to ask them okay like I'm having a kid, what should I do, what should I not do. It's really hard because it's like or do you actually care, are you actually going to listen, are you actually going to support me, are you going to help me like… what is it because i'm not gonna sit here waste my time and money and breath just for you to get a good paycheck if you're not gonna actually do your job. So personally, I rather just look on Google and find my answers, instead of talking to somebody else just for now.

I: What do you get the counselors through?

P: I get the counselors from my doctor. My doctor provided them for me, and it was at [some place in an area]. And everything was going good, but then I just stopped liking her, because every time I would talk to her, she would just make a joke about it, or something like that and I'm trying to be like lady I don’t have time for your jokes like I'm having serious problems so like either helped me or just say okay i'm good to go, because I don't have time for this. But regardless you're going to put me on more medication that i'm already taking so what's the point of this being I call you on the phone for all this. It's freezing cold out here, and you want me to come sit and there talk to you for an hour or two hours just to joke around with me when I'm not even joking, so I rather just do my research on the phone or talk to my boyfriend or something cuz it’s less time, less money and less stress.

I: What about things that don't exist necessarily like programs or something that like either researchers or doctors or social workers, etc, could create like do you have anything that you can think of that again and, like a perfect world could help get information about this out?

P: I think, like this whole world needs to make over, it just needs to do something better, and it actually needs to help people and, like understand where people are coming from, it's not all about the money. It's about how you are presenting yourself to people and how you're talking to people. These kids are growing up to be criminals shooting people because that's what they think that's normal. That's not normal. that's insanity that you're just ready for your deathbed to me that's what it feels like.

So if somebody can take their time out of their busy life schedules which they say they have to actually sit there and listen to a foster kid and listen to some adults, the world would probably be a better place because let's face it, these foster homes they're not doing nothing but either getting your the kid molested or murdered or just treated badly but that's not what it's supposed to be. It's supposed to be okay, I'm gonna help you find a good home. I'm going to take care of you. Knowing that it's with my money, and I know people only take foster kids in because you get paid for it that's what it's not about. it's about showing that you actually care for the kid and actually want to sit there and support it. It’s not about okay, I'm getting 6000, 4000 dollars because you're living here. That's not what it's about, it's about the kid getting good education, the kids getting a good mother and a father and the kids going to make sure that they have nothing to worry about in life now and day these kids just do whatever they pleased they’re cocky, they’re snobby, they're bullying other kids to kill themselves that's not right. God didn’t put you on this earth to treat people badly. God puts you on this earth, to give you a chance of life to bring another human being into life and just how you treat them and how you respect and all the other stuff involved with having a kid or dealing with adults, not just kids– anybody. Teachers are there to help the kids and learn how to do math, and all this stuff but if you're actually sitting at a desk now they don't teach you reality in the world, they're just teaching you the basics that are back in the 1980s and that's not the case anymore. Now you've got to teach them like Okay, when you wake up in the morning you've got to learn how to brush your teeth, you know get dressed and get on the school bus safely what you should do when some stranger approaches you. Stuff like that, not all this stuff– All this bullcrap that they're teaching now and just saying okay here's some homework in a book, a paper just do it. That's not teaching the kid that just shipping them to the next thing in life that's not helping them, so I think this world just needs a lot of help. And less drama and less killing.

I: Right, well, I do want to be respectful of your time. I know we're almost at the end here. Thank you so much for sharing everything so far. Is there anything I didn't ask about that you would want to add in regards to either obstetric care, marijuana, tobacco, anything?

P: No, I think that's good.

I: I’m going to go ahead and stop the recording.
