# Supplementary material for: Experiences of postpartum mental health sequelae among black and biracial women during the COVID-19 pandemic
Source: BMC Pregnancy Childbirth. 2023 Sep 4;23:636. doi: 10.1186/s12884-023-05929-3 (PMC10478375; doi:10.1186/s12884-023-05929-3)
Supplement: Supplementary file 15 — Supplementary Material 15 [file 12884_2023_5929_MOESM15_ESM.docx]

**Supplemental File 1.11 Interview Transcript with Participant 5382**

I: So sometimes in the interview i'm going to be using the term LGBTQ, is it Okay, if I just often refer to your sexuality as bisexual are you comfortable with that do you have a preferred term that isn't that?

P: I'm comfortable with it.

I: Okay cool- that's what I'll do, but before we kind of get into that I am just going to start with some, you know, easier questions and ask about… what it was like for you to be pregnant like you know what it was like for your body, emotionally?

P: Oh, my gosh it was so rough. It was very, very rough. My first, I worked a lot. Because it was obviously with my first child, and so I wanted to be prepared. And it was just really hard on my body, I didn’t- I just didn't enjoy being pregnant.

I: Yeah. That- I feel like there's also kind of a narrative around… You know, like what pregnancy is supposed to be like and pregnancy (overlap)... and all those things.

P: (lagging/overlapping audio) experience any of that.

I: Yeah, was that hard for you?

P: It was very hard, especially being a teen mom and I was constantly worrying about what other people were thinking… were gonna think.

I: Did you feel any kind of judgment coming from anyone about that?

P: Definitely from my parents. My dad was- My dad was quicker to accept it. My mom was not just because she was also she was my age when she had me and she just didn't want me to repeat anything that she did.

I: Yeah, how is she now about it?

P: Oh, she absolutely loves, she absolutely adores- I knew, she was going to come around because she loves kids but… I just wish her initial reaction was to put aside whatever she was feeling to be there for me.

I: Yeah… In your body, was this pregnancy different than your first one?

P: I didn't experience as much nausea as I did with my first. And because I didn't work I wasn't overworking my body so much, but it definitely was a lot harder, I think I don't know if it's because I had a girl, this time… But I would say it was just about the same- just some things were a little bit different.

I: Was there anything that you- what was your favorite thing about being pregnant? What was the absolute worst part of it for you?

P: My favorite thing about being pregnant was just knowing that I was growing a body, a little baby inside me, and then feeling the kicks and then getting to hear their heartbeat and seeing them on the ultrasound. But then everything else was just terrible.

I: How is your… doctor care during your pregnancy your health care when you say?

P: I would say it was pretty good… they definitely did make me feel really comfortable with whatever was wrong, and I could tell that they had my- they really, truly cared.

I: What made you know that they cared about you? What did they do that you could tell?

P: Sorry. Whatever questions I had they always answered it, and I didn't feel as if they were just trying to, you know, just get me out of the room, and they definitely made me feel at ease, especially because I had preeclampsia with my first.

I: Is there anything that you felt like was a bad experience with any of your doctors and nurses or during your delivery?

P: Oh no, especially during my Labor and delivery, the staff and my doctors both times were just amazing and I surprisingly, had a very easy labor and delivery both times. Which made up for the fact that I had a terrible, terrible experience during pregnancy.

I: Yeah, Tell me about your experience with them, what did they do during labor that you like?

P: What was nice was that most of the stuff that I had was around my age, and some of them had kids of their own so they made sure to like… include the fact that they can relate.

I: Yeah, and that is nice.

P: They were just like a lot easier to talk to… and approach with whatever I had if I had a problem.

I: Do you think like- they like- explain things to you and give you options or was it kind of-?

(they cut each other off)

P: Yeah.

I: Oh, sorry, go ahead.

P: yeah I would- they definitely…They thoroughly explained everything I was going on, like, no matter what they're doing, even if it was something small they made sure to tell me… just every step.

I: Was there anything that you like -you know, I'm just curious about this- was there anything that they asked you or did you have to make kind of like decisions where maybe you had to say no to some kind of procedure, or something or was it all pretty like basic?

P: It was all pretty basic. My first, they had me create a birth plan which I love. The second time I didn't create one because I knew this time I was sure of what I wanted, and I was like I was a lot more prepared and I knew that I was going to vocalize how I wanted things.

I: Did any of your- during your pregnancy, either one- did any of your health care providers, so that includes like medical assistants, social workers did any of them asked about your sexual orientation at all?

P: No.

I: Is that something that you wish they had asked about? Do you think they need to ask you about that? What are your thoughts?

P: I don't think they would have needed to… mean I guess when my fiance was in the room, it would have been nice if they asked like ‘who is this?’ That's what happened with my first, but my second they just kept saying supportive person; ‘who is your support person during this time?’, which was also nice.

I: So was the gender of your support person different during both pregnancies or was it the same?

P: The same.

I: So you prefered the support person terminology versus what was the first thing they said?

P: I mean he was the dad- he is that he had, but they just automatically assumed like- I think one asked if he was my brother, which made me a little comfortable.

I: If they did ask you about that, how do you think they should go about having that conversation with young pregnant women?

P: I think they should stick to how they went with my second you know just- ask like ‘who is your supportive person?’ and then they asked like what is his relation to me.

I: Did they ask you about gender anything?

P: No, they did not.

I: Do you feel like you would have been comfortable like telling your doctor that you were bisexual at that time?

P: Yeah I would have been comfortable.

I: What are the things that make you want to open up to them more?

P: When they genuinely listen to whatever I have to say and respond back like… With… what is the word- is it sympathy or empathy? Either one whatever's in there.

I: I think I could be wrong about this, I think sympathy is more like I feel bad for you and empathy is more like I understand how you feel.

P: Yeah, empathy.

I: yeah okay so like just listening to you, expressing empathy, you said that you like to that some of them were like kind of shared identities with you, so they had kids and were young and stuff like that. Were there anything that, like any doctors did or that you can remember that made you feel uncomfortable or they didn't kind of elicit you wanting to open up to them?

P: With my first during delivery when I was trying to push, I expressed that I wanted to try different birthing positions. And he sort of kept pressing that it would- it would be easier for him if I was on my back.

And I had an epidural in and I didn't I I heard a lot of stories about how, when you get an epidural and you and you're giving birth, on your back it messes up your back and I sort of caved in was like okay like let's just get this over with and then I ended up having a lot of back pain afterwards.

I: Yeah. I'm sorry that happened, I feel like… it's not ideal that… you'd have to kind of argue for what you want, during your birthing experience. Did you have that same doctor throughout the whole labor and delivery?

P: No. He came in, it was, I think he was just he was- he did see me during my pregnancy when at one of my appointments, but I didn't know that he was going to be the one to deliver the baby. And he wasn't in there, most of the room, well obviously and that's- I just didn't know who was going to be delivering my baby.

I: Yeah is that normal? I don't really know much about it. Is that how it always is?

P: Well, according to them, they- a doctor is supposed to see you numerous times throughout your OB appointments. I only saw him once. But with my second I did see her frequently and then she told me that she was going to be the one that was going to be delivering my daughter.

I: Is that how it- was that for you- did she actually deliver?

P: Yes, and I got to. She just waited till I was in whatever position I wanted to be in. And this time I was able to get into a position that I wanted to birth my daughter.

I: Do you feel like you prefer either one of those experiences like you know, having a doctor throughout the pregnancy and a delivery and then the other one was it just like you just had different doctors that every OB visit?

P: I had- I'd rather have a consistent doctor, but I did, there were a few times where my doctor wasn't available so I had to see someone else, but I was already used to them as well.

I: Yeah kind of like you have like you were able to establish- like it's easier for me to ask my doctor things and tell him what I want to do if I have an existing relationship with him or her.

P: (affirmative sound)

I: yeah and she let you get in your position that you want it, and all that stuff?

P: Yes.

I: Did you have as much back pain?

P: No.

I: That’s interesting. I'm sorry that he didn't listen to you.

P: Yeah…

I: Yeah. Okay, so were there any resources throughout your healthcare interactions during pregnancy, that you found particularly helpful?

P: I really- when I first started with- when I went to my very first appointment, when I was pregnant with my son they had me create a up you know up UPMC account for the APP which made it so much easier to keep track of all my test results, all my appointments, like everything that needed to be- everything I needed to know and to track of that I really, really like that. And then almost every appointment, they gave me like a new brochure, even with my second with- a whole bunch of resources for it, like everything.

I: [Do you like UPMC?]

P: Yeah.

I: (unintelligible)

P: And I like that it- It automatically creates an account for my children as well, once they enroll with a pediatrician.

I: Oh cool, is that link to your account?

P: yeah and it's linked to mine, so all I need to do is just use a fingerprint to get in.

I: Oh that's so convenient. Did you find yourself, using the brochures that they gave you are just mostly using them to put them in the (unknown word)?

P: I'm sorry?

I: Did you find that you would use the brochures that they gave you? Like all the resource pamphlets?

P: (affirmative sound)

I Was that help- with like i'm trying to think of the word- word like physical goods like car seats stuff like that or was it more help like accessing support groups or?

P: It was both. And then, with my first, I think, towards the end of my pregnancy…I guess- I one of my last appointments they gave me a whole packet full of gift cards.

I: That's really nice.

P: That was very, very nice. I haven't even gone through all of them, yet too.

I: Do they do that for everyone? That's so that's a good idea.

P: I think they do or I don't know if they do it for all the facilities, but I went to the one in West Mifflin.

I: This is a slightly- it's a little bit of a different question, but I think it's interesting. What was it like for you to be pregnant and identify as bisexual?

P: I never really put much thought into it. I did see a few… things online and it's been more like- sorry I’m just trying to figure out how to explain it.

I: Take your time.

P: I noticed that there's a lot of biphobia in society. If you are like a lot of people say and a lot of people in the LGBT community, they say that if you are bisexual but in your if you're with a man and especially get pregnant by man then you're not really- like you're not a part of the community, so that made me feel a little… what's the word? I want to say left out, but it didn't make me feel like there wasn't a lot of support.

I: Yes like- not straight enough not gay enough.

P: Yeah.

I: And then I feel like. It makes me think about what that means for my identity like ‘who does that, then who am I?’ kind of. Did being pregnant change or affect that in any way, since you were like physically? You know you can tell when you look at someone and tell they’re pregnant usually.

P: I kind of added like I kind of just got in my head like you know what this is my life- it's not anyone else's.

I: Where do you- Do you see- where do you see the stuff that you get online, is it like you know Twitter tiktaalik or something?

P: It's basically like every social media platform.

I: You talked about being like biphobia there, is there any have you found any Community there like you know with bisexual appreciation or acceptance?

P: I’d say yes, like it's very starting to get very normalized like the the topic of bisexuality.

I: Okay, so this is the last question for this chunk of the interview, is there any and it says, asking if there's anything important that you know I didn't ask about related to being Bisexual and pregnant that you want to share.

P: Can you repeat that again? I'm sorry.

I: Sure, no it's fine. The last question is just kind of to double check that you know, maybe I didn't ask something or maybe there's something that you want to share, about being Bisexual and pregnant that you know isn't covered in the interview.

P: Not really, no I don't have any questions.

I: So the next two parts are going to be like marijuana and tobacco. First marijuana and then we'll move on to tobacco. And it's totally okay if I'm wrong so correct me, have you ever tried marijuana?

P: No.

I: Okay. So then, it just changes the questions I'm going to ask you, so I wanted to make sure. Okay, why do you think you've never tried it?

P: Just because I, there are a lot of family members that are like heavy users and it just wasn't something that interested me just because of how they acted… and how they basically depended on it, and the same goes for alcohol as well.

I: It’s interesting to me that, you know, it seems like there are people that share similar experiences to you, where you know, maybe it's in the homes that we grew up in and that… kind of pushes you in the direction of not using and then there's people that have similar childhood experiences or family environments that then go on to kind of follow in those footsteps more. Yeah what were the kind of…I don't know how to put this- like the things that turned you off about it, that your family that you saw people do?

P: well… if like a family- like if I'm around a family member, and I can tell they're just in a really bad mood or their mood is just off, and I asked why, and they’re like ‘I haven't smoked in so long’ but like they smoke every day, so you know, like a two second break what they act like it was going to kill them.

(cut off) Oh sorry.

I: No go ahead, I interrupted you.

P: Or they just didn't have much motivation for anything because you know they just like I've seen like really bright family members who had a lot of things going for them- Even though a lot of people say that marijuana isn't bad… it's just like they depended on it so much that they'd rather just sit there and smoke.

And I've seen a lot of people either like gain a lot of weight because they're eating so much because I heard that it gives you the munchies or they lose a lot of weight because they just don't have the energy or they just don't want to eat anymore.

I: Yeah. So it's not- you’ve seen in the, you know, I think a lot of people say there's no negative side effects but you've been able to witness that it does. Yeah… Do you have friendships or like you know is your support person someone that is like, are you able to be around it socially with your friends?

P: When I was in high school, yes, I was, I was able to be around it now- it's- now it's just like whenever I do have people, whenever I am the person- (tends to child)

Sorry, whenever I'm the one throwing -like if I'm personally throwing an event or if I am putting together something or planning a day I will tell certain people to not show up under the influence or to not leave to go smoke.

I: How's that received? Are they okay with that?

P: Sometimes, sometimes no.

I: Is that just something you do for your own emotional well being or you know, for your kids or like?

P: Both, definitely both.

I: How do you think that you, you know I think a lot of people use I'm sure you know there's a lot of people- use marijuana to cope with stress, or when something is making them scared so how do you deal with stress?

P: When I wasn't pregnant, and I’m not pregnant anymore, I did CBD. It was, I did consume like the gummies or… the liquid, so you can just pour it into water. But it was never like a large amount, it was always something you know just to relax my body. But then also because I'm also not really into that as well- I enjoy cleaning. I like to take my stress out on cleaning.

I: What about when you are feeling sad?

P: I usually, when I'm sad I usually just put on a comfort show. And some comfort food or if it's something- You know, deeper I talked to my fiance about whatever's going on. And I also enjoy coloring to help.

I: What about anger?

P: Anger I usually…depending on the situation, I like if I'm angry with someone, I don't like to immediately talk about why I'm angry so because I know that- I'm not ready to talk about it, I don't want to escalate the issue. I go to a separate room and think about why am I angry, is it worth being angry over.

I: Yes, the kind of talking with yourself first?

P: Yeah talking with myself.

I: How do you and then would you share it if you realize that it's reasonable or?

P: yeah I when I realized that like if its reasonable or something stupid I do end up apologizing for if I did- end up snapping out on in the moment I will I will apologize for it, but then I will also apologize, you know for misunderstanding.

I: The next questions are short- This is really short, for you know people that you don't really use marijuana for much shorter or it's tobacco (unintelligible) do you?

P: No.

I: Okay, is there anything else? It's the same question as last time we end up kind of little segment and interview. Is there anything that we didn't talk about yet about marijuana and pregnancy, in particular, that you want to share?

P: Well, I heard that- I noticed this topic, like a lot through social media and then like hearing it from other people. A lot of moms have like- they talk to their doctor and they have to and they get the okay to smoke, so in order for them to eat during their pregnancy, because they're so nauseous. And then, I think the hospital checks the placenta and CPS gets involved if marijuana is- if there's a trace of it. And I'm sort of on the fence about all that.

I: What are you most troubled by or?

P: I see why moms do it because they need to eat. And it's like the last resort. But then I also see that… It also shows me that I wish there was just something that wasn't that [weed], that made things easier for women. Because a lot of the time, you know, you just hear ‘Oh eat crackers’ or ‘drink some water’, because then you're also limited to what medications you can take in order, in order to protect the baby. So I just wish there were a lot more options.

I: Yeah and like severe I forget was called like (unknown) or something like that. Just like severe nausea and vomiting throughout the whole pregnancy is actually… a serious like it can you know it can cause complications, it's a serious thing. Yeah what are your thoughts about you know, then CPS or CYF having to or you know the medical doctors involving CPS and stuff because of it? I don't think it's honestly that big of a deal for them to be involved, because even though there are negative side effects it's nothing like hardcore drugs. And if they get the okay from their doctor then I don't think social services like that now, because then they're going to be- Because then they're going to be involved in the child’s life.

I: Yeah, and then it’s kind of hypocritical for the doctor to kind of give the okay, basically, and then for that to happen.

P: yeah.

I: Well, thank you for thinking about that, because I think that part of it it's not interesting, but it is something that maybe doesn't get talked about enough, but is important to think about. The next part is about tobacco use like the exact same questions so… Have you tried tobacco or like little cigars, vaping anything like that?

P: No, I mean I did try vaping in high school just because, like everyone was doing it and I… went to an incredibly small school and it was predominantly white, so I wanted to fit in.

I: Did vaping or try vaping kind of help you feel like you were fitting in more?

P: A little, but then I realized that I saw how people were acting over it like they were spending so much money to just keep their addiction going.

I: It’s expensive. Did you like how it made you feel, did you not like the effect of it?

P: I did and that's why I didn't want to keep doing it.

I: How long do you think you tried it for before you're like okay?

P: Until I got out of high school.

I: Was it hard for you to stop?

P: Not really, no.

I: That seems good. Do you have family and friends that smoke cigarettes or vapor anything?

P: Yes, both my parents. They actually do both- they smoke and vape.

I: Are you able to be around that?

P: Yeah I am. I just when they’re smoking cigarettes, I don't because I don't like getting it on my clothes. I don't like smelling it. And then my dad is- when I live with my dad for… A good part of- I live in my dad- oh geez I can't talk. I lived with my dad my entire pregnancy, because we couldn't find an apartment yet.

And he was a heavy smoker. He smoked in the basement and it traveled through vents and it made all my clothes stink and I made all my son's clothes think before you even arrived and I had to constantly wash them and detox them and then it did create a problem because he depended heavily on them whenever- I voiced that I wanted him to smoke somewhere else.

I: Yeah. [Did you think that everywhere smelled like cigarettes?]

P: Yeah.

I: Because now it's so kind of offensive smelling to a lot of people. Was he able to like- did he respect your wishes, or was it something where he was like this is my house?

P: yeah. Yeah that's how he reacted. This is my house- ‘I do what I want.’ But then he also had me paying rent so I kind of that made me angrier because I was paying rent and I was young and trying to save up for my own place, trying to save up for a baby. And like that (audio) And I never really complained to him about it either even though I didn't like the fact, so I felt as if that's the least he could do is smoke somewhere else.

I: Have you guys been able to like… to makeup since then, or is it still?

P: yeah.

I: I think, so the tobacco section is very short, is there anything that you think about vaping or cigarettes during pregnancy that I didn't ask about that you want to share, about?

P: I just think to each their own like I can't really- no one will stop unless they truly want to stop. That's the way I look at it.So I don't, I like- me personally, like if I see someone smoking, while they're pregnant I'm not going to shame them for it because that's what they want to do.

I: And I'm not sure shaming people works as a behavior change thing…

P: Yeah.

I: Okay, so this is the last section, besides when I'll wrap up in the same way when I'll say to you, like you know, is there anything else you want to share but… these are kind of fun, they're like a little bit weird questions- but I like them so we're going to imagine that we're in your perfect world right?

So in this world, what do you wish all you know LGBT Q plus women knew about pregnancy?

P: I’m sorry?

I: How much do you want me to repeat? Do you want me to repeat the whole spiel or just the last part, what did you hear?

P: The whole thing, sorry.

I: Okay, so we are just pretending now that we're in your perfect like world, your version of an ideal planet Earth So what do you in this world wish that LGBT Q plus women knew about pregnancy?

P: I wish- everyone that…Like all like what really goes down with being pregnant, because everyone like society has glamorized and romanticized being pregnant and I feel like if a lot of people knew what really went down- they would know what they were getting into and then there's also a lot of other options like surrogacy I know I know that, like when money comes into play, like surrogacy, adoption you don't have to be pregnant, in order to have a family, but then also like I wish there was a lot more support into knowing that it is okay to not have children.

I: yeah. So there's more support around all of the possible you know realities.

P: Yes.

I: And, like, I just want to make sure I understood like- just like more honest representation?

P: (affirmative sound)

I: So what do you wish all healthcare providers knew about pregnant LGBT Q plus women?

P: I never really put much thought into that question, I really don't know. I’ve never really thought about that before.

I: yeah these are like kind of I think of them as the more abstract part of the interview, you know? Can either skip the one or we'll circle back to it, I feel like this was maybe a little easier, what do you wish all LGBT Q plus women knew about marijuana use?

P: Basically, everything that I said before, just… how people actually like how people depend on it and the negative side effects of it.

I: yeah, and then the same question for tobacco So what do you wish all you know, LGBTQ plus women knew about tobacco use?

P: I guess the same as the marijuana question.

I: When you were in high school and like you know you tried it because you wanted to fit in more- was that mostly about race, was your sexuality part of it?

P: It was mainly about race, yes.

I: Did it make you feel more accepted?

P: Not really. No, because they were still just going to look at me as, you know, that brown girl that vapes but she just happens to vape.

I: Is there anything that I didn't ask that you want to share about?

P: No.

I: Is there anything that I asked that I could have asked better or like I was offensive and I didn't know, I was offensive, anything like that, like that I could have done better?

P: Well, I feel like you explained everything thoroughly.

I: Okay, that is all my questions do you have any questions for me?

P: I do not.

I: Okay well what I'm going to do is put $50 on your card now. And I'm so glad that your delivery went well and that you have two healthy babies.

P: Thank you.

I: Yeah I just hope you have a good rest of your day and I really appreciate you taking the time and doing the interview with me.

P: Thank you, I enjoyed this interview.

*Note: The last bit of the interview was removed as it was not relevant to the main topic of this interview.
