# Supplementary material for: Experiences of postpartum mental health sequelae among black and biracial women during the COVID-19 pandemic
Source: BMC Pregnancy Childbirth. 2023 Sep 4;23:636. doi: 10.1186/s12884-023-05929-3 (PMC10478375; doi:10.1186/s12884-023-05929-3)
Supplement: Supplementary file 16 — Supplementary Material 16 [file 12884_2023_5929_MOESM16_ESM.docx]

**Supplemental File 1.18 Interview Transcript with Participant 5399**

I: Before I forgot I want to make sure that I’m using terminologies that are respectful to you. Can I use the term bisexual to refer to your identity?

P: Yea I feel comfortable with that.

I: OK. What was it like being pregnant?

P: I'm not so sick anymore now that I’m in the 3^rd^ trimester, I’m just dealing with like complications and problems, like I just found out about it-- placental abruption, which I have to go see specialist in two weeks. Also, at 20 weeks, I found out I had a two vessel cord.

I: I'm sorry

P: Oh no, it's OK, yeah, it's just a lot.

I: It is-- it is a lot, yeah, have you felt supported by the doctors that you've seen? Have you had good experiences or bad ones?

P: I've had a little bit of both. I've had good experiences for the most part when I go down to Pittsburgh Magee 'cause I was admitted there for about a week, because before I found out about the abruption, I was having bleeding, and this was about a month ago, but there was no sign of abruption then, but since I had bleeding, they were like, “well, we want you to get all growth ultrasounds every three weeks,” and plus the two vessel cord can stop the baby from growing properly… so that's another reason why they wanted me to do that, and I just went to Magee this week for my first one since I got out of the hospital. Now they're saying that there's signs of abruption, and they want me to go back down there for appointment, so that’s good. I feel like, at the hospital up here, UPMC Horizon, I've gone in for problems and I feel like they were just treating me like, “oh, you're a first time mom, so you don't really know what to worry about, you're just freaking yourself out,” but then something always ends up happening, and I'm like, “I don't think I'm freaking myself out,” like I think sometimes I get treated like this since this is my first kid, they just treat me like I'm over exaggerating or like I don't know what I'm talking about. But besides that, my personal doctor that I see… all my experiences with her have been really good.

I: yeah I would feel probably frustrated about having like a serious concern and then being met with not feeling like being taken seriously or listened to. Do you think it's just because you this is your first kid so they don't think that you really know what to expect? Do you think your age has anything to do with it?

P: Definitely age and being my first kid.

I: I’m glad that you have a doctor that you can at least feel like you have a relationship with. Are they going to keep you in the hospital? I know sometimes with abruption they want to do that sometimes.

P: Basically, depend on how my non stress test goes and whatever other tests they're gonna run when I go down there on the 13^th^… that's what's gonna decide if they're keeping me down there or not. Because last time when they found out about the abruption then, I was just having the bleeding, they also found that I also had a subchronic hematoma, which they said that could have been resolving, and that's what could have caused the bleeding. I haven't had any bleeding since. I'm still not now, but basically depending on how the testing goes, and considering that there is an abruption now, and there wasn't before, um, that's what's gonna make the decision of if they're gonna keep me down there or not.

I: how many weeks are you ?

P: I'll be 31 and two days.

I: OK yeah that is a lot of different things that are potentially going wrong or have gone wrong.

P: yeah.

I: So when you're at Magee do you feel like they are listening to you, they're taking you seriously? Or are you kind of just like another patient, you don't feel like they're really caring for you well? What's it been like there?

P: I feel like they have been really good about caring for me and making sure that I'm comfortable and explaining stuff to me so that I understand what's going on. Even when I have questions and stuff, I feel like they've been really good about that. I was definitely more comfortable at Magee than I was at Horizon.

I: Yea, I like when my doctor is like, “well here's why I'm giving you this medicine” like I wanna know what's going on instead of just being told to do something basically. So I'm going to change gears just a little bit: Has anyone in this whole process, like your doctor at either hospital, talk to you about your sexual orientation at all?

P: No.

I: do you feel comfortable sharing with that with them? Do you think it is any of their business? What do you think about that?

P: It really wouldn't bother me to share it with them, just because that is my doctor after all, so yeah, I wouldn't have a problem discussing that with my doctor or any doctor for that matter.

I: How do you think that they should bring that up if they're going to talk about it?

P: Just straightforward ask the question.

I: Just say like, “what's your sexual identity?” basically.

P: Yea.

I: do you think that the environment was at all friendly to LGBQ plus people?

P: Um, yea.

I: Do you think that are there any resources that you know as a bisexual person you'd want to receive at the doctors?

P: Not that I can think of, no.

I: And I like these questions but they're a little bit more abstract… so what was it like to be bisexual and pregnant?

P: Well, I I don't really know, just because I've been with my child's father the whole time, so I haven't really had the chance to experience it I guess, because I've been with a man this whole time.

I: do you think being pregnant affects your identity at all? Does it make people assume things about you or change how you feel about yourself?

P: I think it definitely makes people assume things about you. People would just see me and assume like “oh she likes men” or whatever, without actually knowing who I am and giving me the chance to say my identity as who I am. I feel like when a lot of people see a pregnant woman, they just assume she's straight.

I: I think that is totally true, and that is also supported by what we're finding… there's no research about people that are bisexual being pregnant, even though more people that are bisexual get pregnant between ages of 13 to 22… so exactly what you're saying is what we're seeing, yeah, that there's an assumption basically, and that assumption erases space. So before we go to the marijuana and tobacco questions, do you think there's anything else that you want to share about your sexual orientation and being pregnant?

P: No.

I: OK let's move along. Alright, so before we do the marijuana questions, I always like to just remind you that you know we don't share this information with anybody including doctors, family, all that stuff, OK? And I also like really like this question, I'm very curious about it… what was your first time using marijuana? Paint the scene for me.

P: I was with my best friend (for a long time, since we were young), and I remember her mom was out of town, and we came across her mom's little secret stash, so we took it upon ourselves to smoke, and it was just very funny-- we were just laughing and eating a lot, and it was I guess the average experience, I would say, but it was definitely enjoyable, but it wasn't something that I did on a regular basis. It was more of an every-once-in-a-while type thing in the beginning, but then as I got order, I started smoking more frequently. I feel like as I got older and more stress came about in my life and as I started working and stuff, that's when I find myself wanting to smoke way more often, and making a habit out of smoking everyday.

I: uh-huh yeah that leads me into my next question: So what role does marijuana play in your life right now today?

P: right now I haven't smoked in the past two months, and at first it was kind of hard, because it was just something that I always turn to after like a long day, like stressful situations and stuff, and something that could always calm me down and turn my day back around, and make me feel better. So, at first, stopping was difficult, and I was trying hard by telling myself that I'll be alright and just doing other stuff to distract myself from it, but now I'm just two months in, I'm just used to not smoking.

I: yeah it sounds like it can be like a tough transition.

P: yeah, I was having trouble sleeping at first, and I think that had a lot to do with it, but now everything feels like it's gone back to normal. I don't even really think about it, because it hasn't been something for me to do or something that I've done. I stopped doing it everyday, so it's not really a habit for me. I just figured out other stuff to do-- I definitely eat a lot more, in my personal opinion, than I did before.

I: yeah, is there anything else that you noticed that helps replace that for you?

P: Driving… like any type of activity honestly, even something as simple as driving; but then once I got put on bed rest, that's where it got hard again… because I wasn't really supposed to be doing much at all, so then it was figuring stuff out around the house, I just watched TV and movies, anything I can do around the house, cleaning.

I: what made you decide that you were going to quit?

P: Well the main reason I smoked in the beginning was because I was so sick-- it was hard, I couldn't even keep anything down, I threw up all day and all night, and I didn't even know my pregnancy was high risk yet. It was like so bad that I took off work, so my child’s father has just been working and taking care of all the bills and all that other stuff. He was fine with me taking off because of how sick I was. So that honestly helped me sleep; it would help me get an appetite to want to eat, 'cause it got to the point where I was throwing water up and was going hours and hours and even up to a day or two without eating, and that making me feel even worse and be even sicker than I was. So that helped a lot with being able to put food down and actually keep it down.

I: so it's helping with very basic important human functions like physical eating and sleeping.

P: yeah, 'cause I would throw up all day and all night.

I: yeah, so when you started feeling better, you were able to start thinking about quitting more?

P: yeah, once I started to feel better. When I first found out I was pregnant, I didn't want to smoke through my pregnancy to begin with; and from the beginning I was trying to stop, and I had dropped nicotine and everything, but then the sickness kicked in around six or seven weeks 'cause I found out pretty early, I was not even four weeks pregnant, my body knew immediately something was just off, and I tested positive very early, so I knew very early on. The sickness hit around about seven weeks and then by eight weeks I gave back in to smoking-- I just needed some type of relief, and that was the only thing helping me. The doctor wouldn't see me until I was like 12 or 13 weeks 'cause it just took me that long to get in there, so I couldn't even get any medication until then, and then they prescribed me Zofran, which a lot of doctors won't even prescribe until you get into your second trimester anyways, so then I was entering my second trimester, and I was just taking the Zofran as I needed it, and it worked, and that only lasted for about a month or two, 'cause as time went on, I started being sick less, so I needed the Zofran only sometimes, so I stopped taking that too, 'cause I didn't really wanna be on medication. I didn't want to do anything like that my whole pregnancy other than prenatals. I wanted to be as safe as possible. But there were some days where it was just like a habit, like some days I would still get my sick days, and the Zofran would give me headaches and constipate me really bad, so then I would substitute that for the marijuana. Finally two months ago, as I started getting closer, I was like, “I don't want it to be in my system when I have the baby and have to deal with anything for that,” I don't want to be in his system or any of that. I want to be clean. So finally I was just like, “I'm dropping it,” but now that I'm in my third trimester, it's rare that I even get sick and throw up. I'm more dealing with physical problems and the abruption and having to be on bed rest and stuff like that. My appetite is better than it's been this whole time. The only thing I was able to do is chicken but like now I can eat most things. Before, I couldn’t even do the smell of cooking-- I I'll walk in the house and my wok’s gonna be cooking or something, and I'll just instantly start gagging. But I definitely now, getting into my third trimester, I've definitely been trying a lot more and eating a lot more than I was before, 'cause before it was like, even with the help of smoking and getting an appetite, it was still a struggle to find something that I could get past the smell or taste of, because everything was just off to me.

I: yeah it would be so hard to be that nauseous all the time for so long

P: yeah

I: yeah, and to not be able to eat when you have to eat, not just for you, that was hard. So, when you were stopping smoking marijuana, did you do like cold turkey? Did you do a taper down or cut down things?

P: I just did cold turkey. I'm not really good with just cutting down. When I quit the nicotine, I had to do cold turkey also, 'cause otherwise I would just drag it on and be like, “oh, another day, oh, another couple more days,” so when it came to that, I was just cold turkey. If I don't just stop it altogether, I don't feel like I'm going to find excuses to keep doing it less and less or just push it farther, so I just did it cold turkey, 'cause I'd rather just be done with it, and not keep prolonging it. The more I prolonged, the less I would want to quit.

I: was there anything you can remember in particular after you cut it out cold Turkey that was triggering to you? That made you crave it? Were there any circumstances like that?

P: I did kind of crave it, especially because my boyfriend, he does still smoke, but he will just go in the bathroom or the other room and stuff like that, but that was probably like the first two weeks, and I'd be like, “oh you're gonna smoke, you're so lucky, I wish I could smoke,” and then I just got passed it, I just got over it. Now, he might smoke in front of me here and there, and smoke his little fake pen or whatever, and I don't even think of it now. Back when I see him do it, the first two weeks, it was kind of hard to still see him smoke even though he was trying to be respectful about it and hide it. It's just like I'm not stupid-- I know what you're going to do, which it wasn't a problem, I didn't expect him to not smoke either, but it was tricky at first. But after that first two weeks I just got passed it and I think the first two weeks was just hard for me 'cause I was having headaches and trouble sleeping and stuff like that, because I I smoked every day before that. I was a smoker for years, I really picked up with smoking by age 16 and then didn't get pregnant until I was 20, so for like those four years I smoked chronically everyday.

I: yeah it makes sense to me. I mean it's all actually amazing that it only lasted for two weeks, 'cause you know, that's four years, it makes sense that like, my body would take a little bit of time to adjust to how it is without a substance that I had been using for four years, right, that makes sense to me, even maybe people say that marijuana doesn't have withdrawal or anything, but I feel like there's little things.

P: I feel like it was more of a mental thing than anything and just being so used to doing it every day and like that being my go-to for any stressors in my life and then having to figure out other ways to cope with my stressors, and doing things differently rather than just going straight to smoking, 'cause like that became not an option anymore.

I: Did you think there's anything that like doctors did or could do to support you know a pregnant woman trying to quit smoking or eating weed?

P: Honestly, I don't know, maybe just be supportive about it, 'cause there's nothing else they really can do… you're the one that has to actually quit, so I guess just having a supportive doctor I would say.

I: did your doctor talk to you about it at all?

P: yeah, I did talk to her about it and when I told her I quit, she praised me for it, and she was like, “well that's really good,” and she actually made me feel good about it, like she didn’t make me feel bad for it or anything; she did ask me like why and stuff like that, why was I smoking and why did I decide to quit. And then she was just like, “well that's really good that you quit.”

I: Do you think anything about your sexual orientation has affected the way that you used marijuana throughout your life?

P: No.

I: So that is the that's the end of those questions, but I have one more… so you said when you stopped, were there certain things that you can recall that were stressors specifically to you that made you want to smoke?

P: just like a long day at work, or like, my boyfriend has a daughter already, so say she was having a bad day and it was a rough day with her, after getting her to bed, it would definitely be something to look forward to, going smoking, and then that would relax me for the night after. Just being high strung all day, but mainly being at work all day and then finally getting home and getting relaxed.

I: yeah, do you have anything else that you think is important for researchers or doctors to know about marijuana use during pregnancy and quitting during that time?

P: um, just that I really think, it can be really helpful when you are sick and dealing with stress and stuff, 'cause you shouldn't be under stress, but besides that, I feel like how quitting effects you just all depends on how much you smoke and for like how long you've been doing it or how often. I feel like everybody is different for sure when it comes to that, because it just depends on if you're an everyday smoker and how how long have you been smoking for, everyday or just every once in a while, I feel like that all has a big key to it.

I: do you think that your quitting tobacco made it harder or easier to also try to stop smoking?

P: I think definitely. I feel like quitting tobacco made me want to smoke marijuana more and smoking marijuana push me away from smoking nicotine, which was what I was most concerned about.

I: yeah, that was my next question: were you weighing which one you wanted to stop more?

P: yeah, definitely stopping the nicotine, just because I feel like personally I've heard more about problems from smoking nicotine while you're pregnant and stuff rather than smoking marijuana, so I'd rather do what I thought in my mind was less harmful.

I: yeah, like harm reduction basically.

P: yeah.

I: OK. So for the tobacco questions… so when did you quit? As soon as you found out you were pregnant? Did you smoke in that gray area, or maybe you were pregnant but you didn't know yet?

P: I was smoking before I found out I was pregnant, and then probably that first week I found out … I also had a vape, so me and my boyfriend agreed that once this vape runs out, I'm done, I'm just going to throw it away, but we found out so early and it was just so sudden, and it was a surprise to us, it was just a lot to take in, and I was still working at the time and under stress and just trying to balance… like am I gonna do this? This is gonna be hard. Smoking nicotine is another thing that I did since I was like 16, something that I've been doing for years, and something that I felt was way harder than quitting marijuana, because I definitely did experience withdrawals from stopping the nicotine. I had headaches and trouble sleeping with the marijuana, but nothing compared to the nicotine. Nicotine was what I would smoke throughout my day at work, so that is what made it even harder, it was my stress reliever all throughout the day. Like, yeah, I go home at the end of the day and I smoke marijuana or whatever, but nicotine was what I have to keep me pushing through the day, and I was just very irritable, but yeah it was before I found out and then in the first week or two that I had already knew, and then after that I was just done.

I: yeah, nicotine is such a hard thing to quit.

P: it is.

I: Have you quit nicotine before?

P: I had quit before. I had quit cigarettes like a year previously, but then I was nicotine free for a couple months, and then I got this job at beer distributor, driving to stores and stuff, and they have vapes there, and then that's where my vape situation started, so I went from one to the other, but what I will say was that I'd rather it have been like the vape (even though it's still nicotine) rather than an actual cigarette, because cigarettes containing tar and all types of terrible stuff and just the difference I felt in my body from quitting cigarettes, even though I ended up making the switch over to the vape, I definitely felt a lot better.

I: yeah, vape usually smells better too. You said you started smoking with cigarettes like when you were 16, not with a baby right?

P: yeah.

I: What was that like?

P: It was not fun. They made me feel crappy, but I still wanted one every day, and I don't know, cigarettes give you the head buzz. When I have cigarettes in the morning they would be so strong that if I was laying down in bed I would go back to sleep. I couldn't be like how some people wake up in the morning, first thing they want is a cigarette; I couldn’t do that, I had to get my day started, but then I still end up wanting a cigarette and they just made me feel sluggish. I was sick of smelling like them all the time and it was embarrassing, 'cause like I go to school and they would be like “oh you smell like cigarettes,” it is kind of gross, like my hair smells, all my clothes smell, my house smells, my room all that, and that is what really pushed me to want to quit, and then I went a couple months without nicotine at all, and then got introduced to the vape, and I was like, “Oh well let me try this, it's not as bad,” and then so I was probably like 18 when they get things started.

I: do you think vaping made it harder or easier to quit when you were pregnant?

P: Easier for me to quit cigarettes, for sure, because I feel like vaping is not as strong as a cigarette for one. What I miss the most about it and what was difficult was just the flavor. I had the nicotine addiction too, but what made me want to even start vaping was the flavor, it tasted good. That's what I liked about vaping, and it didn't leave no smell, I just looked at all the pros of it.

I: What helped you stop?

P: It was just cold turkey, so I just relied on smoking marijuana more, because it would help with the headaches and help me be able to sleep more, and I just tried to sleep it off as much as possible.

I: that makes sense, did your doctor talk to you about it, did they try to help you quit at all?

P: No, I didn't talk to my doctor about it.

I: is there anything that you think hospitals could do to support people who want to stop vaping or smoking when they get pregnant?

P: Just like be supportive about it, give advice, and let them know their options, and let them know what they can do, cannot do.

I: OK, do you think anything about your sexual orientation had to do with you starting smoking or how you thought about vaping?

P: No.

I: OK so when you use them together, marijuana and tobacco, do you use them at the same time, or were there times where you use one before another?

P: yeah

I: was that intentional and why would you do that?

P: It was intentional. I always smoke marijuana first and then I always want nicotine after, just kind of gives you like a buzz from smoking nicotine and it is more relaxing.

I: Yea, I hear you, that makes sense to me. OK, so our last question, in your perfect world, what do you wish that all doctors, nurses, healthcare providers knew about bisexual women?

P: um, honestly, I don't know, I don't really have an answer for that.

I: what about… what do you wish that all LGBTQ plus or bisexual women knew about being pregnant?

P: How difficult it can be… that's what took me off guard. I never thought that I could be so sick in my life until I got pregnant, and just like what can happen, what you need to deal with.

I: What about… what do you wish all LGBTQ women knew about tobacco or vaping or nicotine use?

P: how addictive it can be and how hard it can be to quit and what the withdrawals are like.

I: And then the last question is the same thing but for marijuana… So what do you wish you know all bisexual women knew about marijuana?

P: It can also be addictive, because you make a habit out of it, and it can be troublesome to quit, you have to find something else to replace it and fix all your stressors and stuff.

I: What are your plans after? Have you decided if you're going to smoke again, either cigarette or marijuana? After you have the baby.

P: I think about it often. I would like to try to stay away from nicotine, but I definitely see myself smoking marijuana after I have the baby.

I: Do you talk about that stuff with your partner or your friends or anything?

P: With my partner.

I: Has he been pretty supportive of whatever you need to happen for nicotine and marijuana?

P: yeah

I: do you think that there's anything that I should have asked differently or could have asked you another way? What do you think I could do better basically.

P: Nothing. I think everything was perfect.

I: I really appreciate you doing the interview. I was writing the whole time 'cause I felt like you were sharing such important things

P: yeah

I: Do you have any questions for me before I let you go and put that $50 on your card?

P: No I don't have any questions

I: OK I hope that the rest of your pregnancy goes as smoothly as you can, and if you want to let me know whenever you deliver the baby, I'm excited to hear from you.

P: OK thank you so much.

I: thank you, bye.
