# Supplementary material for: Experiences of postpartum mental health sequelae among black and biracial women during the COVID-19 pandemic
Source: BMC Pregnancy Childbirth. 2023 Sep 4;23:636. doi: 10.1186/s12884-023-05929-3 (PMC10478375; doi:10.1186/s12884-023-05929-3)
Supplement: Supplementary file 17 — Supplementary Material 17 [file 12884_2023_5929_MOESM17_ESM.docx]

**Supplemental File 1.15 Interview Transcript with Participant 5159**

I: Alright, so we kind of just like ease in a little bit and ask what it was like for you to be pregnant in general.

P: It was definitely stressful very, very stressful- the beginning of my pregnancy well first off I didn't know that I was pregnant. I was like six weeks, they said in the hospital because I went to the ER and I wasn't feeling good, and this is where I never felt this way, and you know I went there they told me yeah i'm pregnant so as the months went on everything was fine up until about when I started to do the- I forget what it's called but I had gestational diabetes, so I was going through that and, on top of that, I also had high blood pressure, so I was hypertension, as well, and on the verge of preeclampsia but thankfully, I never got it, I was very close to getting it, though.

But yeah it was just a little stressful but I did love it because I was you know, I was growing a human in me like it was so amazing like it's- It was like wow like I didn't- I knew people can get pregnant, but I was like you know being pregnant like it's really- it's really crazy because, like wow like i'm growing a whole human inside of me and i'm about to raise him, and you know to be another human being out here like- I loved it, but it was just like a lot of stress on my body and things like that.

I: yeah yeah so it was like you could appreciate that it was kind of like beautiful but, like the reality was also physically pretty hard.

P: Yes, very. I was by myself for most of my pregnancy so it was definitely hard.

I: yeah so like did you have did you eventually get like a support system or was it pretty much just you?

P: So what happened was uh my son's Father will he's my boyfriend now, but my my boyfriend or whatever he… He- We broke up so we've known each other for like a few years, and before I got pregnant and we were dating, messing around a little bit, but we got into this fight and he left and then like a few months later on my own I found out that I was pregnant. And he said he wanted to be around- no! He looked up my Facebook or something and saw that I was pregnant and he was like ‘that could be mine, that could be mine’ but, like, I mean it is yours, I wasn't just out here you know but… and then it just started a conversation from there. We were just together, and then we started living together and everything was fine like it's- it's been a little rough patch but we've been through a lot, so we are alright so we can get through it easily. For real, for real.

I: Yeah. So everything got better for you when you had some consistent support from him during this time?

P: Yes, way better.

I: it's hard to do it alone, it's a hard thing to do by yourself.

P: We broke up for a little while a few months ago, and I was (unknown) like it was definitely hard being a single mother because he wasn't around a lot.

I: yeah yeah. Do you think that, like when you were pregnant, do you think it affected how you thought about your sexuality at all?

P: No, I wouldn’t say that.

I: Do you feel like being bisexual is a different experience during pregnancy?

P: No I was with a woman, a little while ago, but no I didn't feel different or anything.

I: So when you were going through all the kind of like- gestational diabetes and hypertension and stuff that was hard for your body, how do you feel like your healthcare experiences were?

P: What do you mean by that?

I: Like you know when you were going to the doctor and getting all your care, did you like your experiences or did you wish that they would have been different in some ways?

P: Magee was definitely a little… they were stressing me out a lot. I heard it was not the best hospital to go to, but I want to go there because it's the only one that I can think of that they take care of women there, but I don't know- that hospital was kind of like crazy, so I didn't like going there, the only time I like going there was when I get ultrasounds and I see [my child] in my stomach like that was the only time I liked going there.

I: yeah yeah that I think that makes sense, I heard that, before that, like people like ultrasounds and getting to see the baby.

P: Oh yeah it was amazing, the first time I saw him I was so happy I cried like it was crazy.

I: What about the hospital, what about Magee was crazy to you? Was it how you were treated? What was it?

P: Yes. They just like they just didn't care, like they're very careless and you know they don't really - how do I want to say this, they don't really… You know, they just don't care. I just feel like they don't care about their patients as much as they should like yeah they might you know, take care of them, but not like they should and I definitely heard like a few people like being rude to patients and things like that, I’m just like you know we didn't choose that career for you like you know what I mean?

I: How are your doctors and stuff to you? Was it like that, with them too?

P: No. I actually had one doctor that I loved. She was amazing. She let me- she helped me out with programs and things like that, because at the time I was homeless, but yeah she was amazing.

I: [So she was someone that wasn’t just a doctor, but she was doing other things for you?]

P: Yes, for sure.

I: Okay, what kind of suggestions would you give that hospital system or doctors to [do right by you?]

P: I guess, just good customer service skills. You know, like they take care of people, so they may know how to talk to people, and you know calm people down because I was very upset when I found out that- Oh, I also found out that my son might have had cystic fibrosis. Thank God he didn’t but yeah like they were just explaining things to me, and you know I'm not the smartest so they definitely like- explained in detail because that's the only way I can learn, so I was actually like you know that was very nice of them taking care of me.

I: [So explaining things to you and making sure you knew what was going on with your baby?]

P: Yes.

I: Yeah I mean that makes so much sense to me.

P: Yes, it was great.

I: [Did any of your doctors ask about who you slept with or about your identity or anything?]

P: Yes, they have, they always asked me. Well, I don't know if they asked me if I had sex women but they definitely did ask me about my sexual partners.

I: Okay, what was that conversation like?

P: I'm not really shy when it comes to sexual orientation or anything like that I honestly like this is this is kind of weird to say, but I think I have a sex addiction, so it wasn't really like you know too bad.

I: So you're comfortable?

P: Yes, very comfortable.

I: [Were you okay with the way they handled you talking about that stuff?]

P: You mean like were they okay with it?

I: Did you feel accepted? Did you feel insulted by any of the questions that they were asking you?

P: No, I would say, they were nice questions.

I: [Why do you think it’s important for doctors to know your sexual orientation and partners and stuff like that?]

P: I'm not really sure, I mean… I'm sorry. I'm not really sure.

I: No that's Okay, I mean there's no right or wrong answers here like it's literally what you think so.

P: Okay okay that's why I'm like I know that I just feel so guilty or something like to think that I'm saying something so wrong.

I: yeah there's no right or wrong, like it's just your you know, like your opinions and like what you experienced and stuff like that.

P: ok

I: So yeah if you don't if you, and if you don't think that it's in poor and that's you know, a legit answer too. What can okay so like this is kind of like hypothetical but i'm wondering like you know if a doctor is asking someone like about their sexual partners about the orientation like how could they make them feel comfortable? How would you want them to bring that up to you?

P: Probably sit me down and ask me, you know as me a few questions and in detail things like that, because that's, the only way I'll really like understand and learn and change it, because like I said i'm not the smartest I definitely need to be dumbed down a little bit.

I: See you i'm going to take the time to explain things to you, and that includes like sexual orientation and partners and stuff like that?

P: Yes.

I: Yes, okay okay before we like skip over to or move over to like the marijuana questions i'm just want to ask like is there anything else you want to share, about like sexual orientation and being pregnant or receiving health care?

P: No.

I: Okay, so we're going to move to marijuana questions, and this is what I always remind people like this is totally confidential, so do not feel like you know you have to answer the questions, but we do keep everything private so I like to start with just hearing about like the first you know, like take me into your memory of like the first time you tried marijuana.

P: It was definitely a trip. I was with my oldest sister and my parents were gone, and I rolled up a little joint and it was like… I'm sorry my memory is so bad like I always try to sit here and remember things and I never can. It was different. I was feeling like my ears were ringing like it felt good, though, like it was very like something like I could do this, all the time type of thing. And I did.

I: And then you did, yeah, that was kind of my next question so like what role did marijuana play like throughout your life up unitl now?

P: I am very depressed and I got diagnosed with bipolar disorder a few years back. It's really just like helping me out a lot with it like I would smoke- I'm going to be honest here, you know, I would smoke a blunt, and it would make me feel 10 times better and then my whole mood will change.

I: I really appreciate your honesty and I'm interested in knowing what shifted for you in your mood? What did it do?

P: It would make me like- it's hard to explain, it's like it just makes you very happy. It just I don’t know- it makes me very happy makes me feel better like something like that it's hard to say.

I: [Are there any times when you specifically crave it?]

P: I wouldn't say I crave it, but more so, like there are times, where I'd be like I need it, I need it, I need it type of thing.

I: What kind of like triggers that?

P: Stress.

I: any specific situations?

P: No honestly like I said I'm bipolar so a lot of things set me off easily but probably I could probably name one time. Like when I live with my parents they're a very toxic household and when we would fight, I would just wait until later and just go out, smoke a blunt and that would be really nice.

I: yeah yeah. That makes sense to me. when you like what kind of so when you were pregnant did that change your relationship to marijuana at all, like me, you want it more or less?

P: I wouldn’t say that it made me want (marijuana) more or less. I didn’t smoke, when I was pregnant, though it was definitely I know I would say, I did a few times, but it was definitely hard like a weed is not bad so like it's not- it's a really good like It grows out of the ground, you know, like it's a very good type of thing, but you know it's only a handful of times where I've done it because I was nauseous or you know stressed out because it helps with nausea too.

I: yeah. yeah was that during the Gray period where you like didn't know you were pregnant or was it more just like after you found out you're pregnant you kind of tried to stop smoking?

P: yeah I did try and stop that addiction is very hard, I will, so I yeah it's it was very hard, especially when I was doing it by myself and you know, all I wanted was to just chill out and I couldn't even do it because you know, I was at home (trails off into being inaudible). I was about to cuss, I’m sorry.

I: A lot you can cost as much as you want. I seriously have the worst mouth ever. It takes so much concentration for me not to cuss but I'm getting paid- this is my job, you can cuss as much as you want.

P: Oh great yeah because I can't yeah me too. I cuss like a sailor- it’s bad.

I: Me too. Feel free, it is not, it is very hard to offend me to, or to like weird me out about anything.

P: Great, that's great.

I: yeah yeah So do you feel like you said something that I thought was important like you said that addiction was hard. Did you feel like when you were trying to quit you felt like you had become addicted to it?

P: Yes, I've been- I've been smoking for a little while so it definitely was like yeah it's like addiction is definitely a little difficult, I felt upset and angry and things like that.

I: Okay, so when you quit [you had negative emotions to deal with?]

P: yeah it's like- a metaphor would be like when you try to put your phone down and like you know if you're a teenager you know how some teenagers are with their phones nowadays it's like it's like if you were to put your phone down and just try not to be on it, but you know you know people are texting, you know people are online, and you know well that's for people that you know are addicted to our phones because it's a lot it's common. So I would compare it to that it's like trying to pick up your phone you know, like it's definitely difficult.

I: [Yeah, did you have to change who you were around and all that stuff?]

P: Yes, because everybody I knew smoked weed. It was definitely hard because I’m like oh I can't do it, I can't be here.

I: What helps you cope with that?

P: I'm sorry, say that again.

I: What helps you cope with that when you could when you didn't use it?

P: I just thought of my son, the whole time. It was just like you know what I’m doing I'm doing this for my son and stuff like that, like it was definitely like good… I don’t know it's hard to explain, I'm sorry.

I: No you're doing great. you're doing great so it's just you know, thinking of the baby helped you. Did you pick up any coping mechanisms during that time?

P: I would take walks and stuff like that. Try to go hang out with my sister's kids. I was- she has five kids so I was over there a lot and I'm very close with them. I love them so much, but I will be over there and I don't know I kind of just coped with it honestly.

I: Do you feel like being bisexual has any effect on like your marijuana use like you know how some like you can be discriminated against, for your sexual orientation, do you think like any experiences like that changed how you use weed?

P: Not at all.

I: Do you think there's any like relation between the two or?

P: No, I think, honestly I don't think it would be a problem at all.

I: Okay, so the last question is well just for the marijuana section. How do you think pregnant women can be supported to quit using like either by doctors or by you know their friends?

P: If you have support in your corner, I think anybody can stop doing this or stop doing that or they can do this, or can do that. I think, as long as you've got like some type of support and that one person like that's all you need that one person that you know sit here say you know what we got this I’mma support you and we got this.

I: So just that?

P: Yeah

I: Not feeling alone about it?

P: Yeah.

I: Okay let's move to the tobacco questions. All right, so it's the same, it's a really similar format so just take me back to that first time you use either cigarettes, vaping whenever you use that first time?

P: I've been smoking cigarettes for almost four years now. My ex boyfriend actually got me on them and I don't know at first I didn't like it at all like I smoked a cigarette and it was Okay, but I really didn't like it, so I put it out and then like a week or two later I tried it again and ever since then, it was just like it's a bad addiction, it just calms me down, it helps me stay calm and you know- it's just also a routine for me so I've tried it before, but since I got into a routine where oh i'll wake up and smoke a cigarette or oh every hour I'll smoke a cigarette. You know, things like that, but I just- you know, it's very hard to deal with addictions, especially with cigarettes.

I: yeah. What was it like when you tried quitting?

P: Very, very hard. I actually when was- I barely smoked during my pregnancy I'm not gonna lie I did smoke a few but not heavy. I knew it was bad, but I'm being honest, but it was definitely like it's very hard, because when your body depends on something like you can't have it like it just makes me (cry or fight?) you know? Wow, like I really needed to depend on it for happiness. It is very hard to stop doing that because I messed up or I fucked up and I just, you know, just kept going, but I did stop smoking cigarettes for about four months, when I was pregnant. I didn't touch a cigarette but like pregnancy was very stressful for me, so I definitely smoked one and it calmed me down so quick.

I: So it was like that stress relief and not having that stress really made it really hard to quit?

P: Yes.

I: I got it. I was a smoker for like 10 years.

P: Oh, good for you that you got off, but it's so hard, I definitely don't want to keep going.

I: yeah it's real tough but no judgment from me, for it it's really hard it's tough. It’s super addictive.

P: very. And I didn’t know until like because my parents, they both they've been smoking, since they were younger, you know so being in a household like that, like I'm not blaming them obviously my own woman, you know I made the decision, but you know, being in an environment like that is definitely difficult not to smoke.

I: [Just like you said it was hard for you to quit with weed because the people around you smoked it, did something like that happen with cigarettes too?]

P: Yes, it was very difficult.

I: yeah are there, so you know you said it calms you down it kind of relaxes you, which I totally relate to… are there things that it does to you that you don’t like?

P: weed?

I: tobacco.

P: Oh I'm sorry, the only thing, yes I i'm To be honest, I hate cigarettes and they're so disgusting but if I say that, but then I'll smoke another cigarette like I don't get it. It's very hard, but that's just how addiction is. I know it's gonna kill me. I know it's probably killing my lungs right now for as long as I’ve been smoking but I don't know it's very hard to get off of it.

I: yeah. Are there times where you want to, or you just like you know, whenever I want to get off at all I'll try?

P: All the time. I want to get off it all the time, but it’s so hard.

I: yeah. When you were pregnant, did you stop wanting them as much because you said you were pretty much able to cut down a lot? did you stop wanting them or were you like white knuckling them the whole time?

P: yeah I didn't like them, just the people I was living with- they (unintelligible) But I shouldn’t say that. No I- definitely… I'm so sorry I just lost my train of thought.

I: That's okay I’m just asking like what was it like when you were pregnant with cigarettes like would you think about it?

P: I didn’t want to do it at all, because I knew it was hurting my baby. But I was like I said my whole pregnancy was stressful so I definitely smoked a cigarette here and there.

I: yeah and did you find it relieving when you could smoke?

P: Yes, very.

I: What made you worry about not doing it? Why were you concerned?

P: For my baby I didn't want to hurt him or anything like that.

I: So you know just being bisexual has that affected, how you smoke, or you know when you started smoking being bisexual have anything to do with that for you?

P: No.

I: What do you think makes some people kind of smoke- and other people, you know not interested in it?

P: I’m sorry, say that one more time.

I: What do you think makes you or me or like people that you know smoke cigarettes- what makes those people different from people that maybe don't smoke cigarettes?

P: [Being] healthier, I would say. Being able to breathe better I'd have a little bit more money, because I know for me cigarettes are very expensive so whenever I, like you know, buy them and try to make them last a little longer, but it's definitely healthier. You know, not as much like anger, because when you stop smoking like I’m sure you know, I was just gonna be like I would be so angry, because you know nicotine just helps you calm down so without it's like ‘oh I don't know what to do.’

I: yeah yeah. So what do you think can help people like you, that were smoking when they're pregnant, but cutting down trying to quit, how can we help people like that?

P: I’m not really sure I felt like, in my opinion, I feel like you can't help people, especially if they don't want help. Things like that.

I: So it's more about whether or not they want help?

P: Yes, like if you I feel like, if you want the help you know you will go and figure it out, that's how I've been like all my life, so I definitely sat here and said, you know I'm gonna figure this stuff out all by myself.

I: I'm wondering how you know you were talking about both cigarettes and marijuana how like you know stress plays a part in it, so i'm wondering like- you know what it would be like if people had if you had less stress in your life during that time, do you feel like that would have made it a better environment for you to want to quit?

P: Yes, yes.

I: Let me show you okay so did you use like we're also in i'm interested in like did you ever use them together like marijuana and tobacco together?

P: Yes, all the time.

I: Okay, why is that what is? Tell me about that.

P: I just like when you- when I smoke a blunt, it would make me want to smoke a cigarette after because it boosts it. It like- makes the high better. It just makes you… I don't know and plus like since I'm addicted to cigarettes, it was kind of like Oh well, you know I said this now it's time for me to go smoke a cigarette you know things like that or I would just crave a cigarette.

I: Like how you were talking about the routine.

P: Yes, that's really what it is like I wouldn't be able to stop probably if I wasn't in a routine because I'm going to be honest, I barely have self control for myself.

I: yeah yeah I think that makes sense it's like you know i'm going to smoke a cigarette smoke a blunt because it's just like…

P: You know, like yeah like this is just what I do like every day, every time like and it feels good.

I: yeah yeah. Did you ever do that, while you were pregnant, that you can remember, and did it feel the same or was it different?

P: No, I definitely didn't do that when I was pregnant.

I: You were trying not to?

P: Yes, it was very hard, because I didn't want to put too much, I was already putting it in my body. I didn't want to put too much like back to back in there.

I: yeah yeah. Okay, we are gonna roll into the last like and these questions, please let me know if you need me to like to explain, or like to say them differently they're kind of abstract a little bit maybe is the best way to say it. So we're going to go to your perfect world, so like, whatever your perfect world is and in this world like what do you wish all bisexual women knew about pregnancy?

P: I will tell them that it's not easy. It's a battle every- I wouldn't say that it’s battle, but it's like a battle for me every day- mental health wise if you are very like it just takes a big toll on your body, and they should know that like it's not easy being pregnant like and then, once the baby becomes like it's even worse, like you got to deal with a newborn and you know they require attention 24/seven. So it's more so, like you know if you don't have patience, if you can’t give your child attention all the time and are working all the time, you know I just don't think people should have kids.

I: Okay, so what I'm hearing is like you want them to know the reality.

P: Yes. Exactly what happens.

I: yeah do you feel like that's what you knew before you were pregnant or did you have to like- what did you think it was going to be like?

P: My opinion on the pregnancy was completely different than what I thought I thought it was going to be a piece of cake- walk in the park but after everything I found out that could be wrong, like, I was also stressed out the whole time because I was afraid that I was going to lose my baby.

I: yeah that's so tough.

P: It definitely was. I cried almost every day like I just don't want to lose him, like yeah.

I: yeah do you think that, like you know and it's okay, this is too personal- do you think being pregnant kind of affected your mental health diagnosis like how your bipolar affects you?

P: yeah I would say it definitely played a part. yeah it definitely got worse.

I: Okay So what do you wish all doctors healthcare providers knew about pregnant bisexual women?

P: I'm not really sure.

I: Anyone that's maybe a sexual like has a different sexual orientation than just like 100% straight?

P: I would just say that we're different like it definitely a different experience for people, and you know, being a woman bisexual like it's definitely… I'm sorry it's so hard to explain like I'm not really sure I'm gonna be honest.

I: that's okay you don't have to like that really you cannot say anything wrong here.

P: Right cuz I'm not like I said I'm not the smartest so I really don't know all the time.

I: That's fine. I mean I don't think it has anything to do with how smart you are either, these are like really- I don't know they're like some people love these questions and other people's like we just aren't really interested so it’s no big deal. yeah no problem, so you be you do things like being a bisexual woman, he said, and maybe makes it a little bit different for you?

P: Yes, I’m so sorry I like I'm gonna throw up. Give me like two minutes.

I: Yeah go ahead.

P: I'm so sorry.

I: Do you want to end the interview if you’re not feeling well?

P: I'm fine I just got nauseous- real quick, for a second.

I: I mean there's only two questions left anyway so we're about to be done.

P: That's fine, we can do that.

I: What do you wish all LGBT Q plus bisexual women whatever you, you know, however, you want to say it knew about marijuana use?

P: Honestly that 's good, it's very helpful, I would say. you definitely feel like well the first few times you definitely feel like out of the world like it just takes you out and then like you just feel so good, like but use it wisely, because it definitely does can make you lazy and not wanting to do things like takes a lot of money, yes, a lot of money. A lot of money.

I: yeah I wonder when they're going to start having health insurances cover it as it becomes more legal you know?

P: Yes, that would be great really.

I: yeah I wonder if that'll happen.

P: Probably not- it's not legal in every state yet.

I: You’re right. Why would the government want to do anything that could help people?

P: Exactly or to help people honestly.

I: yeah okay last question, what do you wish, like LGBT Q plus bisexual women knew about tobacco use?

P: It's not healthy and it should not be used, and that is also expensive. it will take your wallet dry for sure. And it's not worth it, I will say that it's not worth it.

I: yeah yeah. Okay, well before I go I always like to ask, like is there anything that I could do better in like the next interview, anything that was confusing? What do you have any suggestions for me please?

P: No, not at all, I think it was perfect.

I: I really enjoyed doing this interview with you, I think you have really smart, interesting answers so I really, really appreciate you communicating with me like I know we had to reschedule a bunch but that never bothers me so thank you.

P: yeah i'm so sorry about that I definitely- things just keep popping up and I’m just like oh my gosh like i'm just trying to do this.

I: yeah no I totally get it. Honestly, as long as I'm very flexible, as long as somebody texted me like I don't you know we can reschedule a thousand times it really does not matter to me.

P: Thank you for understanding seriously.

I: No, thank you for doing this, I really appreciate it. I'm going to put the $50 on your part, now you get X funny for any reason, just let me know it should be totally fine, but you know, sometimes technology has a mind of its own so.

P: Right, thank you.

I: Yeah, thank you.

P: You too. You have a great day.

I: Okay, you too bye.
