# Supplementary material for: Experiences of postpartum mental health sequelae among black and biracial women during the COVID-19 pandemic
Source: BMC Pregnancy Childbirth. 2023 Sep 4;23:636. doi: 10.1186/s12884-023-05929-3 (PMC10478375; doi:10.1186/s12884-023-05929-3)
Supplement: Supplementary file 18 — Supplementary Material 18 [file 12884_2023_5929_MOESM18_ESM.docx]

**Supplemental File 1.12 Interview Transcript with Participant 5102**

I: On the screener you told us that you are bisexual. Is it ok if I refer to you that way? How do you want me to talk about your sexuality for this?

P: I will say bisexual, honestly it doesn't matter.

I: LGBTQ+ is a term we use to capture everyone that identified not 100% heterosexual, so it's kind of like a wide umbrella. A lot of these questions were about pregnancy, healthcare, and I know that you chose to end the pregnancy, so we frame it as reproductive health care, and I just want to go off of whatever you're comfortable talking about.

P: You're good, you can talk about pregnancy and you can say pregnancy, or you could say reproductive healthcare. I'm pretty easy going, so yeah, you're fine.

I: Okay, let's start by talking about some of your healthcare experiences, particularly around the pregnancy and with choosing to end the pregnancy. When we say healthcare it's like doctors, nurses, midwives, and the physical places where you got the care. What were your experiences like with your pregnancy healthcare?

P: I actually think it was good, it was good, they are pretty thorough, always checking in, and just always being on my back about it honestly.

I: What do you mean?

P: Like I truly feel like they were good doctors, they will always have me schedule and talk about things, just always being there honestly. They were good. I don't think any of my health care experiences were bad. All of the doctors were pretty easy understanding.

I: What were they understanding about?

P: I was telling them my choice about termination and they understood. They were very open, and they let me express myself during the time. And I think that's what's most important about being a doctor, because if you’re up in my business, then you should be not judging me or insinuating just because you're against it, or you may not believe that. So, yes, I feel like they were great.

I: I'm glad you had that experience. That's really good to hear. Did anybody during that experience talk to you about your sexual orientation?

P: No, they really haven't.

I: Is that something that you would want them to talk about with you? How do you feel about that in general?

P: It's not really like if I want them or don't want them to. It is just like, what do they have to do with anything? What my sexuality or sexual orientation going to add to my care? So yeah I mean it doesn't really matter. They don't talk about it, obviously, because it's just “none of our business” I guess.

I: Do you prefer not discussing with them about your sexual orientation?

P: I wouldn't say I don't prefer it that way. I just would say that I'm not really against it, or for it. Truthfully it is what it is. I'm not going to be like “no, you can’t ask a question”-- if it's regarding something that they can help me with, or a psychological issue that's going on, then ok we can talk about it.

I: Yea. If they did bring it up to you, how would you want them to talk to you about it, if it was something that they needed to ask?

P: Even though I feel like they will probably go with the way of their professional questions, they will need to ask in order to get to the root of what they're trying to help with or resolve.

I: Do you think that you would feel obligated or comfortable sharing with them that kind of stuff?

P: I’d be comfortable, just because, I work in a doctor's office myself. A lot of the patients were very open to talking to us because they're in our care, we handle their business, so I would be comfortable with talking to my doctor and stuff like that about it.

I: So you're a patient but you're also in some ways, like a healthcare provider yourself.

P: Yes, and a lot of them are open to me just because of my personality makes people want to gravitate towards me or share information with me.

I: Yea, what kind of personality do you have?

P: I have more of an outgoing personality. I understand people on all types of levels. I will say that I really work well with people, and I can really read energies very well too. I'm really just a people person. I'm very outgoing, very ambitious, and yeah that's what I would say about myself. I am hard on myself, and I should stop going that. I’m a self-critic, but that’s how you’ve got to be when you’ve just got yourself.

I: Yes, it's hard, that's a hard one for a lot of people, I think.

I: So what about the physical environment of the clinics, like Magee, or wherever else you got reproductive care. How was the office setting for you?

P: I think it was pretty much normal- I think it was great. I don't think anything was really wrong with them on the setting. I liked how they had to color-code directions and everything, like that was pretty smart. I liked how they did that, I thought that was a good way to direct to patient, because patients don't read and they just don't pay attention.

I: Were there any resources that helped you a lot, related to your pregnancy and termination?

P: I reached out to a woman. She wasn't in the medical field, she was just some of her own kind. She basically just talked to me about the whole situation, and you know, was very open and understanding of why I was terminating and she was like a resource for me.

I: Was she in the office system there?

P: No, she had her own thing going on herself. She wasn't a healthcare worker. She was just there for mental health support. And I feel like I was guided to that woman because I found her through a link and she listened to me and reassured me and I need that.

I: So it's kind of like a mentorship.

P: Yes, that’s what I wanted honestly, yeah.

I: Okay, and she was able to support you and understand your situation.

P: I feel like she was able to support me mentally. A lot of people around me weren't supporting me mentally the way I needed to be supported. That's why I wanted to reach out to different individuals, because sometimes people that I'm around are not always supportive of you the way you need. Like the sensitivity, you know.

I: Yea, did your family support your decision to end the pregnancy?

P: My family weren't really either, or. They know they're not interested abortion, but given my circumstance, given who I am, given the individual I was with and how it all happened-- they were pretty good with understanding why I would make the choice I would have made.

I: So you had your family-- kind of supportive, but a little bit indifferent?

P: Yea, they were kind of indifferent but they weren't rude about it. They were just very indifferent and understanding, at the same time, because of the child's father.

I: Yeah, well that situation wasn’t ideal for you.

P: It wasn't ideal. It was very toxic, and it was very abusive, and it was never what I thought it was. I was very young, so that's why I was given a wake-up call to slow down and look at myself and say to myself “what are you doing?”

I: So the situation showed you some things?

P: The situation opened my eyes to more of myself. I'm a deep person. I feel emotionally, I feel things that's in my bloodline, I feel things that are probably passed on to me from generations before. So the situation opened my eyes to who I want to be like, what I am really doing, why I am choosing this. My ambitions are elsewhere, so I feel like it opened my life on multiple levels.

I: You got something that you needed out of a situation that could be viewed as difficult. Were there any kind of resources or mentorship or anyone that you turned to that was specifically related to your sexuality and being pregnant?

P: Honestly, no. the most someone ever did was a peer helper, and that was in high school, and that was just it, but there was really no resources that really reached out to me. I am more of a self-healer and I don't really like to seek help. Once I have my epiphany, that's when I'm going to start moving on to mental health, motivation, etc. I'm not going to stay in that depressing, really gloomy state, because it is a very depressive state when you go to a termination.

I: So there was depression that went along with your termination before you had an epiphany?

P: Yes, I feel like it was just depression in a way of how everything was just honestly all coming down on me. I wouldn’t say it was chronic depression- it was just a state of I wasn't being myself type-of-state.

I: Like a situational depression kind of thing.

P: Yes.

I: This is an abstract question, but I feel like you already rolled with it-- so far, what was it like to be like not 100% heterosexual and pregnant?

P: I would say that it was normal. I'm a person who's content with myself. I don't need validation from anyone about my sexuality. I like women, I can be pregnant, that's okay, that makes me happy, my mind feels content, it was normal. That's me. All of the package.

I: Yeah, did you have to overcome anything to get to that point with liking women, like was there a time when that was harder for you to accept about yourself?

P: Truthfully, no. I would say that my aunt was a little bit against it. But, really, I would just say no. My family… they're not really against it either. Like taking my girlfriend to family events, reunions, they were always like, “you’re family,” they were cool. But my aunt, she was more judgmental because she was like “oh you're in a thing, a phase.”

I: Yeah, she likes to diminish your relationships.

P: I would say she likes to belittle our mindsets. She was rude about it, then I don’t let her come over, and I nicely manage that. But she just liked to belittle everything, like how you build your relationship, everything… after a while I told her to back off and then she backed off. You can't try to tell me how you live or learn, you gotta let me see it for myself.

I: Yea, so, you set a boundary.

P: Yes, I build a lot of boundaries with that woman.

I: Sometimes that really helps protect the parts of ourselves that we need to protect.

P: Yes.

I: Okay, so before we switch to next topic, we're going to dive into some marijuana questions: is there anything else you think is important to share, about your sexual orientation and like receiving reproductive care?

P: Honestly, no. I think you asked good questions.

I: Okay, thank you. Let’s go to this marijuana use section. It's totally confidential: we don't share it with health care providers, law enforcement, any of that stuff. It's never going to be linked to your name or anything. The first question is, have you tried marijuana before?

P: Yes.

I: Okay, can you tell me about your first experience with marijuana.

P: It was me and my best friend and we were being young and trying to explore and we tried marijuana. It was fun honestly. I can definitely say that it opens another realm in your brain. It makes you feel calm. But I didn't use marijuana all the time. If I was going through things like my pregnancy, I didn't smoke marijuana. I'm not a person who uses while I’m going through a real situation like that. I'm not a user who likes to use it for pain and for getting over things. I only use it for fun times, just being young and just wanting to expand and trying things that are new, and it honestly felt good. I was pretty smart about smoking pretty clean, brushing my teeth, and make sure that it didn't build into my system. Because marijuana has a lot of effects on a lot of people in my life.

I: Wow, you said so many things that I thought were amazing and interesting. So you made this distinction: you didn’t do it to get you through stuff-- what makes you not want to do it through hard times? Why don't you turn to marijuana?

P: Because honestly we're all put through challenges, and we have to be strong enough to see the light at the end of the tunnel, and sometimes it's not always good to escape reality, to escape that realm. Using marijuana, you really can't be yourself, why do you want to put yourself in a deeper funk as it is? People in my family, like my mother and my friends, my mom was on drugs. So I stay away from alcohol, I stay away from everything. My mom has 10 kids, and basically a lot of my siblings, down the line, they have addictive personalities like my mom. I don't want to build that for myself, I don't want to do that. All of their addictive personalities are built in and they were only growing because it was manifesting because of the things going on. You gotta learn how to handle situations. You gotta learn how to control your emotions. You gotta be there mentally to effectively keep moving. If people think doing drugs is going to take away their problem, they're stupid, because there're a million problems in life. You gotta be able to have standards when you have challenges, so you can keep moving on to the next level.

I: How did you come to know that?

P: Because I'm a person who can see a lot of things, since very young, I picked up on a lot of stuff. I knew my mom was on drugs. I know that some of the things that I do today, is why I was affected by her. I see my siblings and everyone else... they're not people who are really mentally there when I talked to them. Those drugs and all that stress and all that stuff take a toll on your life today. It pushes me not to even want to be like them. Like I can't even have a conscious conversation with mom because she's just not intellectually there. Like you're not really on my level intellectually, and I'm not trying to toot my own horn, but this kind of makes you put things in perspective. Like, dang, when I have kids, or when I have nieces and nephews, they will talk to me. I want them to come to me for advice. Because the support is not around them. It's not given to them in their home environment. So, someone has to be that person who is mentally stronger, who is mentally healthy. So that pushes me away from drugs, and also I just don't want to live that lifestyle, because habits grow; once you do one thing again, it becomes habitual.

I: Yea, it's so interesting that when they grow up in an environment like that, like you said, they become an addict, following the steps, whereas for you, you decided, you had the conscious decision of “you're not going to live that.”

shea Johnson: Yes, exactly, and even when I was peer pressured into doing worse drugs, I always used to come out and tell people “no, I'm not doing that” because when you actually speak up for yourself, people look at you different, and people started having a certain type of respect for you. A lot of people come back and say “you were right about that.”

I: So it's like you got to you model that to other people, too.

P: Yes, I might have other people… I call them out and say, “why are you doing this? Don't dim your light!” You know, like us, for fun, that’s fine. But don't use it everyday, don't use before school. Be in your right mind. Because then you're going to complain to me when you're a failure and you want me to help you.

I: Yea, I really liked the “don't dim your light” thing you said.

P: Yeah, because people get carried away; it's super easy to get carried away. And people who you want to see go places and stuff, I have to tell them like, “hey you're already going to great places; you're my friend, like if I wasn't your friend I wouldn't say this but, like you gotta do the right thing.” No. Don't do that every day. Maybe only on weekends when you're having fun, don't become a habitual user like the people around us in our school. Like why would you want to be like them? And my friends would be like “yeah, you're right, honestly.” I actually care about my friends. It just takes someone to speak up.

I: Yeah, because there are consequences for using every day.

P: There is a lot of consequences.

I: What do you do, then, to cope with things that are stressful, or when you're depressed?

P: Okay, I'm in a whole realm of a lot of things. When all the problems are coming at me like all at once, that’s a sign to me that I'm very, very close to accomplishing and getting to what I want. And how I cope with this is, I listen to motivational videos. I honestly talked to myself and I asked myself: “what's wrong with you? What's going on? Why is your energy not where it is supposed to be?” I meditate. I go to the park, I really connect with nature. I'm a very spiritual person. Like trees, they take away your negative energy and angry thoughts. Being in a more intelligent or spiritual space…it's like mercury retrograde now so a lot of things are really happening and stirring up, because it was going on and not within this realm. But yeah, I'm a self-healer. I'm a very motivational person. Where I came from made me motivational, made me better, made me look at the other side, and made me not want to do this.

I: Yes. Nature's very powerful. Also, mercury in retrograde has been causing me a lot of funny car

technical issues.

P: And that's why I’m rolling with the punches, because mercury retrograde is November 1^st^. It’s not permanent, all pain is temporary, and that's why I try to remember that. You only go through it.

I: I liked that you kind of have this understanding of, “okay, when it's really hard, when all of the things feel like they're coming at me, it means I'm about to have a breakthrough,” like it means things are about to be given to me.

P: Yes.

I: I wonder if you could share what helped you that could also be shared with other people to help them.

P: Truthfully, I only share one-on-one. A lot of people say… by the way, I'm 20 years old… a lot of people my age are really not ready to accept themselves first. You need to accept yourself, set all your path, accept everyone for who they are. And set you aside your flaws so you can come to awakening within yourself. When I try to communicate this, they reject it, they always think I am just trying to act like I am better than them. Now I try to only share advice and give this message to people who are willing to listen, and I only connect with people who are at my level with this topic, intellectually. My boyfriend is not on my level, that's where we messed up, because he's not really ready to accept, and he's a little older than me, like two years or three years older than me. And it's time for him to wake up-- he talks about things that triggers him, things are makes him go into these spaces, and I told him that he needed to work on himself and do some inner work. It’s very hard for people to get this understanding. It is very, very hard… not a lot of people understand this.

I: Like you said, I think the key word on how to cope better and feel better, is work! Like there's work involved-- it's not easy.

P: Yeah, and one thing I will say is, I just lost a friend last week from this. Already, I really don't have much friends-- I have associates. Unfortunately, I had to tell a friend, like, you know, “I don’t want to be your friend no more because you don't listen to me, and you're rude to me; when I tell you things I want to talk and venting, you just disregard everything I have to say.” I'm going to watch my words in communication, because only people at my level will understand. For the people who understand me they will know I’m giving them advice, but for people who don’t they are going to think that I am giving them a lecture. I'm not telling you that I'm better than you, I’m not comparing myself to you, I’m just telling you how to master peace.

I: Right, yes, it's hard. So, the last question about marijuana is more about what, in general, do you think about marijuana use and pregnancy?

P: I will say, honestly, marijuana use in pregnancy, I think that is not good, because the baby can have addictive personality. I just don't agree with doing drugs while you're pregnant, truthfully, I just don't agree with it, but people do it.

I: Do you smoke tobacco at all?

P: No.

I: Okay, have you ever tried it?

P: I’ve tried cigarette, and it was terrible. I didn't feel good. I just wanted to know what it was like, what it tastes like-- it tastes terrible.

I: Okay, so the taste was enough for you to be like, “I don't want this.”

P: Yeah, I didn't even like it, I was like, “oh, my God, dirt. People smoke dirt, this stinks.” Yes, that stuff is nasty.

I: Okay. So did you think your family had anything to do with why you never smoked tobacco, or your friends?

P: I would say, no. I just have my opinion on it, I feel like the way tobacco leaves a taste and smell, it can make your breath stink and stuff, and maybe makes your walls yellow like that's dirty. I don't like that.

I: Ok. Next question, imagine being in a perfect world. Like a straight, bisexual person in a perfect world, what do you wish all LGBTQ+ women knew about being pregnant?

P: I really wish they knew that… your current situation is not your permanent situation.

I: So it's like, things are temporary, things will change... And then, what about… what do you wish all doctors knew about reproductive care for LGBTQ+ women?

P: I truly don't have an answer for that one. I just think that I wish they knew more about their orientation, deeper into it. It takes research and understanding to understand that more. Not the attitude of, “we can’t help you, because you're not a woman” type of view. Be respectful.

I: What do you wish that all totally straight women knew about marijuana and tobacco use?

P: I wish they would know more about their health and more about the side effects. I wish they would just be able to love themselves more and stuff like that.

I: Okay, is there anything I didn't ask that you wish I had asked first.

P: I would have liked if you have talked about family upbringing more, and how some of those can have a psychological effect on things.

I: And, did you think that that would be its own section, or kind of mixed-in with the marijuana and the healthcare questions?

P: I feel like it could be the umbrella of it, because your upbringing has a lot to do with how you are today, how you were conditioned to be one today.

I: Okay, and do you think your upbringing affects your sexuality?

P: Really, your upbringing it doesn't affect your sexuality, but I feel like a lot of people grew up in environments where if they've already been hurt young, then they will become homo, and some of them will be forced to be hetero, depending on their families, opinions and views.

I: So it can affect that, you think.

P: Yeah because we have a lot of people deal with parents not accepting other people with partners due to race, gender, you know. Some people may like a straight person, may date a pan-sexual or some person, so I feel like your family takes a big toll, because you want them to accept you.

I: Ok, that love is important.

P: Yea.

I: Before we end, do you have any questions for me?

P: Was I a good constituent for this questionnaire?

I: So, it was hard for me to maintain the researcher interview impartiality and not just want to like have a conversation with you because I was interested in and agreed with so many things that you said. I had a wonderful morning with this interview, and I really am very grateful that you agreed to do it.

P: Did you have any other questions that you feel like you are afraid to ask?

I: Oh, that's a great question. I don't honestly. I don't because I just felt like you were really willing to go there, to tell me about who you are, and I didn't feel like you were very closed off at all.

P: Awesome.

I: Okay perfect. I'm gonna put this $50 on your card, you can always reach out. We're also going to do another study soon; we haven't started it yet, it's kind of about Covid 19 and racism, which is going to be like a similar thing to this. But, the interview is going to be first, and it's going to be $100 for another interview like this. So once we get that started, if you want me to, I can reach out to you and see if you're interested about that.

P: Yes. I’m open to doing that.

I: Okay cool. So it will do like interviews like this, and then based on your responses and everyone's responses, then we'll create a survey. You guys are going to teach us, basically, what we need to ask about racism, so that we know what to ask you.

P: Okay, awesome, are you guys biased against different views?

I: And what do you mean?

P: Because like I feel like this one will be my new best one to do. I feel like it will be a great one for me. The amount of information that I have, the political views on it, I think that this will be a great conversation, you know, but I don't know if what I speak about will be taken out of context or not counted as valuable data.

I: Oh, no, we want it all. Anything that you can think of that you think could add to the conversation is valid, so, yeah, whatever you want to tell us is data.

P: Okay, great.

I: Well, I hope you have a good rest your day I have enjoyed this so much.

P: You have a good one.

I: You too bye.
