# Supplementary material for: Experiences of postpartum mental health sequelae among black and biracial women during the COVID-19 pandemic
Source: BMC Pregnancy Childbirth. 2023 Sep 4;23:636. doi: 10.1186/s12884-023-05929-3 (PMC10478375; doi:10.1186/s12884-023-05929-3)
Supplement: Supplementary file 19 — Supplementary Material 19 [file 12884_2023_5929_MOESM19_ESM.docx]

**Supplemental File 1.1 Transcript of Qualitative Interview with Participant 5188.**

Q1 5188

Interview Transcript

I: Okay, there we go. Um, so first I just want to get to basically know you a little bit and what your experience, um, of being pregnant has been like, so like how have you been feeling? How was your first doctor’s appointment?

P: Um, so this is my third pregnancy. Um, I guess everything’s been okay. Nothing that I’m not used to, I guess. The appointment went well, I guess. I have my prenatal appointment today. So far everything is okay and that’s a good thing.

I: Mhm. Yeah, especially because it’s like just weird times kind of like in the whole world so I wasn’t sure if that was gonna like affect people’s pregnancy or feelings about being pregnant right now.

P: Oh yeah. Umm, well when it comes to that I guess- I can, you know, just depending on who you is, you know. Me, myself I-I just separate myself from that kind of outside world. You know when it comes to things that’s going on out here, you know, I try my best to protect what’s in my household. So, that’s all that I can do cause you know we don’t have control of what’s going to go on outside or later in the future, so I can’t let that affect anything that goes on with me.

I: Mhm, It’s like easier said than done though, sometimes. So, you have had two other pregnancies. This is your third?

P: Yes.

I: Okay, it’s been a similar experienced you said.

P: Um, yeah. Pretty much yeah.

I: Are you someone that gets like morning sickness or is it- kinda how’s the first trimester for you usually?

P: Um, so yeah I have experienced morning sickness. Um, again I can just say it’s nothing that I’m not used to or that I didn’t see coming. Um, but it’s not worse than before or anything like that.

I: That’s good. So now I’m gonna kinda segue, dive right into the questions about marijuana and tobacco and I know you took the survey so you kinda know what we were asking about there. Um, for this interview I just really want to know like, first, what are your thoughts about marijuana and tobacco use like in general?

P: Um, I-I I don’t know. Uhhh, I guess it’s for- I guess it’s for different people’s preference. Um.

I: Mhm.

P: That’s all I can say. You know having different friends and family and stuff. Uh, and I don’t know. Yeah, that’s all I- I guess I can just say it’s useful for people’s different preferences and that’s up to them. Um, yeah I’m not sure how I- I don’t really feel any type of way towards it.

I: Mhm. What are your preferences around it? Like do you feel comfortable using it do you not like using it personally?

P: Yeah, no not me. Me personally, oh like, you know, growing up hearing the same, like, smoking can cause, you know, different things. Um, health habits and stuff and I guess I like- I don’t know if I believe in that. I just always was told that, so I guess it was just my- my preference. I don’t care for it.

I: Mhm

P: But, you know people around me if they do it, again, that’s on them. I talk to people and everyone have different reasons. But, you know, me I don’t care for it to use, to use it.

I: Do you remember like Where you’ve heard, like, where did you get kind of like the information that you have about marijuana?

P: Um, everywhere. A lot of places, different people, you know social media of course. So yeah. It’s like people again have their own uses for it, reasons for it. Um, I read about different uses for it and stuff so it’s like, you know, there’s again a lot of the same way, you know, there’s um. I guess I can just say different uses for it, you know. Like cause I read about and heard about it being used for medical reasons. Um, it being just used for fun I guess. Like, literally like different reasons and so.

I: Mhm. Yeah do you remember specifically the different reasons that you read about. Like you said medicinally. Was there anything else that came up?

P: Um, no that’s all. I just came across it. I just seen it like, um, like I just seen it saying that it can be used for like anxieties, um, stress. Um like, I read about it being used for, um, like, like after like I guess like if say you get into like a really bad accident or something like they just prescribe you different drug uses after. Yeah, um, I heard about it from like a family member. Like I think someone had like schizophrenia or something and they were prescribed like a medical card and that’s what the medical card was used for to get that. I came across stuff like that.

I: Mhm. I’ve seen that stuff too. Is that how like most people in your life that you know that use marijuana is that what they use it for or do people use it for other things like, you know, recreation or whatever other reasons?

P: Mmm. I mean, well, right now I don’t involve myself with too many people. So, um, the people I can think of from the top of my head they’re like older folks so I think there’s probably just think of, you know, maybe something, you know, they’re probably using it for, you know, something like that.

I: Um, what about tobacco use in general?

P: Um, I’ve heard like stories that that can cause like lung cancer and stuff so I never recommend that to anyone or, you know, thought that that was okay. Like I said if people do it, that’s on their (unclear). If like for example, my grandad (unclear) I was always told (unclear). I mean I hate the smell of them.

I: Yeah, he’s like a different generation too. Like, I feel like it kind of changes sometimes for different age groups, what’s normal.

P: Yeah.

I: Did you remember learning about cigarettes or tobacco use anywhere or is this all stuff that you had to like come across on your own?

P: Um, yeah, uh, yeah, I, uh, let me see. Like as far as like like doctors and, um, I don’t remember having- I don’t remember having like a class about tobacco that I can think of, but of course like teachers and stuff have talked about it. As far as like learning if it was pretty much like I said. What I always was just told and then just ran across.

I: You think one of like marijuana or tobacco is worse for you or are they like similar? What do you think about that?

P: Um, yeah I definitely think cigarettes is worse than marijuana.

I: Why do you think that?

P: Um, I think because, um, for an example like it says like tobacco and stuff um like it isn’t good for you and I remember them saying like, like how much tobacco is in like cigarette or something and it can, you know, cause like, you know, lung cancer over time or heart disease over time and I believe, um, I believe, you know there was people who physically was going through that before.

I: Mhm.

P: Compared to marijuana, um, I haven’t you know for an example heard of anyone going through anything like that. Um, it was always the opposite. When they say they use they say relief or whatever it is that they uses it for a lot. My understanding marijuana is not killing people or any type of health problem, anything like that compared to tobacco.

I: Yeah, so it sounds like you’re learning that like tobacco might have more serious negative health-

P: Yes, negative health issues comp- yeah

I: Yeah.

P: But we know that smoking over time causes them.

I: Do you mean for tobacco or for marijuana too over time?

P: Tobacco.

I: Okay. Do you think like certain, you know, like some people smoke blunts, some people use bowls or bongs. Do you think certain ways of smoking marijuana is maybe better or less harmful than other ways?

P: Um, I guess I would say, uh, that I d- I can just- I’m not sure honestly cause I don’t even remember- cause I remember asking people like (unclear) well when you get weed, marijuana off of a doctor or something like how did it come? Was it like the plant? Is it like a pill? Is it a joint or something? So, I don’t even- I wasn’t even told that. So, therefore, like I don’t even know personally. I would just say, um. I would just say I guess however they feel comfortable doing it because, you know, at the time I also learned, you know, different types of- like you just said ways of smoking and, you know, some people’s preferences are they don’t like using different things. Some people probably don’t know how to use it or anything so that would be their preference unless there is a way that they tell you to do it. For example if they prescribe you it unless they give you a “hey this is how you use it” or whatever.

I: That makes sense. Like a doctor kind of recommended way.

P: Yeah.

I: I’m like picking up some feelings that you have a fairly nonjudgmental vibe about people like, you know, you said marijuana is really not for you for whatever reason, but I’m, like I’m curious about how you this reached this non-judgmental stance. Does that make sense? Like what makes you feel like it’s not for you, but it’s okay for other people. It’s an interesting perspective that you have.

P: I mean cause me personally like I can just say like I can understand when, um, you gonna different friends and stuff and I can just say like I can understand when someone say like, you know, “Hey, this is, you know, this is going on or this is what helps me out for this” or whatever and if that is helping it and I see a change in it or something, you know, like I said so be it. I feel it’s just the same as just how we said if the doctor prescribe you something and you’re on that medication or, you know, something’s wrong or you have some type of pain and you need something or relieve that or, you know, something to control that or anything. So it’s like if that’s their preference, you know, marijuana use why not? If that’s what’s helping them. That’s what’s improving it. If so, I feel like it’s the same as you putting them on any other drug to help prescribe something, to heal something or whatever the case may be. So, and like I said, from my point of view, the folks that know witness (unclear) like I said I seen a difference, a change. I was like you know so- you know so be it.

I: You have like some people in your community, in like your network that you feel like you’ve witnessed like some positive benefit from their use of marijuana in like whatever behaviors or whatever was going on for them?

P: Yes.

I: Do you like know specifically? I know that’s kind of like a very specific question.

P: Um

I: Does it help with like appetite, sleep, anger? I’m just like curious about what people might be using it for. Like what they might say it helps with.

P: I mean like I said, um, you know, like anxiety, stress, you know, every-

I: You did say that.

P: yeah

I: Now what about like we talked a pretty good amount about just general marijuana and tobacco. What about like marijuana use for women that are pregnant like during their pregnancy? What do you feel about that?

P: Um, yeah it’s sort of the same. If the woman says she needs it for, uh, you know, for as far as her use like, you know, pain or anything like that. You’re done (speaking to child). As far as whatever use that she needs it for, um, like I said, uh, I’m trying- it’s like, uhhh, I’m trying to think like how can I say it? Like as far as like when you- I can say when you’re going through the process of like having contractions or having going through labor during that time period, you know, you’re in so much pain or what not and for an example like if you, you know, want a medication or something like I just felt like for me cause I had gotten an epidural once and I, you know, seen the difference from that and going natural and I’m like, you know, after experiencing that and knowing people who talked about using it when I ask why and they say people use it for as far as if they’re having any type of cramping or something like that or vomiting if they need it to help them with that, you know, cause some people say it helps them, uh, with like their eating habits. Some people say when they smoke they can eat more after. Different reasons, like I said. If they’re having any type of pains during their pregnancy and while they’re home cause I know I have experienced it pregnant there’s only so much that we can do, that we can take to control any type of cramp- anything we’re having. So, it’s like you know if they felt- if they put that in use and they did feel like that’s something that’s helping, again, why not? Um, and also you know knowing for example my mother she said that she used to smoke before when she was pregnant with me and my siblings and I’m perfectly fine, healthy and, you know, we are living proof and example. So, I don’t have enough- I don’t have a say as far as “Oh, well how would you feel if women are smoking while they’re pregnant? How will it affect the baby or how after?” It’s like I’m a walking proof of someone who smoked marijuana and cigarettes and drank while they was pregnant so, um, again it’s- it’s all your preference. I feel like because we are all different people whose bodies are different and we grow differently and things happen in different periods of times no else can really control that basically. When it comes to that so if someone take advantage of something that they feel as though is going to help them out. I’m not speaking as far as any type of other drug or any other bad habits or anything, but you know specifically marijuana use, you know, go ahead.

I: Yeah, I feel like I have so many questions after that. That answer was like so interesting. Thanks for sharing all that information, like personal information with me. I’m wondering like, um, you know, you still have this people are gon- should do or can do or will do whatever is right for them and so, I’m wondering kind of like how you developed your preference to not do it? You know, like it’s like you don’t, you’re not saying it’s wrong or right, but you’re also like having your preference to not and I’m wondering if that’s just cause like a personal experience where you don’t enjoy marijuana or if like, you know, like what kind of prevents you from doing that yourself? Does that make sense?

P: Um, yeah. Because just like I said as far as for an example with like, uh, tobacco, cigarettes and different types of tobacco like that I don’t like the smell of it. Um, I don’t like the smell of it. As far as like with marijuana, um, yes again I don’t as I can just say I don’t surround myself around the people who does it so it’s not around me to be like let me try it or what not, if that makes sense.

I: Yeah. Yeah, if it’s not there then… Um, I also heard when you- you answered before it’s like, um, do you feel kind of like doctors and like OBGYNs are able or do provide like good care for women that are experiencing those kinds of like negative side effects during pregnancy? Like what has your experience been with like how helpful medical providers are?

P: Um, I mean, uh it’s a weird way to say it because let’s say when it comes to our body naturally we are all different and, and there’s only but so far you can go when its someone else, for example, examining you and then trying to take that second guess, uh, trying to figure out what medication will help that out. So, like from my experience it’s like I’ll be like well you know no they haven’t, but it’s like I can’t say they haven’t because you know for example like they told me, you know, well as far as with my vitamins if my vitamins we can try a different type of prenatal vitamin if that’s causing it or what not. You know I can just take and (unclear), uh, um, give me more Tylenol. So that was only but so far that they were able to help me, personally. Personally, I just have to physically find out myself what can help my body out. What can help my body get over this and overcome this feeling or this habit of vomiting or getting sick or overwhelmed or whatever the case may be? So, you know like when it comes to doctors I just feel like personally there’s only but so far they can go because I always tell people nobody don’t know your body more than you. You know, at the end of the day if it’s a doctor, a scientist, whoever like that’s your body you know what I’m saying and no one can say anything about your body more than anybody.

I: Would you feel comfortable like sharing if you were smoking marijuana with your provider? Is that something that you would talk about with them?

P: Yes.

I: Do they ask you about that at all? Do you remember? Was that like brought up?

P: Yes, oh yeah. It’s common when you’re at doctor’s offices.

I: and you would feel like safe and comfortable disclosing that?

P: Mhm

I: I can’t remember. I know that we talked a lot so far I feel like about marijuana during pregnancy and you did talk a little bit about tobacco too, but I’m wondering if we can kind of like circle back and touch on that specifically. Like, if you feel using tobacco during pregnancy is different than your opinion on marijuana or if it is the same like what- what’s your opinion about that?

P: I mean honestly I can just say it’s not my preference, you know cause, you know, but like I don’t recommend it but I don’t know. I don’t know about that because like I said it’s just it seems for a fact that tobacco causes, you know, things that’s not good for your health. I don’t think, you know, that is okay. Honestly, but when it comes to someone doing it like I don’t know. That’s- I don’t know. That right there- I don’t know. I don’t know honestly. Cause it’s like- like if I was to see one again I would be like that’s not okay like I wouldn’t do that. But if they say “Oh, I’m using it it’s my own preference blah, blah, blah” I don’t have an argument with them. That’s just on, you know, my opinion versus theirs and my opinion is I wouldn’t recommend using it while pregnant. I don’t- I don’t see, see that’s the thing that’s where (unclear) comes in because I never used tobacco cigarettes or tobacco, so I can’t even say like, you know, but so much. I can’t be helping that person out in that type of manner or whatever because I wasn’t in that person’s shoes. Like I don’t know what that relief they feel is or what not.

I: Mhm. Do you think there is like- like what would that benefit- what might the benefits be for that person that’s smoking, you know, cigarettes while they’re pregnant?

P: Oh, I don’t know. That’s what I’m saying.

I: Yeah, that makes sense to me. Um, sorry I’m ping ponging around a little bit, but I want to you talked about how you would not have a problem telling your doctor if you were using marijuana and I’m wondering like what makes you feel like that’s something that you would tell your doctor? Like why would you share that with them?

P: Ummm, I mean, personally, I feel like my first my first comment is y’all want to know. Y’all are asking and I feel like, personally, I was always taught don’t ask something you don’t want to know the answer to like. I mean if you want to get the answer, you know, but and it’s, um, I don’t know. Me, personally, I’m just- I’m just like- I’m just like I don’t- I don’t know how to say it like I don’t care like first it’s like okay if you asked me about it I’m gonna answer truthfully and if you feel like you don’t like the answer or have a judgement, then I’ll just say that’s on you. Like you shouldn’t have asked. You know, I don’t- I wouldn’t judge you because I didn’t ask you what you what you do because there’s bigger- there’s more than- there’s worse things than marijuana. I feel like that’s the least that they should be asking as much things that are around and things that I know people is taking and using. That’s the least that they should be asking and worried about is some marijuana.

I: Do they ask about those other things? Do they screen for like, you know, what other drugs are you talking about?

P: I mean I know for- I mean I know me, personally, I get drug tested. I get tested for different things, but I don’t know like I don’t know and me and my doctor we talk but I don’t know what they ask other people. Um, like I said whatever it is like I do know marijuana is one of the main questions and I just feel like that’s the least you should be worried about.

I: I’m interested. This is interesting to me. How else- or like what else do you think they should be asking young, pregnant women about?

P: Oh well like I don’t know. I don’t know any sorts of I don’t know pregnant women or any young woman taking or having drugs or anything like that, but I just know as far as those other drugs out, you know, it’s all around you know social media and stuff. So it’s like that’s the least that someone should be worrying about I feel like.

I: What do you see on social media about drugs? I’m really curious about that.

P: Just, um, I don’t know the drugs like off names. You know there’s different things and I know there’s worse things out there than if you think weed’s bad.

I: Mhm and so, if doctors are like, you know, you think doctors should be asking people about this. What kind of information do you think doctors should be providing people about this?

P: What do mean?

I: Like, um, say a doctors talks to you and this a whole other world and you say “Yeah, I smoke marijuana and I want to stop. I know that you know I just want to be extra careful with the baby.” Blah, blah, blah. What ways would be helpful do you think for you in your life for a doctor to talk about that with someone? Maybe quitting, slowing down, that kind of stuff.

P: Um, I’m not sure like… I’m not sure. I wasn’t like following along. I didn’t really get that- understand that.

I: It was a long question. So like how do you think…? Let me think about how to ask this so it actually makes sense. If a doctor is talking with a patient about marijuana like what do you think might help someone like, you know, they want to cut down or something. Like, can you think of any helpful information or stuff like that that you think doctors can provide?

P: Oh as far as if they want to stop using it?

I: Yeah, yeah.

P: Oh, um, there’s that- that that number. I’ve been getting the whole packets, the whole number or some type of place or answering machine if you’re addicted to something. They have that.

I: Oh, so they give a resource already to call.

P: Yeah, yeah, yeah a resource list, a resource list.

I: Would you like if you needed to would that be something you’d feel comfortable doing like picking up the phone and calling someone?

P: Oh no, oh no. See that’s the difference between me and other people. I don’t- I’m not an addict. I don’t have an addiction and my parents, my peoples I’d get bopped upside my head before it go down that road.

I: You think you’d reach out to your family first?

P: Yeah, yeah, yeah anything, you know, I have supporters and stuff that’s here and will catch that before anything, but with other people, um, uh, I mean, uh, I don’t be hearing too much of that type of organization, but I know about as far as, um, you know, um, what is it called, uh, other organizations like foundations that help people out who’s in need of help and stuff that need that’s good. So, if that organizations as far as helping you get over an addiction and stuff like that is really good for you and people’s comfortable with reaching out and doing take home steps then yeah I feel like that’s good and like I said they give you- I get that resource packet every appointment so, you know, and they always highlight it and stuff for you so that people if someone really needs it will be a good way to reach out

I: You get that resource packet from like the doctor from like social worker or just from like the front desk? Where do they give you that?

P: Um, yeah. I got it from a social worker before. I got it from yeah the doctors before too cause I think it was like there was a part of when I was leaving. I don’t know if it’s in a doctor’s office, but my doctor give it to me.

I: Yeah, what would- so like what would make you not comfortable calling one of those places? Besides the fact that you’re obviously not like you don’t need them, but like if you did.

P: Um, I don’t know. I don’t know. I can’t answer that question since I don’t exper- I don’t go down that experience that. But me, personally I get into, uh, allowing people in in my business like that so I feel like if it becomes something like that my family should take advantage if anything.

I: Okay that- I hear that. It’s more of like a- like there are people- you have people in your life that support you so those would be the obvious people to kind of reach out to.

P: Yeah.

I: They know you and care about you. Um, is there any difference that you’ve experienced like so far in talking to me as a researcher versus like your OBGYN provider?

P: Say that again? Is there a difference?

I: Yeah, like is there any differences in like communication with a researcher in this kinda situation versus like, uh, you know, your medical care provider?

P: I mean, yeah.

I: Tell me more, please.

P: Yeah, they’re my doctor and you’re not.

I: True.

P: But there’s a, um, I feel like there’s just a- what I mean by that is there’s, um, a level of standards for an example, um, how far we can take the conversation, um, and how much I want to say, how much someone wants to say to you, you know.

I: Yeah, what do you mean? Like are you willing to share more with your doctor cause they’re kind of responsible for your care or are you more comfortable sharing with me because I’m nobody

P: I mean-

I: in your life

P: I mean yeah, but specifically about this I don’t care because that is a topic- that’s a- that’s a, you know, big public topic that I don’t mind speaking about on anyone’s, you know, behalf. But when it comes to like other things, personally, medically or something like that then yeah that’s where as far as the extent of talking to your doctor.

I: So like, okay maybe, tell me if I’m wrong, but I feel like you’re kind of like saying that you don’t mind talking to me in this way because you get to kind of be like an advocate for- for-

P: Yeah, like cause we’re like talking about marijuana but if you was to start asking me, um, like questions about like- like my medical history or like something like that then it’s like I don’t have to tell you that type of stuff.

I: That makes sense. So, what we’re talking about is like of interest to you and you can kind of like share your beliefs about it.

P: Mhm.

I: Okay, I hear that. That makes sense. And do you like- never mind I don’t even know where I was going to go with that one. It was going to be another one of those like long confusing questions so. Um, okay. I feel like we- I’ve asked you a lot of questions and you’ve like shared so much with me and this was the first time I’ve done an interview like this and I just super appreciate that it was with you. I really enjoyed talking to you and-

P: Yes, yes it was fun.

I: It was fun. I feel like I like learned some stuff which is always good, um. I want to know like just the last thing before I let you get on with your day is like is there anything that I’m asking that I shouldn’t be asking? Anything I should ask differently? Something I’m not asking that might be helpful?

P: Um, Um I mean, uh, I feel like, you know, everything, you know, you asked was good. Yeah, I can’t really- yeah I think everything was fine. Um, yeah I don’t know if you like- yeah I wouldn’t do or say anything different or what not.

I: I mean you can always, if you think of anything you can always email or whatever if it suddenly strikes you. Do you have anything else that you want to ask or say before we end the interview?

P: Um, no I don’t.

I: Okay, well I really appreciate you doing this and is it okay if we keep reaching out to you at like your different trimesters?

P: Yeah, that’s fine.

I: Okay, cool. Um, then I’m also going to just add that other $20 onto your card like right after we hang up. Okay?

P: Awesome.

I: Thank you. I hope you have a good rest of your week and stay healthy.

P: You too. Thank you so much.

I: You too. Bye. Have a good day.
