# Supplementary material for: Experiences of postpartum mental health sequelae among black and biracial women during the COVID-19 pandemic
Source: BMC Pregnancy Childbirth. 2023 Sep 4;23:636. doi: 10.1186/s12884-023-05929-3 (PMC10478375; doi:10.1186/s12884-023-05929-3)
Supplement: Supplementary file 20 — Supplementary Material 20 [file 12884_2023_5929_MOESM20_ESM.docx]

**Supplemental File 1.21 Interview Transcript with Participant 5101**

SM Quali Interview with 5101

Interviewed by: 50

Date of interview 2.10.22

1

00:00:01.800 --> 00:00:08.220

NB: Okay, there we go I was I said record to the computer and I don't want it on the computer I want it on the upmc server so.

2

00:00:09.840 --> 00:00:31.350

NB: Okay um I haven't done one of these interviews in a little while, so I might be kind of like I don’t know, bumpy and awkward, so I apologize if that happens. Let me see so i'm going to use the term throughout the interview LGBT Q, so it just means lesbian, gay, bisexual queer or questioning.

And I want you to know or I want to ask you like, do you have a I think you said you identify as “mostly heterosexual” Is that how you want me to like referred to you do you have a preferred identity label or anything like that.

4

00:00:47.490 --> 00:00:49.590

[5101]: I’m just straight. That’s how I prefer.

6

00:00:58.590 --> 00:01:08.130

NB: Okay, so first we just kind of ease in a little bit and talk about your pregnancy so like, how do you feel your pregnancy went.

7

00:01:09.420 --> 00:01:18.240

[5101]: It was like very difficult on I had to keep getting these pregesterone shots because on my cervix kept wanting to open, so I had to get on every Tuesday, until, literally the end when they wanted me to have my baby. It was just a lot and it hurts so bad I hated Tuesdays.

10

00:01:29.850 --> 00:01:30.990

NB: That sounds stressful.

11

00:01:36.150 --> 00:01:41.070

NB: yeah did everything, like did the shots help in the end?

12

00:01:41.940 --> 00:01:47.190

[5101]: I mean they still made my cervix like hard like to open, so I was in Labor for two days. So everything was okay, after that, but it was taken a long time to open so. It was just painful.

15

00:01:59.250 --> 00:02:00.150

NB: sounds painful.

16

00:02:03.120 --> 00:02:11.310

NB: How did you feel about like the like your body changes, like I feel like our bodies change so much during that experience how was that for you?

17

00:02:13.050 --> 00:02:20.160

[5101]: Like okay I didn't never ever have boobs and then I got some of them, they went away after breastfed so it didn't work in the end. But I gained a lot of weight in like I didn't lose all the weight that I gained because before I got pregnant, I was like 110. And now i'm 130, but when I was pregnant, I was hitting like 140-145 during my pregnancy, I was getting a lot of weight, sometimes it seemed like too much, but I lost a pretty good amount of it after my pregnancy.

20

00:02:43.230 --> 00:02:51.330

NB: yeah it's a short amount of time to have like your body just go through all that and it got it has to but it's a it can seem like lot.

21

00:02:52.290 --> 00:02:52.710

[5101]: yeah.

22

00:02:55.740 --> 00:02:58.020

NB: How old is your baby now?

23

00:02:58.050 --> 00:02:58.440

[5101]: he's like 6 months

24

00:03:01.470 --> 00:03:04.800

NB: So he's still very newborn, very new.

25

00:03:05.820 --> 00:03:06.240

[5101]: yeah.

26

00:03:09.480 --> 00:03:10.050

NB: So. The next part of the interview is talking about your health care experiences and you'll probably notice that these kind of interviews like i'll do a lot of like, “Okay, can you tell me more about that” or like you know i'll try to make sure that I understood what you said and then maybe ask you to elaborate So the idea is for me to talk less which is sometimes hard because i'm very talkative.

And really hear about what you have to say so, the next part can you about health care experiences, and that means just during your pregnancy anyone in the healthcare kind of umbrella so doctors, nurses, if you had a doula or midwife in the hospital stuff like that.

32

00:03:58.590 --> 00:04:00.480

[5101]: okay

33

00:04:00.510 --> 00:04:04.020

NB: So we start broad. In general, how do you feel like your experiences with the health care system were during your pregnancy?

35

00:04:09.960 --> 00:04:27.720

[5101]: Really, I was in like a mommy and me like program so I didn't really have a lot of experience with the doctors, because they would just help me with anything other than my Labor and going for appointments, I really got help at the place I was at. When it came to my cramping they would help me with it, but as far as having my baby they were very helpful.

37

00:04:42.390 --> 00:04:43.890

NB: So were you at like at. Were you living somewhere that kind of have like built in classes, is that what you mean is that what you're saying?

39

00:04:51.480 --> 00:04:52.830

[5101]: yeah yeah kinda.

40

00:04:54.810 --> 00:05:05.250

NB: Okay um what kind of like what were some of your best experiences like with the hospital system about your pregnancy?

41

00:05:06.690 --> 00:05:12.930

[5101]: pretty much just finding what gender my baby was, because I just wanted to know so much. That’s all I really. I mean that’s the best part of it. Like I have, I have worse stories than good.

44

00:05:22.050 --> 00:05:23.700

NB: Can you tell me some of those?

46

00:05:26.430 --> 00:05:30.180

[5101]: Well, in the beginning they ask you if you want to like test your baby for down syndrome and stuff like that. And I wanted to get him tested because it seemed like the right thing to do, and they told me he had a chance of having Down syndrome, and then they made me go through all these things, all these tests and until I just said, I didn't want to do it, no more because I’d just rather just wait and see it out, because they had me like panicking.

49

00:05:52.320 --> 00:06:00.270

[5101]: And then we did this test and they said that it was negative, but it could be a false negative, so I was just like i'm just going to go with the negative because I don't wanna think my son has Down syndrome.

51

00:06:03.390 --> 00:06:04.380

NB: That sounds scary.

52

00:06:09.330 --> 00:06:11.280

[5101]: It was, very. It really doesn't bother me but I was still scared.

53

00:06:12.750 --> 00:06:18.120

NB: yeah I think it's seems like natural to be scared about some hearing news like that. Do you feel that the doctors throughout that process like listened to you or was there things that you really feel like they could have did better during that?

55

00:06:32.640 --> 00:06:45.600

[5101]: yeah like Magee treat you like you're a number, like they don't treat you like you're like a patient or a young mom that’s having kid, they didn't really expect me to know what to do either. Like they basically made it seem like yes i'm a new mom, but they didn't assume I knew nothing I didn't know how to feed him, I didn’t know how to do this, nothing. Like. I’m a young mom. I don’t know how to explain it. They just expected me to be careless.

59

00:07:04.710 --> 00:07:06.630

NB: yeah it sounds like they made assumptions about you.

60

[5101]: Yeah they did.

61

00:07:17.220 --> 00:07:27.420

NB: that's important information so thanks for sharing that that's kind of you know, one of the main reasons why we're doing these kinds of interviews we really want to hear what your experiences are like.

63

00:07:30.390 --> 00:07:31.200

[5101]: yeah I mean. When I went to [location? Millvale?], where I started where I went to the mommy and me program it was actually, they're very nice, like it's an all ladies thing kind of like Magee but it's very different it's called [unclear name] and it's so much better they're helpful, all the way, they're very nice there, I really liked it there, it feels like you're at home, like, I was at home, very nice.

66

00:07:55.860 --> 00:08:03.000

[5101]: and encouraging because I was ready to give up so many times, but it was like we were all having a baby, they were so happy for me.

67

00:08:04.290 --> 00:08:05.070

NB: that's awesome. So you said they were like made you feel like you're at home and everyone's kind of like in it with you, do you think there's anything that you can point to that they did better than Magee besides those two like huge things?

72

00:08:24.690 --> 00:08:35.100

[5101]: Well, like the way my, during the birth, they were calming and not like, if I screamed it in like Magee they like, when I had a miscarriage they like kept shushing me, but like there they were saying, let it out, they were very different, like they were lending me like strength that I was going through a lot of pain like, they were just like encouraging instead of shushing me and trying to calm me down, but it was irritating that I wasn't in a calming down mood.

75

00:08:57.000 --> 00:09:15.300

[5101]: And it just wasn't working and in [location? Millvale?] they were more of just trying to let me express my pain, get it out, because it was helping pus,h it was just very like they were understanding, they understood, because they all had babies. Everybody in their had a baby, they all went through it.

76

00:09:17.910 --> 00:09:22.800

NB: i'm so sorry that you were shushed during like a very, what can be traumatic and painful thing.

78

00:09:28.230 --> 00:09:32.310

[5101]: yeah I didn't expect them to do that, I didn’t want to have a baby again, but here I am

80

00:09:36.360 --> 00:09:46.050

NB: It also it helps like you felt like it helped that the women in the other program like had shared the experience of also having a baby.

81

00:09:46.680 --> 00:09:55.080

[5101]: yeah they that definitely made it so much better because, like they understood like “wow you’re in Labor for like too days,” they understood the exact same thing. And I couldn't eat they understood that. I was starving.

85

00:10:05.190 --> 00:10:08.610

NB: yeah I can't go like four hours without a big meal so.

86

00:10:08.670 --> 00:10:12.750

[5101]: ya know they brought me Wendy's actually that's what they did, they brought me Wendy’s. They're very nice.

87

00:10:13.620 --> 00:10:25.290

NB: that's so nice that's i'm so glad you had a kind of better experience since that first one does sound so like sad and frustrating.

89

00:10:28.770 --> 00:10:41.160

[5101]: It was very. And then when I had them they like they really took him in like it was their son, like they can I can I hold him, well I did just have him like an hour ago, but they were like, they gave him a hat this lady made, they took pictures with him if I would allow it. They asked if I needed a break, they would come and get him in the night, they were very helpful, like if I ever have a kid I will go all the way to [location? Millvale} just to deliver my baby.

92

00:10:55.530 --> 00:10:57.120

NB: I love that I love that. It sounds like to they did a lot of like. Like they asked you if they can take a picture of him, like you got to control the situation.

96

00:11:09.690 --> 00:11:21.510

[5101]: They asked permission for everything like” can I touch you here,” “do you mind if I do this” like they and they let me know everything like that was my other experience that I had a problem with inside of where was it. Inside of Magee.

97

00:11:23.490 --> 00:11:24.870

[5101]: Was it inside of. Because the IVs where like, for some reason my skin wasn't liking whatever they were putting into my arm. So my body was like react like it was bubling up in a blister every time they put in he iv me and they kept tricking me, I would be like, “This is burning!” and I look over and the IV’s back in my arm and it was irritating me so bad.

100

00:11:45.120 --> 00:11:54.240

[5101]: I understood I needed it but they didn’t understand like not telling me is making me like panic, because I keep feeling my arm burn. It was just so irritating I kept doing that.

101

00:11:55.110 --> 00:11:59.010

NB: yeah because it's like you actually have the right to refuse any medical procedure that you don't want.

103

00:12:03.450 --> 00:12:06.900

[5101]: Yes, exactly the problem. And the thing with - kind of the same thing happened that me, though, but it was different like I I guess “BV” or something whatever it is that you get tested for… what is called, not BV, what is it called…it's something you get tested for. Strep B or something.

106

00:12:24.180 --> 00:12:33.810

[5101]: I guess I tested positive for it, he said it happens, and they gave me, they have to give you a IV, or something, well you know. Well, that was also my skin still wasn't reacting very well to that. They kept taking breaks and they would tell me when they were putting it in, and it was just it was way easier for me.

108

00:12:43.290 --> 00:12:44.940

NB: yeah they like communicating with you about what was going on for your body.

110

00:12:48.210 --> 00:12:48.570

[5101]: yeah.

112

00:12:55.140 --> 00:13:07.620

NB: Do you have any other like things that you think Magee could change or ways doctors could be like better in general about pregnancy before we kind of move on to the next question.

113

00:13:08.400 --> 00:13:17.850

[5101]: I don’t think. I just think they could be a little bit more considerate when it comes to younger people or first time moms they just need to be more considerate. And careful what they say because, like they made it seem like I was just going to get my kid up for adoption after they gave me the risk of him having Down syndrome. They just let it out, you know they don’t have no filter they don't understand how hard it is to hear that, they just want to jump straight to “is it adoption, do you I want to keep him,” they didn't give me a chance to think hmm.

116

00:13:39.180 --> 00:13:41.190

NB: So it was like here's this really- what can be really hard life changing news, and then the next question was like are you going to keep the baby kind of?

118

00:13:49.260 --> 00:13:53.970

[5101]: yeah and then they were like well there is a test we can do, but it has many risks and i'm like Oh, my God there’s nothing actually going to get better in this conversation. I think they were going to put a needle in my stomach test his blood, but I was like, i'm already having problems with this pregnancy and I don't want that.

120

00:14:09.360 --> 00:14:10.050

NB: yeah. I think that makes sense. Or even just causing after you get that news to check in with you.

124

00:14:22.500 --> 00:14:28.680

[5101]: yeah that would have been way better, but I was at the ultrasound, at the place talking to these case worker ladies, there was a run around.

125

00:14:32.340 --> 00:14:49.470

NB: Okay, so let's, this is still all about health care providers. The next question is more about your sexual identity so did the doctors like ask you about your sexual practices or like if you identified as straight, they have those kinds of conversations with you.

127

00:14:51.570 --> 00:14:54.660

[5101]: I’m not sure, I don't I don't think so no.

129

00:14:58.800 --> 00:15:03.780

NB: Like would you want them to like, how do you feel about that kind of -

130

00:15:03.840 --> 00:15:05.430

[5101]: I mean, it would have been like, I felt like a different kind of topic, but I would have answered because I wouldn't have known what it what it had to do with anything, but I would have still answered. I don't have a problem with it, I would have just been confused what I had to do with it. Obviously I need a boy to have a baby so that would have been yeah a little awkward for me, but I would still answer.

133

00:15:26.610 --> 00:15:36.690

NB: So do you think it's like important for them to kind of ask about that kind of stuff or do you think like it doesn't really have as much to do with that kind of care.

134

00:15:38.070 --> 00:15:47.430

[5101]: I mean it depends. I think if it's appropriate to the person like if they're okay as to answering those questions and topics it's totally fine, but some people who don't like to discuss stuff like that if it’s private I don't think should be done, like they should ask consent first before they go along that topic.

136

00:16:02.340 --> 00:16:06.690

NB: Okay that's good, I like that suggestion so say something like what would you, what would you want them to say kind of about consent.

138

00:16:10.650 --> 00:16:16.800

[5101]: You know, usually they give you like in the beginning, they give you a paper you fill it - I don't know if you do know, but they give you, well they gave me a sheet of things I want done, things I don't want, what I was at [place?] I don’t know if they do that at Magee, but i'm sure they give you a paper of things you want done, on the paper. Right, so they you check off thing you want, like just certain things can we ask you about this, consent for touching, stuff like that.

141

00:16:42.090 --> 00:16:43.230

NB: That sounds really cool.

142

00:16:44.640 --> 00:16:45.120

[5101]: It was very cool.

143

00:16:46.620 --> 00:16:56.730

NB: And I can see to how your suggestion to kind of add these kinds of questions would make a lot of sense in that kind of paper thing.

144

00:16:57.540 --> 00:16:59.190

[5101]: Well, because they asked everything else.

146

00:17:03.540 --> 00:17:07.350

NB: Right . So do you feel like you'd be comfortable talking to your, like your pregnancy healthcare provider that you're straight, if would have had sexual contact with maybe men and women, is that something is that a conversation that you would have with them?

148

00:17:20.430 --> 00:17:26.730

[5101]: So if they asked me yeah, there would have to be a reason, I wouldn’t just out of nowhere be like “yeah I slept with these people,” just a conversation, I feel like it has to be a reason they want to know, and then I have no problem with it.

150

00:17:36.270 --> 00:17:45.690

NB: Okay Is there something about - so you want there to be a reason, or like, which makes sense, like to know why they're kind of asking just not them -

151

00:17:46.200 --> 00:17:48.930

[5101]: Yeah I don't want this to be like a “for fun” conversation.

152

00:17:51.570 --> 00:17:52.560

NB: yeah. Right like so okay so maybe. It would be helpful to know why they're having this conversation with you that they're having.

155

00:18:02.670 --> 00:18:04.170

[5101]: yeah that's exactly why.

156

00:18:04.860 --> 00:18:09.480

NB: Okay, so it's not really . Okay, so I want to you're open to talking to them about it but it's helpful to know like why doctors ask you the million questions they ask you.

159

00:18:17.580 --> 00:18:24.270

[5101]: yeah that's all there is to it, I can definitely have no problem answering just I would wonder why it has anything to do with it.

161

00:18:26.490 --> 00:18:40.020

NB: yeah yeah okay that makes a lot of sense I have really liked your suggestions, so far, and I think it helps that you had these two kind of very different healthcare experiences.

162

00:18:41.970 --> 00:18:43.800

[5101]: Yes, *very* different you know.

163

00:18:44.670 --> 00:18:59.010

NB: I yeah it really helps because, like from one, i'm feel like you're getting all these cool ideas and then the other one is also, you know it i'm sorry that you went through it, but it's it's good that, like you can share that information so hopefully other people don't have to.

164

00:19:00.150 --> 00:19:03.600

[5101]: It gave me a sense of expectations. Now know what I don't want to be around. So I’m kind of happy I had those experiences. Never again.

168

00:19:10.200 --> 00:19:11.310

NB: That’s a very positive outlook. Okay, so you want to know why they would ask you these things. And are there reasons that you can think of that you might want to share that stuff with the doctor, like I know you want them to provide reasons, but what are your reasons for maybe wanting to talk about you know your -

172

00:19:36.060 --> 00:19:41.940

[5101]: So i'm sorry I didn't mean to cut you off. Some girls probably only have slept with girls, so it could have been a new experience with a boy or experience that [?]. You know, some people just probably choose girls and try it one time with a boy, and there was the outcome, so they know that I guess it wasn't a planned pregnancy, because I feel like that's very important. You have to know if it's a planned or unplanned pregnancy, because some unplanned I feel like, unplanned pregnancy don’t go right.

176

00:20:12.360 --> 00:20:13.530

NB: Can you tell me more about that.

177

00:20:14.610 --> 00:20:24.000

[5101]: Like me, I wanted to be wild and free and then there's this this baby, I can't drink, I can't smoke, Party, I can't even run for long. And it was just stressful, and yes it was stressful for the baby, but it takes a lot from you as a person, like it takes you like, it takes a lot to do think I am I supposed to keep it, knowing you probably can't even give that baby a life. Because some people who don't plan know they can't give that may be a good life, so why do I sit here and even try to, and it probably can go wrong, so I think that part is a really big part of the conversation too. They need to start asking is it planned or unplanned because you can’t expect the best from an unplanned one I think.

182

00:20:59.580 --> 00:21:05.880

NB: So maybe there'd be some different conversations around whether someone said it was unplanned versus planned.

183

00:21:06.720 --> 00:21:19.770

[5101]: yeah I think I always thought that was important because it just seemed like it's a big factor, like you, don't know if this person wanted this kid, or was forced to have that kid, like I think that's very important to know that

184

00:21:25.620 --> 00:21:26.700

NB: What are things that - and you might have had some experience with this - what are things that would make you not want to talk about the, like that you've had sex with men and women and identify as straight, like what could a doctor say that you'd be like, “Oh no i'm not talking to this person.”

186

00:21:44.280 --> 00:21:52.170

[5101]: It’s actually funny and I wasn't really like mad about it, but like when was in [millvale?], so I explained that I actually, and she’s a really good doctor and we’re actually having a conversation, it was along the conversation of planned or unplanned, and I was like you know I don't usually sleep, I’m not sexually attracted to females. I'm like I can't touch them, but I can be with them, and she’s just like laughing, she’s like doesn’t that make me like bisexual. No because I don't touch them, it was a funny conversation, but I’ll never bring it up again because I’m starting to notice that it's weird you saying you're attracted to women, and you will sleep with them, but you don’t identify yourself as bisexual or gay, it just doesn't sound right to many people, like what are you.

191

00:22:31.050 --> 00:22:31.560

NB: yeah. I mean, I think that you can identify as whatever you want it's literally none of my business, how you identify yourself. Except for this -

194

00:22:41.010 --> 00:22:41.490

[5101]: yeah.

195

00:22:41.760 --> 00:22:43.080

NB: yeah well, so you. Like so they did ask about that.

197

00:22:48.510 --> 00:23:00.600

[5101]: It wasn’t like a part of like the conversation she was more of just making small talk, while I was doing, she was trying to distract me from getting my cervix, from her checking for how much I was dilated. She's trying to distract me because I hated that part. So she was trying to have a conversation, like “so was the pregnancy planned?” it was that type of conversation it wasn't like it needed to be asked about attraction, which did not work.

201

00:23:15.360 --> 00:23:16.500

NB: So do you feel like, you know, since you identify a straight, but have had like sexual relationships with both sexes or genders, do you feel like that's one reason why it's like that affects the conversations that you have that you might have with doctors, because you don't really fit into like a box.

203

00:23:34.140 --> 00:23:42.150

[5101]: yeah I think it does because she automatically expected me to say bisexual, but I would never want to say that. It was a funny conversation. And yeah I didn’t take it wrong, and it was just confusing, because she's looking at me like “so you're straight?” and i'm like “yeah basically.” Mostly straight.

206

00:23:55.200 --> 00:23:59.250

NB: Mostly straight yeah because it's a very it's a spectrum, we know that so it's like people fall all along it not just straight or bisexual or a sexual there's a million different.

208

00:24:10.380 --> 00:24:10.890

[5101]: yeah. It was a funny conversation.

210

00:24:15.480 --> 00:24:16.860

NB: How do you mean a doctor could like bring that up, like how could they have what would you want them to say to you?

212

00:24:21.390 --> 00:24:25.710

[5101]: That I really don't know, I mean the way she went about it was like. So she was like she basically asked him like “was it planned or unplanned,” and I was like “not really, you know I don't really like him that much,” and she was like you know “what's wrong,” you know. And I don't know Dudes are weird, I really I was I was joking, saying I would just date a girl before I dated a girl. I mean I dated a girl before I dated a dude. And she was like really? Yeah, which is, we had a conversation about it. But I don’t know how a doctor could bring it up on a professional level because they were just on a joking level. So professional wise, I really don't know, I think it would have to come up in small talk, because I feel like straightforward I wouldn't know how to word it.

217

00:25:07.590 --> 00:25:20.340

NB: Right, especially because, like I think it's so, one of the things I think that you're sharing that's really important from this interview, is that, like a doctor could have asked you like, “How do you identify?” and that one question just isn't going to actually capture kind of how complicated like the question actually is, for most people.

219

00:25:30.720 --> 00:25:33.150

[5101]: yeah because I mean i'm not really. It don't really it doesn't affect me, okay? I don't really look at it as bad, but there's some people out here like that, you'd be surprised so there's probably many reasons people probably get judged and i'm pretty sure the solid reasons why they don't want to talk about it, but me personally, I just let it be free.

222

00:25:54.540 --> 00:25:59.700

NB: yeah there is a lot of reasons people don't talk about it, especially with doctors, which is

223

00:26:01.320 --> 00:26:18.420

[5101]: Because some doctors, probably, I mean they’re still humans, I mean you’re a doctor but they still have feelings and beliefs, and some people are probably Christian and don’t believe, you know , in the LGBTQ, so they may look at you different or they may try to talk you out of it, some people don't have like a sense of respect. Like they don’t even notice how wrong they're saying it or how it's coming off.

Like wow who made you gay or what makes you like that, you know it just doesn't sound right when you're telling a person who is gay that sound like you're judging them, so I understand what you mean by that.

226

00:26:36.390 --> 00:26:44.370

NB: yeah that does sound judgmental for someone to say like “what made you like this,” like what makes you straight and who knows?

227

00:26:44.940 --> 00:26:45.300

[5101]/; Yeah, exactly

228

00:26:47.910 --> 00:26:58.020

NB: Are there things that Okay, so your doctor kind of brought it up in casual conversation to distract you from the cervix thing, which I know that gynecologists do that's like a common move I feel like to try to like have a normal conversation with you, while this crazy.

231

00:27:04.530 --> 00:27:05.580

[5101]: Yeah, I hate it [laughs]

233

00:27:08.820 --> 00:27:18.150

NB: Do you think there's any resources that would be helpful, did you get any sources about like sexuality and like sexual habits and stuff like that.

234

00:27:18.570 --> 00:27:24.840

[5101]: Well like I said I was in a place called Bethesda, a mommy and me program like a home and they do groups, like one at the end of the day, around eight o'clock you have a group it's about STD, it’s about different things, that came up, sexuality. Just all types of groups, but that was one of the groups, but it was different on when they say LGBTQ, they say LGBTQIA, they like they added it to the LGBTQ, IA so plus more and it was just basically talked about it, and of course it was

240

00:27:56.160 --> 00:27:57.480

NB: You went on mute for a second.

241

00:28:02.490 --> 00:28:03.000

[5101]: Can you hear me?

242

00:28:03.180 --> 00:28:04.110

NB: yeah I can hear you.

243

00:28:04.920 --> 00:28:08.850

[5101]: Okay yeah, but of course there was somebody in our group was like “i'm not you know I don't believe in that.” But we do have them groups, we just can’t have them for long because people are..

245

00:28:16.110 --> 00:28:17.820

NB: that's frustrating, because I feel like the group. The whole point

247

00:28:22.470 --> 00:28:22.770

[5101]: Is basically for that, yeah.

248

00:28:23.610 --> 00:28:27.150

NB: yeah so that's interesting that someone, that's unfortunate that someone did that.

251

00:28:33.420 --> 00:28:39.480

[5101]: I mean, that’s how she was raised, so I was like well that's a bummer rather, gonna have hate in your heart for no reason.

252

00:28:42.930 --> 00:28:44.430

NB: Did the group, like. So that was there any part of the group, like stuck out to you like that you thought was helpful or

255

00:28:55.230 --> 00:29:06.810

[5101]: The most, it might be weird but the most helpful part was like STDs like STDs that came along that. They always think like gay dudes are so high risk for it, or the only person who can get it, but they made it like, females can get it too, lesbians can get it too, bisexuals can get it too, it’s not always just males like you can get it, and that was a good important part, because, I’m not gonna lie my mind was just stuck on only you know dudes who are gay, you know they are only person who catches STDs. Which is dumb. But they explained it like that’s not what happens.

260

00:29:33.600 --> 00:29:42.120

NB: yeah that is really important, and like bisexual people that identify as women are like really high risk or sexual health stuff.

261

00:29:42.870 --> 00:29:45.960

[5101]: Yes, and I was like I didn’t know much, like what there's no way but even lesbians can get it, I didn’t know that.

263

00:29:50.730 --> 00:29:53.340

NB: yeah it's great to know stuff like that.

264

00:29:54.300 --> 00:29:57.780

[5101]: Yes, he was I was shocked the whole time, so great to know that

265

00:29:59.310 --> 00:30:04.170

NB: yeah, the more you know it's like, yeah the better, you can go into situations prepared.

266

00:30:06.450 --> 00:30:07.680

[5101]: And I was prepared, kinda.

267

00:30:08.490 --> 00:30:15.960

NB: I like that I was really helpful for you to share and like we're talking about sexual health stuff and sexuality so like it will take a lot to weird me out just so you know.

268

00:30:18.030 --> 00:30:22.800

[5101]: Okay i'm just like it's a new conversation for me i'm very open minded, I never -

269

00:30:24.420 --> 00:30:29.820

NB: yeah these interviews are my favorite like I really like this part of the study it's like a smaller part to the big study, but I really I think this stuff is like really important and interesting so like and also just generally like i'm pretty hard to offend and I’m really hard to weird out so.

272

00:30:43.530 --> 00:30:44.070

[5101]: that's a good thing. I don’t know how to I don't know if i'm saying lie lesbian, gay or straight, that's just how I talk and I want to be blunt, but I saw like I say it, I don't know if there's a different term you would like me to say, other than gay you know I don't it sounds harsh kind of.

275

00:30:59.310 --> 00:31:01.020

NB: It all works, gay is great you know. Okay, so this one's kind of I always think of this question as a little bit more like abstract kind of like so like you know you identify a straight you've had sexual contact with [blah blah blah] What was it like for you like in your body pregnant as that person does that make sense?

277

00:31:27.210 --> 00:31:28.740

[5101]: Will you like say it one more time.

278

00:31:29.100 --> 00:31:35.340

NB: Sure, so how did like your sexuality or sexual contact, how did that affect what it was like for you to be pregnant.

280

00:31:41.160 --> 00:31:54.930

[5101]: Well, it didn’t really affect me. I mean really, like I don’t know how to explain it. Like i'm more of a like a tomboy so it was hard, like you got a big stomach out here trying to wear timberlands and a hat and it just doesn't look right. So it didn't affect me, I don't really care what people think, but it was hard, you gotta put on maternity clothes, put away all your normal jeans, and then the closer to like the where, am I, you know sweat pants were were the only thing like I could wear. Maternity leggings were so expensive I would just wear a dress the whole time.

284

00:32:15.240 --> 00:32:27.120

[5101]: So that's, the only thing, not relationship wise or anything, it was just looks-wise like because i'm more of a tomboy and I like to be rough and it was like my clothes did not fit. My style wasn't right, I had to wear a dress 24/7, I barely wear dresses. It was weird I felt like. I don’t know how to explain it. I was weirded out most of the time.

288

00:32:39.660 --> 00:32:43.110

NB: That makes perfect sense for me, I have yeah like you know I think sometimes like I don't think about the ways in which, like I feel most comfortable kind of like with my like gender representation, or whatever it is. Like it's not that i'm like trans or anything but like when that doesn't match up with how I i'm most comfortable I can feel it. And we don't see a lot of tomboy like representation pregnant.

295

00:33:14.550 --> 00:33:17.550

[5101]: [garbled] could have been tomboys, just straight like, I don’t know, like you wanna be a boy. I think tomboy, is just a girl you just don't want to dress all girly

297

00:33:24.750 --> 00:33:26.220

NB: yeah yeah.

299

00:33:28.290 --> 00:33:35.520

[5101]: At least that’s how I do it. I still like being a girl I just don’t like to put on dresses skirts and stuff that's all.

300

00:33:36.600 --> 00:33:39.000

NB: yeah and it's hard when like that tomboyish style that is like you identify with is harder to find when you're in a pregnant body basically.

302

00:33:48.450 --> 00:33:59.040

[5101]: Yes, all I kept finding was mom jeans, no offense to myself, it was leggings, and I was like no I don't want none of this. And it was horrible for me I don't like the color of jeans, they had no like light blue jeans or dark just, mom jeans. I hated it. For a long period of time. I didn’t feel like myself, like that part, me changing, I feel like was the worst part about my pregnancy. I feel like I was a whole new person.

306

00:34:29.250 --> 00:34:32.910

[5101]: If could redo it, I love my son, but I would do it different..

307

00:34:35.430 --> 00:34:45.120

NB: yeah I mean I think it's important if you, you know, like how you dress and like how you look and then also and you can’t kind of do that stuff that is that can be so hard.

308

00:34:46.290 --> 00:34:48.780

[5101]: yeah very hard, especially in the program I was.

309

00:34:55.320 --> 00:34:58.920

NB: Alright we're going to change gears a little bit okay you ready?

310

00:34:59.910 --> 00:35:00.630

[5101]: i'm ready.

313

00:35:06.480 --> 00:35:15.090

NB: So this next section is all about, so the last two sections, one’s marijuana and one’s tobacco and the questions about them each are basically the same. And then the last after those sections are done i'll ask about like your kind of like perfect world, and then we'll be done the interview, I just want to tell you what's next so you know. And then I like to remind you that we're not going to share these like drug questions with anyone your doctor, anyone.

318

00:35:36.870 --> 00:35:39.750

[5101]: i've already showed them before my caseworker and probation officer, so you couldn't show anybody who doesn’t know already

320

00:35:43.530 --> 00:35:44.610

NB: We won't do that don't worry. Your doctor’s not going to hear it.

322

00:35:48.180 --> 00:35:54.630

NB: So I think on the screener it said that you have tried marijuana but you stopped smoking, for your pregnancy is that right ?

323

00:35:55.290 --> 00:35:56.130

[5101]: yeah I smoked a lot a lot a lot. But I stopped.

326

00:36:02.910 --> 00:36:03.930

NB: Okay perfect.

327

00:36:03.960 --> 00:36:05.340

[5101]: I was very hard.

328

00:36:05.880 --> 00:36:06.540

NB: Can you just tell me a little bit about - and i'll ask you like about like how quitting was for you, but could you tell me a little bit about like the first time you use marijuana like paint that picture for me.

331

00:36:18.780 --> 00:36:40.410

[5101]: um first time it wasn't nothing I think it's all your head, because until I like wanted myself to believe I was high, only thing was was my eyes were low, and I was just like well, this is just like normal, and when I started to believe, like “you're high, you're smoking” that's when the high kicked in. Like it's all I was, it was all in my head. Like when I wanted to believe it was high, it’s weird like.

It sounds like who wants to feel like this, but it's like you're in a cartoon, first time ever experienced being high, I felt like I was in the movie. Like I was just a different person out of body experience I was happier I could laugh, everything was funny, I was hungry as heck, but I was just a different me like. I wasn't sad and depressed and anything that would have been sad and depressing before wouldn't have been depressing to me at the time, I could just laugh it off, shake it off. It’s just a good feeling for some people who need it, like you're just you're there but you're no,t you're more in your head, music sounds better.

Sometimes, not even saying. it depends on how I felt. like literally like and I cannot say, but when I feel like it's like it just makes you just free person like I don't know, like my voice will sound different and everybody around me will just. I don't know it just made me look at life way more. Like why was the star so far, why can't we just go to the moon, and just go up there, and just touch it without having to you know, like just made you just think. It’s weird to explain. I don't know, like how can we talk through the phone without a cord

341

00:38:03.990 --> 00:38:05.190

NB: I think you're making perfect sense. What I heard was like I mean you said a bunch of different things, but the one the two things that i'm like picking up the most was kind of like it just shifted first how you felt about yourself, and then also brought things to your attention that are maybe amazing, but that we don't think about as being amazing like talking without a cord, or like you know what I mean.

345

00:38:32.640 --> 00:38:33.000

yeah.

346

00:38:35.640 --> 00:38:39.090

[5101]: yeah. Some questions I couldn’t believe I was asking myself, but while I was questioning I didn’t really care, but it was beautiful to me I really didn't miss it, but it got old.

350

00:38:52.020 --> 00:38:56.340

NB: Sound beautiful. What did you used to smoke like did you do like bong or like vape, or

351

00:38:57.300 --> 00:39:03.420

[5101]: I mean, I never really did a, I wasn't into the vape thing. I used to smoke, well you could say it was a blunt it wasn’t really a blunt but it was leaves, woods. I didn’t smoke like actual blunts but it was a blunt technically. I rarely ever bonged, I don’t like that, it burned my ungs so bad. It’s more smooth when you do a blunt. That’s my main thing out every day, unless I didn’t have a blunt, then I bonged, which was a waste of money and weed but still did it.

358

00:39:38.460 --> 00:39:41.490

NB: yeah everybody has a different preference for what they like. Okay, so now well, let me think about this Okay, so you kind of already told me about what you enjoyed about using marijuana like you said it was like beautiful. Was there anything that you didn't like about it.

362

00:39:59.760 --> 00:40:09.120

[5101]: um money. like God I became broke. Then it's like when you just smoked and smoked. Like weed give you the munchies, you just want to eat, but if you're like to the point where you smoke every two seconds every time you wake up you no longer want to eat. All you want to do a smoke, the first thing I used to do when I woke up smoke, where I will wake up in the middle night what did I do, I smoked. It it's kinda like and if I didn't smoke, the worst stomach ache. I hate to say this kind of like crackhead. I was like God what's wrong with me, like I was changing like weird ways and I felt like at this point.

Because like I was losing weight, it was just taken over my whole life, my money was going down, if I didn’t have weed I was angry person. Like there’s cons to weed and nobody notices it like, if I didn’t have weed, I was like well guess I’m gonna be mean to the world. Like it was like my calm and a very expensive thing to help you be calm. If you didn’t have the money, I was angry.

368

00:41:02.070 --> 00:41:03.300

NB: yeah yeah.

369

00:41:03.600 --> 00:41:09.720

[5101]: it's like any other drug and I don't think people notice. it like people think that weed is so different from meth. It’s really not. It’s really not. Like it's really not because I know people who will do anything to get some money to buy some weed, it takes over your body like anything else, it does and just in different ways, it might help you but.

Some people might have medical weed but, like the ones that are on the street, you don't know and then definitely take over your whole body if you smoke a whole bunch yeah.

372

00:41:33.210 --> 00:41:34.230

NB: yeah I think you're very right. I think a lot of people don't think of it as a drug, really.

375

00:41:43.680 --> 00:41:48.660

[5101]: They think of it as calming. Maybe the ones in the hospital, CBD and all that, but not what they’re doing.

378

00:41:55.740 --> 00:41:56.190

NB: So what wa.s Do you remember any times, where you like really craved it?

380

00:42:02.130 --> 00:42:02.670

[5101]: Yes. When I first ever got pregnant, I was just so depressed and my life was over, I kept telling my life over, I wanted it to do it so bad like, in the moment I knew I was losing my mind.

But I knew I felt like I got high my son got high and i'm like that's horrible like I just thinking about that made me cringe like if you get high your son will get high, and I just couldn’t do it.

I wanted it so bad but I just couldn't. Like I couldn’t stomach the thought about the smoke and your baby inside you, nothing but, I just couldn’t do it. That was my depression point.

387

00:42:52.530 --> 00:43:03.780

NB: I can see why it would be so hard to you know you got this life changing news and if you're used to smoking often and you can't you also can't do that anymore like that would be super hard. What was it like, can you just talk to me about what it was like to quit for you, during that time?

389

00:43:14.970 --> 00:43:17.040

[5101]: um it was hard. It’s what I used to do. I needed a hobby I needed something to do other than smoke, like that’s what I used to do you know. Like smoke, go to sleep, wake up, smoke, go to sleep, wake up, and without that I just sit there and I just I needed something, but I like to read, so when I started reading I mean that took it over, but only for a while. Reading was my hobby, my phone really didn't get me take my attention, but reading did. So I just start reading, my aunt brought me books. I was antsy because weed calmed me.

Like when I was in school I used to smoke before I went to school, so I could pay attention, I could focus because i'm just in my head the whole time so it's nothing but the focus so when I didn't have that it was just antsy, I didn't want to be there, I wanted to leave, I wanted to just walk away like I don't know. There were times, where I needed it bad. That’s what I used to do said, [ unclear: my dress cane s unless you want to say..]

I don’t know it calms me, but I couldn’t do it no more, for my son.

400

00:44:40.140 --> 00:44:40.440

NB: yeah. what kind of books, did you like to like what's your favorite thing to read that's not in the interview i'm just curious.

403

00:44:51.180 --> 00:44:51.660

[5101]: um. it's going to sound strange, but I like like urban books like yours it's where my favorite one was when I read. I read Twilight, I was so mad by the second book of the second book was nothing like the first one.

And I was like this sucks I just want to start reading it or not, they kept telling me keep going it gets better third book gets better I just really did not want to read the second book because i'm into it then it’s just black nothing but her being depressed and i'm like this is horrible. The first book was the best, and this is real man I just I read books like that, like vampire diaries I feel like reading that are so many books me too.

412

00:45:36.000 --> 00:45:38.610

NB: I like fantasy, it's very nerdy I know that I really like it.

413

00:45:39.330 --> 00:45:47.970

[5101]: Harry Potter books are better than the movie, I like them and I can't remember the book that I read it was about magic and it was about a vampire. I cannot remember book that was also a really good book by I love that book, it was a series that only read one I wanted the second one so bad, so what.

415

00:45:58.020 --> 00:46:08.460

NB: i'm going to email you this like what I think is one of the best like science fiction trilogy is kind of it's like it is just like it's so good oh. it's amazing it's my favorite book and it's kind of it's all about kind of like it, like everything is also about like like you know how there's so much oppression and racism here but it's in this like crazy well thought out fantasy world it's really good. I'll send you the i'll email you.

420

00:46:31.170 --> 00:46:35.310

[5101]: Thank you, because I haven’t had any good books in a little bit. [Baby] can barely even sit down.

421

00:46:35.910 --> 00:46:38.610

NB: Oh that's true I bet you're like 100% baby.

422

00:46:39.270 --> 00:46:43.560

[5101]: Yes, i'm just trying to do this interview with you, literally as soon as I turnd my camera on his whole head just turned kind of. That’s my luck.

424

00:46:48.450 --> 00:46:52.680

NB: there's usually like babies or like older kids in the in the interviews.

425

00:46:53.460 --> 00:46:57.000

[5101]: But he I told you, a lot of the time he took a nap, but I jinxed it because as soon as he woke up we were on a call and I’m like oh

428

00:47:01.770 --> 00:47:04.800

NB: he's been pretty good i've heard him talk a little bit, but.

429

00:47:05.190 --> 00:47:08.070

[5101]: yeah I made him a bottle, he’s in there watching a word party. Trying to make me something to eat. Well, there he goes, I jinxed it

432

00:47:23.520 --> 00:47:24.540

[5101]: You drank your bottle fast, yeah because you’re spitting it out.

433

00:47:40.440 --> 00:47:54.120

NB: So there's this thing that they call the Gray period which is like when you were maybe pregnant, but you would have had no idea that you were pregnant, is there a chance that you do you think you like basically accidentally smoked marijuana during that period.

434

00:47:54.210 --> 00:48:00.360

[5101]: Oh yes, I accidently drank during that period, I was two months pregnant when I find out I was pregnant. So yes, I had a big period of nothing but smoking and drinking until i'm like wow you're getting fat. Then I was like what’s wrong with me? I had major heartburn i'm like this is this so like what's going on my body that's where my I was like what did you do today and I hate thinking about my period, because I never want it to come. So let's not even think about it and i'm like whoa if you think about it, you really didn't get it like two three months. That's a good thing right? No it's not.

443

00:48:35.700 --> 00:48:37.530

NB: Like I hate this thing I don't want to get it

444

00:48:38.610 --> 00:48:41.190

[5101]: yeah but I wasn't thinking about it. Carefree.

447

00:48:49.200 --> 00:48:52.740

NB: A lot of people's periods are really unpredictable to so like people don't really know.

448

00:48:52.830 --> 00:49:01.380

[5101]: yeah I had very abnormal ones are also I wasn't thinking in my mind and come one month skip two weeks it just depends on how my body feels like.

449

00:49:03.150 --> 00:49:10.050

NB: yeah I think a lot of people, you know smoke or drink or live their normal life during that grace period because they don't know.

450

00:49:10.770 --> 00:49:18.300

[5101]: I felt so bad when I was in hospital because they're like they pee tested me, and this is also what I hate about magee, they pee tested and they wouldn't tell me that pee tested me um they just kept on they're like this is procedure, but they kept asking like do you smoke, do you smoke. I’m like why do they keep asking me if I smoke and I say no, and hey keep testing me each time I come here. So finally I went to [other location] and they told me that, because your first ever pee tested positive for marijuana and i'm like well finally somebody told me because, y’all didn’t it. I wondering why they kept asking me any time I had appointments at Magee they kept asking me if I smoke, I ould say no, ask me again and I say no, like what do you want me to say, yes? And now I know why, because my first one came back positive, which I feel like would be understandable, but I guess it wasn’t..

455

00:50:05.790 --> 00:50:08.070

NB: yeah I would I want to know is like would you ever feel comfortable talking could a doctor make you feel comfortable enough with them to talk about marijuana use during pregnancy?

457

00:50:20.280 --> 00:50:21.180

[5101]: Oh that's a hard one. It depends how they come, it will have to be how like you just said it like you didn't know , because there's no way i'm going to smoke long time and now we're going to go to automatically feel targeted i've been there so many times.

But if they bring it up how you just bring it up like the gray period, like you said, I will feel more than comfortable to say, because I was ashamed that I found out that I was smoking, but yeah. Don’t hide it though, I hate when they hide it like we think there's a reason I keep tricking you, that was annoying

462

00:50:51.840 --> 00:50:57.090

NB: They should, so did they just drug test you without informing you that they were doing it

463

00:50:57.090 --> 00:50:58.560

[5101]: Each time. Each time, they did it because they kept on, and i'm like, why do you keep doing that, and then I did asked because, it was [?sounds like wasn’t the same at another medical center?] They did it one time and then I was like bam I was done.

466

00:51:15.510 --> 00:51:26.910

NB: And I wonder to how you would have felt if they had been, like to circle back to what you said earlier, with like the consent and like asking like I wonder how you would have felt if they had been honest with you.

467

00:51:27.330 --> 00:51:39.390

[5101]: like if they would have been honest, like you know it came up the first time we just want to make sure you're not doing more, that is understandable, but just tricking me thinking I’m gonna lie, That was what had me kind of upset. Just be honest, I just want to repeat it, because you want to make sure, Okay, no that is normal, but not tricking me.

470

00:51:57.330 --> 00:52:15.510

NB: What is this asking um alright so sometimes people that you know identify as LGBTQIA have specific things that affect their marijuana use like discrimination or you know stigma. Do you think that there was anything out your sexual identity and practices that affected, how you used marijuana?

472

00:52:24.990 --> 00:52:29.550

[5101]: Not really I mean I smoked around boys, a lot more than girls, but that wasn't anything to do with my sexuality, they were just easier to get along with.

Well, I guess it does, because we. I smoked around girls before, they’re kinda weird, I think they get the I like girls part wrong, I said I like, not want to touch, so they would get that part little misunderstood. So yeah because they would take what I said completely wrong. I like from distances, not up close. They would think it meant up close and personal. So that's why, I mean i'm doing it because, when they will do that I’d get so mad. I was disgusted. They would not understand why, but like I understand why. Because I may be – I don’t know I don’t I think [identify?] as straight, because. I’m starting to throw people off.

Really i'm starting to think something, like it's my kind of my fault, I never should of said I like girls, I should just say I’m straight.

486

00:53:37.590 --> 00:53:40.320

NB: Yeah. But it's also good to be honest about you know.

487

00:53:42.300 --> 00:53:43.020

[5101]: But they take it wrong. They think I said that meaning, no. And that's wrong completely wrong.

489

00:53:50.370 --> 00:53:54.450

NB: yeah hard to control what people, like how they react.

490

00:53:55.620 --> 00:53:57.360

[5101]: yeah that’s why I’m gonna just say “nope, straight.”

491

00:54:01.170 --> 00:54:10.980

NB: How do you think i'm pregnant women or people can be supported to quit using marijuana like, how can we help them.

493

00:54:15.240 --> 00:54:26.130

[5101]: Think about how much money is being taken away like you have to bring that up, like you have to because anything else, nobody listened to, like, I remember my stop weed talks and what really got to me was the fact that it's a drug, and I’m like you know this drug is helping me, that's what they think, “it's helping it takes some stuff”

495

00:54:35.010 --> 00:54:50.640

[5101]: To talk about feeling, saying that it can hurt you, people won’t listen to that. Because in their mind it's helping, so nobody i've never really listened to nobody when they said that, like that's not a way to start off the conversation, because a person that smokes weed a lot is definitely going to disagree with you

496

00:54:52.320 --> 00:54:55.500

[5101]: yeah that's important

497

00:54:55.770 --> 00:55:08.820

[5101]: You're not gonna agree that weed isjust messing them up. I think, the money part of weed is what is what could possibly help, but it's up to you, I don't know if I you can, like weed is very addictive, I never thought it could be, very addictive.

So the brain thing, to like your brain cells, my girl, she said, like I said, if somebody smokes weed a lot, it helps, so that does not get to a person who smokes weed, you saying it affects the brain and nobody's gonna listen to that because to them it helps.

499

00:55:28.500 --> 00:55:32.760

NB: I think you have such important insight like. Really, I feel like you're This is like going to be it yeah your interviews, is just so great, because you really have like you have like experience, but you also have this like clear-headed understanding and communication about it, so I have really appreciated your honesty and like your willingness to share with me so far.

502

00:55:55.680 --> 00:55:56.430

[5101]: Yes, because. I'm just, It just seems like something that people need to know about they don't, they just take it.

504

00:56:06.780 --> 00:56:12.360

NB: We don't talk about a lot of stuff, and this is one of them, even though it's like very common in a lot of people's lives so.

506

00:56:13.860 --> 00:56:23.160

[5101]: I think the big picture, should be illegal, like, I feel like that should be the big jaw dropper but it's not it's not the big jaw dropper, it’s illegal, like everybody’s just like “yeah it’s illegal, ok”. That should be the only thing somebody has to say, like you shouldn’t smoke because it's illegal, but no that still doesn't work.

That's what I feel like somebody should have to do, like say you shouldn’t smoke because it’s illegal, and then you’re right it's illegal but it doesn't go like that. People are like Oh well, it's illegal to do it, total opposite of what you expect.

509

00:56:42.420 --> 00:56:49.980

NB: And I think that we're changing in how we think about it being like I think it's getting closer and closer to being legal everywhere, too.

510

00:56:51.780 --> 00:56:55.740

[5101]: Yeah, it is. Some people don't use it for right reasons

511

00:56:57.150 --> 00:56:57.510

NB: Sure. No that's not an argument for it. It's just this thing that's been happening, I feel like.

515

00:57:04.740 --> 00:57:07.230

[5101]: I wonder if they if they actually will.

516

00:57:09.780 --> 00:57:13.980

NB: I don't know Pennsylvania, is an old fashioned state.

517

00:57:15.600 --> 00:57:16.230

[5101]: yeah I wonder. I just always looked at it as illegal and nobody will ever take it serious.

520

00:57:26.760 --> 00:57:28.080

NB: I know it’s legal in like DC and.

521

00:57:29.010 --> 00:57:33.270

[5101]: yeah I used to go to the vendor out there. Very nice lady..

522

00:57:37.290 --> 00:57:39.990

NB: Okay, so before we move to the tobacco questions like do you.

523

00:57:41.730 --> 00:57:47.730

NB: Do you have anything that you want us like me as a researcher to know about marijuana use during pregnancy. what's important.

525

00:57:50.640 --> 00:58:06.990

[5101]: A lot of people use it let's just say that. a lot of people smoke, while using a lot of people smoke, while pregnant and it's very bad like and they only smoke, because they think that it's okay.

I think that's very bad, believe it or not, so many people smoke while pregnant it so like i've been around with women who just do nothing but smoke and it's like a tiny little population. A nice good amount of the world smokes while pregnant.

527

00:58:21.960 --> 00:58:23.910

NB: I agree with you, yes. Yeah it's more common than we think, which is why it's like we got to talk about it, we got to figure out what it really does.

529

00:58:32.460 --> 00:58:35.730

[5101]: Yes, like it oh I can't believe how many people smoke. I felt like a loner.

531

00:58:40.830 --> 00:58:53.880

NB: I think that, I mean are like the funding for this study is coming from that fact that you just said that we're finding out more and more people are smoking, while they're pregnant and so we need to know more about it.

532

00:58:55.020 --> 00:58:55.560

[5101]: Yes.

534

00:59:03.990 --> 00:59:15.210

NB: we're going to go through the same question, basically, but for tobacco, so we just start with like when was the first time you tried tobacco either cigarettes vaping, chew to whatever you know.

538

00:59:32.910 --> 00:59:33.780

[5101]: [talking to baby]. First time I ever, I was like [four?], I didn’t know how to inhale a cigarette, [talking to baby]. I don't even know where I was going. Well my mom smoked, it was cool, and I did it. It didn't affect me at the time, because I didn't even know how to inhale, I was just wasting cigarettes.

540

00:59:49.440 --> 00:59:50.190

NB: mm hmm.

541

00:59:50.760 --> 00:59:52.770

[5101]: And then one day I got caught. I stopped smoking after that. But when I learned to inhale, that’s when it became an addictive thing

543

01:00:05.670 --> 01:00:09.780

NB: yeah Do you remember when you realized, like it was an addictive thing?

544

01:00:15.660 --> 01:00:17.730

[5101]: When I went to the mommy and me program, I’m like whew. But I don’t smoke cigarettes, I vape. But yeah when I went there. That was around January to November 5 of last year. That’s when I realized that. Like I do. I need to vape. I mean I was dumb enough to order one and get caught.

550

01:00:46.020 --> 01:00:46.710

NB: you're not allowed to vape there?

551

01:00:51.420 --> 01:00:55.710

[5101]: Not pregnant, no. There’s a placement but I wasn’t in the placement part because I was pregnant. They put me with a group over in a house and it’s actually a very nice house, but it's a House it's really a placement, it’s basically for juvenile delinquents, so you go there and you’re in there and it’s a lockdown facilities. Like a kid jail. Some people call them. It’s a placement, but I didn't go to that part because I was pregnant. But if wasn't I feel like I would be at that part.

558

01:01:29.430 --> 01:01:30.120

NB: So no vaping in Bethesda home.

559

01:01:31.830 --> 01:01:32.790

[5101]: Now, no not at all. Even really we used to go out weren’t allowed to be around people who smoke, weren’t allowed to go to parks, none of that.

562

01:01:43.800 --> 01:01:46.590

[5101]: wow yeah they were on it. They would come in, smelling like a whole cigarette. But that’s besides the point.

564

01:01:51.420 --> 01:01:54.000

NB: yeah do you vape right now?

565

01:01:54.990 --> 01:02:00.270

[5101]: Yes, sometimes. I don’t like being around [baby] though, so rarely. When I get a break, to get away and run.

Because he does stuff like this, let me see if I can show you. This is why I don't get breaks, because he does this when I walk away.

569

01:02:17.850 --> 01:02:20.130

[5101]: I don't even know how he got to that place this kind of. [baby] Get out, you’re going to fall [talking to baby]

571

01:02:28.980 --> 01:02:29.760

[5101]: So not a lot. I vape every other day, when I get a break from [baby], time to get away.

573

01:02:36.150 --> 01:02:37.080

NB: that's not a lot. What do you like about it ?

575

01:02:44.490 --> 01:02:49.410

[5101]: Well, I have all heart [unclear?] from smoking and it calms me. I thought it was some not having nicotine thing, but no. Weed gave me like this, like now if I try to smoke weed, it makes my heart beat really really fast. Like I’m going to have a heart attack. That's another thing about weed, it feels like I’m about to have a heart attack and it happens to my brother too, but he still smokes like he just wants to kill himself, but that's what the vape does, it calms my heart. Like my nerves are really bad because of smoking weed and like, if I smell weed, contact high, my heart will go like I’m literally about to die. You can see it, my grandma said it to me one day about to go to the hospital. She didn’t know, She was when [unclear? she was alive, wrongly, that we That was the mother always tells me personally] try to calm down but yeah that's what nicotine does, it stops my heart beating like crazy.

583

01:03:42.660 --> 01:03:47.220

NB: It calms you down a little bit like marijuana used to but marijuana now kind of makes you.

584

01:03:48.510 --> 01:03:52.350

[5101]: yeah yeah like it traded, now it’s trying to kill me it seems like.

585

01:03:53.340 --> 01:03:56.880

NB: yeah yeah like it causes like panic now.

586

01:03:57.630 --> 01:04:06.810

[5101]: yeah that's what I thought it was like, panic attack, but if it's not in my head, because I have no control over that body, I mean anytime I would try to like breathe, some water, just calm down, my heart would just. I can still take a step back and think about it, something else, my heart will still go.

Now, if I run too fast, my heart will just not ever slow down, it takes about an hour two hours from my heart just slow down. If I dance too fast, that’s another thing, it’s just messing with your body

590

01:04:36.840 --> 01:04:37.200

NB: yeah.

591

01:04:41.220 --> 01:04:42.000

[5101]: I love coffee but that does it too

594

01:04:55.620 --> 01:05:05.460

NB: So you were in this Bethesda place, and so did they like force you to quit did you have to quit when you were when you found out when you were pregnant like what.

595

01:05:05.550 --> 01:05:05.940

[5101]: well. When you go to Bethesda, they make. The thing is, I didn't tell them because i'm not no dummy. People go there thinking they're smart and hard and want to impress anybody and then their time is longer because you have to do drug and alcohol.

No, I went in there like nope i'm a good girl I don't smoke I don't drink nothing, so when I told them that kind of played myself, because I went and ordered a vape and you don’t smoke you don’t do that. But before that, I told them I didn't. I was like no I don't, you know I don't like the smell of cigarettes, I don’t like nicotine period, , and I didn’t have to do drug and alcohol so my time was still long, but you get longer if you do drug and alcohol.

And you got to be millions of groups and got the pass drug and alcohol class. See they’re idiots, but I’m the idiot who ordered a vape. I don’t know. I think they you have to tell them and they go from that but they don’t drug test you to find out if that makes sense.

Other than if you go on a home pass, when you come back that's where you get pee tested. If you go on the home pass you know you're around all types of stuff, they pee test you when you come back. But you don’t have to tell them, but they choose to and they stay longer.

602

01:06:19.830 --> 01:06:24.420

NB: What did you feel like trying to stop vaping, was it hard.

604

01:06:26.610 --> 01:06:29.850

[5101]: I was literally just a ball of anger, my nerves are so like oh, I just hated everybody. I would catch myself, thinking oh I need to vape, but I can't say that because then they know you vape. I was seeing the staff go out and come back and smell like cigarettes. Like I can't do that, I hate the smell of cigarettes, but I can vape, but they’d come back in and oh it made it so much worse.

608

01:06:53.820 --> 01:06:57.810

NB: did your doctor talk to you and all about like tobacco or vaping or any of that stuff.

609

01:06:58.200 --> 01:07:00.630

[5101]: yeah they gave me all these flyers. nothing but flyers and flyers, and I was like God, I know i'm not dumb. they gave me so many like but they didn't even know It’s just a part of the place they give you all types of stuff. So much.

612

01:07:19.530 --> 01:07:23.730

NB: So i'm going to go out on a limb here and say that the flyers were not very helpful.

613

01:07:25.620 --> 01:07:38.940

[5101]: Well, no, and really when I got caught ordering the vape they made me get a big giant essay on why not to vape while pregnant and I was so irritated I had to do it, and I was so mad, like I said I should have never got caught.

They made me do this big essay like oh my Go,d five paragraphs, I don’t mean short five sentences, big paragraph like I was writing almost the whole page. And I had I was writing big but I still had to redo it.

Like they don't play at Bethesda, like that that's one thing I really like about them, they stay on their game when it comes to your baby, but they're not - I don't listen well.

617

01:08:06.900 --> 01:08:13.860

NB: What made you want to stop vaping while you were pregnant. were there things that you were like maybe I shouldn't or?

618

01:08:14.760 --> 01:08:26.700

[5101]: same thing as weed. like what I am doing my kid gets like it, that was a big thing for me like what everything that I do affects my kid, so if i want my kid to come out with asthma, I can choose to keep smoking.

But if I want a healthy baby and I don't want all that and I don't want all of these hospital trips and complication, you have to stop. Like that's what was my biggest thing like unless you want a kid who can barely breathe, then you should stop. And knowingme i'm a hypochondriac, so the minute my son coughs I would be at the hospital so like it's better to just no. just stop. It's dumb, you’re going to be goign to the hospital every other day, if you decide to smoke. And it’s gonna be on you. And you have yourself to blame and I didn’t want to blame myself.

623

01:09:02.160 --> 01:09:08.340

NB: What about things that like triggered you to want to smoke or vape, while you were pregnant what were those things.

624

01:09:09.660 --> 01:09:18.000

[5101]: My body, like one minute i'm just having a good old time, next minute my throat is on fire and oh my God I can't take this pregnancy, no more.

I will try to sleep throughout the night and wake up with just acid reflex already had it really bad I always acid reflux, it is just my body, but plus heartburn and acid reflux, it was horrible my body was on fire, I was like I just need to calm down but i hated going through it and I would be irritated frustrated and that's the time I would want to smoke. Like I can't smoke in Bethesda. They have a sign, and it was just like God damn. I feel like if there were times I wasn’t in Bethesda and I felt like that, nothing would have stopped me from smoking and then I will regret it after i'm done. So I do thank Bethesda for that, because there nights I just need to get up and go vape but I didn’t have a vape.

630

01:10:00.540 --> 01:10:05.040

NB: That make that sounds I mean it just sounds hard to be so uncomfortable and not sleep and. like I know, nicotine and vapes are like very addictive too so it's like of course you'd want to do that at that time.

632

01:10:14.760 --> 01:10:15.210

[5101]: yeah.

633

01:10:17.550 --> 01:10:31.710

NB: um, how do you feel like we could have as a you know healthcare place as a culture like, how can we better support women to like cut down on vaping or stop vaping while pregnant.

634

01:10:33.360 --> 01:10:42.420

[5101]: And the fact that a lot of kids come out with asthma complications, like, I think that would be a big one, like your kid will have problems with breathing. Like that was like really I was just thinking about the fact like. I was honestly just thinking about smoke blowing into my son face that's a really made me made me not want to smoke, I will just look at it and be like. It helped me not to smoke. I know sounds crazy but it's the way I really, when I wanted smoke, I would just think it’s all in your son's face blowing in his face and that's what made me really stop. When I thought about like that I didn’t want to smoke anymore.

639

01:11:15.150 --> 01:11:21.900

NB: So what was it it wasn't even really about you not wanting to smoke, it was more about you not wanting to take away his

641

01:11:23.550 --> 01:11:37.260

[5101]: It was never about me, it was always about him, me not wanting to do that to him. I never I don't want to give my fine but that's how I stopped myself like if you smoke this right now, you inhale your son will be choking.

And then that's it, sounds crazy, but that’s what stopped me, I’d think like that and I wouldn’t want to smoke.

643

01:11:45.990 --> 01:11:52.320

NB: I mean that's helpful to know like if that was like the image that you needed to not do it like that's such a interesting thing.

644

01:11:53.040 --> 01:11:54.630

[5101]: Exactly. You stop. Just like that.

646

01:11:59.190 --> 01:12:06.450

NB: Okay, so here's the last question, which is for this section and then we're very close to being done and I don't want you to think it's gonna be like a 15 hour interview, you know.

647

01:12:06.480 --> 01:12:07.320

[5101]: It’s ok, I’m cooking and feeding [baby] all about the same time.

649

01:12:11.790 --> 01:12:24.720

NB: um so Sometimes this is the same questions before people say that you know, having an LGBT QIA identity or interactions like changes, how they use tobacco or influences that.

For example, like you know, sometimes LGBT Q, a people face discrimination and that can change or increase their tobacco use do you feel like that is true for you at all.

651

01:12:38.880 --> 01:12:43.980

[5101]: Not for me personally, no I know it happens to people, but not for me personally.

653

01:12:54.210 --> 01:13:00.450

NB: So what about using them together marijuana and tobacco, at the same time, like did you ever do that.

654

01:13:01.920 --> 01:13:05.580

[5101]: It just makes you tired because, because nicotine is a calmer, weed is a…

Just it just made me so tired like I was so like relaxed I could just fall asleep. I know with alcohol you mix it with a cigarette. I could drink all day and be happy jump around the House, and the minute I smoke a cigarette I just want to pass out, go to sleep upstairs. nicotine just takes away the fun of everything.

657

01:13:25.050 --> 01:13:30.780

NB: Okay, so, would you do them together on purpose or would you like, rather, not because you want.

658

01:13:30.840 --> 01:13:38.400

[5101]: um it depends, if I want to party on weed and I want to have fun I’m just gonna smoke, but if I wanna you know, calm down and take a nap i'm gonna have a cigarette, or a vape, whatever you want to call it nicotine.

660

01:13:50.010 --> 01:13:54.360

NB: That makes sense ones like the combos like chill

661

01:13:55.050 --> 01:14:02.880

[5101]: and it seems like nicotine does that to everything, drinking it did that, to my drinking it literally does it to everything.

662

01:14:05.850 --> 01:14:12.960

NB: Were there are times in that Gray period we were talking about where you use them together, because you didn't realize even then that you were pregnant.

663

01:14:13.680 --> 01:14:14.100

yeah.

664

01:14:16.230 --> 01:14:21.630

NB: Did it did being pregnant like change how they felt for you, even though you didn't know you were pregnant this me.

665

01:14:22.860 --> 01:14:26.190

[5101]: um let me see I can think about when I was pregnant. That’s around the time of my heart started beaing actually. Yes. It did change it caused me to freak. And yeah.

670

01:14:41.790 --> 01:14:42.240

NB: wow. So your body started to change how it was reacting.

672

01:14:47.490 --> 01:14:47.790

yeah.

673

01:14:51.240 --> 01:14:55.260

NB: Okay, so we're going into the last series of questions, these are kind of like I really like them, but I think that they can be I don't know the right word they're kind of like different so it can be, if you need me to clarify about them just ask.

675

01:15:08.100 --> 01:15:08.490

[5101]: Okay.

676

01:15:13.020 --> 01:15:19.110

NB: Okay, so these are called the ideal world what they are, as I want you imagine your perfect world okay. And in that world, what do you wish that all LGBT Q I people knew about pregnancy.

680

01:15:40.560 --> 01:15:41.730

[5101]: can you say it one more time

681

01:15:42.090 --> 01:15:55.680

NB: yep so you're in a perfect world, but really The important thing is just remember, is the question, what do you wish that all LGBT Q people knew about being pregnant.

682

01:16:01.710 --> 01:16:07.650

[5101]: That it's like it changes your body. And like this is weird, but like, when you get into a relationship right and like how people do it like, I transgender if the dude is like the girl, and like understand? But in a relationship I don't think they realize, even though in like that world he is the guy, technically he has the baby and the baby goes with him.

So i'd like never thought people understood that because, like my cousin that’s her situation now like she, well she had her, well it's hard to explain now, her girlfriend, transgender boyfriend, had a baby right and she's supposed to be the girl. But really you know she thinks that she's entitled to have the baby, but technically he gets the baby because it's his baby if that makes sense, he pushed the baby out.

687

01:17:05.310 --> 01:17:05.580

NB: yeah.

689

01:17:08.250 --> 01:17:18.090

[5101]: it's his baby. And it's not like it's they really like they did it together, he did the little method where you go to the place and they find you a sperm donor and do all that. I don't think people understand that, like it's a family, but in in life that's how it goes. Especially if you do it, like the sperm donor and it's not technically your child, it goes with that person no matter how attached you get.

692

01:17:37.110 --> 01:17:37.590

NB: Yes.

693

01:17:37.650 --> 01:17:43.320

[5101]: And I hate that for some people, because I watch youtubers who go through that none of us are bad because they should have thought about that in the beginning.

694

01:17:44.250 --> 01:17:48.270

NB: Yes, so yeah you're saying that like regardless of like the gender of the person who had the baby, they're the biological parents, so they have biological parent right, whereas like the partner, no matter what their gender is doesn't have those same parenting right.

697

01:18:09.330 --> 01:18:14.250

[5101]: Yes, I mean it doesn't apply if they had the baby together. Right, but if, like I said you go the way of the sperm donor, then that maybe goes with his biological parents. Right and I be feeling so sad because we're so attached and they’re like that’s their son, and it feels so bad, but by all rights that's their baby and they get to take the baby with them.

700

01:18:32.220 --> 01:18:47.160

NB: yeah that's interesting to think about like as this becomes maybe more common, as we see people like gender shifting around like or they're going to have to be rules and contracts and stuff like that just for these kinds of situations

701

01:18:47.190 --> 01:18:51.570

[5101]: Yes, I feel like that's exactly that's exactly what needs to be done, because I feel so bad for them people who gett their feelings hurt like that.

703

01:18:54.060 --> 01:18:55.920

NB: yeah because they basically raise a kid.

704

01:18:56.700 --> 01:19:03.690

[5101]: yeah and they look at it as their kids, then they separate, then they don’t have a son because technically you didn’t.

707

01:19:07.260 --> 01:19:15.060

NB: Okay So what do you wish all healthcare providers knew about LGBT Q if people that were pregnant.

709

01:19:19.260 --> 01:19:26.070

[5101]: I think I just knew that some are probably uncomfortable, like they're not as open to pregnancy like most people. like they're not I don't know how to explain it like most people are just open like you know well yeah I wanna know the gender and.. some are excited like they want to know when a baby's coming they want it to hurry up, and some are just scared like you know I feel like it's a different experience for people. That are like that. Because some people don't know if they want their child to grow up like them, like I may bring him into a house where I like girls, but I don't want my son to think it's okay to be gay if that makes sense. I think, some people are scared because they don't want to affect the child, the way they were affected like if I don’t know how to explain it. Like a gay couple doesn't want their son to be gay.

714

01:20:06.600 --> 01:20:13.650

NB: I think you're explaining it

715

01:20:14.160 --> 01:20:26.790

[5101]: and I feel like health care providers need to know like the way you word things is probably very important because they're probably scared, like my life might have a big impact on his baby's life I could be rooting for him to be exactly what I don't want him to be just by being myself.

716

01:20:28.590 --> 01:20:30.540

NB: yeah I understand what you're saying. I definitely think that your son is lucky to have you as a parent, regardless of whether, whatever your identity is you seem like you're obviously super intelligent you're very kind you're also funny like whatever that stuff is, he's lucky to have you.

718

01:20:53.670 --> 01:20:54.240

[5101]: Thank you.

719

01:20:54.900 --> 01:21:01.500

NB: you're welcome. what do you wish all LGBTQIA people knew about tobacco or vaping use.

720

01:21:03.330 --> 01:21:03.840

[5101]: um.I don’t really know that one. It’s bad for you, no matter what you are, how you identify. That's what I think everybody should know. Yes, it probably calms you but it comes with side effects like lung cancer all that.

Yes, it calms you, but no matter who you are what you are what you do that, it definitely finds you. like it hurts your body, it’s not good for you yeah.

725

01:21:36.360 --> 01:21:44.340

NB: yeah that makes sense because it's doesn't the tobacco doesn't know whether or not you identify a certain way.

726

01:21:44.700 --> 01:21:49.500

[5101]: yeah it doesn't matter does it that's like saying you’re shooting a gun with someone’s name on it, but it doesn't do that. It goes to wherever you put the gun like it doesn't matter. who, you are what you are yeah.

729

01:21:56.940 --> 01:22:05.850

NB: So the last question is the same question but about tobacco or i'm sorry marijuana use So what do you wish LGBT Q, a people knew about marijuana use.

730

01:22:06.960 --> 01:22:08.310

[5101]: The same things like. The same thing. It’s bad for you, It doesn’t help you, even if you think it helping you get through your problems or issues if somebody doesn't accept you if somebody doesn't.

It doesn't it doesn't help it’s only you're only there for that moment for that minute of high. Once the high’s gone, you’re back to reality. I wish that they knew that like yeah it's probably helping you get through your recovery probably helping you get out there, it’s probably helping a you read lot of comments and anger that people are giving you Once the high’s gone, you’re back to reality.

Its not like you’re going forever.

735

01:22:45.630 --> 01:22:46.950

[5101]: Just for the moment.

736

01:22:48.630 --> 01:22:49.530

NB: Right like it's like the high is brief and it doesn't actually change what you're going to come back to once you're not high.

738

01:22:58.710 --> 01:23:12.060

[5101]: yeah another thing. Now a high affects the way people act, so one day they might feel like I’m high I can tell the world that i'm gay, I just want everybody to know, and so you kind of regret the fact that you ever said that. That you ever told anybody, because now, here comes the backlash in the people who don't believe in that stuff.

Also that's the thing it affects how you act so on that high people need to really be careful around the people they choose to hang around because some people take advantage of you also when you are in that type of thing. Right, no matter what you are a dude will talk to, will touch a girl who wants to identify as a man, dudes have done that plenty of time, but also, they just because they're your friend or whatever they need to be careful. Drunk, high, anything whatever they need to be careful because you identify as a dude, but dudes don’t look at that. They look at the fact that inside you're still the a woman.

744

01:23:56.220 --> 01:24:01.230

NB: It sounds like can make you vulnerable to be high or drunk or whatever.

745

01:24:02.160 --> 01:24:07.770

[5101]: Definitely even high it makes you vulnerable because you're just happier. You're you're an idiot you just want to be goofy. And everything is a joke and somebody literally being serious and you're just laughing like everything’s a game until it's not.

747

01:24:19.020 --> 01:24:21.660

[5101]: [baby] if you wipe that on my shirt one more time.

748

01:24:23.670 --> 01:24:24.780

NB: It’s his napkin.

749

01:24:25.920 --> 01:24:27.570

[5101]: yeah that’s why there’s Cheetos all over him.

750

01:24:29.400 --> 01:24:39.030

NB: Those are all my questions, but I want to open it up, if you have questions for me or comments like you can tell me something I could do better like literally anything.

751

01:24:41.700 --> 01:24:42.270

[5101]: No. not really. Basically it was a really calm conversation I really liked it I didn't feel uptight or scared to answer anything. Actually really liked the conversation, you’re very good at what you do, how you word things actually. You don't make me uncomfortable at all, even with some questions I find somebody else asked me i'll probably jump back like what did you say, but you're very like the way you come off is very good.

756

01:25:06.690 --> 01:25:23.250

NB: Thank you, I also think it helps that like I actually remember you from recruiting and I felt like we just get along good like we have a certain energy that matches well, so I think that that always helps me like your [? ] are so interesting i'm like I could talk to you, for you know way more than an hour or whatever, but.

759

01:25:33.480 --> 01:25:40.350

[5101]: Yes, it really is good to talk to you. I could tell it was you've got a way to get off immediate conversation laughing. That's what I I like I like stuff like that I hate uptight and it feels uncomfortable because you just met this person and you gotta you know you don't want ask about my sexuality like. No. It actually will really good I liked it.

764

01:25:59.010 --> 01:26:04.140

NB: right because i'm talking we're talking about something that there's like a lot of stigma and taboos sex and drugs and like.

766

01:26:06.630 --> 01:26:15.720

[5101]: it's definitely very awkward with the person you just don't feel comfortable when she just I feel like Nancy I made would have never been given if I wasn’t comfortable .

767

01:26:16.500 --> 01:26:28.230

NB: Well, I really like I value your honesty and your experiences a lot, so I appreciate your willingness to kind of go into some uncomfortable zones.

768

01:26:29.580 --> 01:26:30.090

[5101]: Thank you. Thanks for making it comfortable.

770

01:26:33.750 --> 01:26:37.860

NB: I try I try it helps when it's like we can have kind of fun, I think.

771

01:26:37.980 --> 01:26:38.820

[5101]: yeah true.

772

01:26:39.780 --> 01:26:41.430

NB: i'm going to send you that book.

773

01:26:42.270 --> 01:26:42.660

[5101]: Oh yeah. I was about to ask you that.

774

01:26:44.400 --> 01:26:50.070

NB: yeah i'm gonna send you the book and it's actually three books it's a trilogy it's so good um. And then i'm going to well i'll first off with $50 on your card, let me see what else. First I’ll put that $50 on your card.

777

01:26:59.700 --> 01:27:02.280

NB: yep you can use the same card i'll add that money right now.

778

01:27:02.940 --> 01:27:05.370

[5101]: Okay hold on before you do that, let me go look for it.

779

01:27:05.730 --> 01:27:09.090

NB: Yes, yes, if you don't have it, I can send you another one that's no big deal.

781

01:27:14.610 --> 01:27:16.350

[5101]: Look there's only one place, it could be. .. I'm so discombobulated.

785

01:27:48.840 --> 01:27:53.880

NB: i'm not a very organized person, especially with like wallets and cards like it's really.

786

01:27:54.720 --> 01:27:56.220

[5101]: I’m not a wallet or card person at all

787

01:27:57.600 --> 01:27:57.900

[5101]: well. There goes that. Dropped the whole water. Have like six cards in here .

792

01:28:12.330 --> 01:28:13.740

NB: Hopefully, they all have money onthem.

793

01:28:16.020 --> 01:28:17.730

[5101]: No i'm pretty sure they’re bankrupt. I wish.
