# Supplementary material for: Experiences of postpartum mental health sequelae among black and biracial women during the COVID-19 pandemic
Source: BMC Pregnancy Childbirth. 2023 Sep 4;23:636. doi: 10.1186/s12884-023-05929-3 (PMC10478375; doi:10.1186/s12884-023-05929-3)
Supplement: Supplementary file 21 — Supplementary Material 21 [file 12884_2023_5929_MOESM21_ESM.docx]

**Supplemental File 1.8 Interview Transcript with Participant 5207**

Q1 5207

Interview Transcript

I: Okay, Um, okay so. I hope that you have like some a little bit of a private or quiet space, just so that you know you're not- you feel like you can-

0:08

P: Yeah.

0:15

I: answer the questions

0:16

P: Yeah, I have headphones in.

0:17

I: Oh, cool. Yeah. I'm also in my house by myself. So no, there's no risk of anybody hearing us. Okay, um, let me see. Okay, so. How, how, how have you been? How’s the pregnancy going?

0:30

P: I'm good. Honestly, um, but I was I'm kind of a little bit worried for I had my first ultrasound yesterday and they, uh, they told me that my placenta is anterior. Which means like the baby the placenta is in front of the baby, but my mom was, when she was pregnant with me she had placenta previa which means like, um, it blocks the cervix, I guess. I don't know. And my mom said- I told my mom about how my appointment went yesterday and she said like I should be like really careful and like be on bed rest because if that is placentia, uh, placenta previa then, um, it's really dangerous. So I was just a little bit worried about that.

1:04

I: Yeah, that's- that sounds scary.

1:19

P: Yeah. But, um, other than that, it’s going pretty smooth, honestly. Yeah.

1:21

I: Yeah, so that was something you heard that yesterday at your appointment you found that out?

1:32

P: Yes

I: So what does that- What does that like mean for you like maybe bedrest or like anything else?

P: I honestly, um, the tech didn’t tell me, like, okay, we have to do bed rest. I was thinking that maybe I should just in case. I actually contacted them to make sure. Like, what should I do? Um, but she- she didn't tell me like it was anything bad. I just hope it's not what my mom had because she said it can be dangerous and I don't want anything dangerous. You know this is first child and I just want everything to go right

2:06

I: Yeah.

2:08

P: So, yeah.

2:10

I: Have you, um, talked to like your OB about it? Like will you?

2:13

P: Yeah. Actually, um, on the app you can text them like message them about like any questions you have. I actually contact them right after my mom told me to so they should be, you know, getting back to me pretty soon.

2:16

I: Have you had, um, do you like your OB GYN so far, like how's that relationship in for you?

2:31

P: Um, yeah, she yeah she helps me out. Yeah, I'm pretty- I'm satisfied with her.

2:38

I: Yeah, does she, um, has she asked you, like a questions about marijuana or tobacco use it all when you, when you went to your visits?

2:45

P: Um, yes, they did ask me did I, um, she has asked me did I ever smoke, like do I smoke right now, alcohol use and all that. I do not do any of that. Yeah, but she has asked me before.

2:59

I: Yeah, have you ever tried any of that or you just like no, never.

3:04

P: I have tried, um, alcohol because, um, I, well, New Years, you know, celebrations and stuff. But other than that, I really don't drink or anything. I'm not really the type.

3:12

I: Have you ever like smoked a cigarette or any like tobacco or vaped or anything?

3:22

P: I hate the smell of cigarettes.

3:27

I: Is that why? Why don't you? Can you tell me more about like why you have never tried that?

3:29

P: OK, so my mom and my father smokes cigarettes and my grandmother she, um my mom's mom, she actually passed away from lung cancer and she's just smoke cigarettes, too, and It's just really scary for me and I don't like the s- I hate the smell and it gives me like a major headache. So, I just stay away.

3:48

I: Yeah, that sounds like yeah that would- I mean, I'm sorry about your family member. That's sad.

P: Yeah

I: It's sad that, you know, that's how we learned that it's bad for-

P: Yeah, right.

4:06

I: Yeah. Um, what do you- okay so that's how you feel generally, like I'm wondering also about how do you feel about women smoking tobacco when they're pregnant?

4:08

P: Okay, I think that's a big no because, um, it can affect your pregnancy. It can affect your baby and I don't, I don't think that's a good idea at all. I feel like that's a big no. Once you find out like once you're confirmed pregnant, your pregnancy is confirmed, I feel like the tobacco and alcohol should be a big no. Like you should just push them to the side until you know you're done with the pregnancy at least or until your doctor, you know, gives you the yes that you can smoke again, or you can drink again.

4:39

I: Okay, that, I'm wondering, it could you tell me how you feel about marijuana use during pregnancy specifically

4:55

P: Um, okay, so I do know that a lot of woman do smoke marijuana like in the beginning of their pregnancy because the nausea and they can't really keep, um, food down. So, um, I feel like at the beginning I mean, if you, if that's the only way you can keep something down, you know, if that's the only way you could provide food for your, for your baby, then you know once or twice shouldn't be like that bad. But I'll still say, no, I would not do it. Uh-uh, I got medicine. I have had problems with keeping food down too be- well I'm still in the beginning, but like in the beginning beginning and, um, I went and got medicine because I-I, personally, don't think that smoking is, you know, good for the baby at all.

5:42

I: So what, um, did that medicine end up working for you for your nausea and stuff?

5:51

P: Um, yeah, it works. It just makes me feel like really droopy, like really woozy all day. I take it, like, three times a day and, um, I take one in a more I take- Um it's two pills that have to take together and I take them three times a day, and I do the morning, the afternoon, and then I do at night and, um, they pretty much work for me. I just don't like how it makes me feel like really, really woozy all the time.

6:17

I: Yeah, that would, I would not want to feel woozy all the time.

6:23

P: Yeah. But, um, I just know that it's all going to be worth it at the end, so I'm willing to do whatever.

6:33

I: Yeah, that's a main thing.

P: Yeah.

I: What about marijuana use generally like not when you're pregnant. How do you, what do you feel about that?

6:37

P: I honestly feel like if you want to do it, then, you know, you do it. It's your choice, but, um, me I actually, I tried marijuana before and I don't like it. I don't like how it makes me feel. I don’t like how it gives me like, oh, I just, I just don't like it. I just don't support that. Like I just, I don't like it. So after like if- if you want to do that I mean, that's your choice. I mean marijuana is not really that bad compared to like any other addictive drugs, but I do think that I will never do it again. Ever.

7:20

I: Can you tell me what that was like, like why- what happened that you didn't like?

7:21

P: Okay, so it makes it, it made, well, I don't know. Uh okay, so after I feel like it makes me feel like, um, like not myself like as I think a lot, I panic and it’s just like I don't like to panic. You know a lot of people will, they usually do drugs, not saying that I did it, just to do it. I just did it to try. I was really young, you know, but, um, a lot of people do drugs to hide pain and to, uh you know, hide, hide away their thoughts. They don't want to think about the bad things that’s going on in their life and stuff and I just did it just to try it. Like, because my friend, I had a friend that, you know, did it and she's like, convincing me so I'm like, “okay, whatever”. And I did it and it's like, I will never do that again ever. I didn't like how it made me feel and I felt like- I felt like really weird for like the past couple days afterwards, like two, two days afterwards I was still feeling like weird like it was in my system. So, like, yeah, I won't ever do it again.

8:23

I: Yeah, yeah, that makes- I- thanks for telling me that and I mean, that makes sense if that's how-

8:27

P: Yeah, I mean everyone's different, but my personal experience, I really did not like it.

8:29

I: What about your like do you have friends that that use it and have other experiences with it and stuff?

8:35

P: Um, I don't really go around people that smoke honestly, because I just, I just don't like it. Like, I don't like the smell. It gives me headaches. I just, I just, I'm not that- I just hate it. Like I just, I don't really go around people, and I barely have friends, honestly, due to like COVID and, you know, this pandemic. I want to stay safe, so I usually just stay in house honestly.

8:51

I: Okay, yeah, it's an important time to limit who we’re around, I guess, too.

9:10

P: Mhm. Yeah

9:12

I: Yeah. Um, how old were you when you tried it that one time? Just wondering.

9:15

P: I was like I was really young. I was like like 16, I guess, well, not really young. But yeah, I was still a teenager. Well, I'm still a teenager now, but I was like 16. But I wouldn't do it again. I will not do it again.

9:30

I: You're like an adult teenager now.

9:35

P: Yeah, exactly. Yeah, I’m way more mature. Like, I know what’s I know like that was wrong and I won’t do that again.

9:37

I: Um, um, I'm curious. Like, where do you or did you at all learn anything about like- hold on one second- about like marijuana and pregnancy, like, have you learned anything about that? Are you hearing anything about that?

10:00

P: I just know it’s wrong. I'm the my- my-. I just I just know that it's wrong. I, I haven't liked read into it or anything. I don't really go around it like, you know, I don't really care too much for. So, I don't- I don't like search around for information about it because I don't do it and I know that it's wrong. So I don't really- I know that like the doctors tell you like no, no smoking, no drinking, but I already knew that. So it's like I don't, you know, I don't do that.

10:30

I: Yeah. What about both of them like, what do you think about using marijuana and tobacco at the same time?

10:43

P: That is really, really bad. Ah well, I feel like that's really, really bad. I never did it before, but I feel like that's really, really bad because it's just that's a lot on your lungs honestly, and you need your lungs to breathe. And like I said, my grandmother passed away from that type of stuff. So, so it’s like no. This is really bad. I feel like people who do that should get it together and, um you know, try to lay off of it on a little like a little bit, you know, because that's not healthy at all. Yeah.

11:06

I: Yeah. So you're, you're like experience with your grandma kind of informs your belief about this. Is there anything else that has like happened in your life or that you, that you learned or how you were raised, that's kind of informed why you think this is bad?

11:36

P: Oh yeah, I actually was raised like I don't, don't, don't, um, smoke, don't do none of that because it's not it's not good. Like, and plus, if I was like to get addicted to it, then it would be we really hard for me to, you know, get out of that stage. So I was definitely taught to, you know, don't do that. But, um, it's pretty- yeah it’s more of my, my belief, too, because I just think that it’s wrong in general because you know people lose their lives from just, you know, smoking this. So, I just feel like it’s wrong in general. So my mom- but my mom, definitely, definitely put into my head like, you know, this is not healthy, you should not do this. Um, especially because when I was younger, like I-I wanted to be a singer and she's like, you can't, you know, do that if you smoke or, you know, so just stay away from this. Stay far away from this because you need healthy lungs. So, um, I definitely just know that it's wrong. I was taught it, too.

12:36

I: You said something about like how some, you know, people might use these kinds of things when they're, when they're in pain.

12:40

P: Uh huh.

12:48

I: And I'm wondering, like, what, what you maybe do when you're in pain since you don't do these things.

12:49

P: Um, well, when I'm in what like mentally? Or well, yeah.

12:57

I: Yeah mentally for sure.

13:01

P: Well, mentally, when I'm in pain. I-I like being by myself, my misery does not like company. I don't like being around people when I'm feeling some type of way. I usually just stay by myself and, um, I try to just think positive about like how I'm feeling. Like if I still sad- if I feel sad about something I want to be by myself have a full head to myself and just think like positive things about how things can get better. But honestly, I don't think I'll ever run to a drug, if I was- If I was in pain again like because I don't really get depressed or anything. But if I ever was to I wouldn’t run to drugs because I just know it's wrong and I wouldn't want to harm my baby and wouldn’t want to harm myself so I wouldn’t. It would just be no.

13:55

I: Yeah, yeah. It sounds like your firm in that like you know yourself in that way.

P: Yeah.

I: Um, I'm wondering, like, if you, you needed to talk to your doctor about drug use do you feel like you would be able to?

14:07

P: Um, absolutely. Um, yeah, yeah because I would, I would know if it was effective. I will want to know if it was affecting my child. Um, I wouldn't, I mean like I keep saying, I wouldn't personally do it like I would not do that. But I definitely will absolutely, absolutely want to know if it was affecting my child. So I would tell my doctor.

14:23

I: How do you think doctors can like get information about this kind of stuff to like young pregnant women better or are they doing that? What do you think about that?

14:39

P: Wait. Um, can you repeat that please?

14:51

I: Yeah, sure. I think that was my fault. I think I asked-

14:52

P: No, it's okay.

14:55

I: It was like five questions in one. Um, so how do you think, uh, is there a way that you think doctors could maybe get information about marijuana use to patients better than they are. Do you think they're, you know, is there something that basically doctors could be doing better.

15:13

P: Um, honestly, it's not really like the doctors like fault that like woman like people don't like coming out about it because like when people come out about drugs and stuff like they, they probably do feel like they're being judged or, you know, looked at like a weird way. Um, so that's probably like another reason why a lot of people don't come out about, you know, doing drugs and things because they don't want to be judged. But I feel like doctors it’s not really I mean, yeah, they should want to know, and you know, so they can know like what to expect. But I feel like it's more of the person who's doing the drugs problem. It's more of their responsibility to come out to the doctors because they can help them, you know. I feel like they shouldn’t just do it, they should not do it if they see like the side effects and how, what it does to people. I'm not saying that marijuana is a horrible drug. I mean, if, if anyone does it you know desk that's your, you know, that's your decision. But it's more of the person who’s do- who's on the drug it’s more of their responsibility to come out about it.

16:33

I: Okay. Do you think that, um, have you, have you heard from like friends or, you know, people in your family, um, messages about what you should and should not tell your doctor.

16:38

P: Um, no, I'm, I'm all, I always- I was always been like an honest person. Like I always just, I'm really open about a lot of things. Um, I tell my mom like literally everything, especially since I got pregnant because she has more experienced than me and, um she told- like I wouldn't be, I wouldn't have a problem with, you know, even if someone were to tell me, like, “oh, don't do this”, whatever if I knew that was the right thing to do then I will do it. Because you know I'm all, I was always open person I don’t really hide things. So, yeah.

17:24

I: So, um, it seems like your mom has had a lot of like effect on some of the things that we've been talking about. Is there like anyone else or anything that you'd like any other place that you learn this kind of information from?

17:45

P: Um, no, not really. I mean just experience of- of losing a family member from it just made me like, it just really opened up my eyes like it was it was bad to do and it's life threatening. But my mom is like a really big inspiration to not do drugs or not do smoke or anything, because what happened to her mother, you know. So she's, she's really big on that. Yeah, only for- honestly, my mom only.

17:59

I: How do you think that we could better- like is there a way that doctors might better help some of these young women quit doing marijuana or tobacco?

18:32

P: Um, it’s really up to the person that's doing the drugs. Honestly, because they're probably addicted to it or they're probably, you know, they probably feel better whenever they do it. So I honestly, I really don't know what the doctors can do. They could try to, you know, get them to talk about it more often or, you know, get them to open up about it. And, you know, tell them about their problems because maybe they feel like they can't talk to someone and that's why they smoke or maybe. Yeah. Maybe they feel alone and they feel like they can't talk to someone, but it's honestly really up to the person on if they want to stop or if they want to you know, open up about that topic.

19:23

I: Mhm. Yeah. Let me think about that, where we can where we can go next.

19:26

P: Okay.

19:30

I: Are you doing okay? Have all the questions been okay? How do you feel?

19:33

P: Um, yeah, I'm doing okay. I just am I, yeah, I'm doing okay.

I: Are you sure?

P: Yeah, I am a really big thinker, too. So like I think a lot.

I: Yeah

P: But, that's, that's another- I just think a lot. That's it. I just have that natural brain to just think a lot

19:50

I: Always going.

P: Yeah.

I: Mhm. Oh, this is a good one. So, what makes it or is it different at all to talk to someone like me like a researcher versus like a doctor, is there a difference, or what do you think?

20:10

P: Honestly, no. I told the doctors exactly what I can tell, I can tell a doctor whatever I tell you, because I don't have anything to hide so. And I'm not ashamed of anything because I don't do it. Um, so really, no. There's no difference. Because I don't, I don't, I'm not ashamed of anything I do, you know. I don't do anything so

20:47

I: Yeah. What would you maybe tell if you had a friend that did this? Would you feel comfortable talking to her about like smoking while she's pregnant or?

20:49

P: Oh yeah, I will be like really not harder, but I would definitely tell her, like, you know, that's not safe and that's not good for a baby, um, or you. Because, you know, that has a lot of side effects and, you know, you can get your baby taken from you if that's in the baby's system, you know, you don't want that. You know, if you're- who will want to carry a baby for nine months, you know, deal with all the nausea, fatigue and all of the breakouts and the pain and, you know, just for their child to get taken away from them because they, they couldn't change something on their end. So I wouldn't want it. I would definitely try to encourage them to do better because you wouldn't want your child just to be taken away from you because you could have changed it. You could have fixed it.

21:35

I: Do you know people that that's happened to?

21:46

P: Um, no, not really.

21:49

I: How do you know that that's the thing that happens? I'm wondering.

21:53

P: Um, because I do know I have, I watch a lot of movies and I do know that’s a thing, you know, if a baby's born with something in their system. And I read too. I read a lot. I do a lot of research. So, I do know like if a baby's born with something in the system- because I know like whatever a mom takes in, the baby takes in. So, um, If the baby's born with that in their system and they see it in the baby’s system, you know, they're going to be like “Okay, well you had every chance. You had so long to stop this” you know. “Why didn't you do this?”, you know, “You're not putting your child first” and, um I just know that they will. There can be like CYF involved and, you know, because, you know, you don't want your baby living with those side effects. And now, now, since you couldn't, since you couldn't stop what you were doing while you're pregnant what makes you think you're going to stop now. You're probably gonna smoke in front of baby, you know.

22:43

I: Do you think, uh, uh, pregnant woman using marijuana throughout her whole pregnancy is like the same as someone using like heroin or something like that?

22:51

P: Um, I don't, I don't think so. I mean, because I know heroin is I probably a deadly like you can overdose like you die from, you know, taking too much of that in. But, um, marijuana? I don't think you could die from it, but their definitely two completely different drugs, but they're both, you know, their definitely different from each other. And heroine is I feel like is way stronger because I see a lot more people addicted to, you know, them type of drugs and in in rehab trying to, you know, get off them. I feel like it’s harder to get off of that, harder to get off of heroin drug than marijuana. But, I honestly wouldn't now.

23:37

I: Do you think it affects the baby differently, like marijuana versus heroine-

23:48

P: Oh, yeah.

23:51

I: or something like that?

23:52

P: Yeah, so I feel like it might, because if it can affect a- a human being, like, if it can kill a human being or if it, if it can really affect you like that bad, just one person. What makes you think- what can it do to a baby that hasn't even developed everything yet.

24:10

I: Do you think that it's okay that a baby is, you know, that maybe a baby tests positive for marijuana and then, you know, it gets removed. Like, what are your thoughts about that?

24:16

P: Um, it's honestly really the mom's fault because, you know, you had every opportunity, every chance you had a long time to stop this and if you really thought about your, your child, you wouldn't ever did it. Because like I said, there's a lot of side effects and you don't want your child living with major problems because you didn't change something. So I honestly, maybe the baby and who knows the like when the baby's here if she gets frustrated or something, she goes to the bathroom and she smokes or whatever, or she smokes in front of the baby and the baby dies from, um, smoking. What is it? Smoking infant? SIDS! If the baby dies from SIDS. Um like, what happens then? Then she's going to be blaming herself and, you know, it's your fault. It's your fault because you should’ve, you should’ve knew. You should have knew to stop that, because your baby comes first. You know, your baby's health is supposed to mean everything to you. You're supposed to want your baby to be healthy. You're supposed to want them to be normal and you don't want them to die from, you know, or have side effects, um birth effects and stuff because you couldn't stop something that you could have. You had an opportunity. You had a chance

25:40

I: When you, um, you talked like about doing some research and I'm, I'm like really interested in where you get like your information about this kind of stuff. So is that, like you said, movies, where else do you feel like you look for like facts and stuff, you know?

25:46

P: Okay, I, I haven't really, like I said, I think a lot. So I do like just like a question will pop up in my brain and that's, like, okay, I'm a look this up. Because I want to know and like if, if something pops up in my brain or like it'll bother me until, until I know like the answer. You know, like, I'll have to have to keep you know. It's just it just I’ll look something up, and then I'll continue reading about it because my-m like I don't know I'm just really interested in stuff like this. Well, not in stuff like this, but I'm just really interested on what can go wrong and what can't go wrong. And even though I don't do drugs or anything I just, I'm just really interested, like I just, I don't know. I'm just a really curious person. I just, I just want to know.

26:31

I: So you like, would you like Google it. Like do you use just the internet?

26:47

P: Yeah, yeah I'll just get on Google and I'll just look it up like okay, well, you know, whatever it is, but honestly, I don't really ever look up anything about drugs because I don't do drugs. But if I was, I would definitely ask Google or or I can ask my doctor but honestly, I mean, I wouldn't it wouldn't be about drugs because I don't do drugs.

27:02

I: Right, right. How do you know that that info that you're getting, like, how do you determine that that's like trustworthy, you know?

27:12

P: Yeah, yeah and I have questions like that too. So, I don't, I don't really trust everything that I read. But if I if I see that answer multiple times then I'll be like, okay, well, okay, well more than one researcher put this on this website so maybe that is a little bit true. Because I see the answer more than once. So it's like okay, but if I wanted to verify something yeah I would ask somebody that knows the truth.

27:43

I: Because there's a lot of information out there.

27:49

P: That's not true. Yeah.

27:51

I: Yeah, it's so hard to know. Would you ask like, you know, a doctor or someone that you trust?

27:59

P: Yeah, I mean, yeah, if I had- if I had a really like question that, uh, yeah. Yeah, I don’t really, yeah I’m a really open person and I'm like I say, I'm really curious. I don't really mind, you know, going to somebody who knows the truth or know the facts about it. I don't really mind because if I want to know, I want to know, you know?

28:26

I: Yeah, this is like a little bit of a leap, but I just realized, um, I didn't ask like, do you think marijuana or tobacco use, like, one is worse than the other or they about the same like?

28:27

P: Mmmm, I feel like maybe, um, they're about equal. I don't know. I know tobacco use is really, really bad because nicotine is really bad. Um, and tobacco period is bad. Um, yeah, I feel like honestly, don't know. Um, yeah I don’t know.

29:00

I: Yeah, it's just your opinion like?

29:05

P: I'm gonna go with, um, you want me to take either/or or?

29:11

I: You don't have to, like, if you don't- your answer can totally be, I don't know and like-

29:13

P: Okay, yeah. Yeah cause I don’t know.

29:20

I: Yeah, that's fine. There's so much stuff I don't know. Yeah, and that's one of the things so

29:25

P: That’s a good question.

29:28

I: Yeah, it's interesting.

29:30

P: Uh huh. Yeah, it is.

29:32

I: I- tell me if I asked this already is there- oh, I think I did. Never mind. I answer my own question. How do you feel like, like the COVID 19 pandemic has like is like affecting your, your pregnancy and your experience right, right now?

29:43

P: Um, well COVID-19 is really, really dangerous for a pregnant woman so I feel like I can't really do as much, but I'm okay with that because I'm, I'm an inside person anyways. I stay inside anyways, um. But about like doctor’s appointments and stuff, um, okay with one person coming to a doctor's appointment with me because like why would I want, you know, everybody with me? But, um, labor I don't, when you're in labor and delivery I don't know when like I don't know when that happens, if only one one person can be there still, but if that's- if only one person can be there I'll be like a little bit-. That will- that will affect me a fair bit because I would like my mom to be here. Um, yeah, I would like more than probably one person to be there, but it'll be okay. I guess I just don't COVID is just really dangerous period. So, I try to stay away from it. It stops me from doing a lot

30:53

I: Yeah. Like, like, like what

30:55

P: Like, like from like from getting on a bus and I'm I live in an apartment building, like I gotta wear a mask around to get mail and stuff. Um, it makes me really cautious. Like, I carry sanitizer everywhere I go. After everything I touch I feel like I got it. Or every, every touch I feel like those germs, even though there is germs everywhere. After every touch I have to put sanitizer on because I be panicking and I think a lot. So, it's like, I don't want to give it to my child, you know, so I don't want to get it all. I'd be really scared that I'll get it if I go out and stuff.

31:21

I: Yeah, it sounds like it's like scary.

31:37

P: Mhm, yeah, it really is.

31:39

I: Yeah, do you- when you go to doctor’s appointments do you think about it?

31:43

P: Yeah, yeah I do because I know like in hospitals there you never know like, you know, you never know who has it or you never know because there's people that can have it that won’t have symptoms, so they won't get checked because they don't know if they have it or not. But it definitely is a scary thing. But I try to, you know, follow the rules and wear my mask. I don't know why people wouldn't want to wear a mask. But I wear my mask and I do the social distan, social distancing, social distancing. Sorry, so hard to say that. But I did social and I try to stay away from people, you know,

32:17

I: Yeah yeah

32:30

P: So that's it.

32:32

I: It's important to keep your baby safe. You and-

32:33

P: Yeah that's, that's my main concern is, you know, if my child’s okay. If I were to get it I mean, yeah, I'll still be like, “No. Oh my god.” but if it's, it will be worse if my child were to get it.

32:47

I: Yeah, yeah Thank you for sharing that with me. I have like enjoyed talking to you today-

P: Yeah!

I: So much.

32:50

P: So, yeah. Yeah, I mean, I'm really open about this type of stuff.

32:56

I: Yeah, and you have like very, it's been very, I can tell that you like, one that you kind of like really know yourself.

33:00

P: And yeah.

33:04

I: You do look this kind of stuff up and you know you are-

33:08

P: right yeah, yeah.

33:10

I: Do you have any like, am I not asking a question I should be asking? Am I asking something in a way that's like offensive, like, do you have suggestions for how I could do this better?

33:12

P: Hmm. Let me think. Hmm. Um, No, honestly, I felt like you were really like specific about what you were- what you wanted to know, um, maybe, maybe like before, the before we start the questions and everything, maybe should I give a warning like okay well this doesn't like- if it, if it does effect anything, you know state that or if it or if it doesn't, you know, just to ease that person's mind or if that person is really nervous or, you know, really not really like an open person. Maybe you can help that person like ease up a little bit like more than like, okay, well, this, this, this does nothing you know, like it doesn't affect anything, I just, you know, I'm just a researcher and I'm just really curious and I want to know, um.

I: That’s a great suggestion.

P: Yeah, because there are some people out here. It is like, oh my God. Well, she's not about to ask me questions like, “what if what if this affects my baby? Like, what if they're trying to get my baby?” You know, so there's something and then there's people like “oh my god like I don't really like talking to people.” I'm really anti-social so, you know, so maybe you can get people to talk if you warn them at the beginning, like, okay, well, this is not gonna, you know,

34:45

I: Yeah, like, so we like re-going over that thing from the consent. That's like this is totally confidential.

34:46

P: Like yeah

34:51

I: Yeah, you're totally right. I should have definitely started the interview with that that's actually part of this script and I just brushed through it.

34:57

P: That’s okay. I understand

35:02

I: You should be a researcher because you have a natural inclination.

35:07

P: Yeah, I'm telling you, I really do. I read a lot. It's just that I'm, I'm a really lazy person.

35:08

I: Well, there's time to be not lazy later when you're not-

35:17

P: Right, yeah. Yeah, yeah, you're right. Well, I definitely did enjoy having this conversation because I know a lot of people don't talk about it. And, um, it does need to be talked about, talked about in my opinion because it's not, you know, it can affect certain people, you know. And to me even though I don't do it to see another mom or another pregnant woman do you know smoke or anything around their children, it does make me feel some type of way, because you know that's not good.

35:39

I: Yeah, yeah. I mean, I just, I have really enjoyed all of your opinions and like this is exactly why we're doing this study is because we like, like you, your experience has so much to teach us.

P: Mhm.

I: So yeah, it's been a real pleasure this morning.

35:56

P: Yeah, and I appreciate it. Because, I mean, I don't really do anything I be in house all the time. I watch TV and I sit on the couch and eat. That's it. So this kind of game you some free time, and it can make me feel better because, you know, I don't really- my boyfriend works all the time. So, you know, feels good to talk to another female, you know,

36:27

I: Yeah. Yeah. Yeah, definitely. It's enjoyable and we have so many other parts of this studies. So, if you let us will keep you very busy.

36:39

P: Okay, yeah. Yeah, I understand. I think, um, I think her name was Nicole, she explained some of that yet to me yesterday. Wait.

36:51

I: Me, I'm Nicole. But…

36:56

P: Ohhh. I’m so sorry. You're like wait, yeah, I'm Nicole. But

37:05

I: I should have introduce myself. You’re like schooling in me and all these very basic manners like-

37:15

P: I'm sorry I Really, oh wow

37:18

I: Don’t apologize. It's so good, I feel like, you know, I feel like this is only my second one of these interviews. So I came into it nervous and like, you know, usually I would introduce myself, but I just

37:31

P: Oh okay. We all have one of those the mornings. We all had those mornings, we forget I know I forget a lot. So yeah, I understand. I should’ve introduced myself to you because you didn't know how to pronounce my name, so you know

37:42

I: Make it like a Spanish, which is like, why would I do that, it's very obviously (participant’s name)

37:53

P: That's really- that's really funny because a lot of people really do think, my name is like really weird. And it's like, literally I understand though because I, you know, I pronounce people's names wrong sometimes. So I understand, but my name is spelled the way you say it.

37:54

I: It’s very like what's the word? Phonetically. We literally said this to each other. We said (participant’s name) that's such a pretty name and then-

38:19

P: Thank you.

38:24

I: Yeah, it is beautiful.

38:25

P: Yeah, my mom puts on her name and my father's name together to get that name. Yeah, my dad, my dad's name is Brandon. Yeah, and her name is Jacqueline so she got the [Participant’s name] from her name, and she got the [Participant’s name] from my dad and the [Participant’s name] and just put it together.

38:50

I: Like so unique, but it actually it doesn't sound like-

38:51

P: Weird or anything

38:55

I: Yeah. Yeah. Yeah, it's like oh yeah of course that's the name

38:57

P: Right. Yeah, exactly.

39:00

I: Well, I hope that you have a good rest of your- I don't even know what day of the week. It is Thursday.

39:07

P: Oh, yes. Thursday.

39:12

I: Thursday and do you have any questions or anything. Before we go, I am going to pay you. I'm going to put $20 on your card for you.

39:14

P: Yeah, I'm not worried about but, um hmm. No, not really. Um, what we talked about is like when when my doctor know about this too?

39:28

I: Like, like, would we share this information with your doctor?

P: Yeah

I: No, no.

39:39

P: Oh, okay. All right. Um, because I was thinking maybe my doctor will notice too so they can stop asking me about smoking because I don’t smoke. But, um, but yeah, that's okay. That's okay.

39:51

I: That's funny because we keep it like very separate from your doctor like very intentionally don’t tell your doctor

39:55

P: Yeah. Yeah, um, that's okay. Anyways, um, I mean I can keep I guess I'll keep answering their questions about it.

40:03

I: They ask every time

40:10

P: Yeah, every time, but

40:11

I: the same question about smoking?

40:13

P: Yeah, I mean I feel like they do it at the beginning a lot because you know I don't know, I feel like they just do it in the beginning a lot. Because, I mean, this is my first pregnancy I don't know how many times they’re going to do it, how many other times they're going to do it in the second trimester or third trimester, but I know in this trimester they do ask lot but understand what I do because you know some people really do not care. And they do it anyways. Yeah.

40:45

I: It's, it's annoying that they would be the same exact questions over and over again.

40:45

P: Yeah, yeah

40:49

I: Yeah, but I do get why they, you know.

40:51

P: Yeah, I understand. Yeah, I definitely understand 100%.

40:54

I: Yeah. Well, I will. I can't wait to talk to you again in your second trimester and I really wish you in the baby like the most healthy pregnancy journey you can have

40:59

P: Thank you and I can't wait to talk to you again. Um, I definitely I really did enjoy this. I really did. That, like I said, I don't do anything. Yeah. Thank you.

41:20

I: Yeah. Thank you. Have a good day. Me too.

P: Thank you. Bye bye.

41:26

Okay, stop recording
