# Supplementary material for: Experiences of postpartum mental health sequelae among black and biracial women during the COVID-19 pandemic
Source: BMC Pregnancy Childbirth. 2023 Sep 4;23:636. doi: 10.1186/s12884-023-05929-3 (PMC10478375; doi:10.1186/s12884-023-05929-3)
Supplement: Supplementary file 22 — Supplementary Material 22 [file 12884_2023_5929_MOESM22_ESM.docx]

**Supplemental File 1.19 Interview Transcript with Participant 5214**

SM Quali 5214

4.26.22

Interviewer: 50

OK, so the. Beginning it's just like kind of about your pregnancy in really in general and about like what it was like for your body when you were pregnant, so can you just tell me a little bit about what it was like for you to be pregnant.

Ami Michielli: Um well in the beginning, I told you, I was a um a drug user. So, I, it did not feel real at all. And I was very small, so I didn't get a belly until, like six months in. You know, like at all. I was very skinny. You know, I didn't, I didn't even acknowledge that I was pregnant for a while and so I didn't know-- it didn't have a feeling. But then once, I, you know, got clean and was acknowledging that I was pregnant, I got my belly and it was amazing. Like, I loved it. My body was tired, but, you know, yeah. It was very nice to me, though, like I got really clear skin really long nails. I didn't get my stretch marks really until the last month. Yeah, so I don't know if that answered the question but.

Perfectly I also wonder like versus so interesting to hear you describe it as just like. Look at first you just didn't really think about being pregnant like it was it kind of on your radar until you got clean and then I was wondering like you know it seems like pregnancy really agreed with you. then also part of it i'm thinking about like you know when you get clean from stuff like you just get that glow kinda. Right and i'm wondering if that was part of it, too, is like all these positive forces.

I think it was, I really think it was, you know, and I started on my Prozac which depression medicine very soon after. Oh.

I think you muted.I cant hear

Okay, my microphone is unmuted my video has stopped, I want to go back did it work.

I can hear you now, can you hear me.

Yeah, sorry, she just grabbed my phone. Um, yeah, so, I started with my Prozac very soon after I got clean. And, you know, all of that put together just made my pregnancy very like great, amazing, positive like, you know .I don't know, yeah, so.

What were your like Okay, so you, you know you're going through kind of not only were you pregnant but you were getting clean and you're starting some kind of like medicine. So what were some of your like Healthcare experiences like during that whole time?

Um literally, the only doctor that I saw was Ms Stewart actually. She was from mcgee women's hospital. She was like the gynecologist, the pregnancy doctor, I didn't have a psychiatrist, I didn't go to my PCP none of that um because I hadn't really been to the doctors and two years before that, so I was, you know, really not in the loop of like normal routine with doctors. The only, you know, the only doctor routine I was in at that point was starting my pregnancy one so I only saw her and she was amazing. I didn't want to see any other doctor um in the. And oh there was another one, Ms Cami, and she was amazing. She was at Magee, they were just really good so. They were very understanding of my situation, they weren't judgmental at all, you know, very helpful gave me all sorts of resources. Um, yeah, they made the made the whole transition process very nice and smooth so.

that's so nice to hear.Did you have any you know, maybe not with your regular doctors but when you were in labor or anything, any experiences that you werent just super comfortable or you just didn’t feel like heard or during or was it all positive?

Um, actually my birth experience was pretty traumatic, but it wasn't all the doctor’s fault, because she was stuck on my pelvic bone. So they they were giving me pitocin, and I was induced, so they were giving me pitocin and every time I was put on the pitocin, her heart rate was dropping, but I was so tired because I was in Labor for like 16 hours at this point, and before I got the epidural, and I'm so tired I was trying to go to sleep and, like every hour or two they would run in and be like, “get on you back, get on your hands and knees, get on your hands and knees, hurry up we can't hear the baby blah blah blah!!!” And I was like asleep, not even and theyre just like rolling me over like a fat cow or something, it was ugh very traumatic. But it was always a different doctor and I didn't like that part of it, it was always a different doctor, or different nurse or, you know. There was no real one on one like intimate connection with a doctor or a nurse. And now like that I've gone through that, if I go through another pregnancy, I will not do the same thing, I will go to like a midwife Center or something, because I don't really like that. And they were all telling me different things, “oh you're one centimeter dilated”, “no youre two centimeters”, “no you're a half a centimeter dilated” and it's like, well which one am I?

Sounds also like it must have been a lot of people checking too.

Yeah it almost felt like it was consistent, like constant, like not consistent, constant checking, just fingers all up in me all the time and like, you know, ugh I just it was hard.

What was the bedside manner, like you know if someone's basically manually checking your cervix they're sticking their fingers inside of your vagina, did they ask or what was that conversation like?

Um, I think in the beginning, I don't know their names or anything like I said, there was a bunch of them, but in the beginning, there was a nurse that was very, like just very calm, and like that, asking, “oh here's what we gotta do next, are you ready?” But really every nurse after that, like, especially the overnight nurses, I didn't even know like. They were just like. it's time for your cervix to be checked, boom, go back to sleep. And I think they were, I think they were doing it to try to like, you know, not interfere as much with my sleep, as they could, you know and, at the time, you know, and now I really don't think it was like invasive, it didn't feel invasive but it definitely wasn't intimate like I said, you know talking it through, stuff like that. So, it would have been, it could have been nicer had it been that way. You know But I didn't it didn't make me feel any kind of way about the process, I wasn't sleeping anyways, though, you know, they were coming in and check my cervix so. Had they been more like, “oh its time to do this,” so, more like walking me through the process instead of just doing what their job was, you know?

It's interesting like you said it's not necessarily invasive but there's probably a few things that could happen to make it a little bit better for you. Right yeah or you're gonna hear my dog like screaming in his sleep.

It's okay i'm sure you hear my daughter daughter over here gurgling.

She sounds so happy.

She's not just sitting here either like I wish I could have done the video, but I just look crazy, she's over here like jumping across my legs trying to get to the phone. And I keep wishing her back that's the stuff.

That you're able to like communicate so clearly while also juggling a phone and the baby.

Thank you. Im trying

So this is, I mean it's not a curveball but it's a little bit of a different question so did you have any doctors or people in the hospital anyone during the whole healthcare experience ask you about your sexuality your sexual orientation.

Actually, uhm maybe my first visit with the doctor, honestly, I don't know. Actually, yes, I did. And she asked me because when I had come in for my check, I had trichomoniasis or whatever, you know the STI or whatever, and I got treated for that and she asked me, you know, or she didn’t ask me but she just kind of said, “you shouldn't have any sex partners. I don't know what you're interested in men, women, but you shouldn't have any further six weeks or whatever.” And I answered, and I said, “yeah, I don't, I won't have any.” You know, like it wasn't a question, but it was brought up for that split second.

that's interesting that she would include that, how did you feel about that?

I thought it was pretty cool, you know, like she wasn't making assumptions. And i'm not one to be like “oh you're assuming who I am or what I am”, I'm not one to be like that at all, you know, cuz I don't even know who I am so. I thought it was really cool because, you know, she could have just, i'm sitting there pregnant, obviously I had sex with a man, you know, she could have just been, “ oh well, you cant have any sex with men”. You know, I don’t know, I just thought it was very open, kind of like were living in 2021, you know.

yeah and clarifying like she you know she didn't leave it. yeah I like. that she it's also really good education, because, like I wouldn't have known that necessarily.

Yeah, exactly, you know, and I was, I wouldn't have expected that from her either, you know or any doctor. They just assume that I am straight, especially if they were straight. You know, I wouldn't expect them to acknowledge my possible other sex partners, but you know, it just seemed like she was covering all bases, without being rude or invasive of my personal information, you know.

mm hmm do you think that, like they should bring this up kind of an Ob gyn here like they shouldn't be asking people sexual orientation.

No, honestly, no I don't think that it's important. Um I think that, and I don't know me as a patient, I'm one to be pretty open about the situation at hand. So like say I had gone, I thought I got the STI from a woman. You wouldn't have to ask me to know I got it from a woman. But um you know, like you, wouldn't have to ask me what's your sexual orientation for me to be like, yeah, I think it was my girlfriend or something that gave this to me, like you know. But, I don't think either way it's important who I got it from or what their gender was anyway, so you know. I mean, I don't think that it's important for them to bring that up or ask specific questions, you know because they don't need the answer to them to do their work.

When you said that like your baby you like a bunch of resources when did you were any of them like related to being bisexual or what resources were like really helpful to you.

No, none of them were because im not actually bisexual, um I don’t even know, I don't put labels, but if you want to put a label on it I am sapiosexual.

Is that what you want me to like refer to us as the interview, I can.

No, you don't have to, that's okay. Like I said, it's not important. It's, you know, the difference is when you are sapiosexual, you're attracted to way to the way people's minds work and the way they view the world. It's not about gender or what they have between their legs or what their sexual orientation is or anything like that so. You know, it's, it's okay to just say bisexual because ,you know, whatever for me.But what was the question. (laughter) i'm sorry.

No, that was that was very clarifying. I I like wish I would have asked you at the beginning, what you were most comfortable with in terms of like terminology, but um and I can see how like Is it safe to know that gets collapsed into like bisexual just like, how can sexual gets collapse into bisexual. yeah yeah um, but the question was about resources like what they gave you that.

Right, so, like I said, you know, I was struggling with my drug addiction and I was homeless, living in a whole nother state from my mom. I actually moved up here to be back with my family, and so it was all new to me, so the resources were like a genesis program or things to help me with being a mother, you know not with my sexuality. And I actually think that, my personal opinion is that, that was awesome because my sexual orientation had nothing to do with my situation that I was in and what I needed help with. So, you know, I don't know, had I been somebody that needed like resources and help with my sexual orientation than it might have been, you know, like they lacked resources because they didn't give me anything like that, but you know I wasn't in that position in that situation. So what they did give me was very helpful and helped me know that I had options and I had support with the things I was struggling with most, which was life and becoming a mom six months into the game, you know. Where i'm feeling like i'm running out of time and I don't have anything ready, you know. So I could go take these classes and get diapers and get a diaper genie and get a crib and get a car seat, you know things like that. I didn't even have a job, so I. So I couldn't get all that stuff if I wanted to you know. Things like that so.

It sounds like the resources more so helped you with like direct things that you actually have to have to have a baby.

Right.

Yeah is genesis is that, like a just like a housing program for pregnant people, or is there a substance use component to it.

No, I don't think there's substance use component to it. I did get resources for that, too, because I was honest with my doctors about my substance abuse and things. But, I had went to one na meeting during my whole sobriety journey, it just wasn't for like for me. And it was very overwhelming actually to get a whole bunch of substance abuse resources because it felt like I couldn't handle so much information, just so much so many things that I needed to handle. And I couldn't do it, it was just very overwhelming, so I actually had dropped completely um the idea of working on my substance abuse and I focused on being a mom which actually ended up helping. I did, you know, quit my substance for that reason. But you know. Its was nice to get you know but so they did give me that yeah.

You it sounds like you kind of have to like wade through a lot of resources, but like in terms of substance use kind of find carve out what you needed.

No, I think that, I mean, I think that, I think that's how it is like with anybody substance abuse, though. Like, its just my personal opinion, but you got to do that anyways. Because, you know, people can help, can offer you as much help as, you know, they're able to, and as the world allows, but if you don't want it or if it's not resonating with you, it's not going to help you. Whereas somebody like with the genesis program or something where they give you products for your baby, you know, it's help that anybody can take. You know, it's just useful, 100%. Whereas with substance abuse help, it's almost like you have to want that help in order for it to be of any use. Yeah, I think they gave me plenty of resources and it was just because of me personally and how I wanted to approach it that I didn't use any of the resources and I found it overwhelming, you know. But I think it was still a good thing that they did. Andthey offered me, you know, like ( she's playing with my toes i'm sorry) You know, I think they offered me, you know, like therapy behavioral therapy and stuff like that in the hospital and everything, so you know I think actually got more resources for my substance abuse than I did for anything else I just didn't use it.

What i'm hearing is like what you really needed was like the resources to be like a mom because that's what you were focusing on and like what was next for you.

Yes. You know, everybody struggles for their own reasons with substance abuse, you know, like it's all for their own reason and a part of my reason was definitely being overwhelmed with life, you know, like just not knowing the next step to take, and you know, just shutting down because I was overwhelmed and just didn’t know how to handle anything. So, yeah getting all those resources was kind of just overwhelming. It wasn't what I needed. I needed to focus on something that was real, you know, and substance abuse is real, but I needed to focus on something that was happening tangibly, that was really the best way for me to heal from my substance abuse my way, so. That is what ended up helping me, was focusing on being a mom

(you're sticking their fingers in my ears....some talk to the baby from us both blah blah blah)

So this is the last like sexual orientation happens in question ill ask and then ill just move to the tobacco question and ill probably ask one or two marijuana question. So do you think being sapiosexaul affected your pregnancy at all?

Naw, nope. It really doesn't affect anything except It makes me less sexually active because I find even less people sexually attractive than if I was just attracted to like body parts and sex itself.

So being sapiosexual, it can actually like kind of decrease the number of people you find attractive?

Number wise, yes, because I don't look at men and its like all of them are attractive because they're men and then I can just sleep with them. When its men or its women, it's about the mind, so I have to get to know you first, before there's even a possibility for me to find your sexually attractive so yeah.

So there's like a whole, and do you think being pregnant like slow that process down or just like you know you weren't focused on that so that's why.

Um, I definitely was not focused on that it. Actually it did slow the process down, but for me that was definitely a positive in a way, because I have so much going on already the last thing I need to worry about is trying to find a sexual partner. So it gave me something else to focus on, you know.

yeah yeah.

And kept it from happening because i'm out here trying to be a mom and not meet people to sleep with or to have a relationship with or whatever.

yeah.

yeah.

it's like the mom identity kind of like. Is a head of like not only like you know, a sexual orientation, but also like a substance you like you know someone that identifies like a substance users and things like that mom it seems like for you, like the mom just kind of like when it came out at six months and it was like forefront.

yeah it was and it honestly, it was like the best thing that's ever happened to me so.

(We both talk to the baby a bit here)

Okay, so let's do some tobacco questions. Do you remember, and I kind of love these because I think it's so interesting to hear people's like stories about their first use Do you remember, like the first time you smoked a cigarette.

I, do. Um, OK, so my mom was a smoker and she never smoked in the house, except for in the basement if she was on the computer, but it was very rare because she knows, like you know, smoke rises you don't want the house to smell everything like that. But, we were like, me and my twin, we were probably like eight or nine, and you know, my mom smoking cigarettes down there and we had our computer game which was a daycare nightmare, and I don't know if you've ever played that game. You know what that game is?

Yes!

So yeah we were playing daycare nightmare. And we, you know, our mom bought that little CD, downloaded it on the computer, all that, you know. And so, we're down there, and me and mia, were down there, and I was like, “look, we could be like mom she left a cigarette butt in the ashtray like Look, we can be like mom.” And I lit it and I hit it. But then, you know, that was it until I was like 13, you know. Then my friend Kirsten was, she was like, she's like the the emo bad girl, you know and so she was the person that people talked about in school like, oh she's a ho, but she wasn't, you know, people just-- she was very pretty and didn't really get along with people because she just wasn't a social person. Well, you know, we became friends, because we were both very smart and worked together all the time in school. Well, we started hanging out outside of school and she's like, “oh, have you ever smoked a cigarette?” And i'm like, “Yeah! You know, my mom’s in my moms basement I smoked a cigarette butt.” So we, but what she did to smoke cigarettes, we thought it was so cool, was to walk around parks and pick up cigarette butts off the floor---off the ground and light them! And that's how I started smoking cigarettes until I started making, well I asked my mom for like allowance for my chores, it was the first time she would pay us for chores because I asked her so I could buy packs of cigarettes and yeah. This constant smoking until I was, you know, pregnant.

There is just like something like so teenage about someone walking in a park picking up cigarettes and just smoking them, you know. Right yeah it's a Sir i'm like a little nostalgic, but also like now that there's a pandemic, I feel like I have a whole other perspective on doing that's.

Right. I look back on it, I'm like duh ugh like that's just disgusting like. We could have gotten sick, we could have had bird poop in our mouth like, everything I think of it all, and then just.

Oh yeah.

Totally, not worth it, because cigarettes are disgusting, you know, but back then, it was so cool.

And no concept of like consequently they're like.

Yeah so when you quit i've heard so many different kinds of stories about what it can be like when you're pregnant and smoke, you know, did you smoke cigarettes or was it like a vape or something.

No, I smoked cigarettes.

Okay, so i've heard you know, some people get pregnant and its like I soon as they do they are like I hate the smell, some people are like wanting to smoke during my whole pregnancy like what was your did it make you want to quit did you crave it was awake.

All the way up until I quit my drugs, I was smoking cigarettes all the time. That's all, like, that's all you can do whenever you're sitting like homeless in somebody else's house, just sitting around doing drugs, you know. All you can do is sit there, do the drugs, smoke the cigarettes, walk the block, whatever you know. So, you know, me being pregnant didn't have any effect on that, but then, when I got clean, it was literally one daym I was already up here, you know away from Georgia, you know that’s where I was doing my drugs and whatever, and I was still doing my drugs up here, you know um and, one day, you know, I look at my sister and Im like, “i'm fucking done.” Like, you know, I’m just done. And I didn't touch my drugs or my cigarettes again. Until I had her.

It was cold Turkey.

It was. Actually, it was because the day prior I had read an article that said, you know, because my choice of drug you know, whatever I don't think I need to say that or yeah well, okay well, i'll tell you it's fine, but you know my choice of drug was meth and heroin. You know, I quit heroin a couple months prior to, but you know, like meth, you know, I was looking it up and it said in the article like it can stay in your placenta, which is like you know the nutrients for your baby and stuff, for like four months. You know, three, four months. You know people quit, it said, like people quit doing the drug and their placentas tested for drugs and it still shows up positive months after. And like, it wasn't even about the being positive for drugs, because all my doctors knew that I was a drug user, it was about that it means that’s in my baby system, even if I quit right now, it's in my baby system until she's fully grown. You know, and I was just like I can't do that. Like that, it just hit me just so real and so like hard. I was like oh my God like, i'm pregnant, there's a life in me and even if I quit today, she's gonna still be affected. So that's when I quit that and I quit cigarettes, you know. I put down cigarettes, because I, you know, I just wanted what was best for my baby, it was literally in that one moment I was just instant mom so. And thenvafter I had her, you know, first thing I did was smoke a cigarette. (laughter) After I healed a little bit, I had a C section, so I was like pretty out of it for a while and then my sister came to visit and I was like please take me outside in this wheelchair and give me a cigarette. I smoked one, and then just that one or two times while I was in the hospital, and then I started vaping when her dad came to visit me. But, yeah. I mean, even now, you know, like I vape but I don't smoke cigarettes. I cant. They are disgusting to me, she doesn't (motions to her baby), she I don't allow for in the room, with cigarette smokers or weed smokers, you know my sisters does all that, but you know.

So do you prefer vapes now to cigarettes?

I do, yeah. I want to quit the vapes, you know. I’m kind of upset because I wasn't doing any of it, I wasn't doing any of that until her dad came to visit me. And then you know, he was like, “oh look at this vape”. You know I want to quit it, but I kind of almost feel like I have more of an addiction to this vape than I ever did with the cigarettes. I spend more money on them and I hit them more often, because, you know, you can. I have this like, I don't know, I feel like I can smoke them in the house because they smell and they taste good. You know? I don't have to walk out. I don't have to walk outside to smoke my vape so i'm smoking it more often.

Ive also found that when you vape, it doesn’t give you the same kind of sick feeling as cigarettes.

there's a queasiness that comes with the kinda like a nauseous start again.

Yeah it doesn’t usually but it will depend on the vape. I honest have had that happen to me before but I don’t think it’s the nicotine it mores like oh my god, I am dizzy, did I just inhale some sugar?

Is there anything that like makes you like crave the vape or like trigger you want to use it?

Driving. Yeah. Driving. Like when I drive, I have to have a vape otherwise i'm like uncomfortable driving, like zoned out driving. Because i'm just thinking about how my vape is not in my hand or something you know, like. Like almost fiending for it. Before bed, you know, and then after I eat--so pretty much like cigarettes, you know. But's not stress, you know, like if i'm stressed out i'm not like Oh, let me go puff this fruit bar like, you know, it's not like that you know. And there's not, there's not a social aspect to it either. I felt like with my cigarette smoking, it was also a lot of social aspect with it like, if I had been around anybody, even like if they came to my house or something and we hung out and then they left, I have to smoke a cigarette now because, like I just went through this social experience, like you know. It's nothing like that, you know.

Yes! Did you...so her dad is the one that kind of like introduced you to it, because he vapes.

Yeah. Cuz he quit smoking cigarettes way after I did, because he was in jail he quit. And then, when he got out, he was like, “I went this whole time without smoking cigarettes, I don't want to start smoking cigarettes again.” So he picked up a vape because he just wanted to smoke something, I guess. Well, then when he came up to visit, you know, I didn't allow any smoke around her because I wasn't smoking anything, you know, and he was like “Well, I have this vape. Can we step outside?” And I was like, yeah sure and brought the baby outside, you know, and he's like, well---he showed it to me and he sitting there hitting it and I was like, “hey can I try it, it smells good.” and that's when I was like Oh, this is good! And then he bought me one and he spent like four days up here. And so, we were just smoking, the vape for four days, well then, when he left, like right before he left, he bought me another one and I found myself smoking it without him here. You know what I mean? It started with me going like outside to hit this vape but you know everything progressed from there.

When you when you were pregnant, did you, did you like crave cigarettes when you were pregnant at all?

No because I completely removed myself away from anyone who was smoking them. Yeah, I treated it just like my, just like the drugs. I isolated myself, it was just me and my mom. And, you know, my mom doesn't do any of that anymore. She had quit cold turkey like nine years ago, sorry seven years ago. So, you know, we don't do any of that here, none of it, so. Yeah. You know, I didn't go around my sister, I didn't hear anyone talking about how they wanted a cigarette or oh, can you buy me a pack of cigarettes. I didn't hear anything about any of it, and so I had no craving for it, no, like, nothing to bring it up in my mind. I was solely able to focus on my pregnancy.

And then, when you had her it was just kind of like this is like the light bulb turned on or were you like thinking like planning like okay once I deliver her i'm going to have it, or was it just spontaneous.

No, it was like spontaneous because I saw, you know, my sister she smokes cigarettes, she came to the hospital to visit me. It was actually her and our friend Kirsten, the one that got me, you know, that we started smoking with or whatever and I told you, my birth experience is very traumatic and I was just very stressed, I had to have an emergency C section, it was just all so much, and you know, that was just like my moment of weakness, I guess, you could say, and it was there in front of me. That was strictly to relieve the stress and then it didn't even do that, like it was just gross but I did it anyway, like because we were all outside smoking and whatever. That's why I didn't pick the habit back up because it was really gross tasting and everything to me.

Yeah, yeah that makes sense to me.

Is there anything that like I don't know your Ob gyn or anyone in that system could have done to help you quit over or reduce your smoking.

Um, Im sure that a lot of people benefit from what they already do because there were a lot, like, like I said, a lot of resources, a lot of, you know, offering of help, but you know me personally, i'm kind of like a stubborn bull, i'm a Taurus so, you know.

Awww happy birthday!!

Thank you, on april 20^th^ I turned 21.

Well, happy birthday!

Thank you, I don't really get along with my twin, but, I know some tauruses that I like. Um, so, yeah i'm just very stubborn headed and like want to do everything by myself. If it's not my choice if it's not my doing, then it's not even like it's a setback, but it's like when people were telling me, “Oh well, here, we want to help you quit doing your drugs, quit smoking cigarettes here's all the things”, it's almost like I will quit when I want to quit, I need a cigarette now! Because you just like keep bringing it up, like you just keep telling me that you're here to help me quit but I didn't ask you for help to quit. I’ll quit when I want to quit, i'll quit when i'm ready to quit. It's just how, like, you know, my mind was working. I appreciate it, though, like now I appreciate it, but in a way, it's like subconsciously it was almost like bringing out that defiancy in me and like now I kind of don’t want to quit because now its what everybody else wants.

Yeah that is a good insight.

Yeah, you know. And, yeah every time I went to the doctors, “did you quit smoking cigarettes?” or “do you smoke cigarettes?” They ask you the question every time. “Do you smoke cigarettes?” and “how many?” And then I would tell them, “yeah but i've been, like, starting to quit though, you know.” When they're like, “oh great! do you need resources on quitting?” Im like, “No, I don't.” I just said I was quitting myself, you know? I just, i've been down to less than a cigarette a day, what do you mean I do I need your resources, you know, like it was almost like a buck that type of thing every time. Yeah, but you know that's just for somebody like me. I know, I know people who those resources help you know, and it makes them feel like they have support, you know, and options.

Yeah but it sounds like for you it's like when you want to help you want help and that's when you want help and until then. You don't want it.

Right because i'm not stupid is how I feel, you know. Like i'm not stupid, I know, like the consequences of what i'm doing. I know my options, I know, you know, the choices i'm making. Does that make sense?

yeah.

You know, not like I know it all, but like i'm aware of what's going on and what I should be doing, and my options. So when i'm choosing to like smoke cigarettes, you know, it's not because I don't understand that there's an option that I don't have to smoke cigarettes right, you know what I mean so. And that that really mainly stems from my substance abuse and how everybody around me handled that with me does that make sense, not so much cigarettes. If it was just cigarettes, whatever. But it was like almost like a triggering thing from my drug abuse. You know, everybody trying to fix my situation, I know what situation I'm in. You know, i'm choosing to do this for reasons that you don't know. So, you know, what I mean? It was almost like a hostile type of, I have to defend myself all the time and i'm defending actions that I know are wrong, so it was just like all around negative behind anyone trying to help me. Does that make sense? So if I didn't want help, if I didn't seek out the help myself, that's how it made me feel.

Yeah more like an attack or an insult or just something kind of negative or even just like pressure

Right, exactly, a pressure. Or like Im trying. Yeah exactly.

yeah yeah that's interesting that is not something I would have thought, but it seems now you know it's like one of those things like when you say it or someone else says that it's like Oh, that seems so common sense, but I wouldn't have put you know those things or experiences together in that way. But It seems obvious hearing you say it now.

Right.

Okay, so I think this is there's like two more kind of quick sections, the one I know you didn't use marijuana is not your drug of choice I get it i'm just wondering like kind of why, like, I want to know about your first time using marijuana and then like what makes you like not like? it does that make sense?

Yeah can I show you something real quick?

Yeah!

See how she was just acting crazy? (Camera pans to sleeping baby)

Awww little sleeping angel. Does she usually sleep right now?

Yes, she usually, well she woke up pretty early this morning, i'm not gonna lie, so this is a well needed nap.

Aww yeah.

Okay, yes, so I used to smoke marijuana all the time. It started, you know, I didn't think first of all, I didn't think marijuana was real and it only happened in movies until I was 14. (laughter) And I moved to Georgia, and my boyfriend, my first boyfriend ever, smoked weed. Um and he showed me how to smoke it out of a pepsi can, yeah (laughter). And so we did, I did that for a while and he gave me the weed and I mean, you know, we smoked it together. I would smoke it at home because, like you know, yeah I liked it, I loved it. And for a while I did that and then it was, it was I know I keep going back to like my other drugs but it literally was because they changed my life, like they just changed my life. When I did meth for the first time it chemically changed my brain. And so, after the first time of me doing meth and then trying to do weed again or smoke weed, it gave me, like, anxiety, it made me paranoid. And I started, not just like feeling anxious or anything, I started seeing things and I saw my sister tell me that it was my time to die, she looked me my face and told me, it was my time to die. Oh yeah, and I started freaking out and i'm like, like cussing her out, you know, how could you say that's me, that's my twin sister the person I trust the most. And, you know, she was like, “Amy, I wasn't even looking at you, I was talking to our little sister tyra, like I don't know what you're talking about.” And I finally snapped out of it, and I was like, Okay, maybe weeds not for me. So yeah. So that was about when we were 16, so I smoked it for a few years, and then now, you know, I can't, I just can't do it, it makes them paranoid or anxious. Sometimes it's not all the way paranoid, it's just anxious, and I have some horror stories about that, like me, trying to smoke weed again but that's just the overall gist of it, it just makes me too paranoid.

Okay, so like it was good for two years. You did this other thing that was like actually really good for you. You know like the experience you prefer, and then, when you try it again, it was just like you legit couldn’t.

Yeah. Yeah. So, I had actually, it's funny that you like brought this study up, I had just signed up to get a medical marijuana card a couple days ago. I was going to try it, you know, because street marijuana is different than dispensary marijuana. They have strains and, you know, hybrids that are like, I don't know, just made to, like, give you certain effects and there's anxiety relief ones and there's, you know, ones that help you sleep and then there's ones that make you feel creative and energized, and so I wanted to do it the right way, and maybe that, you know, would help with some of my issues. And so, I signed up and the day I signed up, you know, I smoked with Kirsten, she smokes weed too. She has dispensary weed and she gets it for her anxiety. So I was like, oh well, that, you know, maybe I can just try it and it was good, it was okay, you know, I did fine. Yeah, you know, I did fine actually, I really enjoyed it. I slept, I didn't sleep that great, actually, because my daughter sleeps in the bed with me. And sleeping I don't know, I guess, because I was high like I didn't want to be like crammed so I felt really uncomfortable sleeping that night but I went to sleep okay which is something I usually don't do. And when I woke up, though, the problem was that I felt like I was on a come down. And me, you know, being a drug, you know, recovering drug addict, like, that's not something I want to do and I don't want to smoke weed consistently because that's just creating another addiction, you know what I mean. So, you know, I talked to my mom and she had already paid for me to get the like psych evaluation and everything and to get the card, and I was just like, you know, I think i'm going to call them and cancenl it because while it did work, I mean it felt nice the day I did it, you know, the next day, I felt like I was on a come down and that's now what I want. I don't want to go through come downs every day because I don't want to smoke weed consistently, so yeah.

yeah because its like the way to avoid a come down is to get high again.

Right and that's exactly the mindset it put me in. And that's, i'm doing really good, I have been doing very good without that type of drug addiction, you know, i'm doing very good, so I don't want to start something with that. Not to mention, i'm on parole, probation, um so you know, that is another reason why I haven't tried it sooner. I just got the okay that I could get a card and do it legally. Which is why that started but.

i've always wondered about that.

Yeah, me too. And I asked him the other day, and he was like, “yeah you can.” There's limits like I don't think you can smoke weed or something or like you have to do like them tincture drops. Um, hold on. Sorry, my probation officer is just texting me that was crazy um. You have to do the tincture drops or the oils, you know. I don't think you can do, like, I don't think you can smoke a blunt. But I, like, you know, I don't think you're, because I think like if they come and check out the place you can't have buds, like the flower laying around.

You know, I was wondering, too, if you could get your medical card, while you were pregnant. I have always kind of been curious about rules around that.

Oh, while you were pregnant I don't know.

I don't know either it was wondering. So weird that you can have oils and all this other stuff but no, no, no, no, weed.

You know, I wasn't even, i'm not even legally able to drink. Even though i'm 21 just because i'm on probation. But that's okay.

When is that over for you

Next June.

Okay. Do you think that, like your tobacco use marijuana use like your other drug use, do you think it's at all related to identifying as sapioexual like. Is there anything about that, that like connects those two things?

Um, the only, like, connection that I can put to it is like the type of people that being sapioexual, like, attracts me to. Does that make sense? Like her father. Ugh, you know, this is this is harder for me to talk about than my drug addiction, but i'm 21 and he is 43. I met him when I was, I had just turned 18. I was already a drug user but um not to the extent that I was like while we were together. But, you know, me being the way that I am and what i'm attracted to and my life prior to being a drug user, um, I never encountered anybody who went through the type of struggles he did; being a drug dealer, coming from a family of drug users, being a drug user since he was 16, you know, and all the experiences that come with that, you know. I've never met somebody like that. I've only ever seen those type of people and things on TV. So, when I met him, it was like I was instantly like interested in his life and who he was. So, then, you know, then he would tell me about it, and you know, that made me attracted to him, you know. It made my, you know, drug addiction worse because then like, you know, he shoots up drugs so that's what I was doing. And he was a drug dealer so I was just getting it supplied to me and I gave up my life to be with him because I’m like, oh my God like this soul needs saving, this soul needs to be loved because he's been through so much, you know. Like things like that. Yeah, I feel like if I wasn't like interested in that kind of thing, if I wasn't sapiosexual and I was just like interested in men, and it was the just that, like, I don't want to say basic, I don't want to you know talk about people's sexuality that way, but, you know, if it was that that broad of a category, you know. I feel like he would have never even been on my radar. Does that make sense? And I wouldn't have gotten pregnant and I wouldnt have been a drug user like that thing, you know, all things of that sort. So that's the only connection that I can put to that.

I think that makes, I mean it makes perfect sense it paints like a clear picture, and I also think there's like a lot of like You know you gave like one example in your life, but like what I think you're talking about is also i'm feeling like it's like pretty transferable to a lot of other people situations like it's more about like maybe what my sexuality does to my social system and, like who I befriend and stuff like that.

Right.

Thank you for sharing that. The last part of the interview is kind of I think these are like the there's like four questions I think they're kind of abstract So if you need me to like reframe them or if it's just not clear to you, let me know. We call these the ideal world questions the perfect world questions so The first one is, what do you.

Oh that’s going to be really hard.

Ha really just wait. they're really tough So what do you wish LGBT Q plus or sapiosexual women knew about pregnancy.

Ooooh. Can you give me like something else to go off of? Like a different direction?

Yeah, I think the next one is a good place to start. What do you wish all health care providers knew about lgbtq or sapiosexual women?

Let me think. I have no idea.

That’s alright, there is no right or wrong. You don’t have to have an answer.

Its hard, because I don't think that, you know, when it comes to doctors that, you know. Whatever, I don't think that they really need to know much about people's specific sexualities. I feel like that's like a problem that we have in this world, honestly. That everybody wants so much detail about their sexual-ness (laughter)to be known and understood when these details are stuff that, like, the only people that, you know, need to understand them are the ones that are engaging in them with you. So, like, people don't like, I don't go around explaining to everybody my sapiosexualness, you know. Like the only people that even know that i'm saying is actually like you, because we're doing this study and, like my partners.

mm hmm.

You know, so because, you know, when they want to know, like why I won't sleep with them or why i'm with them, that's a conversation to have with them. But, like my doctor, even my OBGYN, you know, like she doesn't know that i'm sapioexual. She doesn't know whether I have sex with men or women, you know, like she doesn't know that unless it pertains to what I am doing. Likem she knew that I had sex with a man because I had a baby. (Laughter) You know, I didn't explain to her, oh yeah I had sex with him but my relationship now is with a woman because I love the way her mind works, like, you know, Like, this is not a topic, this is not something I discuss with everybody and anybody, so. I wish that, you know, like I don't, I don't wish that they knew more about any of it, honestly. It's always great to educate yourself about the different types of people in this world, you know, just so, then you can be more tolerant, more understanding, more accepting of the people you encounter but I don't think you need to know specifically, like specific things about something that doesn't pertain to you. You know? Like, I don't think I need, I don't think my doctor should educate herself on sapiosexualness in order for her to be qualified to treat me for whatever I am in there for. Does that make sense

makes perfect sense is also really good answer because it's like. I mean it's just like honest and I think it also kind of rephrase it in an important way where it's like you know what you're worried about is like how they can like what they need to know to treat what you're there for.

Right.

yeah that's yeah I get it. And then, so this is the last one, but i'll combine them kind of because I feel like the last two are they the same question So what do you wish all maybe sapioexual women knew about marijuana and tobacco use or substance use in general.

That they change. OK, for sapiosexual women, I could say this, and it goes back to how I answered your one question about, you know, how it affected whatever. So. They should know that marijuana, drugs, tobacco use it doesn't change, I mean it changes people but there's a reason why people use these drugs, there's a reason why people do that and that reason, you know, is who they are. And so sapiosexual people are attracted to people's mindsets and how they work, you know. How do I word it? They should just know that some people who are doing these things are traumatized, sick, you know, hurt broken people who will in turn and can in turn hurt you. They're not, you know, they aren't people who like, like I told you my baby's father--had I known that a traumatized soul and a hurt soul, you know a broken soul, you know what I mean, that's what he was, and he had no capability of loving me, caring for me, caring for this baby, you know, being a good person, a good friend, a good associate. Like you know what I mean? Had I known those things about him and been able to see that like him being a drug user was like a sign of that, like not a red flag because it's not like I want to say that it's a red flag for everybody, but you know, was a pointer to that, you know, then maybe I wouldn't have pursued him in the way that I did because he's not sapiosexual, you know what I mean, so he wasn't with me for the same reasons I was with him. And I was with him because I thought he was just so amazing, like just like so interesting and so real and raw and been through so much, but that in turn, made it like it put me in a lot of horrible situations with him, with my drug use, with all of that, and the only reason I was attracted to him is because I am sapiosexual and that's what I look for in people. So, had I known that I would have, you know, been able to acknowledge that, I would have been able to see that and maybe moved on, maybe, like you know, maybe chose a different path to go down with somebody else who wasn't a drug user or who, you know, learned a little bit more or just knew what I was in in for, does that make sense?

It makes perfect clear sense. And it like really does echo the other question about like the connection between the. Two and like what and I also think it just like good in like a good advice and a suggestion to someone in a city like that might be a similar situation. yeah because I do think you know if you're someone that's like attracted to someone's like story in their mind, then, like your suggestion to maybe like. You know, see the other things too.

mm hmm.

could be like you know, a powerful thing in terms of like what their substance use patterns might become. Right yeah. Do you think there's anything that like I could have asked differently any questions that I, like you know, maybe insulted you asking like anything I can do better for the interview.

um no, you did not insult me, you did not offend me, you are actually very. Like you made it a lot easier than most people do to talk about these things, because I could just tell that like just the way you approach it.
