# Supplementary material for: Experiences of postpartum mental health sequelae among black and biracial women during the COVID-19 pandemic
Source: BMC Pregnancy Childbirth. 2023 Sep 4;23:636. doi: 10.1186/s12884-023-05929-3 (PMC10478375; doi:10.1186/s12884-023-05929-3)
Supplement: Supplementary file 23 — Supplementary Material 23 [file 12884_2023_5929_MOESM23_ESM.docx]

**Supplemental File 1.16 Interview Transcript with Participant 5257**

SM Quali 5257 10/28/21

I: Thank you so much for doing the interview. The first question I want to ask is just to get us warmed up. What was it like for you to be pregnant?

P: It is all around good, but I have some problems with my pregnancy.

I: What do you mean?

P: At the end of my pregnancy, I had high blood pressure. I also had PUPPP rash all over me, and had to deal with it for a bit. Other than that, it was good.

I: At the end there, things got complicated. Ok, what did you like about being pregnant?

P: Honestly all of it. All. I didn't really have a problem with that. I loved it.

I: Did you have a favorite thing?

P: No.

I: What did you think about how your body changed?

P: Big time. Basically, it changed I feel and how I am. I gained a lot of weight and my clothes didn’t fit me. My identity changed and I don’t think people who didn’t go through it can understand it.

I: Mhm, yea, people don't really talk about that very much, and how that probably can be really hard.

P: Yes. It’s harder than what everyone explained to me.

I: Yea? Was it just like the gaining weight was hard, or like how your body looked was hard to you?

P: How my body was changed maybe.

I: Okay well, so throughout your whole pregnancy, I want to ask about some of your healthcare experiences-- that's your early Ob care, all the way up until when you needed special care for your complications, and towards the end of your pregnancy, and Labor and delivery, so everything. What were your experiences like with the healthcare system?

P: Honestly, they were great about everything. They were never rude.

I: That's great. Did you have the same doctor throughout or were you seeing different providers?

P: For this pregnancy, I saw different ones.

I: Ok. Did you have any experiences that you would describe as negative?

P: No

I: Ok. So, I want to talk a little bit about your sexual orientation and I’m wondering if any healthcare providers during your pregnancy talk to you about that.

P: No. they never asked me about it

I: Is that something that you would like them to bring up with you or ask you about? How do you feel about that?

P: Honestly, it wouldn't bother me if they brought it up or asked me and wanted to talk about it.

I: How do you think that they could do that best … what's a good way to ask that for people?

P: Just ask me what my sexuality is, you know?

I: Ok. Do you think the Environment was friendly to LGBTQ people?

P: Yes, very.

I: Can you tell me more about that?

P: They were pretty flexible about it. My doctor, he didn’t care about my sexuality at all. He asked me about my sexuality like on personal identifying information, so I figured maybe you know people like to talk about it, but they first read about it from paper work. But they are interested in it, and treated me like I was an actual person.

I: Yea, that’s so great to hear. I’m glad you had a good experience. How did they make you feel comfortable? What did they say to you to make you feel comfortable talking about gender and sexuality?

P: They gave me a choice to talk about it. If I feel comfortable talking about it I can but I don’t have to, and I didn't mind for me to talk about it or anything.

I: Okay, so giving you that choice was nice. And what do you think would make you feel uncomfortable? What are things that healthcare providers should not do for people that are LGBT Q plus?

P: Give them an attitude, telling them “This is what sexuality you are.” Respecting them, don’t be patients their own values.

I: Yeah, not judging them, it sounds like. Ok, so just in general, did anybody in the healthcare world give you any resources that were helpful to you during your pregnancy?

P: They gave me, yes, a lot of resources for help. Like if I was needing diapers or anything. Or like stuff to look over if I had questions if I was in over my head.

I: That sounds helpful. Did you use any of them or was it just like, it was good to that they offer that stuff to you?

P: I did not use any of them, but it was great for if I really needed help.

I: Yes. Do they offer anything that was specific for pregnant LGBT Q plus people?

P: Yeah actually. There was my first time seeing an OB and they gave me a paper about it, and I wanted to go but I couldn’t go.

I: That's awesome was it like a support group?

P: It was like a small get together type thing or something like that, yes.

I: Did you feel like you were interested in that?

P: I was, but I never went because I have a pretty shy personality and also I was pregnant.

I: Yea, I get that and I feel like, if I were pregnant, it would probably just make me want to stay in more. Okay, so in general, what was it like to be pregnant and identify as bisexual?

P: Honestly. It didn’t really change anything, it’s about the same.

I: Ok, do you think that being a, this is a tricky question… do you think that being pregnant had any effect on your sexual identity? And also, same question but the reverse.

P: Yeah, the question makes sense, but, no, honestly, I did not think it had any effect.

I: Okay, perfect. Thank you. So, before we move on to the marijuana tobacco questions, I wanted to ask if there was anything else you wanted to share regarding your sexual orientation and being pregnant.

P: No, not that I can think of. And I was saying that the respect I got from people from before I got pregnant, it never really changed when I got pregnant.

I: Ok, thank you, that’s important. That’s exactly the kind of information that we need to hear. Now, we're going to move along into these marijuana questions. So, I like to remind people at this time that anything you share with us, is totally confidential so we're not going to report it to anyone; that includes family, health care providers, law enforcement-- we don't share this with anyone. So, I just like to remind people, and hopefully help you feel a little bit more comfortable because some of these questions can be kind of sensitive-- I get that. So first, can you just tell me about your first time using marijuana.

P: I was with a friend and they were using, and I was like, “Okay. Now let me try this.” And it all started from then on.

I: Were they something like a bowl or was it a bomb? How did you get high that first time?

P: It was a bowl.

I: Okay, did you like it, how it made you feel that the first time you used it?

P: Honestly, I didn't really feel any different… it just helps a lot with my anxiety and my depression.

I: Okay. That is a good segue into the next question, which is: what role does marijuana play in your life now?

P: It still helps a lot with my anxiety. The medications my therapist gave me… they didn’t really work. So, that's the only reason why I still do it.

I: Mhm. Do you have a preferred way? Do you like bowls or joints? What's your favorite method?

P: Joints.

I: Ok. When you talk about it helping with your anxiety, could you tell me about what that's like? Do you smoke when you're anxious, or do you smoke every day and that helps you not get anxious? I just want to know what it does for you.

P: Before I started smoking, my anxiety makes it so that I couldn’t go into the crowd, I would freak out. And now because of marijuana I’ve been able to go out and hang out in the crowd, it really calms me down. And I'm able to go around and talk to people now and everything because of it.

I: Okay, so is that something that you do every day? Smoking?

P: I hardly smoke before going out or anything like that. So I only smoke if I know later I will be stressed or if I’m stressed.

I: Is there anything that you don't like about using marijuana?

P: No.

I: Okay, when do you find yourself smoking a joint? What's your pattern like? Do you smoke a joint in the morning and the evening, or maybe once a month? What’s your routine?

P: In the morning.

I: Is there any time that you feel like you've craved marijuana? Like craving to be high?

P: No, I can stop anytime and be okay with it.

I: Great. That's a perfect transition because we want to know if you have tried to quit or quit smoking before.

P: Yes, I have actually, I haven’t smoked for almost two years.

I: Okay, so what was that like?

P: Honestly, not really any different except for my anxiety, but I was starting to able to handle it better.

I: Okay, did you like stop cold Turkey or did you slowly phase out?

P: I stopped cold turkey.

I: I wanted to ask if you used marijuana while you were pregnant this time?

P: Yes, I did.

I: And when you smoked, was it just a normal joint, or did you use different things because you were pregnant?

P: It was a normal joint.

I: How often do you think you smoked throughout your pregnancy? Like, once or twice within the month? Like the same amount you were smoking, or did you cut back?

P: I cut back.

I: What helped you? Was there anything that helps you cut back, like any support or coping mechanism or something like that?

P: No, it was just knowing that I was pregnant… it was everything that I needed to slow down or pull it together.

I: Ok. Did your doctor talk to you about that at all?

P: Yes, she told me not to quit all at once, just slowly because otherwise it could hurt the baby.

I: Ok! And, have you noticed during your pregnancy if there are specific things in your life, such as stressful events or trauma or whatever that may be, that may encourage you to use marijuana, or that made you want to smoke?

P: Honestly no.

I: Okay, and do you think being bisexual has anything to do with your marijuana use? Do you think those are related at all?

P: No, they are not.

I: Ok. So we really want to know, like the whole part of the study, basically, is to better understand marijuana use during pregnancy. So what do you think is important for us researchers to know?

P: It helps a lot when you know you sometimes need to use marijuana to feel better. I wasn’t eating a lot or anything, and I needed something to help with my appetite, I knew I needed to smoke. Smoking helped me get a little get something to eat.

I: Okay, so, there is like physical things that it helped you with.

P: Smoked and ate. Yes, it did help me eat.

I: OK, so it helps with your appetite. Did you use it for any other physical things, like sleeping or stuff like that?

P: No.

I: Okay, so really it was just the appetite; any nausea?

P: Yes, first trimester I had horrible nausea.

I: Did marijuana help you with the nausea? Did you use it for helping the nausea go away?

P: No, it didn’t help with the nausea. I just chewed gum.

I: Interesting ok. And, did you doctor tell you to do that?

P: No, it’s a trick I learned from friend.

I: Yea, because I've heard that for airplanes, but I hadn't heard that for pregnancy, so I was wondering how you heard about that it does work for pregnancy nausea, so yeah. OK, so the next group of questions are really similar so I’m sorry if it's like a little bit repetitive, but they're about tobacco. Do you smoke, do you vape? Do you smoke cigars, or cigarettes?

P: Cigarettes.

I: Ok. Can you talk about the first time you smoked a cigarette?

P: I was 15 year old-ish, and I was hanging out with my friends and saw someone smoking and it looked cool, so I went to them and got a cigarette, and I’ve just been smoking.

I: Did you like how it felt the first time?

P: I mean, not really, but it did help relieve my stress.

I: Okay, yeah, they definitely are a coping mechanism, they definitely soothe people. So, have you tried other kinds of tobacco, like baked or anything like that?

P: I smoked and I’ve baked.

I: What are you smoking right now?

P: Cigarettes.

I: Okay. And what do you like about smoking cigarettes?

P: It also helps with the stress, like a lot.

I: Ok, and what do you dislike about cigarettes?

P: The smell. It’s impossible to get the smell off of your clothes, your hair, your car. Everything.

I: Yes, it sticks. And I feel like it sticks around longer in the winter too. So, when do you smoke usually?

P: First thing when I wake up.

I: And are there times when you feel like you crave a cigarette?

P: Yes.

I: When you were pregnant, what did your tobacco use look like?

P: One cigarette a day, or two a day.

I: Okay, during pregnancy, is that something you still did first thing in the morning, or at night? When would you smoke when you were pregnant?

P: I was never able to smoke in the morning because of my morning sickness.

I: I see. And were there certain things that made you want to smoke during pregnancy? Any craving triggers?

P: No.

I: No, okay. Was there anything that made you want to stop smoking during pregnancy? You kind of already mentioned it in the morning that your morning sickness was pretty bad.

P: Yea, when I had morning sickness, even if I smelled cigarettes I would throw up

I: Were there things that encouraged you to smoke?

P: No

I: Are there a lot of people in your life that smoke around you?

P: My family.

I: Okay, do you think that affects how you use cigarettes?

P: No

I: Okay, were there any other things that discourage you from smoking, while you were pregnant?

P: No

I: Okay, have you ever tried to quit?

P: I have. I quit for a while and then I saw someone smoking, so then I reverted.

I: Okay, how long did you stop for?

P: Two months.

I: So do you think that cigarette use and identifying as bisexual have anything to do with each other?

P: Not at all, no.

I: OK. So this is the same question that I asked last time: is there anything else that you want us to know about tobacco use during pregnancy.

P: No.

I: And okay, tell me about the timing where you used marijuana and tobacco: Did you usually use tobacco and marijuana at the same time or did you use them at different times?

P: At the same time.

P: [baby cooing/crying]

I: Aww, someone has very healthy vocal cords, huh!

P: Oh yeah.

I: Did you have a boy or girl?

P: Girl.

I: She sounds like she's like telling you she's starving. How old is she?

P: She’s three weeks old.

I: Man, have you been sleeping at all?

P: Couple hours here and there.

I: Just a little bit every night. And… What makes you want to use marijuana and tobacco together? What was it like for you?

P: Um honestly, I don’t really know how to explain it.

I: Do you usually smoke a joint first and then a cigarette, or do you smoke the cigarette first and then a joint, like what's your routine?

P: A joint first, and the cigarette.

I: okay and has it always been like that?

P: Yes.

I: Okay, why do you think that is, because that's so interesting!

P: Honestly, I have no idea. After I smoke, I feel, “Oh maybe… I want a cigarette,” so I go grab a lot of cigarettes.

I: Ok. Do you think that smoking a joint makes you want the cigarette or is it not related but you just do them at the same time?

P: No, not really related.

I: Ok. If you had to choose between cigarettes or marijuana, what would you choose?

P: I wouldn’t do cigarettes.

I: Okay, so you would keep the marijuana and get rid of the cigarettes. Do you prefer how one makes you feel more than the other?

P: Yea, cigarettes stinks a lot more.

I: OK, so, I want you to imagine being pregnant and up in whatever you think of as a perfect world, so these are perfect world questions. In a perfect world, what do you wish all LGBT Q plus women knew about pregnancy?

P: Just like how people react.

I: What do you mean?

P: Just like when people explain pregnancy to first time moms, we are not all like that. We're different people, and we will have different experiences.

I: Okay that's so interesting. Did you feel like what were you told by others did not end up being true for you?

P: Yes. Everyone said it will be really, really, really rough. But honestly I didn't think anything of it was really that rough for me, personally. People told me about their birth experiences and saying that it was so bad, but mine went good and really went the way I wanted to.

I: Okay, so you had a more positive experience, and people were kind of scaring you beforehand. That's so cool that you want people to know that, like pregnancy can be good, and like not to be scared by all the stuff. Okay, next question, so in the perfect world, what do you wish all health care providers knew about pregnant LGBT Q plus women?

P: Honestly, nothing for me because they already know everything.

I: In the perfect world, what do you wish all LGBT Q plus women knew about marijuana?

P: I don’t even know how to answer that.

I: Yeah it's a tricky question to answer.

P: Honestly I don’t even know.

I: What do you think about tobacco use? Do you have any idea what you wish they knew about tobacco use?

P: Yes, I wish they knew that it is not really cool like other people are saying is.

I: Finally, I want to get your feedback about what I could do better, or what questions you think I should be asking that I'm not asking.

P: Honestly, no, not really any more questions. It’s a lot of questions.

I: Yes, a lot of questions… it's a long interview for that, I think.

P: Kind of.

I: Well, if you think of any like feedback that you want to give me, you can always email me or text me. So now I'm going to put the $50 on your card, right now, do you have any questions about how to use it.

P: No.

I: Okay Thank you so much for doing this, I really appreciate all your responses and all the time you gave. Have a good rest of your day.
