# Supplementary material for: Experiences of postpartum mental health sequelae among black and biracial women during the COVID-19 pandemic
Source: BMC Pregnancy Childbirth. 2023 Sep 4;23:636. doi: 10.1186/s12884-023-05929-3 (PMC10478375; doi:10.1186/s12884-023-05929-3)
Supplement: Supplementary file 24 — Supplementary Material 24 [file 12884_2023_5929_MOESM24_ESM.docx]

**Supplemental File 1.5 Interview Transcript with Participant 5300**

I: Okay, so just [to] get started. How is everything with your pregnancy going so far?

P: (very poor audio) [I have been sick]

I: Sorry I'm having a little bit of trouble hearing you. Is there like a microphone or anything that you might be able to speak into directly?

P: Can you hear me better now?

I: Yes, a little bit better, a little bit better. Thank you so much. Can you repeat what you said about how your pregnancy is going?

P: Can you hear me a little bit better?

I: Yes, that's much better. Thank you so much.

P: Okay. Yeah, so, everything's okay. I just have been feeling sick but I've been cramping.

I: Okay- you haven't been- I'm sorry to hear you haven't been feeling well. Overall, do you feel like your doctors are, you know, being supportive of you when you're not feeling well?

P: Yes.

I: And other than not feeling well how has everything else been going with your pregnancy?

P: Pretty well.

I: What are some other more positive experiences that you've had, since it started?

P: My family has been supportive, I didn't think they will be as supportive, because in my family, most of them got pregnant at an early age so I figured that they will probably be upset that I'm 20 and having a baby but they are supportive.

I: Okay I’m glad to hear that, and how has that made you feel with that kind of support?

P: It makes me feel a little better, because I felt like I wouldn't have the support that I needed.

I: Yeah. Yeah, absolutely. It's very important. What kind of things have they done to sort of make you feel supported?

P: My cousin to be exact- She said that she wanted to do my gender reveal. And she told me if I needed anything to let her know, so if that made me feel better.

I: Oh that's so great. So, like I said that this interview overall is going to be sort of about, you know your thoughts and feelings about marijuana and tobacco and alcohol. So when it comes to marijuana and tobacco, smoking, vaping, other forms of use- What overall are your thoughts about marijuana and tobacco?

P: Alcohol I really don't like because I feel like it can go to a different level. I can say that I did do it when I was a little bit younger but I did stop. Marijuana… I do like smoking marijuana because it eases me when I have hard times, but since I've been pregnant, I stopped. Yeah but I do think that like during pregnancy- it shouldn't be used at all, because it's a risk for miscarriage, or anything in general.

I: And can you tell me a little bit more about that you said you know the risk of miscarriage. What sort of other- What other risks are you aware of?

P: I'm aware that maybe something could be wrong with the baby's development. And I also know that may be like if you continue to do that while you're pregnant- That, like your baby will have difficulties, when the baby's born.

I: Sorry I'm just taking some notes here. How about with tobacco? What are your thoughts on that?

P: I feel the same thing is, alcohol, if you just use it I think it will affect the baby's development, and then the baby might have problems breathing maybe because just like smoking like if you smoke your whole pregnancy, the baby will have breathing problems, just things like that.

I: And do you feel that one marijuana versus tobacco: Do you feel that one is safer than the other?

P: I think they’re both the same.

I: How about the different ways that you can use marijuana and tobacco? Do you feel that there are ways to use that are safer than others?

P: I feel like marijuana is a little bit safer than tobacco- because tobacco can get you cancer and other things like that but marijuana most people just use it because they just like to feel mellow. Like when I'm going through hard times, things like that.

I: How about something like if you were to vape versus smoking, which, how do you feel about the safety- (cut off by P)?

P: I think it's the same thing as tobacco- vaping cuz the same thing. It's the same level as tobacco.

I: Do you feel like this is something that you're able to talk to your doctor about, if you had questions during your OB visits about marijuana or tobacco?

P: Yes.

I: And why would you feel comfortable talking to your doctor specifically? Is there anything that your doctor does to make you feel comfortable?

P: No. It’s just, like, I felt like if you just be straight up and honest about how you feel about it then they can give you the benefit of the doubt.

I: And has your doctor talked to you specifically about marijuana and tobacco use during pregnancy?

P: Yes.

I: What are some other resources or other places that young women could get information on things like the safety of marijuana or tobacco during pregnancy?

P: I know that they probably have websites that you can go on. I also know the little booklet that the doctors gave to me when I went to my appointment that has like all the information about marijuana and things like that, what to do, what not to do. I was just reading it today.

I: And have you found any websites that you feel are good sources of information?

P: Not yet, I've just been looking at the booklets that my doctors gave to me.

I: And the booklet that they give you, are there additional resources within those or is it just the information presented in the pamphlet?

P: It’s the information but they also gave me a paper that has like all this like if you need somewhere to stay they have a whole bunch of housing, if you have depression at the end after the baby, they have resources on there. They just have different sources on that paper that you can look at.

I: That's great. So, since the start of the pandemic, how have things, you know overall changed for you? I know that you had said that you were using marijuana before your pregnancy and now are not. Has the pandemic impacted that in any way?

P: No. The only thing that the pandemic impact[ed] was just we're having to wear masks being safe around people. Just being cautious of a lot of things ever since covid went up.

I: Has there been has- You mentioned increased safety precautions, can you tell me a little bit more about that when it comes to things like your doctor's visits for your pregnancy?

P: Yes. Like when I have to get checked in for doctors, I try to stay away from people because the doctor said that it's not safe for me and the baby especially since I'm pregnant now, so I just try to stay away from people now that I'm pregnant.

I: And how has that made you feel overall? Do you feel any sort of way about that?

P: No, I actually feel okay because I’m the type of person that likes to be by themselves anyway.

I: Right, so, this next section of questions is going to talk a little bit about sort of like your own personal experiences with marijuana and tobacco I know that we touched on it a little bit but just as a reminder, you know these, you know, if some of the questions or personal, you don't feel comfortable answering you don't have to answer any question. So, just start off… So what have your own experiences with marijuana and tobacco been?

P: For tobacco I don't smoke. I never smoke tobacco. I know a lot of my family members that smoke and I hate the smell and I just think that they do it like my dad. I want him to quit but he will never quit so I just try to stay away from it.

Marijuana, I have been smoking marijuana since I was about 18 and around that time, I was going through some dark times. I was going through some things, and I just felt like maybe just smoking marijuana would just ease the pain, which really helped, but then like once it's over, you go back to reality so…

I: Can you walk me through that a little bit more like how you were feeling and things like that?

P: Yeah, so, at the time I was living with my grandmom, and she has a boyfriend that was doing things to me that I felt uncomfortable, and she didn't believe me, so I moved in with my ex boyfriend at the time and his family. So that really changed me and their environment like they've smoked marijuana like every day and stuff like that, so it kind of like drag me into the point where I started doing that just to ease the pain.

I: And you said that was when you were 18?

P: Yes.

I: And then when you were using marijuana regularly, did you have a method that you preferred like smoking blunts, bowls…?

P: I smoked mostly blunts and joints. That's pretty much it. I didn't do all the extra stuff like bongs and all that, but I only smoke maybe like two times a day.

I: So that past experience that you had using marijuana, did that influence your decision on whether or not you use them when you found out you're pregnant?

P: No. The moment I found out I was pregnant. I was smoking marijuana but like I stopped immediately. I didn't even know I was pregnant until I took a test. I was like feeling sick but I wasn't throwing up, my boobs were feeling tender so I was like okay something's wrong I don't feel like myself, but I wasn't really thinking of it, until one day I told myself just take a test, so once I took the test and then I found out I was pregnant and I stopped immediately.

I: Can you tell me a little bit more about that? I know you said that since you've been pregnant you got some of that information from your doctor's office. What sort of information did you have about marijuana before you were pregnant that sort of led you to stop once you found out you're pregnant?

P: The doctor told me that the baby can have difficulties when coming or I might have difficulties, even while being pregnant because of me smoking marijuana, and it's not a good idea to do it.

I: And was this at- sort of like your, you know, first doctor’s appointment that you had, or was this before you were pregnant that you've got this information?

P: This was my first appointment.

I: And how did you feel about that when they told you that?

P: I felt okay, I'm not really like marijuana like I have to smoke. I have to smoke, like, it's like, I only smoke like two times a day so I really wasn't like, really, craving it too much so I was okay with it. Like today I'm okay. I don't feel like I need to smoke or anything.

I: When you were smoking with, would you usually do that alone or do you usually do that with someone else?

P: Sometimes I'll do it alone. Sometimes I do it with my boyfriend.

I: And how, how did it make you feel, overall?

P: It made me feel calm and happy because I wasn't really thinking about the pain that I went through, like, today I still suffer from that pain because my grandma still went home even though I told her everything that happens. So to me it just feels like she doesn't care.

I: Yeah, that sounds really hard. I'm so sorry that you have to go through that. How about afterwards when you would be done smoking and sort of, you know after (cut off by P)?

P: Afterwards I feel the same. I told myself that I wanted to breastfeed so I know I'm not going to be smoking until maybe an extra year or two, because I don't want the weed in my system and then I have to feed the baby.

I: And how do you feel about sort of that coming to that decision and looking you know looking towards once your baby's here?

P: I'm okay with it- anything to make sure that my baby’s okay.

I: Yeah, that's great. When you said that when you first found out you're pregnant you stopped using, did you have any periods of time where you did use when you're pregnant or did you basically quit cold turkey?

P: Yeah I quit the whole thing once I found out.

I: You said that you know it's easier for you to talk to your doctor about this kind of stuff. Do you feel pretty- do you feel it's easy to talk to them about pretty much anything, or is it this specific thing you feel very comfortable with them?

P: I can talk to them about anything that makes me feel comfortable talking to them about. Like, how I was comfortable enough to tell you my situation, or my grandma. I'm comfortable with telling people that because it's good to get feedback from somebody.

I: Yeah, absolutely. And was there anything you know specifically, thinking about talking to your doctor about marijuana, was there anything specific that influenced that decision or was it just something that you went in knowing you would be open, talking to them about that?

P: It was just something that I was open to talk about, and once my doctor talked to me I felt a little bit better, and happy that I did stop.

I: Tell me more about those feelings. What made you feel happy about it?

P: It made me feel happy because I know some moms that are pregnant and they still smoke and still do whatever they do, and like they end up saying like something’s wrong with the baby or stuff like that and I don't want to go do stuff like that so I'm just happy and grateful that I stopped when I did.

I: Do you feel that there's any difference? So talking to someone like me who's a researcher and I'm, you know, kind of getting this information just for the purposes of the research study versus a health care provider, do you feel like there's a difference talking to someone like me or talking to someone like that as far as disclosing information about marijuana or tobacco use?

P: No, I feel like if you feel comfortable talking to somebody about marijuana tobacco. I mean, you feel comfortable saying it. I don't have a problem with telling people because it's best that you just be honest and just let them know, instead of just you know being scared about it.

I: That's great. When you were at your appointment, did your doctor basically bring any of this up or was it sort of you asking them questions?

P: They most likely brought it up. Just tell me what I can do and what I can't do.

I: So, you know, for other women who are your age and sort of seeking this information out- outside of their doctors were- what sort of, how do you think would be the most helpful to get them information if they had questions about marijuana and tobacco?

P: To talk to another pregnant person because nobody else will understand, unless the person is in your shoes.

I: And do you feel like you have other pregnant women that you can talk to about any questions like this?

P: Yes, there's a group on Facebook, called young moms that I joined, and they be answering questions, asking questions, they give out a lot of information so if I realized I needed something from them like they're supportive.

I: That's great. And how, how did you- can you tell me a little bit more about the group, how did you find them?

P: I was looking through Facebook one day, it was weird because I didn't even know I was pregnant yet and I'll just say, Oh, this looks like a group I might want to just add, just in case. Whenever the time comes, so then when I found out I was pregnant I was like, well, this is useful.

I: What kind of topics or what kind of information do you guys share in that group?

P: They post a lot of things like what is really like during birth, what is like after the baby's born like today I found out that you have to clean the baby's mouth at like (unknown) a month, I don't even know that that's something I just found out today, so they just be saying like helpful things for other moms and expecting moms that's helpful.

I: Yeah, that's great. So you mentioned, you know, what was there any other like bit of information that they helped share with you that you were like oh glad I knew that before my baby came?

P: Oh yeah, they told me about the rips like there's second degree rips third degree rips- where it's like while you're pushing this stuff like that I don't know about that so I was kind of like, I'm a little scared, but I'm glad that I know about it instead of going in and being surprised.

I: Yeah, having all of the information… Do any in that group do, does anyone ever ask questions about, like, maybe using marijuana or tobacco during pregnancy?

P: No.

I: Do you think that that would be a safe space for someone if they had a question about that?

P: Yes, they pretty much ask about anything. And all of them, mostly answers too. If any of them have a question or anything about their baby, or if they're pregnant like some people will be saying like they are spotting: Is this normal? Things like that and they'll come they'll be like, ‘well make sure you call your doctor to double check’, things like that so…

I: That's great- that sounds like they provide quite a lot of support, which is very important… Is there so within your health care providers like your OB, doctor- Is there anything that a doctor can do to help a young woman, you know, feel comfortable like you do, you know, sort of asking the questions and getting the information? Like is there anything that the doctor themselves could do to sort of make that a safe and open space for their patient?

P: Just let them know that it's okay that they can talk to them about anything because some parents or some expecting parents don't really feel comfortable enough talking about things because they have never been in a life- [what am I trying to say?] They never been the type to come out and just stay how they feel. They always keep stuff in, Maybe because of their lifestyle or how they live and stuff like that.

I: Yeah. Do you think that or what are some other reasons that people might not feel comfortable talking to their health care providers?

P: They might feel like they're being judged. Like, if they're doctors, like you to look or it could be like anything you can like give them a look or shrug shoulder or something like that and then they might just feel like you're judging them so they like ‘okay I'm not going to ask this next question because I feel like they're going to judge me’.

I: Yeah, absolutely. So maybe you know, is there anything with the doctors like body language that they could do to sort of, you know, What sort of things could they do to help… basically the patients not feel- not feel judged in those scenarios?

P: Especially the young moms like me because I know that very young moms are like 15, 16, 17 I know they would come in and be looking at them like ‘you're young’ and stuff like that. Like they already get it from their families and stuff like that all they just want is your support.

I: Yeah, absolutely. And is that something- Have you ever, you know, been in a situation where you felt like that?

P: I have plenty of times like my dad, he's not really too happy about my pregnancy. He's just been like, I'm too young and things like that but I'm just like telling him like at lease on that 16 or 15 am the baby like I waited until I was 20, but it was even planned just something that happened so…

I: Yeah, and how do, you know, how does that make you feel overall? If you know someone says something like that to you?

P: It makes me frustrated because it's like I'm not young. I'm 20 years old. I've been an adult since I was 18 so I just feel like you should be supportive of me and not try to come at me because I'm pregnant.

I: Yeah. And you said that, you know, as you've gone along in your pregnancy- You know, there have been people in your family that have made you feel supported, you mentioned the gender reveal party- Was there anything else that anyone your family said or did for you that made you feel supported in your pregnancy?

P: My granddad, I thought my granddad was going to be mad at me when I told him I was pregnant but he told me that he would never be mad at me. He told me to… not regret what I did, because I was thinking about getting rid of the baby at first. I was like, I don't know if I'm ready for this and things like that.

And my granddad would be mad at me but then he was just like, ‘Don't do it, you'll regret it’, stuff like that- ‘I still love you, I'm happy for you’. He just made me feel more happy and he was just like I gotta tell my mom I got to tell your aunt and stuff like that so.

I: And after you talked to him, did that change any way you felt about your own pregnancy?

P: Yes.

I: How did you feel sort of like before that conversation and then after that conversation?

P: Before I was kind of like doubting…like ‘is this really what I want to go through’, ‘am I sure I’m ready to be a mom?’, things like that; but then after I talked to him I was just like, ‘yeah, I'm going to be a mom, I'm going to be a great mom, I’m going to make sure my baby’s okay.’

I: That’s very sweet. So, as we- you know, sort of wrap up the interview… How do you know- how do you feel sort of going forward? It sounds like you're very, you know prepared to go forward in your pregnancy. How are you feeling about, you know, the next, you know, several months?

P: I am prepared for anything. I'm trying to get myself together. I'm trying to get my own house for me and my baby because the one I stay at now is not enough space for us. So I'm just trying to get that together. Try to get a car, ‘cause I do take the bus. So once my baby comes I will have all that.

I: Anything else that you're doing to prepare?

P: I'm just trying to prepare for the negativity because I know it's going to come soon. Like, coming forward with my grandma like if she brings her boyfriend to like my gender reveal or my baby shower even like I don't know how I'm going to like feel about that because like everything that happened, and I'm still not even on good terms with my grandma, [we don’t] speak, so I don't know how it's going to go.

I: Yeah. Do you have any support to help you through all of that?

P: Yes.

I: What sort of support do you have in place for yourself?

P: My boyfriend- he checks on me all the time, he tells me not to stress about it. He said that I need to let the past go but it's kind of hard to let the past go, especially like, that's my grandma.

I: Yeah, absolutely. Any other support within the community?

P: The doctors at Magee hospital are very supportive. They all were like ‘Congratulations, Congratulations we're happy for you’. They made me feel happy, because like I said at first I wasn't really too sure about the pregnancy, like, kind of scared, because I didn't know what to expect, or if I'll be able to do it.

I: And it sounds like they are, you know, helping you feel very, you know, positive and well prepared.

P: Yes.

I: That's great. Alright so… [P’s name] we're about, you know, wrapped up. You know I really enjoyed talking to you. Thank you so much for being so forthcoming about yourself. I really appreciate just getting to have this conversation with you and I know the whole team and the research team appreciates your participation in our study. Before we end the interview, is there anything else you'd like to add that we didn't discuss today?

P: No ma’am I think that's pretty much it.

I: Great, and just want to check you still have your payment card from when you did the survey?

P: Yes ma’am.

I: Okay then after we hang up I'm going to load your payment onto that card, it'll be $20. And we do have, you know, as you go along your pregnancy- These types of interviews you know we can do, we have three more that you can possibly do so will reach out to you at those time points. But if you have any questions or if anything comes up, just feel free to give us a call. And again, thank you so much for your time today.

P: Thank you so much. I appreciate it.

I: Yeah. All right, well you have a great, great rest of your day.

P: You too, thank you.
