# Supplementary material for: Experiences of postpartum mental health sequelae among black and biracial women during the COVID-19 pandemic
Source: BMC Pregnancy Childbirth. 2023 Sep 4;23:636. doi: 10.1186/s12884-023-05929-3 (PMC10478375; doi:10.1186/s12884-023-05929-3)
Supplement: Supplementary file 26 — Supplementary Material 26 [file 12884_2023_5929_MOESM26_ESM.docx]

**Supplemental File 1.25 Interview Transcript with Participant 5311**

I: um how what's your pregnancy, going so far.

P: So far, so good. Good but says like it's some stomach pains here and there, but don't feel good other than that.

I: Is it going like as you expected or different than you expected.

P: Is going a lot different than what I expected to be honest. I thought I was going to be the same as how my daughter was because I was thrown up all the time, was able to eat or drink or do anything but this have surprisingly, I could do eat drink a whole bunch other stuff I was able to do before. So.

I: mentally how how's it been going.

P: it's been a lot better than what it was previous pregnancy.

I: All right, well let's go ahead and jump right into. Some of the interview questions so i'm wondering first like what are your thoughts about where you started.

P: I mean level like I know there's people that smoke when they're pregnant and they stop eventually for like it's not the smartest thing, because then that could cause damage to the baby, so I feel like people should do it.

I: What are some of them like good things you've heard related to marijuana in pregnancy like why people do it.

P: I just heard, like people could have like stillborn baby face about birth defects, I never hear anything good from it.says that help with people's like stress it is that he knows about it but you're not doing anything good come out for the baby.

More about some of the effects you've heard of using now one thing.

I heard that maybe could come out stillborn the baby could have birth.To baby i've heard that not even just with marijuana her with any type of JC is a babies, they could come out like.

They get addicted to come out like screaming and crying because they went, that it will take forever for the baby to like. get off of that stuff so don't have to worry about it, like does that make sense, like, I heard that when I started with my daughter I overheard this girl she has she has taken a whole bunch of these pills have to second her baby their sister whenever how she was taking these pills, and how do I have a birth defect and.

That is like I was thinking to myself, like this crazy that people really does do out as a babysitter come out addicted stillborn birth defects, or they die and Sergey and that's that's just crazy to me.

I: Oh sorry go ahead.

P electrolyte people should put more consideration to the failure, have a human being inside them when they can't make decisions for themselves, because very sad you're trying to process and grow in form it's a human and do what they need to but. that's that's all I really know.

I: Where have you heard those things.

P: um i've heard things from like other studies that i've done i've heard it or wine i've heard it from my mom i've heard it from my grandma i've heard it from a whole bunch of people around me that the baby to have birth defects stillborn i'm one of my baby days sisters um.Pregnant at all, and she had like she has really bad is it so she was smoking marijuana here and there, so she didn't notice, she was pregnant it so it sounded like give birth to the baby so she was real quick used to stillborn. So that's where I person, what is the from.

I: His marijuana something you've ever tried.

P: In between my daughter's pregnant like when I started my daughter, and this pregnancy, like in the middle man Daddy was real high up.So I was smoke like hearing their elders, but a lot of sense, but I was smoking in there, and there was never able to like marijuana it was just like a medical weeping which is like I think thc.

I: mm hmm.

P: That was about it, I didn't actually touch waiter that's it like that.

I: Tell me about that, like, how can, how can it happen, thank you.

P: Basically, whenever you get like take thc or something like that I feel like it just like it just takes you away from every stressful thing that's going on in your life. Like for me it really like a lot of stress like I wasn't thinking about it as like well was more laid back and calm and I was actually able to good throughout my day and doing I needed to and I sit there and just thought about was happy.

I: Have you so you've stopped doing that, since you found out you're pregnant.

P: God, because I, I was, I found out, I was pregnant like three or four weeks into the pregnancy like I knew like right off the BAT that like I didn't have my period, and I was like this is weird and then.I thought I was pregnant and I was just like a month in so it was like I stopped way before that sounds like that was good.

I: What have you done to like cope with anxiety. Since you've quit.

P: I have a therapist I talked to now so that's how the her everything's been like more calm right and i'm able to talk to her whenever I need to it, I just got myself around the people that would cause me stressor.

57

00:06:12.180 --> 00:06:17.820

Participant: is just like meshes is going, if I have any type of stress or anything I just checked my therapist or.

58

00:06:19.170 --> 00:06:26.400

Participant: Talk to my daughter's day or Whatever the case may be, I just talked to people around me to check up to that.

59

00:06:30.030 --> 00:06:34.950

Interviewer: Why did you stop using marijuana like even before you find out you're pregnant.

60

00:06:36.030 --> 00:06:41.730

Participant: Stop because it was just so funny where it was like Hello.

61

00:06:44.430 --> 00:06:45.660

Participant: mommy told you know already.

62

00:06:47.670 --> 00:07:00.270

Participant: I stopped because after like after a certain point, like i'd be to like back in calm and then there'd be stuff around me happening in I will get paid enough attention so.

63

00:07:01.560 --> 00:07:02.760

Participant: I just I just stuck.

64

00:07:03.840 --> 00:07:04.440

Participant: around me I.

65

00:07:05.490 --> 00:07:08.250

Participant: had to pay more attention to it and it was just like.

66

00:07:09.660 --> 00:07:12.780

Participant: Just just like I said too much going on, I need to pay attention.

67

00:07:13.470 --> 00:07:20.610

Interviewer: mm hmm so, is it something, you would think about using again when you're not pregnant anymore, or do you think.

68

00:07:21.720 --> 00:07:25.050

Interviewer: it's like this therapy so much better like coping mechanisms.

69

00:07:26.640 --> 00:07:27.270

Participant: To be honest.

70

00:07:28.800 --> 00:07:34.230

Participant: I only look like in them, what I do know that that is baby cuz that's frustrating and.

71

00:07:34.680 --> 00:07:43.350

Participant: I just figure out a way to deal with the stress on its own or just start to address people myself or other people were to stress that cost me like other stuff so.

72

00:07:43.920 --> 00:07:56.850

Participant: Smoking I really don't advise anybody to smoke or less if you really have to because, for me it would work and then it just got to a point where I was too laid back in there was stuff happening in our society live.

73

00:07:59.370 --> 00:08:02.010

Interviewer: A little bit more about what you mean when you say like mid.

74

00:08:02.130 --> 00:08:02.490

To late.

75

00:08:03.870 --> 00:08:12.510

Participant: So whenever I sound laid back, I mean like i'll either send a bit all day and be on my phone just sitting there or i'll fall asleep.

76

00:08:13.200 --> 00:08:22.260

Participant: But me while I mixed with a baby was a can do it a headset for her, but it was just like other than that I wouldn't really changing the credentials lay back and.

77

00:08:23.460 --> 00:08:26.520

Participant: be calm and not have to worry about missing so.

78

00:08:27.810 --> 00:08:30.750

Participant: just be a laid back like to send them baby and.

79

00:08:32.130 --> 00:08:38.760

Participant: Then what the baby and then going back to lay back down and just do that stuff tonight to worry about listen.

80

00:08:42.150 --> 00:08:45.900

Interviewer: Like marijuana being many lies like.

81

00:08:47.250 --> 00:08:49.470

Interviewer: You think you would have used it if if you.

82

00:08:52.440 --> 00:08:53.400

Interviewer: Know okay.

83

00:08:54.420 --> 00:08:57.150

Interviewer: So what do you think about the medical legalization of marijuana.

84

00:08:57.810 --> 00:09:02.790

Participant: I feel like people are taking advantage of it like people that don't need to smoke.

85

00:09:03.240 --> 00:09:17.430

Participant: By people don't know kindness around with them, they just get a court justice smoke, or just to sell it to me that make it harder for the people that actually need like a medical record or something that actually have problems like when i'm when i'm a boy, she has.

86

00:09:18.510 --> 00:09:31.980

Participant: A she actually has a medical problem and her doctor prescribed her to get a medical week or and she eventually got it, but she doesn't do it all like that, but it's just like to the other people that do it, I just feel like they took advantage of it.

87

00:09:34.980 --> 00:09:36.240

Interviewer: monkey me Mina.

88

00:09:37.410 --> 00:09:42.480

Participant: Mina Mina I when I say people took advantage of it like they don't go go get the week auto pay for it.

89

00:09:42.870 --> 00:09:54.090

Participant: And then either those started smoking, all the way they get or they will sell it or do whatever with it and just run free and think that oh if they get caught with weed they're not going to get shot like they were before.

90

00:09:57.420 --> 00:10:00.300

Interviewer: Thinking about using tobacco during pregnancy.

91

00:10:09.120 --> 00:10:21.090

Participant: But i've heard people could get things to nicotine so it's just like oh oh either way like how we did all like people using it while you're pregnant and i'll let people use Internet to.

92

00:10:24.210 --> 00:10:27.000

Interviewer: Have you I couldn't hear she said this, have you heard.

93

00:10:28.140 --> 00:10:30.870

Interviewer: Has any effects on during pregnancy.

94

00:10:31.290 --> 00:10:33.510

Participant: I love her love, I really talk about the bed.

95

00:10:35.130 --> 00:10:36.030

Participant: Because anything.

96

00:10:37.950 --> 00:10:38.310

Participant: But.

97

00:10:42.540 --> 00:10:45.840

Interviewer: i'm tobacco using tackle something you've ever tried.

98

00:10:46.470 --> 00:10:47.340

Participant: Over I would never.

99

00:10:49.170 --> 00:10:49.920

Interviewer: Tell me more.

100

00:10:51.780 --> 00:11:04.260

Participant: I was I grew up watching my granddad's look any eventually quit, but I just don't like the smell of it, I just don't like none of it like that's That to me is just so pointless like what's the point is look at the back.

101

00:11:07.980 --> 00:11:14.880

Interviewer: um do you think take using like marijuana versus using tobacco is different at all for pregnant women like is one worse on the.

102

00:11:17.340 --> 00:11:20.070

Participant: Mobile What did you say the baby had a whole bucket of toys.

103

00:11:20.400 --> 00:11:25.470

Interviewer: Just if if marijuana versus tobacco is different for pregnant woman or if it's like just the same.

104

00:11:25.530 --> 00:11:26.040

TV.

105

00:11:27.540 --> 00:11:39.450

Participant: I feel like marijuana is worse for pregnant women because it's because we're effects on the baby that I live tobacco, I really hear nothing say it affects the baby, but so evangelizing isn't.

106

00:11:42.510 --> 00:11:45.090

Interviewer: curious if the paint on it like living to the.

107

00:11:46.290 --> 00:11:46.830

Interviewer: More.

108

00:11:47.190 --> 00:11:53.730

Interviewer: marijuana use it all like does that worse in your anxiety and lead to you getting marijuana did that impact you at all.

109

00:11:58.170 --> 00:11:58.410

Participant: My.

110

00:12:00.000 --> 00:12:00.780

Participant: toys.

111

00:12:03.240 --> 00:12:04.230

Participant: can repeat your question.

112

00:12:05.130 --> 00:12:06.600

Interviewer: I just asked us to pin damaging.

113

00:12:07.650 --> 00:12:08.970

Interviewer: Your marijuana use.

114

00:12:10.560 --> 00:12:12.870

Participant: Because I only use it and we played a pregnancy.

115

00:12:14.520 --> 00:12:15.180

Participant: dropping.

116

00:12:16.890 --> 00:12:17.490

Participant: Baby point.

117

00:12:19.980 --> 00:12:23.670

Interviewer: You tell me more about the process of you getting medical marijuana.

118

00:12:26.460 --> 00:12:41.460

Participant: One of my friends had a weeping and is hidden here and there, and she just gave it to me to use, and then I basically gave it back to her, because it was just like there's no point me to keep to use it, because the way I was just trying to lay back and everything.

119

00:12:45.210 --> 00:12:47.100

Interviewer: And, have you talk.

120

00:12:47.160 --> 00:12:48.780

Interviewer: to your doctor.

121

00:12:49.800 --> 00:12:51.900

Interviewer: or anyone about marijuana your.

122

00:12:53.580 --> 00:12:54.750

Participant: love it was that for.

123

00:12:55.410 --> 00:12:56.640

Interviewer: Have you talked to her like.

124

00:12:56.820 --> 00:13:00.030

Interviewer: The nurse or doctor at your appointments about marijuana use it all.

125

00:13:00.960 --> 00:13:04.980

Participant: And then they asked and I said before, I got pregnant, but does about it.

126

00:13:05.700 --> 00:13:06.030

Okay.

127

00:13:07.590 --> 00:13:09.870

Interviewer: And did they give you any information about it.

128

00:13:11.970 --> 00:13:14.160

Interviewer: Okay, what about tobacco, did they ask about that.

129

00:13:14.550 --> 00:13:14.820

He.

130

00:13:15.960 --> 00:13:18.090

Participant: never used that I would never touch that you.

131

00:13:19.410 --> 00:13:24.930

Interviewer: Did you have any worries going into like telling them that you have used marijuana in the past.

132

00:13:27.000 --> 00:13:30.150

Participant: I did at first, but I thought they were going to check my daughter for me.

133

00:13:30.900 --> 00:13:31.350

Participant: But.

134

00:13:31.920 --> 00:13:41.010

Participant: I mean whenever you're pregnant or light before your privacy all like that, so you still gotta be honest with them so they can actually help you with certain points in your pregnancy so.

135

00:13:41.490 --> 00:13:51.570

Participant: I just sort of that I used it like in between the pregnancy, so my daughter in this baby per day same with an agent said, as long as it was before I got pregnant, it was fun.

136

00:13:53.520 --> 00:13:59.010

Interviewer: Tell me more about like you're worried about your daughter being taken away what makes like, why did you think that.

137

00:14:00.210 --> 00:14:11.970

Participant: Oh, my actually my daughter, my friend her God or her daughter was got take it from her because she was smoking in the pregnancy and.

138

00:14:12.570 --> 00:14:19.320

Participant: She told her doctors and they said well if you keep smoking and we're going to take the corner has see let's get involved and take the baby from me.

139

00:14:20.280 --> 00:14:29.310

Participant: And like she would have stopped smoking at all like she was smoking puffballs just smoking weed she was doing everything, while she was pregnant with her daughter and.

140

00:14:30.000 --> 00:14:38.610

Participant: The baby she didn't come up with I think she has like a lazy I robot but that's the only birth defects yet other than that same really heavy lifting but.

141

00:14:39.690 --> 00:14:46.740

Participant: Since she told me that they were going to try to take her daughter, I was scheduled to take my daughter from me since I smoked in between the pregnancies.

142

00:14:48.480 --> 00:14:50.370

Interviewer: What do you think about see why.

143

00:14:52.920 --> 00:15:00.570

Participant: we're all I had to live off waste money and it's honestly annoying because there's 10 so they'll do stuff with us as well, they don't do nothing at all.

144

00:15:04.860 --> 00:15:13.350

Interviewer: And I guess i'm wondering, like, I think you told me this a little bit, but so you were worried about that, why did you decide to tell your doctor anyway.

145

00:15:14.610 --> 00:15:19.800

Participant: I love well I think it's a no, just in case if it could cause something to happen to the baby will for.

146

00:15:20.310 --> 00:15:21.870

Participant: Like all of that makes sense for like.

147

00:15:22.020 --> 00:15:31.050

Participant: You know how like it can still be in your system and it could either affect the baby, one way or work, but I just told them just so that, in case it didn't affect the baby that they wouldn't live.

148

00:15:33.990 --> 00:15:39.690

Interviewer: um, are there any other words you have other than the current or maybe taken away.

149

00:15:43.020 --> 00:15:51.390

Participant: Just the fact that this pregnancy, I was scared that it was gonna be a lot more difficult, but does about it, but it doesn't have nothing to do with like weight or anything.

150

00:16:09.810 --> 00:16:10.800

Interviewer: Did you feel like.

151

00:16:11.850 --> 00:16:13.170

Interviewer: In the moment to talk about it.

152

00:16:15.570 --> 00:16:27.240

Interviewer: Like was it obviously had those words before like in the moment, did you feel like it was hard or did they help you like can feel comfortable and actual conversation like where your worries bigger than the situation up.

153

00:16:28.830 --> 00:16:30.390

Participant: Everything so comfortable for me.

154

00:16:30.900 --> 00:16:32.700

Participant: No matter what the situation was.

155

00:16:33.630 --> 00:16:37.200

Participant: Even with like home problems that I had that I was able to talk to them about.

156

00:16:38.490 --> 00:16:39.480

Participant: Like when David asked.

157

00:16:40.530 --> 00:16:54.600

Participant: The pregnancy that could cause anything they were always like making me able to feel comfortable soccer soccer but everything so that's why with this previous data it really is it safe to some that I use a week before.

158

00:16:55.650 --> 00:16:57.060

Interviewer: You have the same doctor.

159

00:16:58.290 --> 00:17:00.360

Participant: yeah I see all the same, midwives.

160

00:17:00.630 --> 00:17:00.900

Okay.

161

00:17:03.120 --> 00:17:14.520

Participant: There are like there's like three or four of them, we see and like I seen them when I was trying to look for so they already know my situation I said I think so that's why I was like easier for me to actually be able to talk to them.

162

00:17:15.510 --> 00:17:19.320

Interviewer: How do they help you like feel comfortable like what kinds of things have they done.

163

00:17:23.310 --> 00:17:25.920

Participant: Just but just by listening like.

164

00:17:26.940 --> 00:17:37.080

Participant: And just by saying why to help me with any way they can and just say what was that they were say even am I supposed to say stuff like that, but.

165

00:17:40.020 --> 00:17:40.620

Participant: Primarily.

166

00:17:44.520 --> 00:17:52.110

Interviewer: So have they asked since like your very first visit or was your first visit with this pregnancy, the only time they asked.

167

00:17:52.800 --> 00:17:56.400

Participant: They asked they asked all the time like this, every single day.

168

00:17:57.510 --> 00:18:01.350

Participant: Even whenever you post my mistake or even if you didn't.

169

00:18:03.600 --> 00:18:04.320

Like they always.

170

00:18:07.440 --> 00:18:07.710

Interviewer: Like.

171

00:18:09.060 --> 00:18:11.070

Interviewer: To bring up with them about it.

172

00:18:13.470 --> 00:18:18.720

Participant: not about whether it's a bad thing, but other questions I have for the love always it.

173

00:18:19.680 --> 00:18:20.040

Okay.

174

00:18:21.600 --> 00:18:32.010

Interviewer: um do you think talking to your doctor about marijuana is different than talking to them about like other substances like alcohol or tobacco or other drugs is it is there a difference.

175

00:18:33.240 --> 00:18:37.710

Participant: Oh no I never had, I never thought I never touched my back.

176

00:18:38.430 --> 00:18:41.970

Participant: mm hmm so I don't really know what that was but.

177

00:18:43.740 --> 00:18:44.580

Participant: yeah oh man.

178

00:18:45.630 --> 00:18:50.520

Interviewer: In your opinion, should doctors like deal with those different substances in different ways, when.

179

00:18:50.640 --> 00:18:52.830

Interviewer: Like depending on what someone tells them they're using.

180

00:18:53.640 --> 00:18:56.280

Interviewer: Like how should they handle it differently different.

181

00:18:57.690 --> 00:19:10.650

Participant: Oh think I should be different, I think it should be treated the same way, unless it's like coke or crack or pills or other stuff like that that stuff up as ministry second a little bit more serious than waiter tobacco.

182

00:19:12.300 --> 00:19:13.230

Interviewer: me more about that.

183

00:19:14.310 --> 00:19:29.700

Participant: Oh no I feel like like concave kills here with other stuff is like 10 times worse than I feel like week because I know people use like that's up to like called learners but people just use that just to get ahead.

184

00:19:30.120 --> 00:19:35.940

Participant: And the way people use it to conduct nerves and just begin to have, but I feel like we doesn't have the same effect or you.

185

00:19:36.330 --> 00:19:38.370

Participant: know other other stuff will have an effect or you.

186

00:19:40.170 --> 00:19:55.860

Participant: didn't make sense, like I know i've seen some people around me get addicted to crack and a whole bunch of other stuff and I just seen how it affects people because they started off where we, and then I would see them start boy way more crazy than what they will go on before.

187

00:19:58.230 --> 00:20:01.710

Participant: So I feel like that other session we've taken more seriously, especially while you're pregnant.

188

00:20:04.260 --> 00:20:10.530

Interviewer: What do you think would help young pregnant women like yourself get more information about marijuana in tobacco use during pregnancy.

189

00:20:11.220 --> 00:20:20.460

Participant: up for like low reception as social workers they always tell us like we talked it, I, like you could sell them like if you have to censor.

190

00:20:20.880 --> 00:20:30.720

Participant: they'll help you get stuff then they'll give you numbers to call the doctors give you numbers to call but there's really nothing, where I think they could do.

191

00:20:33.360 --> 00:20:36.060

Interviewer: Tell me more about what social work has been able to do.

192

00:20:37.080 --> 00:20:50.730

Participant: So the social workers there's like four six little girl never having to do is like they give you numbers to call for like housing first them with like a whole bunch of stuff that that you would need in a pregnancy, they help you get.

193

00:20:53.550 --> 00:20:54.240

Participant: In do you think.

194

00:20:54.600 --> 00:20:56.700

Participant: The numbers to call it everything.

195

00:20:58.920 --> 00:21:08.040

Interviewer: You think like your doctor social worker or midwives giving you the numbers to call is enough or do you think that still went to kind of unapproachable and there should be.

196

00:21:08.340 --> 00:21:19.020

Participant: no need for me it always helps like the never ending to help getting my supportive housing things they gave me number to call and then the people I call day help.

197

00:21:20.370 --> 00:21:22.230

Participant: So that they stepped out.

198

00:21:24.090 --> 00:21:26.460

Participant: But the number that they give out definitely help.

199

00:21:27.810 --> 00:21:39.360

Participant: them out so it's like you're really going to worry about and that helping but the only way I will actually help you out is, if you actually check causing those people, they just give numbers to and they'll be like oh yeah i'll do it.

200

00:21:40.500 --> 00:21:41.850

Participant: And it'll get nowhere so.

201

00:21:43.410 --> 00:21:47.790

Participant: Actually, called the numbers, if you ask me for anything to try work.

202

00:21:49.740 --> 00:21:52.440

Interviewer: What else do you think would help young women get information.

203

00:21:55.440 --> 00:21:55.740

So we.

204

00:21:58.170 --> 00:22:02.670

Participant: were all just like a lot of girls, like me, that are pregnant ahead babies after like.

205

00:22:03.240 --> 00:22:18.210

Participant: Anybody that can help you whether it's other pregnant women or other adults or anything like that that could give you as much information as possible well but they're really like no specific thing I can say to like help us young woman out while being pregnant.

206

00:22:20.970 --> 00:22:26.820

Interviewer: um i'm just I guess i'm wondering based on your common do you have a way that you like connect with.

207

00:22:29.070 --> 00:22:30.060

Participant: So.

208

00:22:31.800 --> 00:22:40.980

Participant: let's so basically I don't really know what the Bible says pregnant that's like my age or younger anything like whenever I was pregnant with my daughter.

209

00:22:44.250 --> 00:22:50.790

Participant: Like at the end of my pregnancy, the girl has my God daughter she just ended up getting pregnant as as in my friend.

210

00:22:51.660 --> 00:22:58.740

Participant: And then she was like a mother to pregnant so she was just pregnant and then another friend of mine that I went to school with.

211

00:22:59.850 --> 00:23:08.640

Participant: She was pregnant like three four months pregnant with her daughter right whenever I had my daughter so it's just like we really didn't connect much because.

212

00:23:09.360 --> 00:23:17.430

Participant: At that time I was already almost at the end of my pregnancy and then Now I know there's a girl I go to school with and she's pregnant.

213

00:23:18.060 --> 00:23:24.690

Participant: But I don't talk to her like that, like me, are you see close but outside her no more because, just like to activate a new law that but.

214

00:23:25.140 --> 00:23:34.800

Participant: If anybody is able to connect with it probably would have been hard because we're the same age she's pregnant pregnant Emily like two and a half, three months ahead of her.

215

00:23:37.230 --> 00:23:46.560

Participant: So I mean connector while they're pregnant women it's just like there's really nothing for me to connect with them because i've been different like we all been a different sense.

216

00:23:47.910 --> 00:24:01.080

Participant: But my baby sister she's 26 but, and I could say for a fact I connected with her to my pregnancy, because we'd have to have my daughter she got pregnant with her son and then.

217

00:24:01.890 --> 00:24:11.550

Participant: It was like I was actually able to talk to her about everything that's happening my pregnancy, she didn't want to text me about her pregnancy, so there was able to connect with her a lot more.

218

00:24:11.970 --> 00:24:19.170

Participant: On the pregnancies, even a new and separate says there's just like I was actually able to like connect with her in understand certain things.

219

00:24:21.660 --> 00:24:22.410

Participant: If that makes sense.

220

00:24:24.060 --> 00:24:32.130

Interviewer: Do you wish there were like more ways or resources to connect with other young women going through the same things at the same time.

221

00:24:35.310 --> 00:24:48.270

Interviewer: Like you wish that instead of like meeting people in your personal life, you were pregnant that you could kind of like go to like a support group or meet people online like would you want to seek out other young.

222

00:24:48.840 --> 00:24:51.270

Participant: Definitely, I definitely will want to do that so.

223

00:24:51.630 --> 00:25:00.660

Participant: projects that like I know they do stuff like that, with other young pregnant women, but I have to bed with a sign the paper because she said to me in the link on the email then.

224

00:25:01.380 --> 00:25:09.780

Participant: don't ask them to the side, but they do stuff like that, and then actually getting like i'm willing to do the meeting the other pregnant women that are around my age.

225

00:25:10.230 --> 00:25:18.600

Participant: Because it's like consuming I want to know what what they're kind of going through and for like to get those those type of advice, because I was pregnant before.

226

00:25:19.440 --> 00:25:28.290

Participant: And had they'll give me advice like just stuff like that, like i'm i'd be willing to do it, like, I want to do it and it's just like that's something I know what it.

227

00:25:30.720 --> 00:25:34.740

Interviewer: sounds like that's an important source of information and support for you.

228

00:25:37.920 --> 00:25:48.840

Interviewer: So any anything you haven't already mentioned that you think would be useful to like get more information, even things that don't exist just things you can like think of that would be nice to have.

229

00:25:52.410 --> 00:25:57.240

Participant: Another have support programs with you like diapers and wipes and stuff like that, but I feel like.

230

00:25:58.890 --> 00:26:15.030

Participant: It would be better like if there was ones that would help you get like food for the baby clothes and stuff like that, because it'd be a lot more keeping it fresh as women and women just internal that are pregnant, that are China still going to get stuff together for the baby.

231

00:26:17.160 --> 00:26:26.190

Participant: If people would like if there was more like stuff like that open to help us get more stuff like that that'd be good, but other than that nothing I really think of.

232

00:26:26.970 --> 00:26:38.040

Interviewer: Okay, and what do you think of healthcare providers can do to help young women feel more comfortable talking to them about either marijuana or tobacco use, depending on their situation.

233

00:26:40.260 --> 00:26:48.690

Participant: It just depends on how they talk to them about it, like if they have personal a certain way, then it's like it could go good or bad it just depends on how the doctor approaches it.

234

00:26:51.120 --> 00:26:52.740

Participant: Dr treats them about when if.

235

00:26:52.740 --> 00:26:54.150

Participant: They say they do or they don't.

236

00:26:55.770 --> 00:26:57.600

Interviewer: Tell me more how should they approach it.

237

00:26:58.530 --> 00:26:59.220

Participant: I feel like.

238

00:27:00.420 --> 00:27:09.150

Participant: it's not they should come into and talk to you on like one On one level not like as a friend that as an adult learning somebody like to take your kid from you.

239

00:27:09.510 --> 00:27:17.850

Participant: As like somebody that's actually trying to figure out what's going on in your life in see what's going on, because under his doctors that I had a doctor in.

240

00:27:18.240 --> 00:27:26.250

Participant: She was extremely judge because whenever I found that I was pregnant added that I was literally almost in my second time, Mr issue is extremely judge.

241

00:27:26.970 --> 00:27:37.200

Participant: So I feel like they should commit to as like not not on a friend level, but not on a lot on a friend level but not.

242

00:27:37.890 --> 00:27:52.260

Participant: As like a disrespectful of who are as effective of like somebody that you can actually talk to and explain to them what's going on and why, if you were using it, or if you're not using it like for like there's like no love, we could be on just like a person, a personal level.

243

00:27:55.980 --> 00:27:58.200

Interviewer: not sure if you can explain more if you can like.

244

00:27:58.890 --> 00:28:06.090

Interviewer: What what how what kind of exactly things would they do to to show that they're approaching on like a person, the person level.

245

00:28:08.490 --> 00:28:18.390

Participant: of me I guess I don't know how to explain that part but like it, because you just had to like see it and then kind of see how the but like if they come talking to you like.

246

00:28:19.890 --> 00:28:29.100

Participant: Oh, my God it's like this, so not been like us so girl what you do today, like like that, but not be like oh so do you do this, that or third like.

247

00:28:29.430 --> 00:28:40.620

Participant: commit to and actually have a conversation with you, we like so anything, though, like were you doing this thing the third and be calm about it, not like, not on a friendly level, but not on a doctorate level.

248

00:28:42.390 --> 00:28:44.730

Participant: If that makes sense and it's hard to explain.

249

00:28:44.910 --> 00:28:45.990

Interviewer: I agree.

250

00:28:47.100 --> 00:28:50.580

Interviewer: Tell me more about approaching something on a doctor level what does that mean do.

251

00:28:51.180 --> 00:28:55.950

Participant: So, Dr love, I feel like gases is real judging just in general, and I feel like.

252

00:28:57.780 --> 00:29:10.110

Participant: How do I explain this so not not catching it attitude, because I know doctors always have edited with no matter what the situation is this had that little ton of attitude, so I feel like.

253

00:29:10.710 --> 00:29:22.500

Participant: Get maybe they lost the attitude and just was like Karma whatever it might take some time but it's hard to explain because it's like you have to like has certain doctors for it to be like that.

254

00:29:24.480 --> 00:29:29.070

Interviewer: And you also mentioned it matters, how the provider responds to.

255

00:29:30.120 --> 00:29:30.660

Interviewer: respond.

256

00:29:32.400 --> 00:29:42.480

Participant: I wouldn't say I wouldn't respond probably I wouldn't say what them to respond, a certain way, but it's just like if they would not be so judging like.

257

00:29:43.710 --> 00:29:55.800

Participant: Like say say I would sell them like I was using for like two months instead of pregnancy, a she like Oh well, this, then the third just imagine somebody coming at you, and be a road judging your neck will feel comfortable to talk to them.

258

00:29:56.610 --> 00:29:58.350

Participant: So, like if they would turn down.

259

00:29:58.380 --> 00:30:04.440

Participant: Like the judging this and be like commonly talked to you and just not have its own inner voice or.

260

00:30:04.950 --> 00:30:16.020

Participant: You know, like you could get a look at somebody and they like they're just judging you after look or whatever like they can look at you a certain way, like not look into and judging wave or not do any of it, it might be better.

261

00:30:22.410 --> 00:30:34.380

Interviewer: What do you think about i'm not sure if we talked about this already about like using the Internet as an information source they don't think we really talked about that it's not something where you go to answer questions about marijuana.

262

00:30:35.730 --> 00:30:37.470

Participant: I usually just ask for around me.

263

00:30:38.070 --> 00:30:38.340

Okay.

264

00:30:39.570 --> 00:30:42.180

Interviewer: And I I wouldn't talk a little bit more about your experience.

265

00:30:42.930 --> 00:30:44.040

Interviewer: Your own experience was.

266

00:30:44.130 --> 00:30:44.580

marijuana.

267

00:30:46.200 --> 00:30:50.880

Interviewer: So what like lead you to trying it like, why did you try it at the time that you.

268

00:30:52.020 --> 00:30:59.640

Participant: Alright, so the gripes we got the vpn from she did say she because I was talking to her like I was real close with her and.

269

00:31:00.210 --> 00:31:10.110

Participant: At this time, I was loud, just so stressed out, I mean something to like charity water stress, because they gave me is that it but it's in before I pray for my daughter, but it wasn't working.

270

00:31:10.920 --> 00:31:19.110

Participant: And so I was just like I need something that commoners, though, like I don't want to smoke out of this I don't want to do that and then she was like well then.

271

00:31:19.470 --> 00:31:33.480

Participant: You should try to read pink because it's not weed and you're going to worry about it it's just thc you know, like is it going to affect me in the same way, she said, I might just make you more calmer, and this, then a third so that's what really got me to try that.

272

00:31:36.030 --> 00:31:36.720

Interviewer: Was it like.

273

00:31:37.500 --> 00:31:37.920

Interviewer: You said.

274

00:31:38.880 --> 00:31:39.330

Participant: Would you say.

275

00:31:39.720 --> 00:31:41.400

Interviewer: What was it like when you first used it.

276

00:31:42.720 --> 00:31:49.410

Participant: were not first used it, it was to me it was like it was a good feeling, but it wasn't a good feeling, it was like.

277

00:31:50.670 --> 00:31:54.870

Participant: It was like I don't know how to explain it, it was like it was weird like it was like a really weird.

278

00:31:57.930 --> 00:31:59.010

Participant: And it was my.

279

00:32:01.140 --> 00:32:08.850

Participant: Point is it's like I didn't want to do it, but it I know it helped so that I kept doing it for a little bit, because I know it helped me.

280

00:32:09.450 --> 00:32:11.040

Participant: But I would only do it like.

281

00:32:11.490 --> 00:32:19.500

Participant: Twice once like twice a week or something like that not even like it'd be twice every two weeks, like I wouldn't do it a lot, because the way I don't like to fill in.

282

00:32:24.420 --> 00:32:33.060

Interviewer: Just the last question for me has your involvement in this study impacted how you feel about marijuana tobacco at all.

283

00:32:36.600 --> 00:32:43.530

Participant: So far, the same way about it, no matter what the situation is, I still collect payment people shouldn't use it for people that don't need us to lose it.

284

00:32:44.520 --> 00:32:47.520

Interviewer: Okay, is there anything I didn't ask that you'd want to add.

285

00:32:51.090 --> 00:32:54.030

Interviewer: i'm gonna go ahead and turn off the recording here.
